# NGFR induces melanoma invasion and immunotherapy resistance through myosin light chain 2 modulation

**DOI:** 10.1038/s44318-026-00803-2

**Published:** 2026-05-26

**Authors:** Laura Nogués, Vanesa Santos, Juan García-Agullo, Susana García-Silva, Oscar Maiques, Alberto Hernández-Barranco, Marina S Mazariegos, Manuel Pérez-Martinez, Raúl Torres-Ruíz, Annalisa Saltari, Patrick Turko, María Mannino, Ali Nejatie, Hassan Nassour, Diego Megías, Isabel Peset, Carmen Blanco-Aparicio, Sonia Martínez, Mitchell Levesque, H Uri Saragovi, Victoria Sanz-Moreno, Héctor Peinado

**Affiliations:** 1 Microenvironment and Metastasis Laboratory, Department of Tumor Biology, Spanish National Cancer Research Center (CNIO), Madrid, Spain; 2 Department of Molecular Biology, University Institute of Molecular Biology (IUBM-UAM), Centre for Molecular Biology “Severo Ochoa” (CBMSO) UAM-CSIC, Autonomous University of Madrid, Madrid, Spain; 3 Instituto de Investigación Sanitaria La Princesa, Madrid, Spain; 4 Melanoma Laboratory, Department of Tumor Biology, Spanish National Cancer Research Center (CNIO), Madrid, Spain; 5 Cytoskeleton and Cancer Metastasis Laboratory, The Breast Cancer Now Toby Robins Research Centre, The Institute of Cancer Research, London, SW3 6JB UK; 6 Centre for Tumour Microenvironment, Barts Cancer Institute, Queen Mary University of London, London, UK; 7 Department of Oncology, University of Lausanne, Ludwig Institute for Cancer Research, Lausanne, Switzerland; 8 Division of Pediatrics, Department of Clinical Sciences, Lund University, Lund, Sweden; 9 Cofocal Microscopy Unit, Biotechnology Programme, Spanish National Cancer Research Center (CNIO), Madrid, Spain; 10 Molecular Cytogenetics Unit, Human Cancer Genetics Programme, Spanish National Cancer Research Center (CNIO), Madrid, Spain; 11 Department of Dermatology, University of Zurich, University Hospital Zurich, Zurich, Switzerland; 12 UOC di Dermatologia, Dipartimento di Scienze Mediche e Chirurgiche Addominali ed Endocrino Metaboliche, Fondazione Policlinico Universitario A. Gemelli - IRCCS, Rome, Italy; 13 Dermatologia, Università Cattolica del Sacro Cuore, Rome, Italy; 14 Department of Pharmacology and Therapeutics, McGill University, Montréal, Quebec Canada; 15 Centre for Translational Research in Cancer-Jewish General Hospital-Lady Davis Institute, Montréal, Quebec Canada; 16 Advanced Optical Microscopy – ISCIII Madrid, Madrid, Spain; 17 Experimental Therapeutics Programme, Spanish National Cancer Research Center (CNIO), Madrid, Spain; 18 Microenvironment and Metastasis Laboratory. Department of Immunology and Oncology, Centro Nacional de Biotecnología. Consejo Superior de Investigaciones Científicas (CNB-CSIC), Madrid, Spain

**Keywords:** Cancer, Immunology

## Abstract

Immunotherapy has reshaped melanoma treatment, yet the majority of patients fail to respond or develop resistance. Nerve growth factor receptor (NGFR/p75NTR/CD271) has been linked to melanoma aggressiveness and therapeutic resistance, but the underlying mechanisms remain unclear. Here, we demonstrate that pharmacologic inhibition of NGFR with THX-B significantly reduces distant metastasis and restores sensitivity in immunotherapy-resistant tumors. THX-B treatment also enhances intratumoral CD8⁺ T-cell infiltration. Further, we show that immune-resistant melanomas acquire an invasive, ameboid phenotype characterized by elevated NGFR, PD-L1, and activation of non-muscle myosin light chain 2 (NM II; MLC2). Mechanistically, NGFR regulates RhoA/ROCK-dependent MLC2 phosphorylation and proteasomal stabilization. Analysis of melanoma patient samples reveals that high NGFR expression correlates with reduced progression-free survival following immunotherapy. Moreover, NGFR and phosphorylated MLC2 are consistently enriched at the invasive fronts of primary tumors, metastases, and in immunotherapy-resistant cell lines from melanoma patients. Altogether, these findings identify the NGFR–MLC2 axis as a mediator of invasive immune resistance and support NGFR inhibition as a strategy to enhance immunotherapy efficacy.

## Synopsis

Nerve growth factor receptor (NGFR; aka p75NTR, CD271) expression correlates with skin cancer aggressiveness and therapeutic resistance, but the underlying mechanisms remain unclear. This study demonstrates synergies between NGFR inhibition and immunotherapy in countering melanoma progression, and uncovers non-muscle myosin II (NM II; MLC2) as a post-translational target of NGFR.

- Pharmacological NGFR inhibition reduces metastatic burden, increases intratumoral CD8⁺ T-cell infiltration, and enhances the response to PD-1/PD-L1 blockade in preclinical melanoma models.
- NGFR promotes an invasive, amoeboid state in melanoma cells through RhoA–ROCK-dependent MLC2 phosphorylation.
- Combined NGFR and ROCK inhibition decreases MLC2 stability, countering actomyosin-dependent invasiveness.
- NGFR and p-MLC2 are enriched at the invasive front of primary and metastatic human melanomas.
- High expression of NGFR in tumors correlates with shorter progression-free survival in melanoma patients treated with immunotherapy.

## Abstract (web summary)

Targeting Nerve Growth Factor Receptor counters skin cancer progression, preventing post-translational modulation of non-muscle Myosin II.

## Graphical abstract

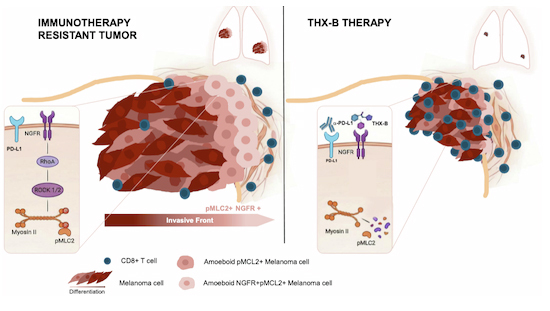

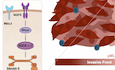

## Introduction

In recent years, the incidence and mortality of melanoma have increased exponentially, seriously affecting human health. Over 90% of melanoma patients survive if they are diagnosed at their earliest stages, but treatments fail in patients diagnosed with distant metastasis (30% efficacy at stage IV) (Homet Moreno et al, 2016; Mishra et al, 2018; Saginala et al, 2021). For these patients, immunotherapy (IT) against checkpoint inhibitors such as Programmed cell Death protein 1 (PD-1), Programmed Death-Ligand 1 (PD-L1) and/or Cytotoxic T-lymphocyte associated protein 4 (CTLA4) has remarkably improved patient survival (Queirolo et al, 2019; Topalian et al, 2014). However, 60–80% of the patients still do not respond or develop acquired resistance, side effects and tumor relapse in the form of metastatic disease (Arnaud-Coffin et al, 2019; Brahmer et al, 2021; Hogan et al, 2018; Topalian et al, 2014). Growing evidence suggests that melanoma cells can elicit non-genetic reprogramming, adopting multiple phenotypic states impacting therapy efficiency (Rambow et al, 2019). In this sense, phenotypic plasticity has arisen as one of the main drivers of resistance regardless of the therapy (Arozarena and Wellbrock, 2019; Haas et al, 2021). This phenomenon is characterized by the ability of tumor cells to adapt to different needs and involves the transition from a proliferative state (MITF^+^) towards a dedifferentiated invasive phenotype led by the neural crest marker Nerve Growth Factor Receptor (NGFR) (Arozarena and Wellbrock, 2019; Mehta et al, 2018).

NGFR is considered a key regulator of phenotype switching in both targeted and immunotherapy resistance (Boshuizen et al, 2020; Lehraiki et al, 2015; Restivo et al, 2017). Also known as p75NTR or CD271, NGFR belongs to the tumor necrosis factor receptor (TNFR) superfamily, acting as a low-affinity common receptor for multiple neurotrophins (NGF, BDNF, NT3, and NT4) and proneurotrophins. NGFR lacks catalytic activity and elicits its functions by interacting with different co-receptors and intracellular partners, triggering the activation of either JNK, p-NFKB and/or RhoA signaling pathways (Hempstead, 2005). Therefore, NGFR can modulate cell fate by controlling processes as diverse as survival, invasion, migration and stemness (Boiko et al, 2010; Hempstead, 2005; Radke et al, 2017; Redmer et al, 2014). In melanoma, NGFR is heterogeneously expressed but associated with tumor and metastasis-initiating potential (Boiko et al, 2010; Civenni et al, 2011). Besides this intrinsic regulation, NGFR can also modulate tumor and distal microenvironments to favor tumor progression (Boshuizen et al, 2020; Garcia-Silva et al, 2021; Lehmann et al, 2023). In addition, high expression of NGFR is associated with resistance to targeted therapy (Lehraiki et al, 2015; Rambow et al, 2018) and IT (Boshuizen et al, 2020). Importantly, high NGFR expression is characterized by elevated PD-L1 levels (Liu et al, 2021), and the PD-L1-NGFR axis additively suppresses the activation of melanoma-specific cytotoxic T lymphocytes (Furuta et al, 2014). This is consistent with recent studies demonstrating that NGFR regulates anti-tumor immunity mediated by T and NK cells (Boshuizen et al, 2020; Lehmann et al, 2023).

Considering this body of evidence, several therapeutic strategies modulating NGFR levels have been developed during the last few years. For example, the use of small molecules targeting the transmembrane domain of NGFR, or a short β-amyloid-derived agonist peptide (Aβ(25-35)), induces cell death activation and reduces melanoma tumor growth (Goh et al, 2018; Saltari et al, 2021). Similarly, the use of anti-NGFR antibodies has shown success as monotherapy to target cancer stem cells (Morita et al, 2019) or, when combined with α-CD47, an anti-phagocytic signal, has shown anti-tumor and anti-metastatic effects in immunodeficient models (Ngo et al, 2016). In addition, reducing NGFR expression indirectly when using a heat shock protein inhibitor of HSP90 (Boshuizen et al, 2020), MNK1/2 inhibitors (Huang et al, 2021) or Ranolazine (Redondo-Munoz et al, 2023) led to improved anti-tumor immune cell responses. In this sense, we have previously found that treatment with the NGFR small molecule inhibitor THX-B (Bai et al, 2010; Garcia-Silva et al, 2021; Platon-Corchado et al, 2017) reduced melanoma lymph node metastasis in immunocompetent mice (Garcia-Silva et al, 2021), but the mechanism involved was not defined.

Here, we report that NGFR inhibition by THX-B, used in combination with current immunotherapies, reduces both tumor growth and distal metastasis. Moreover, THX-B treatment reverses immunotherapy resistance and increases intratumoral CD8^+^ T cell infiltration. Additionally, we observed that immune-resistant tumors shift from a proliferative to an invasive phenotype characterized by the upregulation of NGFR, PD-L1 and activation of the non-muscle myosin light chain 2 (MLC2). Mechanistic studies revealed that NGFR is expressed at the invasive front (IF) of melanoma tumors, and regulates MLC2 phosphorylation (pMLC2) via the RhoA–ROCK pathway. In addition, our study uncovers a previously unrecognized post-translational mechanism in which NGFR and ROCK cooperatively stabilize myosin II protein. Importantly, histological analysis of melanoma patient samples revealed a significant correlation between higher NGFR expression and reduced progression-free survival (PFS) following immunotherapy treatment. Finally, we found high NGFR and pMLC2 at the IF of primary tumors and metastasis of melanoma patients, suggesting that this axis is critical for regulating invasion in melanoma. Our data suggest that NGFR could be used to identify IT-resistant tumors and provide evidence for the use of NGFR inhibitors to improve immunotherapy efficiency and revert resistance in melanoma.

## Results

### NGFR blockade decreases melanoma metastasis

**Figure EV1:**
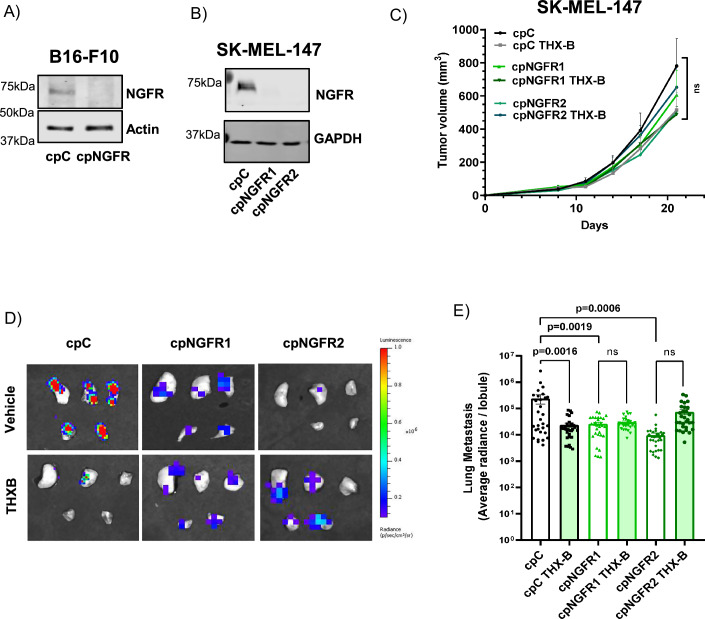
THX-B decreases lung metastasis of human SK-MEL-147 melanoma tumors. (**A**) Representative western blot showing NGFR expression in B16-F10 cpControl (cpC) and cpNGFR cells. (**B**) Representative western blot showing NGFR expression in SK-MEL-147, cpControl (cpC), and cpNGFR1/2 cells. (**C**) Tumor growth of SK-MEL-147 cpControl, cpNGFR1, or cpNGFR2 -GFPLuc tumors on Nude mice upon twice a week i.p. treatment with THX-B (5 mg/kg) or vehicle, starting at D7. Data were Mean + SEM of *n* = 6 mice/group. Two-way ANOVA with Tukey multi-comparison test was applied. ns, not significant. (**D**) Representative images of lung metastasis. (**E**) Quantification of the ex vivo SK-MEL-147 GFPLuc lung metastatic burden by measuring the average radiance per lobule by IVIS at endpoint. Two-way ANOVA were used to analyze tumor growth (**C**). Average radiance/lobule was calculated by one-way ANOVA with Tukey correction for multi-comparison (**E**). ns, not significant. Source data are available online for this figure.

**Figure 1:**
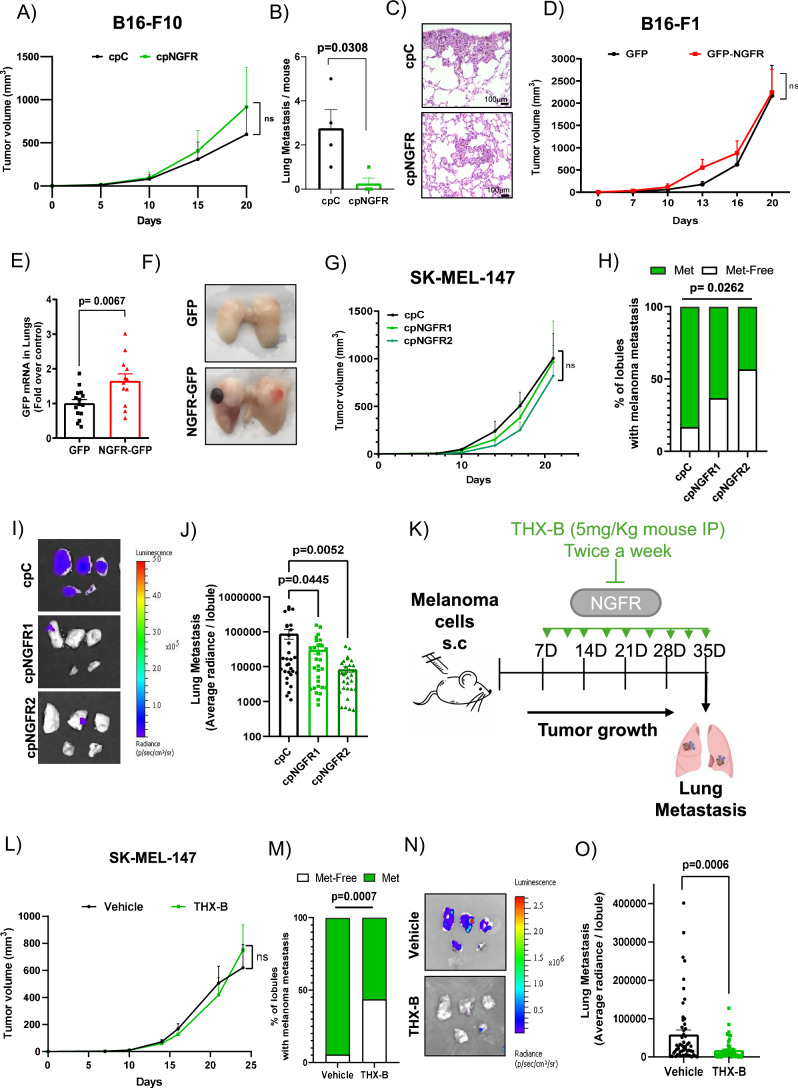
NGFR blockade reduces melanoma metastasis. (**A**) Tumor growth analyzed in mice injected in the flank with 200,000 control (cpC) or cpNGFR B16-F10 cells. Tumor growth was measured every 5 days. (**B**) Number of lung metastases per animal in the experiment described in (**A**). Three sections were analyzed per lung (*n* = 4 mice/group). (**C**) Representative images of lung metastases in animals from the experiment described in (**A**). (**D**) Tumor growth was analyzed in mice injected in the flank with 10^6^ B16-F1-GFP or overexpressing NGFR-GFP(Garcia-Silva et al, 2021). (**E**) Lung metastasis addressed by detection of GFP mRNA expression of ex vivo digested lungs at endpoint (D21) and represented as fold increase of the control group. (*n* = 12–15 mice/group from three independent experiments). (**F**) Representative images of lungs with B16-F1 (wt or NGFR-OE) metastases from the experiment described in (**D**). (**G**) Tumor growth analyzed in nude mice injected in the flank with 300,000 control (cpC) or cpNGFR1/2 SK-MEL-147-GFPLuc cells. Tumor growth was measured every 5 days. (**H**) Percentage of lung lobules with melanoma metastases detected by luciferase expression at the IVIS system. (**I**) Representative pictures and (**J**) quantification of Luc expression (average radiance/lobule) by IVIS. (*n* = 6 mice/group). (**K**) Schematic of THX-B treatment in melanoma subcutaneous xenograft models. Briefly, 300,000 human SK-MEL-147-GFPLuc melanoma cells were subcutaneously injected into the flank of nude mice. After 7 days, when tumors reached around 100 mm^3^, intraperitoneal THX-B treatment was initiated at 5 mg/kg, twice a week. Tumor growth was monitored every 2–3 days, and mice were sacrificed when tumors reached 1000 mm^3^ (~1 month later). Lungs were analyzed for metastases detection by IVIS ex vivo. (**L**) Tumor growth of SK-MEL-147-GFPluc tumors on Nude mice upon twice a week i.p. treatment with THX-B (5 mg/Kg) or vehicle, starting at D7. (**M**) Percentage of lobules with melanoma metastases detected by luciferase expression at the IVIS system. (**N**) Representative images of lung metastases. (**O**) Quantification of the ex vivo SK-MEL-147-GFPLuc lung metastatic burden by measuring the average radiance per lobule by IVIS at endpoint. (*n* = 11–12 mice/group from two independent experiments). Data were mean + SEM. Two-way ANOVA were used to analyze tumor growth (**A**–**D**, **G**–**L**), *p* values of the % of lobules affected, and the average radiance/lobule was calculated by unpaired *t*-test (**B**–**E**, **H**–**J**, **M**–**O**). ns, not significant. Source data are available online for this figure.

Despite several attempts to modulate NGFR activity in melanoma showing an effect on tumor growth (Boshuizen et al, 2020; Goh et al, 2018; Saltari et al, 2021), there is little evidence of the impact of targeting NGFR on the metastatic process. To define the relevance of NGFR in melanoma progression, we performed CRISPR/Cas9-mediated NGFR knockout (KO) in the highly metastatic B16-F10 cells (Fig. EV1a) and injected them subcutaneously into syngeneic immunocompetent mice. Subcutaneous injection of B16-F10-GFPLuc NGFR KO (cpNGFR) cells did not impact significantly on tumor growth (Fig. 1A). However, lung metastasis were significantly reduced in mice injected with NGFR-KO cells (Fig. 1B,C). To confirm the relevance of NGFR expression in the B16 model, we overexpressed NGFR in the low metastatic cell line B16-F1 (Fidler and Nicolson, 1976) cell line. Its subsequent subcutaneous implantation in immunocompetent mice enhanced lung metastasis without affecting primary tumor growth (Fig. 1D–F). To validate our results in human models, we subcutaneously injected metastatic human melanoma SK-MEL-147 cells (expressing high NGFR levels (Garcia-Silva et al, 2021) and SK-MEL-147 NGFR KO cells (Fig. EV1b) into nude mice. NGFR depletion did not affect tumor growth (Fig. 1G) but significantly compromised lung metastasis development (Fig. 1H–J).

We next evaluated the effectiveness of pharmacological NGFR inhibition using the NGFR small molecule inhibitor THX-B (Bai et al, 2010) in distal metastasis (see Fig. 1K for a scheme), since we have previously demonstrated that THX-B blocked early dissemination of melanoma cells to lymph nodes (Garcia-Silva et al, 2021). We found that THX-B treatment had no significant effect on primary tumor growth (Fig. 1L), but it significantly reduced lung metastasis (Fig. 1M–O). To test whether THX-B activity in vivo depends on NGFR, we compared its effects in tumors that express NGFR versus NGFR-knockout tumors. THX-B reduced metastatic burden in NGFR-expressing SK-MEL-147 tumors, whereas it did not further affect tumor growth or metastasis in mice bearing NGFR-KO SK-MEL-147 tumors (cpNGFR1 or cpNGFR2) (Fig. EV1C–E). These results support the idea that THX-B limits melanoma metastasis in an NGFR-dependent manner and are consistent with genetic and pharmacological targeting of NGFR converging on reduced metastatic dissemination.

### Pharmacological NGFR inhibition improves the immune checkpoint blockade efficacy

**Figure EV2:**
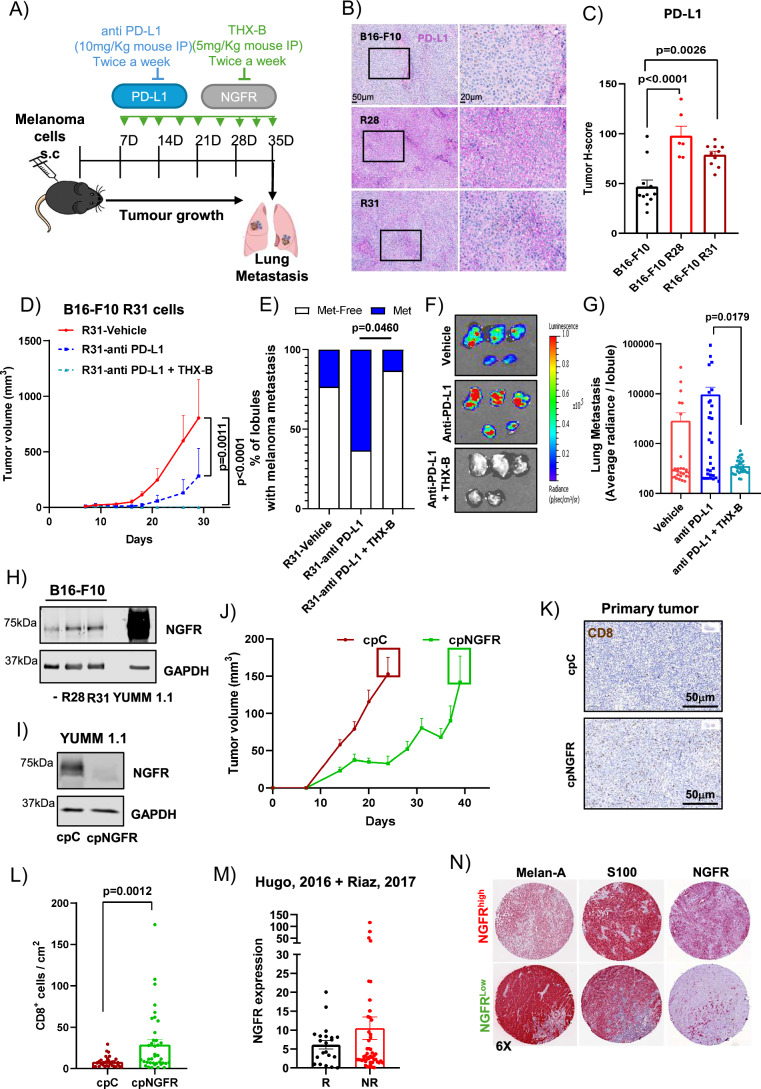
Targeting NGFR restores immunotherapy responsiveness and promotes intratumoral CD8⁺ T-cell recruitment of IT-resistant tumors. (**A**) Scheme of the experiment performed to study the effect of the THX-B and anti-PD-L1 combination therapy in melanoma subcutaneous immunocompetent models. Dosages of treatment are indicated. Treatments were administrated as monotherapy or in combination intraperitoneally twice a week beginning 7 days after tumor cell injection. (**B**,** C**) PD-L1 expression was measured by IHC of B16-F10 control and anti-PD-L1-resistant (R28 and R31) tumors. *n* = 6–11 different fields from 3 to 6 tumors /group. (**B**) Representative IHC image of PD-L1 staining in different groups. Black square indicates magnified area in the right. (**C**) Qupath quantification of intra-tumor PD-L1 expression. Data were mean + SEM. (**D**–**G**) B16-F10-R31 cells (200,000) were injected into the flank of B6 mice. On day 7, treatment started as indicated in (**A**) (*n* = 6 mice/group). (**D**) Tumor growth curves showing mean + SEM. (**E**) Percentage of lung lobules with melanoma metastases. (**F**) Representative IVIS image. (**G**) Quantification of the average radiance/lobule. Data were mean + SEM. (*n* = 6 mice/group). 1 (**H**) NGFR expression in melanoma cell lines with acquired (B16-F10 R28 and R31) or intrinsic (Yumm1.1) immunotherapy resistance. A representative Western blot is shown. (**I**) Representative western blot of the efficient KO of NGFR in Yumm1.1 cells. (**J**) Tumor growth of cpC and cpNGFR YUMM1.1 tumors in B6 mice sacrificed at the same tumor size (100–300 mm^3^). The square indicates the mean size of the tumors analyzed by IHC. Data were mean + SEM of *n* = 12 tumors from 6 mice (injected in both flanks). (**K**) Representative image of CD8^+^ IHC staining in tumors from (**J**). Scale bar (50 μm) is indicated. (**L**) The number of CD8^+^ T cells was calculated from 3 to 4 consecutive slides/tumor using QuPath-0.5.1 software and normalized to the area of the tumor. Data were mean + SEM. (**M**) NGFR expression in responders (R) or non-responders (NR) to anti-PD-1 therapies from datasets GSE78220 (Hugo et al, 2016) and GSE91061 (Riaz et al, 2017). Mean + SEM of *n* = 70 patients is represented. (**N**) Representative immunohistochemistry of TMAs from melanoma patients under immunotherapy. Serial sections stained for Melan‑A, S100 and NGFR are shown. Upper panel: full TMA 258. Red and green boxes indicate representative cores with high or low NGFR expression, respectively. Objective magnification as indicated. (**N**) Statistical analyses performed were one-way ANOVA (**C**), Two-way ANOVA (**D**) and unpaired Student *t-*test (**E**, **G**, **I**). Tukey correction was used for multiple comparison. Source data are available online for this figure.

**Figure 2:**
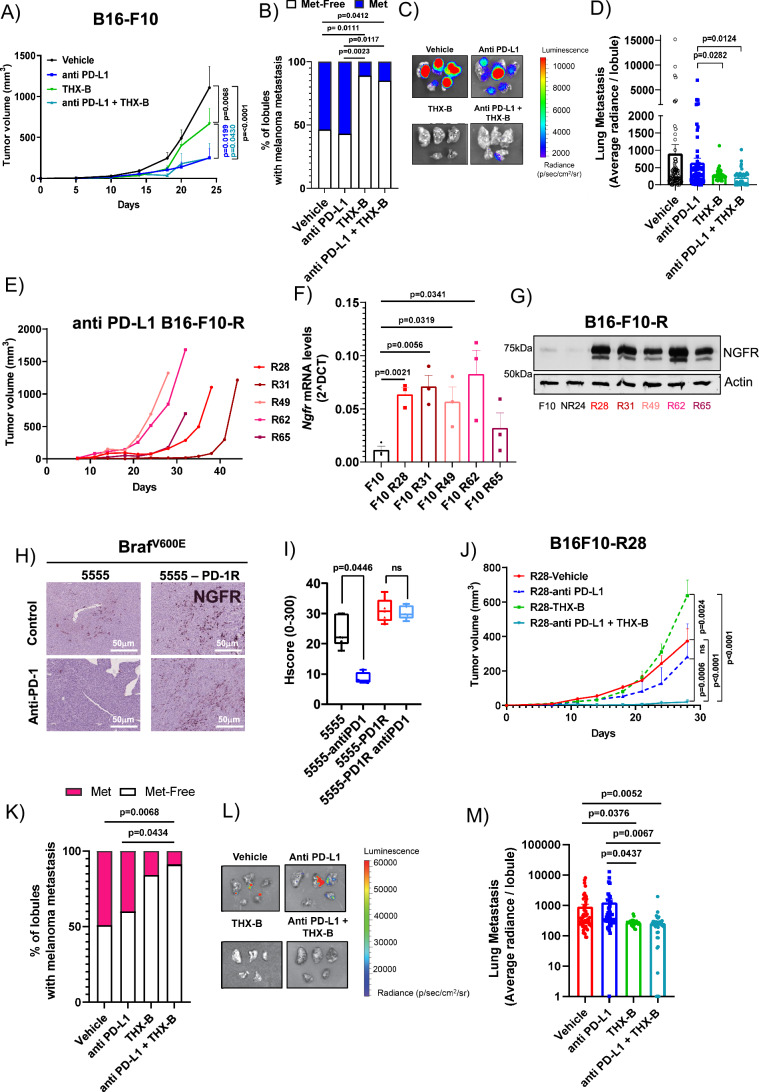
Combination of THX-B and anti-PD-L1 reduces melanoma metastasis and reverts immunotherapy resistance. (**A**) Orthotopically implanted B16-F10-GFPLuc tumors were treated with THX-B (5 mg/kg) and PD-L1(10 mg/kg), alone or in combination i.p., twice a week. Anti IgG2a (10 mg/kg) in PBS 1%DMSO was used as a vehicle. Tumor growth curves are shown. (**B**–**D**) Lung metastasis at the end of the treatment. (**B**) Quantification of the percentage of lobules with melanoma metastases, as in Fig. 1). (**C**) Representative pictures and (**D**) quantification of Luc expression (average radiance/lobule) by IVIS. *n* = 8–16 mice/ group from 2 to 4 independent experiments. (**E**) B16-F10-GFPLuc melanoma cells were injected s.c into B6 mice. 7 days later, i.p. IgG2a or anti-PD-L1 (10 mg/Kg) treatment were administered twice a week over 3–4 weeks. Mice were sacrificed when they reached >1000 mm^3^ or at day 45. Tumor growth is shown. (**F**,** G**) RT-PCR of murine Ngfr (**F**) or western blot of NGFR (**G**) was analyzed in melanoma isolated cells from anti-PD-L1-resistant B16-F10-GFPLuc tumors. A sensitive cell line (NR24, non-resistant) was used as a control in the Western Blot. *n* = 3 independent experiments. (**H**,** I**) 5555 murine melanoma cells (BRAFV600E) or anti-PD-1 resistant derivatives were injected subcutaneously into B6 mice and treated with anti-PD-1(Orgaz et al, 2020). NGFR expression was analyzed by IHC (scale bar = 50 μm) (**H**) and quantified using H-score maps (**I**). *n* = 4–6 mice/group. Box plots show the mean (center), the 25th–75th percentiles (box), and the whiskers denote minimum and maximum values. (**J**) At day 7 after s.c injection of 200,000 anti-PD-L1-resistant B16-F10-R28 melanoma cells in C57/BL6 mice, THX-B (5 mg/Kg), anti-PD-L1 (10 mg/Kg), alone or in combination, were intraperitoneally administered twice a week. Tumor volume was measured using a caliper. (**K**–**M**) Analysis of distal metastasis after treatment. Distal metastasis were analyzed by measuring luciferase expression in the lungs using the IVIS system. (**K**) Percentage of lung lobules with melanoma metastasis. (**L**) Representative IVIS image. (**M**) Quantification of the average radiance/lobule. Error bars represent SEM of *n* = 5–13 mice/group from 1 to 3 independent experiments. Statistical analyses performed were two-way ANOVA with Tukey correction for multiple comparison (**A**, **I**, **J**), unpaired *t*-test (**B**, **D**, **F**, **K**, **M**) and one-way ANOVA with Tukey correction for multiple comparison (**I**). ns not significant. Source data are available online for this figure.

We next tested whether the use of THX-B combined with anti-PD-L1 in melanoma tumor growth and metastasis (See Fig. EV2A scheme). Analysis of THX-B efficacy using the syngeneic murine melanoma model B16-F10 revealed that THX-B treatment reduced both primary tumor growth and metastasis (Fig. 2A–D). Moreover, we observed that combination treatment decreased primary tumor growth similarly to anti-PD-L1 treatment (Fig. 2A). Importantly, despite the strong reduction in tumor growth (Fig. 2A), analysis of the lung metastases showed that anti-PD-L1 treatment alone did not decrease the metastatic burden. (Fig. 2B–D). However, the combination of anti-PD-L1 with THX-B significantly reduced distal melanoma metastases (Fig. 2B–D), suggesting that the combination provides dual control over primary tumor growth and metastasis.

Since previous studies have linked NGFR expression with immunotherapy resistance (Boshuizen et al, 2020; Mehta et al, 2018), we generated cell lines derived from B16-F10 tumors resistant to anti-PD-L1 (Fig. 2E) and evaluated NGFR expression. We found increased NGFR expression in all anti-PD-L1-resistant cell lines at both the mRNA (Fig. 2F) and protein levels (Fig. 2G). Moreover, PD-L1 expression was also increased in anti-PD-L1-resistant tumors (Fig. EV2B,C). We validated this association in additional models of immune resistance, such as murine 5555 tumors resistant to anti-PD-1 therapy (Orgaz et al, 2020) (Fig. 2H,I). Notably, NGFR levels remained elevated and unchanged in PD-1-resistant tumors but were reduced in tumors that responded to therapy (Fig. 2H,I).

We next assessed the efficiency of THX-B treatment in immunotherapy-resistant tumors in vivo. Notably, while anti-PD-L1 monotherapy induced only a partial response, its combination with THX-B inhibited primary tumor growth in anti-PD-L1-resistant tumors (Figs. 2J and EV2D). Importantly, while lung metastasis was not affected by anti-PD-L1 treatment (Figs. 2K–M and EV2E–G, THX-B in monotherapy (Fig. 2K–M) and its combination with anti-PD-L1 reduced both the number and size of lung metastases in two resistant models (Figs. 2K–M and EV2E–G). These results demonstrate that NGFR inhibition reduces metastasis and enhances the efficacy of immunotherapy, highlighting its potential as a therapeutic strategy to overcome treatment resistance and reduce metastatic progression.

**Figure 3:**
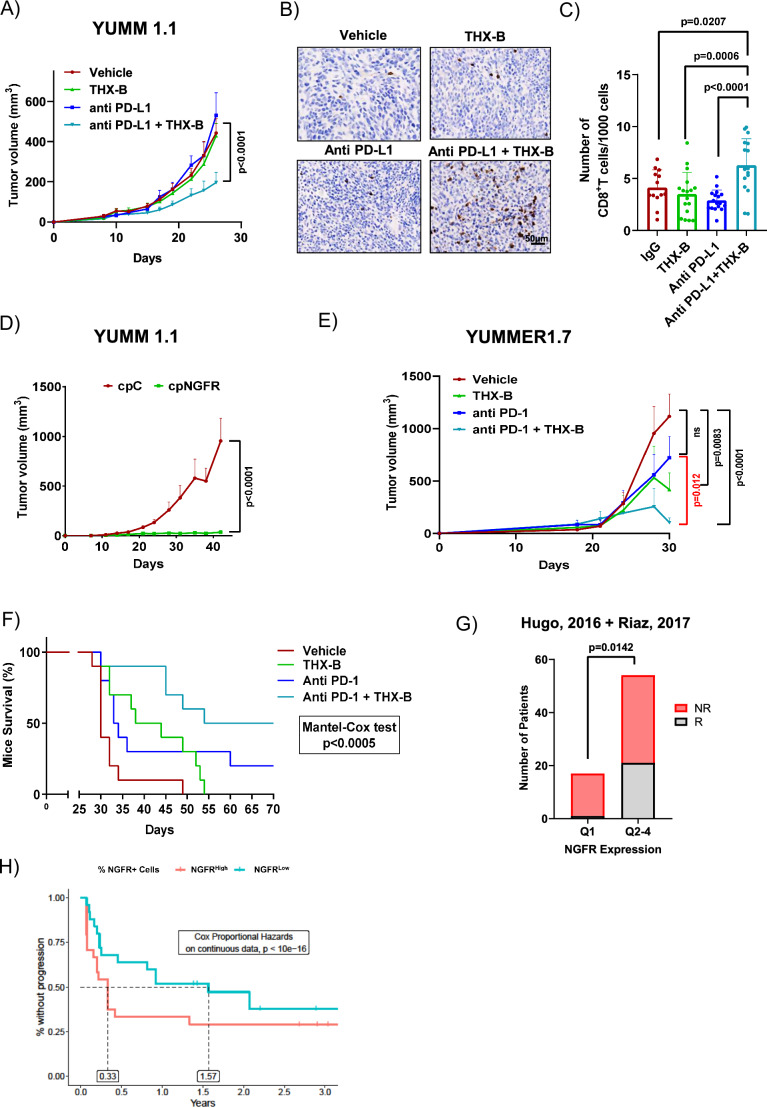
THX-B complements immune checkpoint blockade in preclinical model and NGFR is associated with reduced response to immunotherapy in melanoma patients. (**A**–**C**) 10^6^ YUMM1.1 were subcutaneously injected into B16 mice. After 7 days, anti-PD-L1 (10 mg/Kg) and THX-B (5 mg/Kg), alone or combined, were administrated twice a week intraperitoneally (IP). (**A**) Tumor growth curves are shown. Data were mean + SEM of *n* = 9 mice/group from two independent experiments. Two-way ANOVA with multiple comparisons was performed. (**B**) Tumors from (**A**) were resected and T cell recruitment was analyzed by IHC staining of CD8. A representative image of each group is shown. (**C**) The number of CD8^+^ T cells was calculated using QuPath-0.5.1 software and normalized by the number of cells. Data were mean + SEM of *n* = 13–17 tumor slides from mice of two independent approaches. One-way ANOVA with Tukey correction for multiple comparison was performed. (**D**) 10^6^ cpC or cpNGFR1 YUMM1.1 cells were subcutaneously injected into B16 mice. Tumor growth was measured using a caliper twice a week. Mean + SEM of *n* = 9–10 mice per group from two independent experiments. Two-way ANOVA was performed. (**E**,** F**) YUMMER1.7 tumors growth in syngeneic mice were evaluated using anti-PD-1 mAb, THX-B, or their combination versus controls. Mice were inoculated by subcutaneous injections of 600,000 tumor cells in the left flank. Once tumors were visible (10 days), anti-PD-1 (10 mg/Kg), THX-B (5 mg/Kg) or a combination of anti-PD-1/THX-B, was IP administrated once a week. Data were mean + SEM of *n* = 10 mice, two independent experiments. (**E**) Two-way ANOVA with multiple comparisons was performed. (**F**) Survival Kaplan–Meier plots of mice used in (**E**). Mantel Cox test was performed. *P* < 0.0005. (**G**) NGFR stratification in quartiles (Q1, NGFR highest expression) from datasets GSE91061; (Riaz et al, 2017) and GSE78220 (Hugo et al, 2016) of IT responders or non-responders melanoma patients. *n* = 71 patients. A chi-square Fisher's exact test was used. (**H**) Analysis of NGFR expression inversely correlates with PFS upon immunotherapy treatment. 45 patients of melanoma received immunotherapy (Ipilimumab, Nivolumab, Pembrolizumab, anti-LAG3, or some combination thereof). TTP curves and median progression time for the patients from whom the top and bottom 20% of NGFR^+^ expression is shown. The effect of NGFR on TTP was calculated using the Cox proportional hazard model, *p* < 10^−16^. Source data are available online for this figure.

To determine whether the combination of THX-B and PD-L1 is also effective in models with intrinsic resistance, we tested it in mice with tumors derived from YUMM1.1 cells, which have intrinsic resistance to immunotherapy (Homet Moreno et al, 2016; Meeth et al, 2016) and express high levels of NGFR (Fig. EV2H). While monotherapy with either THX-B or anti-PD-L1 alone did not affect tumor growth (Fig. 3A), their combination significantly reduced primary tumor growth (Fig. 3A). Moreover, tumors from mice treated with the combination therapy showed increased infiltration of CD8^+^ T cells (Fig. 3B,C).

We next generated a YUMM1.1 NGFR knockout (KO) cell line (Fig. EV2I) to evaluate the impact of NGFR deletion on tumor growth. Interestingly, the knockout of NGFR completely abrogated primary tumor growth (Fig. 3D). Additionally, we evaluated the impact of NGFR knockout on CD8^+^ T cell infiltration using this model. To rule out differences due to tumor size, we implanted cells into immunocompetent mice and analyzed tumors of comparable volume (100–200 mm³; Fig. EV2J). This analysis revealed that tumors derived from YUMM1.1 NGFR KO cells exhibited increased infiltration of CD8^+^ T cells compared to control tumors (Fig. EV2K,L). We also evaluated whether the combined NGFR inhibition and immune checkpoint blockade would affect survival using the YUMMER1-7 tumor model, which is partially resistant to anti-PD-1 therapy (Fig. 3E). While THX-B alone reduced tumor growth, its combination with anti-PD-1 significantly impaired tumor growth (Fig. 3E) and importantly, enhanced mouse survival (Fig. 3F). To assess the clinical relevance of NGFR expression in melanoma patients, we analyzed NGFR expression in two independent cohorts of patients treated with immune checkpoint therapy (Data ref. (Hugo et al, 2016; Riaz et al, 2017)). Across both datasets, NGFR expression showed a trend towards higher levels in non-responders (Fig. EV2M). Stratifying patients by NGFR expression revealed that those in the highest-expression quartile (Q1) were predominantly non-responders (Fig. 3G), suggesting that high NGFR levels may help identify patients refractory to immune checkpoint therapy.

Based on these findings, we next analyzed NGFR expression by immunohistochemistry in tumors from a cohort of 45 melanoma patients undergoing immunotherapy treatment (Table EV1). We observed that patients with high NGFR expression (Fig. EV2N) exhibited significantly reduced progression-free survival (PFS) following immunotherapy (Fig. 3H) and experienced disease recurrence within the first 4 months after the initial immunotherapy infusion, resulting in approximately fivefold shorter survival (Fig. 3H). Altogether, these results demonstrate that THX-B enhances the efficacy of immune checkpoint blockade. Moreover, our results support the use of NGFR as a novel PFS biomarker in melanoma patients undergoing immunotherapy.

### NGFR-MLC2 axis promotes melanoma cell invasion at the invasive front

**Figure 4:**
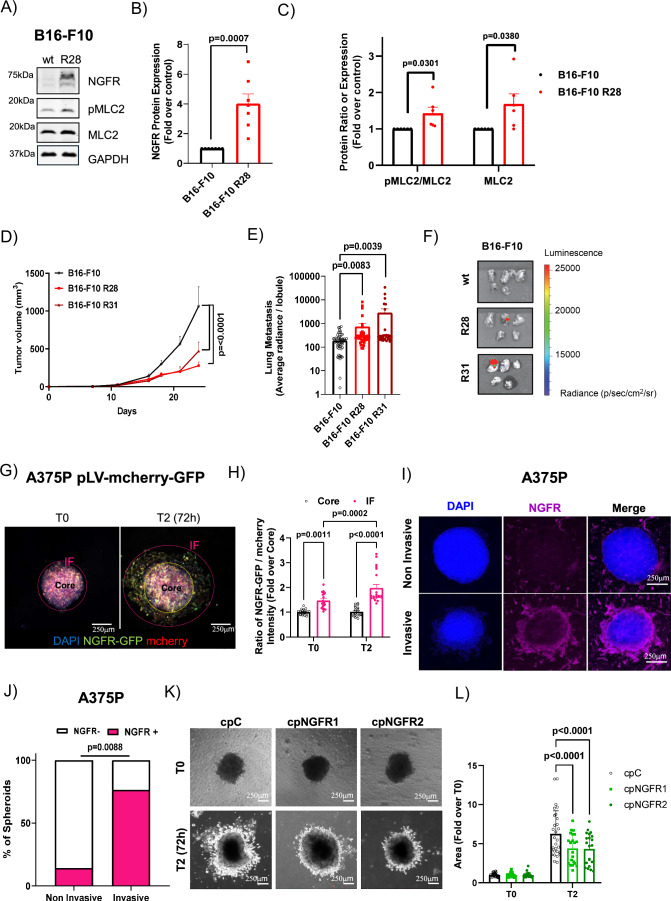
NGFR is expressed at the invasive front of invasive spheroids and is required for melanoma cell migration and invasion. (**A**) Anti-PD-L1-resistant cells and tumors harbor invasive phenotypes. Representative western blot of B16-F10 wildtype and R28 cells with the indicated markers. (**B**,** C**) Quantification of NGFR (**B**), pMLC2/MLC2 ratio and MLC2 expression (**C**) measured by western blot and plotted as fold over the control group. *n* = 7 (**B**) and 6 (**C**) independent experiments. Data were mean + SEM, unpaired *t-*test was used for statistical analysis. (**D**) 300,000 B16-F10 GFPLuc wildtype or anti-PD-L1-resistant R28 and R31 cells were injected in the flank of B6 mice. Tumor growth was measured twice a week. Data were mean + SEM of *n* = 14–21 mice/group from 2 (R31) or 3 (F10wt and R28) independent experiments. Two-way ANOVA statistical analysis with Tukey correction was used. (**E**,** F**) Mice were sacrificed when tumors from the control group reached 1000 mm^3^ and lungs were analyzed ex vivo at endpoint by measuring Luc expression at the IVIS system. *n* = 6–12 mice/group from 1–3 independent experiments. (**E**) Melanoma metastases intensity measured by luciferase average radiance expression per lobule of each mouse. Data were mean + SEM. Unpaired *t*-test was performed. Figure **E**, **F** includes a redisplay of some of the data originally presented in Fig. 2A,L (vehicle-treated groups), as required for comparative purposes. (**F**) Representative IVIS images. (**G**) Multicellular spheroids of A375P pLV-mcherry-GFP (3 × 10^3^cells) were prepared in hanging droplets and embedded in 3D type I collagen gels (2.2 mg/ml, prepared from acid extracts of rat tail). After 3 days, samples were fixed and stained with DAPI. Invasion was monitored by confocal microscopy at 0-day (T0) and after 72 h of culture (T2). (**H**) NGFR expression was evaluated by measuring the ratio between the intensity of the GFP^+^ (cells with active NGFR promoter) and mCherry expression (expressed by all cells) at both the distal site (IF) or at the core of the spheroid. Data were mean + SEM of each spheroid measured (*n* = 20–21 from two independent experiments). Two-way ANOVA with Tukey correction was applied. (**I**) 3D spheroids from A375P cells grown after 3 days into 3D type I collagen were fixed in 4% PFA and stained with NGFR by immunofluorescence. A representative confocal plane from the Z-stack is shown. (**J**) The number of NGFR^+^ spheroids were analyzed using a chi-square Fisher exact test. (*n* = 7 non-invasive spheroids and 17 invasive spheroids from two independent experiments). (**K**) cpC, cpNGFR1 or cpNGFR2 A375P cells were grown in spheroids and placed into a 3D collagen I matrix as (**I**, **J**). Bright-field images were captured with a bright-field microscope. Representative images are shown. (**L**) Invasion area in 3D type I collagen gels was calculated by estimating the area of spheroids at T0 and T2 using ImageJ software. For each cell population, the mean invasion area at T2 was normalized to the mean invasion area at T0 and plotted with error bars representing +SEM (*n* = 21–39 spheroids for each cell population from two independent experiments). Two-way ANOVA with Tukey correction for multiple comparison was applied. Source data are available online for this figure.

**Figure EV3:**
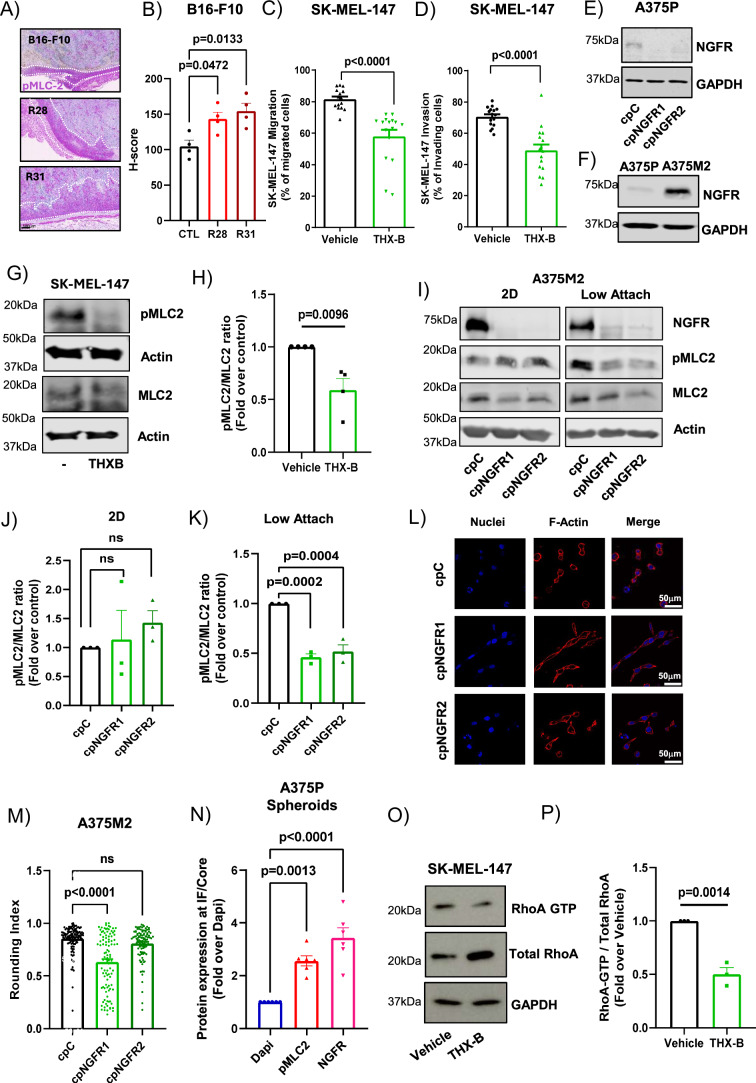
NGFR is required for the ROCK-MLC2 ameboid metastasis of human melanoma tumors. (**A**) Representative IHC of pMLC2 activation at the invasive front of parental (CTL) or anti-PD-L1-resistant (R28 and R31) B16-F10 tumors. The invasive front (IF) was indicated by white dots. (**B**) Qu-Path quantification of pMLC2 expression at the IF (defined by white dots). One-way ANOVA with Tukey multi-comparison correction was performed. Representative data (Mean + SEM) from 1 experiment *n* = 4 mice replicated in 2 independent experiments. (**C**,** D**) Transwell SK-MEL-147 cell migration (**C**) and invasion (**D**) to FBS (% of total cells) after treatment with vehicle (DMSO 1.6%) or THX-B (20 μM, ON). Data were mean + SEM of four fields per condition of two independent experiments performed in duplicate. Student’s *t*-test. (**E**) Representative Western blot of NGFR knockdown in A375P cells. (**F**) Representative Western blot showing increased NGFR expression levels in ameboid A375M2 cells compared to parental A375P cells. (**G**) Representative Western blot showing reduced pMLC2 levels in SK-MEL-147 cells upon THX-B treatment (20 μM, 24 h). (**H**) Quantification of the pMLC2/total MLC2 ratio and referred to SK-MEL-147 treated with vehicle. Data were mean + SEM of *n* = 4 independent experiments. Unpaired *t*-test was performed. (**I**) Representative Western blot of the effective CRISPR/Cas9 knockdown of NGFR in A375M2 cells and the activation of pMLC2 in 2D standard adhesion or low adhesion conditions. (**J**,** K**) Quantification of the pMLC2/total MLC2 ratio in A375M2 cpControl vs NGFR KO cells in 2D conditions (**J**) or low-attachment plates (**K**) Data were mean + SEM of *n* = 3 independent experiments. One-way ANOVA with Dunnett multi-comparison test was performed. ns not significant. (**L**) Confocal images of cpC, cpNGFR1 and cpNGFR2 A375M2 cells seeded on top of collagen for 24 h. (**M**) Quantification of rounded versus elongated cells. Data represent mean + SEM of 126, 102, and 108 cells from two independent experiments. One-way ANOVA with Dunnett correction was performed. ns, not significant. (**N**) Quantification of DAPI, NGFR, and pMLC2 protein expression at the invasive front of A375P-3D invasive spheroids upon 3 days of culture into collagen I matrix. Mean intensity was calculated as the ratio between the expression at the IF vs Core of the spheroid and represented as fold over the DAPI condition +SEM. *n* = 6 spheroids/group. One-way ANOVA analysis with Tukey multi-comparison was performed. (**O**,** P**) Pull down of RhoA-GTP in SK-MEL-147 cells treated with THX-B (20 μM, 24 h). (**O**) Representative Western blot. (**P**) RhoA-GTP relative activation to total RhoA expression is represented as fold over the untreated condition. *n* = 3 independent experiments were performed and represented as mean + SEM. An unpaired *t-*test (**P**) statistics test was applied. Source data are available online for this figure.

Previous studies have demonstrated that therapy resistance in melanoma is associated with ameboid invasive behavior mediated by the activation of MLC2 (Orgaz et al, 2020). Interestingly, NGFR is highly expressed by ameboid melanoma cells, although the molecular mechanisms remain unclear (Georgouli et al, 2019). To investigate the potential relationship between NGFR and MLC2 in melanoma, we first analyzed NGFR, total MLC2, and pMLC2 in anti-PD-L1-resistant B16-F10 cells. We observed that PD-L1-resistant cells have increased levels of NGFR, MLC2, and pMLC2 (Fig. 4A–C). In addition, analysis in vivo showed that PD-L1- resistant tumors were smaller in size (Fig. 4D) but exhibited increased metastatic behavior (Fig. 4E,F). Moreover, the number of pMLC2-positive cells was increased at the invasive front (IF) of resistant tumors (Fig. EV3A,B).

In order to understand the relevance of NGFR regulating MLC2 activity and the consequences in the acquisition of an invasive phenotype, we analyzed the effect of NGFR inhibition on migration and invasion. Treatment with THX-B reduced both cell migration (Fig. EV3C) and invasion (Fig. EV3D) of SK-MEL-147 cells, characterized by high levels of NGFR expression (Garcia-Silva et al, 2021). To further explore the role of NGFR in melanoma invasive behavior, we analyzed NGFR expression at the IF of A375P spheroids, a model system that is useful for studying the ameboid invasion of melanoma cells in vitro (Georgouli et al, 2019; Kozlowski et al, 1984; Rodriguez-Hernandez et al, 2020; Xu et al, 2008). To monitor NGFR expression, we generated a reporter model where GFP is expressed under the control of the human *NGFR* promoter while mCherry is driven by a constitutive promoter. This model allowed us to track NGFR expression (GFP^+^) in melanoma cells (mCherry^+^). Using confocal imaging, we analyzed NGFR expression at the IF of tumor cell spheroids embedded in collagen I over time. NGFR expression was significantly enriched at the IF of the spheroids at the beginning of the experiment (Fig. 4G,H, T0). Furthermore, after 72 h, NGFR expression was further increased at the IF (Fig. 4G,H), supporting an important role for NGFR at IF.

To gain further insights into NGFR expression at the IF, we analyzed its expression by immunofluorescence in A375P tumor cell spheroids embedded in collagen I for 72 h (Fig. 4I,J). We observed that NGFR expression was enriched at the IF in ~80% of spheroids able to invade the collagen (Fig. 4I,J), while its expression was absent in almost all the non-invasive spheroids (Fig. 4I,J). Next, we analyzed the effect of NGFR KO on the invasive behavior of A375P spheroids. We generated CRISPR NGFR KO cell lines (Fig. EV3E) and assessed the invasive properties of the spheroids in collagen I matrix. We observed that NGFR KO reduced the invasive capacity of the spheroids (Fig. 4K,L). Overall, our findings demonstrate that NGFR is crucial for driving the invasive phenotype in melanoma cells, being enriched at the IF.

### NGFR drives melanoma invasive phenotype through modulation of MLC2 and RhoA activity

**Figure 5:**
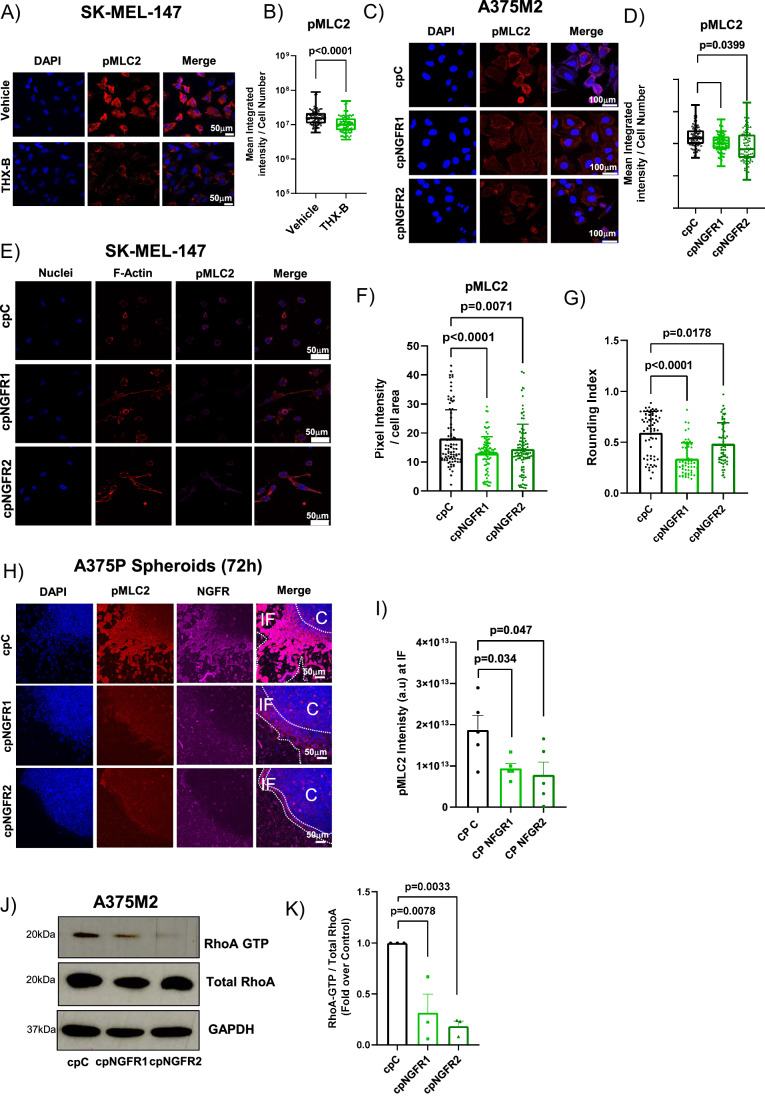
NGFR regulates ameboid cell invasion through myosin activation. (**A**) Confocal images of pMLC2 in SK-MEL-147 cells treated with THX-B (20 μM, 24 h). (**B**) Integrated intensity of pMLC2 of the FOV/cell number of the FOV represented in arbitrary units of fluorescence. Unpaired *t*-test was performed. Data were mean + SEM of 30 fields captured from three independent experiments. (**C**) Confocal images of pMLC2 (red) and DAPI (blue) in cpC, cpNGFR1 and cpNGFR2 A375M2 cells seeded on collagen I surfaces. (**D**) Graph the integrated intensity of pMLC2 of the FOV / cell number of the FOV represented in arbitrary units of fluorescence. Data represent mean + SEM of 27–30 fields captured from three independent experiments. One-way ANOVA with Tukey correction for multiple comparison was performed. (**E**) Confocal images of cpC, cpNGFR1, and cpNGFR2 SK-MEL-147 cells seeded on top of collagen for 24 h. (**F**,** G**) Quantification of S19 light phospho-myosin 2 intensity (pixel intensity in single cells relative to the cell area **F**) and roundness ImageJ quantification of (0–1) using F-actin staining (**G**). Data represent mean + SEM of 59–101 cells captured from three independent experiments. One-way ANOVA with Tukey correction was applied. (**H**) NGFR, pMLC2, and DAPI Immunofluorescence staining were captured in z-stacks of 40X confocal images of 3D invasive spheroids of cpC, cpNGFR1, and cpNGFR2 A375P spheroids after 3 days of culture into collagen I matrix. Representative images are shown. Invasive front (IF) and core (C) of the spheroids are delimited by white dots. Scale bars (50 μm) are indicated in the merged images. (**I**) Activation of pMLC2 at the IF of 3D invasive spheroids as in (**G**). Arbitrary units of mean intensity of pMLC2 relative to the mean intensity of DAPI are shown. *n* = 5 spheroids/group. Unpaired *t-*test was performed. *p < 0.05. (**J**) Representative immunoblots of RhoA-GTP in pulldown samples and total RhoA and GAPDH in total lysate of cpC, cpNGFR1 and cpNGFR2 A375M2 cells. (**K**) RhoA-GTP relative activation to total RhoA expression represented as fold over A375M2 cpControl (cpC) condition. *n* = 3 independent experiments. Mean + SEM is represented. One-way ANOVA with Dunnett post-statistic tests were used. Source data are available online for this figure.

Ameboid migration is characterized by rounded cell morphology and elevated actomyosin contractility driven by Rho GTPase and MLC2 signaling (de Winde et al, 2021). Interestingly, NGFR expression is increased in ameboid A375M2 cells, associated with more aggressiveness and therapy resistance in melanoma (Georgouli et al, 2019; Orgaz et al, 2020) (Fig. EV3F). To explore the molecular mechanisms involved in tumor cell migration, we analyzed the effect of NGFR inhibition and KO on MLC2 activation in melanoma cell lines. NGFR inhibition by THX-B decreased MLC2 activation in SK-MEL-147 cells by immunofluorescence (Fig. 5A,B) and western blot (Fig. EV3G,H). Similarly, NGFR KO (also reduced MLC2 activation on collagen surfaces in the A375M2 cells (Fig. 5C,D) and SK-MEL-147 (Fig. 5E,F). Notably, NGFR knockout in A375M2 cells reduced MLC2 activation under low-attachment conditions analyzed by western blot, whereas this effect was not observed under standard 2D adhesion on plastic (Fig. EV3I–K). Given that adhesion‑independent migration is a hallmark of ROCK/MLC2‑driven ameboid invasion (Paul et al, 2017), these data support a role for NGFR in regulating ameboid behavior through modulation of MLC2 phosphorylation.

We next analyzed the effect of NGFR KO on the regulation of the ameboid phenotype in human melanoma spheroids on collagen. We observed that NGFR KO in both SK-MEL-147 and A375M2 cell lines reduced the rounding index on collagen surfaces (Figs. 5E–G and EV3L,M). Furthermore, NGFR expression and pMLC2 levels increased at the invasive front of spheroids (Figs. 5H upper panels and EV3N). Importantly, NGFR KO in spheroids reduced pMLC2 at IF (Fig. 5H,I), suggesting that NGFR is crucial in the regulation of MLC2-dependent invasion at IF in melanoma cells.

Since ameboid melanoma cells rely on RhoA for ROCK1/2-dependent MLC2 activation (Sanz-Moreno et al, 2008), and NGFR has been involved in RhoA activation depending on the physiological context (Johnston et al, 2007; Yamashita et al, 2002; Yamashita et al, 1999), we tested RhoA activity in NGFR KO cell lines using pull-down assays to measure guanosine triphosphate (GTP)–bound RhoA. We found that NGFR KO in the A375M2 cell line reduced RhoA activity (Fig. 5J,K). Similarly, RhoA activation decreased in SK-MEL-147 cells treated with THX-B (Fig. EV3O,P). These findings highlight that NGFR regulates melanoma invasion by activating the RhoA-MLC2 signaling pathway, supporting NGFR inhibition as a promising strategy to decrease invasive behavior

### The NGFR–ROCK axis sustains MLC2 stability and promotes invasion in melanoma cells

**Figure 6:**
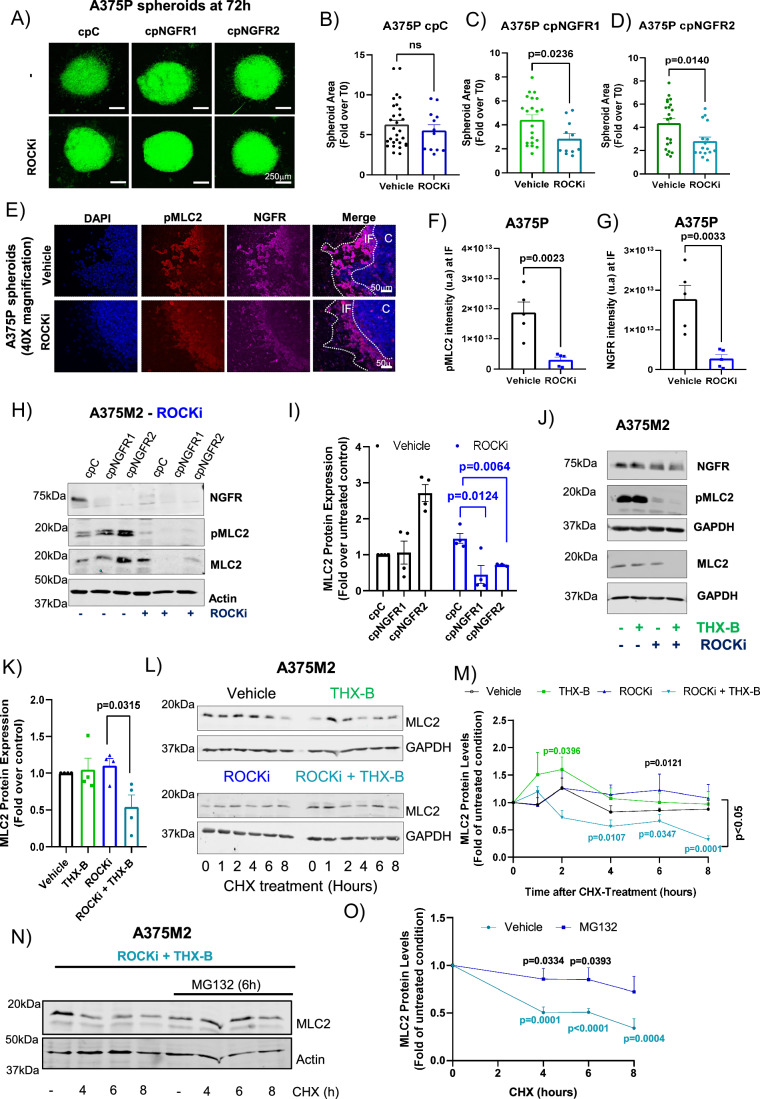
Combined inhibition of NGFR and ROCK1/2 decreased MLC2 stability. (**A**) Multicellular spheroids of cpC, cpNGFR1 and cpNGFR2 A375P-GFP cells (3 × 10^3^) in collagen I matrix upon vehicle or ROCKi (1 μM). Representative confocal images of GFP-spheroids at T2 (72 h) are shown. (**B**–**D**) Invasion area at T2 was normalized to the mean invasion area at T0 and plotted with error bars comparing vehicle vs ROCKi treatment in each condition. Data were mean ± SEM (*n* = 11–29 spheroids/group from two independent experiments). An unpaired *t*-test was performed. (**E**) Representative images of 40X confocal magnification of DAPI, pMLC2, and NGFR expression at the invasive front (IF) of 3D collagen I invasive spheroids formed by A375P cells in the presence of vehicle or ROCKi (1 μM). Core (C). (**F**,** G**) pMLC2 (**F**) and NGFR (**G**) protein expression at the invasive front of 3D spheroids after 3 days of culture in 3D collagen I matrix. Quantification was performed using FIJI software. The mean intensity for NFGR and pMLC2 was normalized by DAPI mean intensity and represented as arbitrary units of fluorescence relative to DAPI expression. Mean + SEM of *n* = 5 spheroids analyzed/group are represented. Unpaired *t*-test was performed. (**H**) Representative western blot of NGFR, pMLC2, MLC2, and GAPDH of cpC, cpNGFR1, and cpNGFR2 A375M2 cells treated or not with ROCKi (1 μM) 24 h. (**I**) MLC2 protein expression was quantified and represented as fold over untreated cpC cells. Data were mean + SEM of *n* = 3–4 Independent experiments. Student's *t*-test was used between different conditions. (**J**) Representative immunoblots of NGFR, pMLC2, MLC2, and GAPDH in the total lysate of A375M2 cells treated with vehicle, THX-B (20 μM), ROCKi (1 μM), and the combination of THX-B and ROCKi for 24 h. (**K**) Total MLC2 expression was calculated using Image Studio Lite Ver 5.2 (LI-COR), corrected by GAPDH expression levels and represented as fold change over the vehicle-treated condition. Mean + SEM of *n* = 4 independent experiments. One-way ANOVA plus Tukey multiple comparison was performed. (**L**) Representative immunoblots of A375M2 cells treated with vehicle, THX-B (20 μM), ROCKi (1 μM), and the combination of THX-B and ROCKi for 24 h. Cycloheximide (20 µg/ml) was added to cells for the indicated periods before cell lysis. Total MLC2 and GAPDH levels were shown. (**M**) MLC2 protein levels were normalized with GAPDH expression and depicted as fold of expression over 0 h of cycloheximide treatment. Data (mean ± SEM.) from four independent experiments are shown. An unpaired *t*-test was performed comparing each time point vs time 0 of cycloheximide treatment or comparing the combination treatment (ROCKi + THX-B) with the other treatments at the end point. *p* < 0.05 means *p* = 0.0204 (Combo vs vehicle), *p* = 0.0325 (Combo vs THX-B) and *p* = 0.0258 (Combo vs ROCKi). (**N**) Representative immunoblots of A375M2 cells treated with the combination of THX-B and ROCKi for 24 h and cycloheximide (20 µg/ml) for the indicated times. MG132 (10 μM) was added for 6 h to half of the wells to block proteasome-dependent degradation. Total MLC2 and GAPDH levels were shown. (**O**) MLC2 protein levels were normalized with GAPDH expression and depicted as fold of expression over 0 h of cycloheximide treatment. Data (mean  + SEM.) from four independent experiments are shown. Unpaired *t*-test was performed comparing the protein decay at each time point vs T0 of cycloheximide treatment (*p* in blue) and comparing protein levels at each time point of MG132-treated vs untreated A375M2 cells (*p* shown in black). Source data are available online for this figure.

**Figure EV4:**
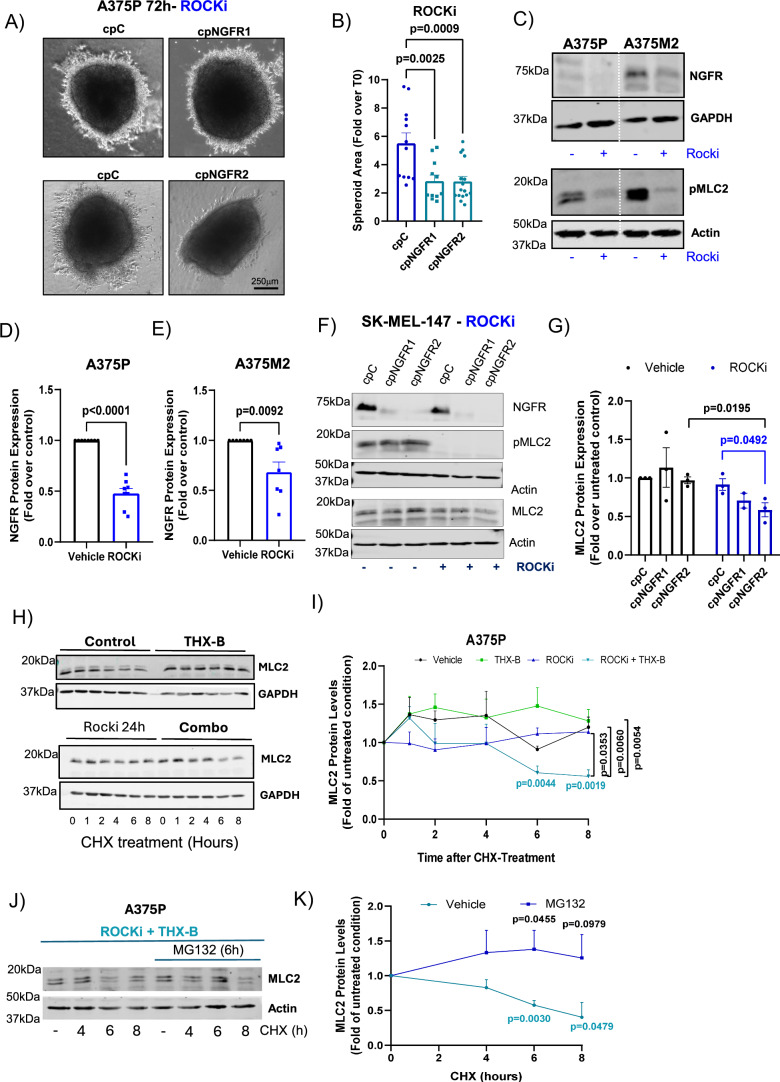
Depletion of NGFR in combination with ROCKi compromised MLC2 stability. (**A**) Multicellular spheroids of cpC, cpNGFR1 and cpNGFR2 A375P (3 × 10^3^cells) were prepared as in Fig. 4J–L in the presence of ROCKi (1 μM). Representative bright-field images of T2 (72 h) invasive spheroids upon ROCKi are shown. (**B**) Invasion area at T2 was normalized to mean invasion area at T0 and plotted with error bars representing ±SEM (*n* = 11–16 spheroids/group from two independent experiments). One-way ANOVA with Dunnett multiple comparison was performed. (**C**) Representative western blot of NGFR / GAPDH, pMLC2/Actin of A375P and A375M2 cells treated with ROCKi (1 μM) 24 h. (**D**,** E**) NGFR protein expression represented as fold over vehicle-treated condition in A375P (**D**) and A375M2 (**E**) cells. Data were mean + SEM of *n* = 7 and 8 independent experiments (A375M2 and A375P cells, respectively). Student's *t*-test was used as a statistical method. (**F**) Representative Western blot of NGFR, MLC2, and GAPDH of cpC, cpNGFR1, and cpNGFR2 SK-MEL-147 cells treated or not with ROCKi (1 μM) 24 h. (**G**) MLC2 protein expression was quantified and represented as fold over untreated cpC cells. Data were mean + SEM of 2–3 independent experiments. Student's *t*-test was used. (**H**) Representative immunoblots of A375P cells treated with vehicle, THX-B (20 μM), ROCKi (1 μM), and the combination of THX-B and ROCKi for 24 h. Cycloheximide (20 µg/ml) was added to cells for the indicated periods before cell lysis. Total MLC2 and GAPDH levels were shown. (**I**) MLC2 protein levels were normalized with GAPDH expression and depicted as fold of expression over 0 h of cycloheximide treatment. Data (mean + SEM.) from four independent experiments are shown. Unpaired *t*-test was performed comparing MLC2 expression at each time point vs time 0 of cycloheximide treatment. (**J**) Representative immunoblots of A375P cells treated with the combination of THX-B and ROCKi for 24 h and cycloheximide (20 µg/ml) for the indicated times. MG132 (10 μM) was added for 6 h to half of the wells to block proteasome-dependent degradation. Total MLC2 and GAPDH levels were shown. (**K**) MLC2 protein levels were normalized with GAPDH expression and depicted as a fold of expression over 0 h of cycloheximide treatment in the control group. Data (mean  + SEM.) from four independent experiments are shown. Unpaired *t-*test was performed comparing the protein decay at each time point vs T0 of cycloheximide treatment (*p* in blue) and comparing protein levels at each point of MG132-treated vs untreated A375P cells (*p* in black). Source data are available online for this figure.

ROCK1/2 regulates ameboid cell migration through the activation of MLC2, and it is implicated in therapy resistance in melanoma cells (Totsukawa et al, 2000). Importantly, the use of ROCK inhibitors (ROCKi) has been used to revert therapy resistance with effectiveness (Orgaz et al, 2020). Thus, we decided to explore the effect of the combination of both ROCKi and THX-B. We first compared the effect of ROCKi treatment on the invasive behavior of NGFR-expressing versus NGFR-KO cells. While ROCK inhibition reduced the invasive capacity of A375P spheroids, this effect was not statistically significant (Fig. 6A,B). In contrast, ROCKi treatment significantly decreased the invasion of NGFR-KO A375P cells into collagen matrices (Figs. 6A,C,D and EV4A,B), suggesting functional cooperation between NGFR and ROCK signaling pathways in promoting melanoma invasion. Interestingly, we observed that ROCKi treatment reduced both NGFR and pMLC2 expression at the IF of the A375P spheroids (Fig. 6E–G). Interestingly, we found that ROCK inhibition led to a decrease in NGFR expression in both A375P and A375M2 cell lines (Fig. EV4C–E), suggesting a potential auto-regulatory loop between NGFR and ROCK.

We next analyzed the combined effect of ROCK and NGFR inhibition in metastatic melanoma cells. As expected, we observed that ROCKi impaired MLC2 phosphorylation (Fig. 6H). Notably, ROCKi treatment in NGFR KO A375M2 and NGFR KO SK-MEL-147 cells led to a decrease in total MLC2 expression (Figs. 6H,I and EV4F,G). Similarly, only the combined inhibition of NGFR and ROCK reduced total MLC2 expression (Fig. 6J,K), suggesting that the coordinated activity of both pathways is critical for maintaining MLC2 protein levels.

Since ROCK inhibition affected total MLC2 levels only when NGFR expression or activity was suppressed, we hypothesized that the ROCK–NGFR axis may regulate MLC2 protein stability. To test this, we treated A375M2 and A375P cells with THX-B, ROCKi, or a combination of both. After 24 h, cycloheximide was added to block de novo protein synthesis, allowing us to assess MLC2 protein stability under each condition. MLC2 expression levels were then analyzed at 2-, 4-, 6-, and 8-h post-treatment.

MLC2 levels remained invariable in control cells and in those treated with either THX-B or ROCKi alone, indicating that MLC2 is relatively stable in A375M2 and A375P melanoma cells (Figs. 6L,M and EV4H,I, green and blue lines). In contrast, combined treatment with THX-B and ROCKi significantly reduced MLC2 half-life to approximately 4 h in A375M2 cells, with continued decline at 6 and 8 h post-treatment (Figs. 6L,M and EV4H,I, light blue line). Notably, treatment with the proteasome inhibitor MG132 rescued MLC2 protein levels in both cell lines (Figs. 6N,O and EV4J,K), supporting the idea that NGFR and ROCK cooperatively maintain MLC2 stability by preventing its proteasomal degradation.

### NGFR/MLC2 axis is enriched at the invasive front of human metastatic melanomas and in melanoma cells derived from immunotherapy-resistant patients

**Figure 7:**
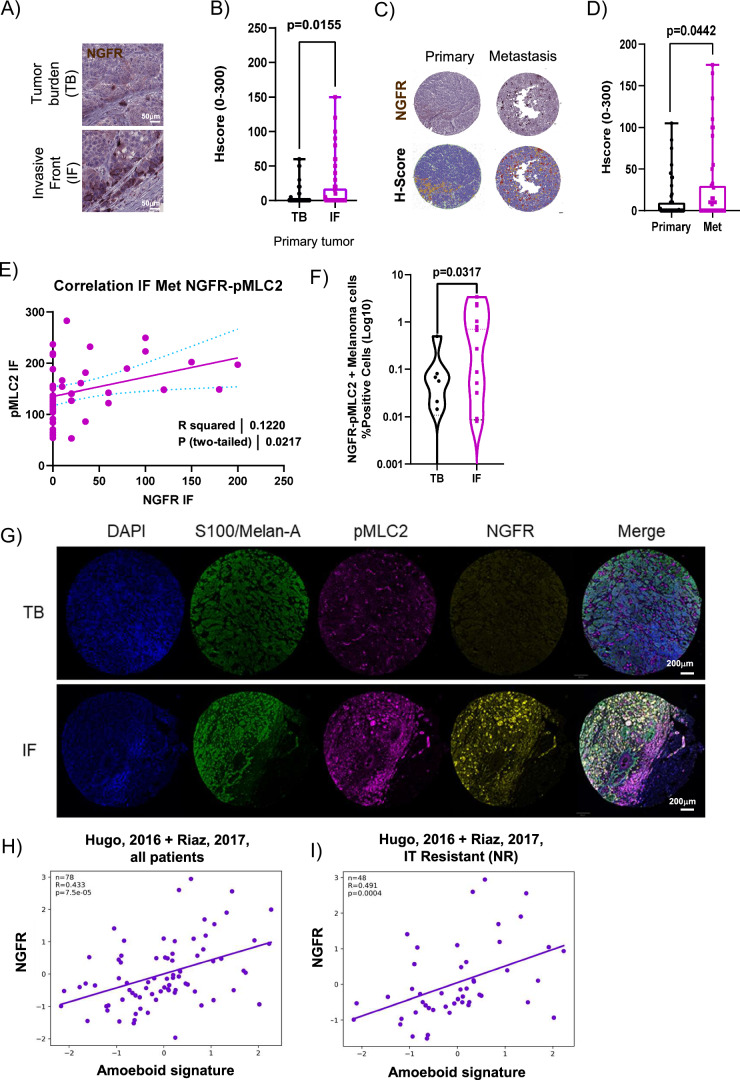
NGFR/MLC2 as a new axis indicative of melanoma metastasis and immunotherapy relapse. (**A**) Representative IHC NGFR staining in tumor body (TB) or invasive front (IF) from human primary melanomas. Scale bar (50 μm). (**B**) H-score of NGFR staining in the tumor body (TB) or invasive front (IF) of matched samples from human primary melanoma. Values range from 0 (no NGFR expression) to 300 (all cells with very intense NGFR staining). *n* = 53 per condition. Box plots show the mean (center), the 25th–75th percentiles (box), and the whiskers denote minimum and maximum values. Mixed effect with Tukey multi-comparison test was performed. (**C**,** D**) Representative image (**C**) and quantification (**D**) of NGFR expression in 53 primary vs 43 metastatic melanomas. Box plots show the mean (center), the 25th–75th percentiles (box), and the whiskers denote minimum and maximum values. Unpaired *t*-test was performed. (**E**) Correlation between NGFR and pMLC2 expression at the Invasive front of metastatic samples. *n* = 43. The Pearson correlation *p* (two-tailed) test was applied and indicated in the figure (*p* = 0.0217). (**F**) % of NGFR^+^ /pMLC2^+^ cells (represented as log10) at the invasive front (IF *n* = 22) vs at the core (TB, *n* = 24) of primary tumors from two different TMAs. Student *t-*test with Welch correction was applied. (**G**) Representative image of the multiplex IHC analysis of **G**). S100/MelanA was used to differentiate melanoma cells from other stromal cells. Scale bar (200 μm). (**H**,** I**) Correlation analyses of NGFR expression and the ameboid signature across two melanoma cohorts (Data ref (Hugo et al, 2016; Riaz et al, 2017)), including the global cohort (**H**, *n* = 78) or IT non-responders (Resistant) cohort (**I**, *n* = 48). Associations between NGFR and the ameboid score were quantified using Spearman’s rank correlation (*R*) with corresponding *p* values. Results were visualized using scatter plots with a fitted linear trend line and annotation of *n*, *R*, and *p* value. Source data are available online for this figure.

**Figure EV5:**
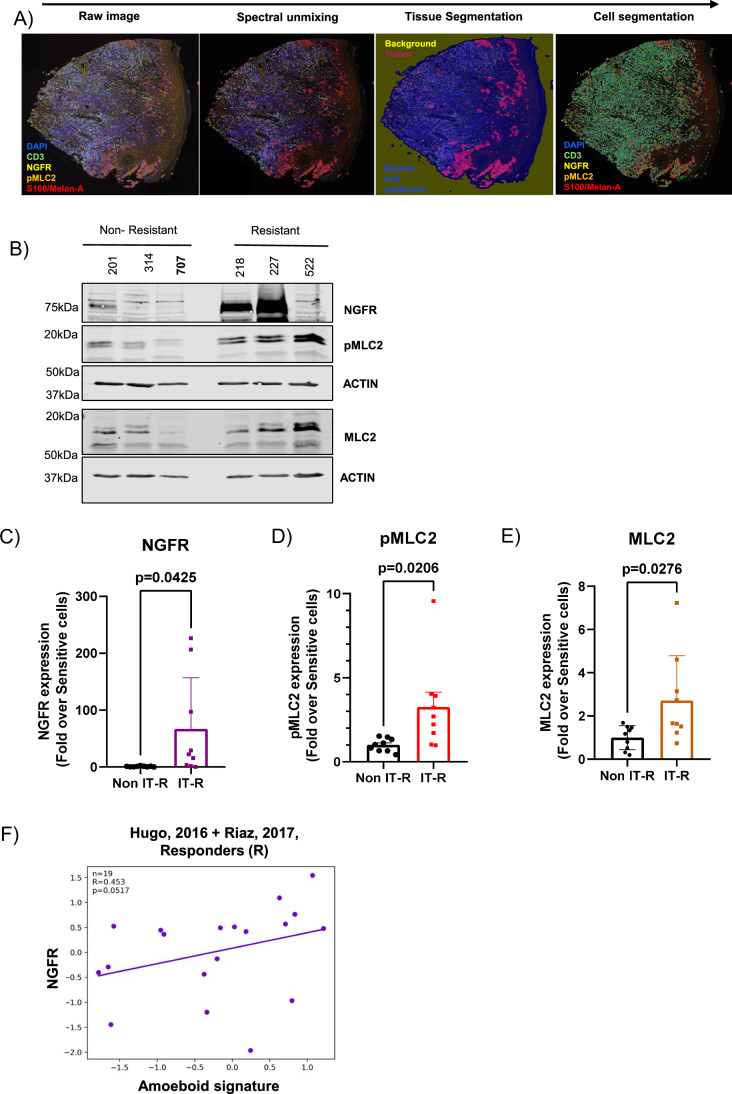
NGFR and pMLC2 are enriched in patient-derived immunotherapy-resistant cell lines. (**A**) Representative images of a core of TMAs from primary melanomas illustrating the analysis pipeline performed with the *inForm®* software. Different staining and tissue sections are indicated in colors. (**B**) Representative immunoblots of NGFR, pMLC2, MLC2, and Actin of non-immunotherapy resistant vs immunotherapy-resistant cell lines derived from metastatic samples from melanoma patients. (**C**–**E**) Quantification of the NGFR expression (**B**), pMLC2/MLC2 ratio (**C**), and MLC2 total levels (**D**) in cells indicated in (**A**). Data were mean + SEM of cell lysates grouped as non-IT-resistant and IT-resistant cells of three different lines in three independent experiments. Student's *t*-test was performed. (**F**) Associations between NGFR and the ameboid signature in responder patients (*n* = 19) were quantified using Spearman’s rank correlation (*R*) with corresponding *p* values. Results were visualized using scatter plots with a fitted linear trend line and annotation of *n*, *R*, and *p* value. Source data are available online for this figure.

To assess the relevance of NGFR expression at the invasive front in human tumors, we performed immunohistochemistry on a cohort of 53 primary and 45 metastasis melanoma patients (Rodriguez-Hernandez et al, 2020) analyzing NGFR expression at the IF and the tumor burden (TB) areas. Notably, we observed a significant increase in NGFR expression at the IF compared to the TB area (Fig. 7A,B). Additionally, analysis of NGFR expression in primary tumors and metastasis demonstrated that it was enriched in metastatic samples compared to matched primary tumors from the same melanoma patients (Fig. 7C,D). We also observed a significant correlation of NGFR and pMLC2 expression at the invasive front of metastatic lesions derived from melanoma patients (Fig. 7E). Analysis of a second cohort of melanoma patients, composed of TMAs from 46 melanoma patients (Table EV2), demonstrated an enrichment of NGFR and pMLC2 at the IF compared to TB (Figs. 7F,G and EV5A).

Finally, to assess the relevance of the NGFR–MLC2 axis in the immune-resistant phenotype of human melanomas, we examined the expression levels of NGFR, pMLC2, and total MLC2 in melanoma cell lines derived from patients who were either responsive or resistant to immunotherapy. We observed a significant upregulation of NGFR, pMLC2, and total MLC2 in cells from immunotherapy-resistant patients compared to those from responders (Fig. EV5B–E). Consistent with this observation, NGFR expression correlated positively with an ameboid gene signature in two independent melanoma immunotherapy cohorts (Hugo et al, 2016; Riaz et al, 2017) when considering all patients (Fig. 7H) and remained evident when restricting the analysis to non-responders (Fig. 7I). In responders, the same relationship showed a similar direction but did not reach statistical significance (Fig. EV5F). Together, these data support an association between NGFR expression, ameboid-related programs and an immunotherapy-resistant phenotype in melanoma.

## Discussion

NGFR has been linked to phenotype switching and therapy resistance, including resistance to immunotherapy in melanoma (Arozarena and Wellbrock, 2019; Boshuizen et al, 2020; Restivo et al, 2017). Previous studies support that indirect targeting of NGFR is a promising approach for melanoma treatment (Boshuizen et al, 2020; Huang et al, 2021; Lehmann et al, 2023; Redondo-Munoz et al, 2023; Saltari et al, 2021). In this study, we have observed that the small molecule inhibitor of NGFR THX-B reduces distal metastasis and enhances the efficacy of immunotherapy. Importantly, combining THX-B with immune checkpoint inhibitors such as anti-PD-L1 or anti-PD-1 restores sensitivity in resistant tumors.

NGFR acts through association with multiple binding partners, activating three main pathways: NF-κB, JNK, and RhoA GTPase, mediating survival, apoptosis, and motility (Gentry et al, 2004; Roux and Barker, 2002; Bruno et al, 2023; Wu et al, 2021). Due to these diverse effects, it remains unclear whether NGFR agonists or antagonists would be more beneficial for melanoma treatment. Strategies that activate NGFR-mediated death pathways have shown success in reducing tumor growth (Goh et al, 2018; Redondo-Munoz et al, 2023; Saltari et al, 2021). Similarly, both direct (Morita et al, 2019; Ngo et al, 2016) or indirect (Boshuizen et al, 2020; Huang et al, 2021; Redondo-Munoz et al, 2023) blockade of NGFR has demonstrated anti-tumor efficacy. In our previous work, we used the small molecule NGFR inhibitor THX-B and found that it reduced melanoma lymph node metastasis; however, its impact on distant metastasis has not been tested (Garcia-Silva et al, 2021). In this work, we observed that NGFR knockout in melanoma cells or THX-B treatment did not affect primary tumor growth in melanoma models unless the tumors were intrinsically resistant to immunotherapy, suggesting that NGFR signaling plays a critical role in tumor survival under immune pressure. Interestingly, NGFR has been reported to promote immune evasion in melanoma by inhibiting the function of T and NK cells (Boshuizen et al, 2020; Furuta et al, 2014; Lehmann et al, 2023). We observed that NGFR depletion or inhibition reduced lung metastasis and prevented the development of immunotherapy resistance. Our data were consistent with a predominantly tumor cell–intrinsic component. THX-B reduced metastatic burden in nude mice, indicating that its anti-metastatic activity does not require an intact adaptive immune system. In addition, in the intrinsically anti-PD-L1-resistant YUMM1.1 model, the increase in CD8⁺ T-cell infiltration observed upon THX-B plus anti-PD-L1 was also recapitulated by tumor cell NGFR loss. However, we cannot exclude that THX-B may additionally influence immune compartments; this is particularly relevant given that NGFR signaling has been implicated at the interface between neurotrophin pathways and immune regulation (Bandola et al, 2017; Hernandez-Barranco et al, 2024).

NGFR expression has been associated with elevated PD-L1 levels, and both correlate with poor prognosis and reduced CD8⁺ T cell infiltration (Furuta et al, 2014; Hudson et al, 2020; Liu et al, 2021). In this study, we observed that murine NGFR^high^ IT-resistant tumors also exhibited increased expression of PD-L1. We found that NGFR deletion enhances T cell infiltration in immunotherapy-resistant tumors, while THX-B treatment amplifies T cell recruitment induced by anti-PD-L1 therapy. Our data support previous studies in organoid-immune cell co-cultures in which a combination of anti-PD-1 with NGFR knockdown increased the effector capacity of T cells (Sun et al, 2023). Taken together, these findings support the notion that targeting NGFR enhances tumor immunogenicity and provide a rationale for its combination with immune checkpoint inhibitors. Nonetheless, we acknowledge that murine melanoma models do not fully recapitulate the genomic heterogeneity, antigenic landscape, and immune complexity of human melanoma; therefore, future work will be required to validate these mechanisms across genetically diverse, patient-derived, and additional in vivo systems.

The spatial localization of tumor cells within the tumor and their interactions with the tumor microenvironment critically influence disease progression. NGFR regulates cell fate by controlling diverse processes such as survival, invasion, migration, stemness and metastasis-initiating potential (Boiko et al, 2010; Civenni et al, 2011; Hempstead, 2005; Radke et al, 2017; Redmer et al, 2014). We found that NGFR expression is enriched at the invasive front of 3D invasive spheroids, primary melanoma tumors, and metastatic patient samples, correlating with pMLC2. This pattern of NGFR localization at the invasive edge is not exclusive to melanoma; it has also been observed in other cancer types such as hypopharyngeal cancer and oral squamous cell carcinoma, where it associates with poor prognosis and early recurrence (Imai et al, 2013; Soland et al, 2008). These findings suggest that NGFR, through its role in invasion and interaction with the TME, may serve as a key marker of tumor aggressiveness across multiple cancer types.

A plausible regulator of NGFR activity at the invasive front is neurotrophin signaling, particularly NGF, which has been reported to promote melanoma cell migration/invasion through neurotrophin receptors including NGFR/p75NTR (Herrmann et al, 1993; Restivo et al, 2017). This regulation is likely context-dependent and may differ substantially between spheroid experiments and the in vivo setting. In vivo, melanoma cells at the tumor–stroma interface are in close proximity to diverse TME components, which can provide paracrine neurotrophins and additional cues that cooperate to induce or maintain NGFR expression/signaling (Marchetti et al, 1995). Consistent with this, NGF/proNGF and components of neurotrophin signaling have been described in melanoma specimens, supporting the presence of this axis in human disease (Boshuizen et al, 2020; Lehmann et al, 2023; Marsland et al, 2022). By contrast, spheroid systems primarily recapitulate intrinsic stress gradients and cell–cell/ECM constraints and therefore may capture only a subset of the microenvironmental signals that shape NGFR expression at the invasive front.

Beyond chemical gradients, ECM remodeling and stiffness also serve as potent inducers of ROCK-MLC2 mechano-transduction, reinforcing ameboid/invasive behaviors and providing a new biophysical route to NGFR-high phenotypes at the front through ROCK (Maiques et al, 2025). This dependence of the matrix also supports our observation that NGFR induces pMLC2 in low adhesion and/or 3D collagen spheroids rather than in 2D adherent plastic surfaces. Despite these events, at the invasive front of human melanomas, NGFR upregulation will likely reflect a convergence of intrinsic cell signaling routes, biophysical signals and inflammatory/stromal cues that are only partially recapitulated in spheroid systems. Indeed, the pro‑inflammatory cytokine TNF- α from infiltrating immune cells drives neural‑crest‑like invasive states in melanoma by inducing NGFR expression of melanoma cells (Landsberg et al, 2012). These considerations provide a mechanistic framework for the heightened NGFR expression we observe at the invasive front and support the notion that microenvironmental crosstalk plus mechanics synergize with the RhoA–ROCK–Myosin II axis to promote invasion and immunotherapy resistance.

Tumor cells located at the invasive front play an active role in aggressive and invasive behavior (Maiques and Sanz-Moreno, 2022). Indeed, interactions between tumor cells, immune cells, and the extracellular matrix predominantly occur at this tumor boundary (Mai et al, 2024; Sutherland et al, 2023). These areas are often immunosuppressive environments enriched in vasculature that allow local invasion (Fang et al, 2023; Nirmal et al, 2022). Interestingly, activation of pMLC2 in melanoma cells at the invasive front has been shown to recruit tumor-associated macrophages by inducing the secretion of factors such as NGFR (Georgouli et al, 2019), which in turn promote abnormal vasculature formation and facilitate tumor progression. Moreover, NGFR^high^ tumor cells located at the invasive tumor front have also been reported to exhibit elevated PD-L1 expression and were observed in close proximity to immune cells (Liu et al, 2021), suggesting that the invasive behavior of NGFR-expressing cells may create an immune-privileged microenvironment. However, the specific role of NGFR and the mechanisms involved was not described. Our data establish a novel functional link between NGFR and MLC2 at the invasive front in melanoma cells. We demonstrate that NGFR is necessary for MLC2 activation on both 2D and 3D matrices. NGFR knockout reduces phosphorylation of MLC2 at Thr18 and Ser19, along with the associated rounded cell morphology and invasive capacity on collagen. Mechanistically, NGFR activates ROCK through RhoA, promoting MLC2 stabilization and extending its half-life. The regulation of MLC2 protein stability remains relatively understudied. Data suggest that myosin turnover can involve ubiquitin–proteasome pathways (Acharyya et al, 2004; Gonczy, 2004; Manivannan et al, 2020). MLC2 ubiquitination has been reported and linked to specific E3 ligases, including MARCH8 and MuRF-1, although the upstream enzymes and context dependence are not fully defined (Guo et al, 2019; Witt et al, 2005). In our study, combined inhibition of NGFR and ROCK1/2 reduces pMLC2 levels and is accompanied by a decrease in total MLC2 abundance. Together with the partial rescue observed upon proteasome blockade, these data are consistent with proteasome-dependent regulation contributing to MLC2 downregulation under NGFR/ROCK inhibition. These findings extend the established phosphorylation-based control of MLC2 by suggesting an additional post-translational layer operating in a tumor context, and they motivate future work to evaluate therapeutic opportunities based on co-targeting NGFR and ROCK.

Beyond direct modification of MLC2 ubiquitination and degradation, ROCK/NGFR could impact on RhoA degradation or NF-κB signaling to modulate MLC2-dependent cytoskeleton dynamics since both are highly regulated by ubiquitination and deubiquitination (Wang et al, 2003)-(Sun, 2017). Indeed, NGFR, in addition to RhoA activation (Blochl et al, 2004), may influence MLC2 activation through NF-κB (Roux and Barker, 2002), while ROCK/MLC2-mediated activation of NF-κB, in turn, enhances NGFR expression, creating a regulatory feedback loop that sustains melanoma invasiveness.

Activation of ROCK-MLC2 ameboid program at the tumor-host interface has already been associated with enhanced metastatic potential and resistance to therapy (Georgouli et al, 2019; Orgaz et al, 2020). Ameboid pMLC2^+^ cells at the IF are both invasive and proliferative (Rodriguez-Hernandez et al, 2020). However, NGFR^+^/pMLC2^+^ IT-resistant cells form smaller yet more aggressive tumors. This suggests that double NGFR^+^/pMLC2^+^ cells constitute a different subset of ameboid non-proliferative invasive cells that may be responsible for metastasis and therapy resistance in melanoma. We hypothesize that NGFR is a key component of an actomyosin-dependent program at the tumor invasive front, characterized by an immunosuppressive state conductive to metastatic colonization. Taken together, targeting NGFR/pMLC2-positive cell populations offers a promising therapeutic strategy, as these cells are highly invasive, possess stem-like properties, evade anti-tumor immunity and contribute to therapy resistance.

Our previous work using melanoma patients’ samples showed that NGFR expression is elevated in local metastatic sites such as lymph nodes. This increased expression correlates directly with poorer patient survival, indicating that NGFR is a reliable marker for early melanoma metastasis in lymph nodes (Garcia-Silva et al, 2021). Moreover, although other studies have demonstrated a correlation between NGFR expression and immunotherapy resistance (Boshuizen et al, 2020), its potential as a predictive marker for treatment response has not been analyzed. Building on these findings, our analyses indicate that higher tumor NGFR expression is associated with earlier relapse and reduced progression-free survival in immunotherapy-treated melanoma patients. Moreover, NGFR-based readouts at the invasive front of the tumors, including NGFR-to-pMLC2 metrics, were associated with subsequent development of distant metastases and resistance to therapy. However, as some of the transcriptomic analyses rely on bulk RNA sequencing, these signals may also incorporate contributions from the tumor microenvironment in addition to those mediated by tumor-intrinsic NGFR expression. Taken together, our results are consistent with NGFR being linked to metastatic progression and reduced benefit from immune checkpoint therapy, and they support the potential utility of NGFR/pMLC2 as stratification biomarkers to identify patients at higher risk of early metastasis and/or primary resistance—an approach that will require prospective validation.

Our findings also provide a rationale to explore combinations of NGFR-targeted approaches (such as THX-B) with immune checkpoint blockade to limit metastatic dissemination and potentially delay or overcome resistance. Although combining immunotherapy with targeted therapies can improve patient outcomes, clinical implementation is often constrained by toxicity. NGFR signaling and ROCK/MLC2-dependent contractility have been linked to resistance to both targeted therapies and immunotherapies (Boshuizen et al, 2020; Mehta et al, 2018; Orgaz et al, 2020); suggesting that targeting this axis may be beneficial in selected contexts. ROCK inhibitors have been tested but exhibit narrow therapeutic windows and adverse effects (McLeod et al, 2020). Additionally, in our preclinical models, THX-B reduced metastatic burden and improved disease control in combination with checkpoint blockade without evident additional toxicity under the conditions tested. These data support further evaluation of NGFR-based combinations, particularly in settings of primary or acquired immunotherapy resistance.

## Methods

**Table:** Reagents and tools table

Reagent/resource	Reference or source	Identifier or catalog number
**Experimental models**		
B16-F1 cells	ATCC	ATCC CRL-6323
B16-F10 cells	ATCC	ATCC CRL-6475
YUMM1.1 cells	Dr. M. Soengas (Spanish National Cancer Research Centre, Spain)	N/A
YUMM1.7 cells	Dr. M. Soengas (Spanish National Cancer Research Centre, Spain)	N/A
YUMMER1.7 cells	Dr. M. Soengas (Spanish National Cancer Research Center, Spain)	N/A
SK-MEL-147 cells	Dr. M. Soengas (Spanish National Cancer Research Center, Spain)	N/A
A375P cells	Dr. Sanz-Moreno (The Institute of Cancer Research, UK)	ATCC CRL-1619
A375M2 cells	Dr. Richard Hynes (HHMI, MIT, USA) via Sanz-Moreno (The Institute of Cancer Research, UK))	N/A
5555 mouse melanoma cells	Dr. Richard Marais (Cancer Research UK Manchester Institute)	N/A
Patient-derived melanoma cell lines (#201, #314, #707, #218, #227, #522)	Dr. Mitchell Levesque (University of Zurich, Switzerland)	N/A
HEK293T cells	Dr. Juan Mendez (Spanish National Cancer Research Center, Spain)	N/A
C57BL/6 J mice (in-house)	CNIO animal facility / Charles River	IMSR_JAX:000664
Athymic Nude-Foxn1nu mice	Envigo	069
**Recombinant DNA**		
lentiCRISPRv2	Addgene	#52961
pMD2.G	Addgene	#12259
psPAX2	Addgene	#12260
pFUGW-FerH-ffLuc2-eGFP (GFP-Luc lentiviral vector)	Addgene	#71393
pLV-mCherry-NGFR > EGFP	VectorBuilder	VB200830-1119xmw
**Antibodies**		
anti-beta-ACTIN (WB 1:10,000)	Sigma-Aldrich	#2228
anti-GAPDH (WB 1:10,000)	Abcam	ab8245
anti-phospho-ERK1/2 (p42/p44 MAPK) (WB 1:1000)	Cell Signaling Technology	#9101
anti-ERK1/2 (p44 MAPK) (WB 1:1000)	Cell Signaling Technology	#9102
anti-human/mouse NGFR (WB 1:1000)	Abcam	ab52987
anti-human NGFR nori138B (WB 1:500)	Generated at CNIO and Abcam	ab271289
anti-mouse NGFR nori146C (WB 1:500)	Generated at CNIO and Abcam	ab271290
anti-phospho pThr18/S19 pMLC2 (WB 1:1000)	Cell Signaling Technology	#3674
anti-MLC2 (WB 1:1000)	Cell Signaling Technology	#3672
anti-RhoA (WB 1:1000)	Cell Signaling Technology	#2117
Amersham ECL anti-rabbit IgG-HRP (WB 1:5000)	Cytiva	NA934V
Amersham ECL anti-mouse IgG-HRP (WB 1:5000)	Cytiva	NA931V
anti-rat IgG-HRP (WB 1:5000)	Thermo Fisher Scientific	#31470
IRDye 800CW anti-rabbit IgG (WB 1:5000)	LI-COR	#926-32211
Alexa Fluor 680 anti-mouse IgG (WB 1:5000)	Thermo Fisher	#A21057
IRDye 680RD anti-rat IgG (WB 1:5000)	LI-COR	#925-68076
NGFR (IHC 1:100)	Invitrogen	MA5-13314
pMLC2 (IHC 1:100)	Cell Signaling Technology	#3671
MelanA/MLANA (IHC 1:200)	Novus Biologicals	NBP1-30151
S100 (IHC 1:600)	Novocastra/ Leica	NCL-L-S100p
anti-human HMB45 (IHC)	Dako/Agilent	IR052
anti-human NGFR nori138B (IHC 1:50)	Monoclonal Antibody Core Unit, CNIO	AM138
anti-human/mouse pMLC2 (IHC 1:100)	Cell Signaling Technology	#3671
anti-mouse HMB45-Mart1 (IHC)	Abcam	ab732
anti-mouse PD-L1 (IHC 1:2000)	Abcam	ab213480
anti-mouse NGFR nori146C (IHC 1:5)	Monoclonal Antibody Core Unit, CNIO	AM146
anti-mouse CD8 (IHC 1:200)	Monoclonal Antibody Core Unit, CNIO	AM94A
anti-rat secondary (IHC)	Vector Laboratories	BA-9400-1.5
anti-rabbit secondary (IHC)	Vector Laboratories	AI-5000-1.5
anti-mouse secondary-HRP (IHC)	Abcam	ab6789
anti-human NGFR NORI138B (IF 1:100)	Abcam	ab271289
anti-human pS19 pMLC2 (IF 1:200)	Cell Signaling Technology	#3671
Alexa Fluor Rabbit 555msecondary antibody series (IF 1:500)	Thermo Fisher	A31572
Alexa Fluor 647 Rat secondary antibody (IF 1:500)	Thermo Fisher	A21247
anti-PD-L1 monoclonal antibody (clone 10 F.9G2; in vivo 10 mg/kg)	BioXCell	BE0101
anti-PD-1 monoclonal antibody (clone RMP1-14; in vivo 10 mg/kg)	BioXCell	BE0146
Rat IgG2b isotype control (clone LTF-2)	BioXCell	BE0090
Rat IgG2a isotype control (clone 2A3)	BioXCell	BE0089
CD3 antibody (IHC 1:200)	Dako/Agilent	A0452
**Oligonucleotides and other sequence-based reagents**		
sgRNA Ngfr (murine): 5’-AGGTGCTGCCTGCAGCGCCA-3'	This work/cloned into lentiCRISPRv2	N/A
sgNGFR1 (human): 5’-GGTAGTAGCCGTAGGCGCAG-3'	This work	N/A
sgNGFR2 (human): 5’-GTGTGGACCGTGTAATCCAA-3'	This work	N/A
sgRNA Rosa26 control (murine): 5’-GGTAGGCCTAGCACATGATC-3'	This work	N/A
sgRNA AAVs1 control (human): 5’-CCTCTAAGGTTTGCTTACGA-3'	This work	N/A
TaqMan probe NGFR (murine): Mm00446296_m1	Applied Biosystems/Thermo Fisher Scientific	Mm00446296_m1
TaqMan probe HPRT (housekeeping gene)	Applied Biosystems/ Thermo Fisher Scientific	Mm03024075
**Chemicals, enzymes and other reagents**		
DMEM High Glucose	Sigma-Aldrich	D5796
Ham F12 medium	Gibco/ Thermo Fisher Scientific	31765027
MEM non-essential amino acids solution	Gibco/Thermo Fisher Scientific	#11140050
Fetal bovine serum (FBS)	Thermo Fisher Scientific	S1810-500
L-Glutamine (2 mM)	Gibco/ Thermo Fisher Scientific	15410314
Sodium pyruvate (10 mM)	Gibco/Thermo Fisher Scientific	S8636
Gentamicin (20 ug/mL)	Gibco/Thermo Fisher Scientific	15710064
RPMI 1640 medium	N/A	11530586
THX-B33 (NGFR small molecule inhibitor; 20 uM)	Dr. H.U. Saragovi/ Experimental Therapeutic Program, CNIO	N/A
ROCKi GSK269962A (1 uM)	TOCRIS	4009
Cycloheximide (20 ug/mL)	Merck	C4859
MG132 (10 uM)	Merck	C2211
Polybrene (8 ug/mL)	Sigma-Aldrich	TR1003
Puromycin (1 ug/mL)	InvivoGen	P8833
Normocin (50 ug/mL)	InvivoGen	ant-nr-1
Lipofectamine 2000	Life Technologies/Thermo Fisher Scientific	11668019
RIPA	Merck	R0278
Protease inhibitors	Merck	11836170001
Phosphatase inhibitors	Merck	4906845001
Pierce BCA Protein Assay Kit	Thermo Fisher Scientific	23227
ECL Western Blotting Substrate Kit	GE Healthcare/ Cytiva	RPN 2106
EndoFree Plasmid Maxi Kit	QIAGEN	12362
RNeasy Kit (RNA extraction)	QIAGEN	74104
QuantiTect Reverse Transcription Kit	QIAGEN	205311
TaqMan Gene Expression Master Mix	Applied Biosystems/ Thermo Fisher Scientific	4369016
Opal Multiplex IHC Kit	Akoya Biosciences	NEL871001KT
DAPI (5 ug/mL)	Merck	D9542
Prolong Gold Antifade Mountant	Thermo Fisher Scientific	11539306
Paraformaldehyde 4% (PFA)	N/A Electron Microscopy Sciences	157-4-1 L
Triton X-100 (0.1%)	N/A Sigma-Aldrich	X-100
Rat Tail Type I Collagen (5 ug/mL and 2.2 mg/mL)	Millipore	08-115
Fibrillar bovine dermal collagen PureCol (1.7 mg/mL)	Advanced BioMatrix	5005-B
GST-Rhotekin RBD beads (RhoA pulldown)	Cytoskeleton	#RT02
Liberase TH (75 ug/mL)	Roche Diagnostics	LIBTH-RO
DNase I (1 ug/mL)	Sigma-Aldrich	10104159001
BioCoat Matrigel Invasion Chamber (8-um pores)	Corning	#354480
Transwell filters 6.5 mm, 8-um pores	Costar	#3422
0.45-um low-binding protein filter	Corning	CLS431220
**Software**		
GraphPad Prism v5.03	GraphPad Software	N/A
ImageJ/Fiji v1.54 f	NIH	RRID:SCR_003070
Image Studio Lite v5.2	LI-COR Biosciences	N/A
QuPath v0.4.4 and v0.5.1	Bankhead et al, 2017	N/A
inForm software	Akoya Biosciences	N/A
CellProfiler v4.2.6	Broad Institute	N/A
Imaris v7.3	Oxford Instruments/ Bitplane	N/A
LAS-X software v3.75	Leica Microsystems	N/A
LAS AF v2.73 / v2.75	Leica Microsystems	N/A
ZEN software	Zeiss	N/A
DefiniensXD v2.5	Definiens	N/A
R package coxme v2.2-17	Therneau T	N/A
**Other**		
Xenogen IVIS-200 bioluminescence imaging system	PerkinElmer	N/A
QuantStudio 6 Flex Real-Time PCR System	Applied Biosystems/ Thermo Fisher Scientific	N/A
BD Influx Cell Sorter	BD Biosciences	N/A
Bond RXm Autostainer	Leica Biosystems	N/A
Vectra Polaris automated quantitative pathology imaging system	Akoya Biosciences	N/A
Discovery ULTRA automated immunostaining platform	Ventana-Roche	N/A
Autostainer Link 48	Dako/Agilent	N/A
AxioScan Z1 slide scanner	Zeiss	N/A
TCS-SP8 STED 3X confocal microscope (63x/NA1.4 oil objective)	Leica Microsystems	N/A
TCS-SP5 confocal microscope	Leica Microsystems	N/A
Thunder imaging system	Leica Microsystems	N/A
Tissue arrayer device	Beecher Instruments	N/A
Odyssey CLX imaging system	LI-COR Biosciences	N/A

### Cell lines and reagents

Low metastatic B16-F1 and high metastatic B16-F10 cells were purchased from ATCC. Mouse BRAF^V600E^ YUMM 1.1 (Meeth et al, 2016) and human melanoma SK-MEL-147 cell lines were kindly provided by Dr. M. Soengas (Spanish National Cancer Research Center, Spain). YUMMER1.7 cell line was generated from BRAF^V600E^ YUMM 1.7 cells upon irradiation with UVB (Wang et al, 2017). Human melanoma A375P cells were purchased from ATTC (Clark et al, 2000; Sanz-Moreno et al, 2008), A375M2 cells were obtained from Dr. Richard Hynes (HHMI, MIT, USA) (Clark et al, 2000) by Dr. Sanz-Moreno. Braf^V600E^ mouse melanoma cell line 5555 (established from the Braf^+/LSL-V600E^;Tyr::CreERT2^+/o^;p16^INK4a−/−^ C57BL/6 mouse model (Orgaz et al, 2020). Cells have been STR-tested and routinely checked for mycoplasma.

Melanoma cell lines were grown in high-glucose Dulbecco’s modified Eagle’s High-Glucose medium (DMEM, Sigma-Aldrich) except YUMM cells that were grown in F12/DMEM supplemented with MEM Non-Essential Amino Acids Solution (#1140050, Gibco). All media were supplemented with 10% fetal bovine serum (FBS, Thermo Fisher Scientific), 2 mM glutamine (Sigma-Aldrich), 10 mM sodium pyruvate (Sigma-Aldrich) and 20 μg/mL gentamicin (Sigma-Aldrich). Melanoma cells were grown at 37 °C in a humidified 5% CO_2_ atmosphere or, in the case of A375P and A375M2, 10% CO_2_ atmosphere.

Patient-derived melanoma cell lines (#201, #314, #707, #218, #227, and #522) were generated from biopsies of metastatic melanomas at the University Hospital Zurich after ethical regulations compliance (see Ethical compliance section). Cells were grown in RPMI 10% FBS. Patient #201 and #314 cell lines were established from lymph node metastasis of patients without neoadjuvant treatment before surgery. Patient #707 cell line was derived from a dermal/subcutaneous metastasis of a patient who received one dose of ipilimumab (anti-CTLA-4) and showed a complete response upon pembrolizumab (anti-PD-1) treatment after surgery. Patient-derived resistant cell lines #218, #227, and #522 were established from subcutaneous metastases of patients who received combinations of ipilimumab plus nivolumab (anti-PD-1); pembrolizumab (anti-PD-1) plus epacadostat (IDO1) or pembrolizumab plus epacadostat followed by ipilimumab before surgery, respectively. All resistant patients showed progressive disease.

When indicated, melanoma cells were treated with the following compounds: NGFR small molecule inhibitor THX-B (Bai et al, 2010) (20 µM; provided either by Dr. H.U. Saragovi or synthesized by the experimental therapeutic program at CNIO); ROCKi GSK269962A (1 μM; TOCRIS); Cycloheximide (20μg/ml; #C4859 MERCK), and MG132 (10 μM, #C2211 MERCK) as indicated in the figure legends.

### CRISPR editing and shRNA tools

Knockout cell lines were generated using the CRISPR/Cas9 system. Two different sgRNAs targeting NGFR were used in both murine and human models. In murine cell lines, the *Ngfr* target sequence 5’-AGGTGCTGCCTGCAGCGCCA-3´ was cloned into the lentiCRISPRv2 (Addgene plasmid #52961). For the human models, we used sgNGFR1 sequence 5´-GGTAGTAGCCGTAGGCGCAG-3´ and sgNGFR2 sequence 5´-GTGTGGACCGTGTAATCCAA-3´. As control, we used sgRNA Rosa 26 5´-GGTAGGCCTAGCACATGATC-3´ sgRNA and AAVs1 5´-CCTCTAAGGTTTGCTTACGA-3´ for the murine and human cells, respectively. Lentiviruses were prepared as previously described (Torres-Ruiz et al, 2017). Briefly, 9 μg of target lentiviral vector, prepared with the EndoFree Plasmid Maxi Kit (QIAGEN, USA), was transfected into HEK293T cells together with 3 μg of pMD2.G (Addgene plasmid #12259), 5 μg of psPAX2 (Addgene plasmid #12260), and 62.5 μl of Lipofectamine™ 2000 (Life Technologies) in a 10-cm dish plate. Forty-eight hours later, supernatant containing lentiviruses was recovered and filtered through a 0.45-um low-binding protein filter (Millipore). Cells were transduced with lentiviral supernatants at low MOI in suspension. 200,000 cells were seeded in M6 well plates, followed by the addition of the corresponding lentiviral suspension diluted 1:4 in the presence of 8 µg/ml polybrene. Cells were selected with 1 µg/ml of puromycin (InvivoGen). Expression of wildtype and NGFR KO was verified through Western blot. NGFR KO cells were further selected from negative NGFR expression using a BD Influx cell sorter.

### Generation of GFP-Luc and pLV-mCherry-NGFR-GFP models

GFP-Luciferase models and A375P pLV-mCherry-NGFR-GFP cells were generated by the transduction of a GFP-Luc lentiviral (pFUGW-FerH-ffLuc2-eGFP, #71393 Addgene) or the pLV-mCherry-NGFR > EGFP (VB200830-1119xmw, VectorBuilder) construction. Lentiviruses were produced as described in the section before. Forty-eight hours after transfection, supernatant containing lentiviruses was recovered and filtered through a 0.45-um low-binding protein filter (Millipore). For the generation of stable melanoma cell lines expressing GFP-Luc, 200,000 cells were infected in suspension with 1.5 ml of virus-conditioned media (ratio 1:4–1:8) in the presence of 8 µg/ml Polybrene (Sigma-Aldrich), followed by a second round of infection in adhesion on the T6 cell plate 24–48 h later. Cells are expanded and sorted by selection of GFP or mCherry positive expression using the BD Influx cell sorter.

### Mice

For in vivo experiments, 8–10 weeks-old male C57BL/6J (generated in-house at the CNIO animal facility) or Athymic Nude-Foxn1nu (nude, Envigo) mice were used. The effect of THX-B and anti-PD-1 therapies in the survival and development of YUMMER1.7 tumors were performed in 10–12 weeks-old male C57BL/6J purchased from Charles River (Laval, Quebec, Canada). About 5555 tumor growth assessment was performed in 5–7-week-old female C57BL/6J mice from Charles River UK. Tumors were allowed to establish, sizes (average 60–100 mm^3^) were matched, and then mice were randomly allocated to groups of 6–8 animals. No blinding was used in the treatment schedules for these experiments since the different treatments were identified by ear notching/mark on the tail. Based on previous studies in the literature (Hong et al, 2017; Kong et al, 2018) and our own experience, groups of 6–8 animals were used to have sufficient animals per group to provide statistically significant data while keeping the number of animals used to a minimum. Mice were given access to food and water ad libitum and housed in cages of five mice maximum at 21 °C ± 2 °C, and the humidity was 50–60%. Light cycle was light:13 h/dark:11 h.

### THX-B and immunotherapy experiments

SK-MEL-147-GFPLuc (300,000); B16-F10-GFPLuc (200,000), YUMM1.1 (1 million), YUMMER1.7 (600,000), and 5555 (1 million) cells were subcutaneously injected into the flank of 8–10-week-old male nude (SK-MEL-147 cells) or C57BL/6J (murine models) mice. After 7 days, mice were randomly allocated into groups of 4–7 animals and treated twice a week with THX-B (5 mg/kg, intraperitoneally (i.p)) or vehicle (1% DMSO in PBS) and/or the immunotherapies anti-PD-L1 monoclonal antibody (B7-H1; InVivoMab clone 10 F.9G2™, BioXCell #BE0101, 10 mg/kg, i.p) or anti-PD-1 monoclonal antibody (InVivoPlus clone RMP1-14, BioXCell #BE0146, 10 mg/kg, i.p.). Rat IgG2b (clone LTF-2 BioXCell #BE0090) or rat IgG2a isotype control (clone 2A3 BioXCell # BE0089) were used as vehicles for anti-PD-L1 or anti-PD-1 experiments, respectively.

Tumor growth was monitored twice a week by measurement of the two orthogonal large and small external diameters (a, b) with a caliper and tumor volume was calculated by the formula: a * b^2^ *  0.5236. When tumors reached 1–1.5 cm^3,^ mice were sacrificed, and tumors and lungs were collected and analyzed by ex vivo bioluminescence imaging (BLI) using a Xenogen IVIS-200 machine (PerkinElmer). Afterward, tissues were fixed in 4% PFA and paraffin-embedded for histological analysis.

### Anti-PD-L1 and PD-1 resistant cell lines generation from in vivo tumors

Anti-PD-L1-resistant B16-F10-R cell lines were established from 1000 mm^3^ tumors. Anti-PD-L1 non-responder B16-F10-GFPLuc tumors were mashed with a p1000 tip to unbundle cells and filtered through a 100-μm strainer. After washing with 10 ml of DMEM plus 10% FBS, cells were spun down by centrifugation (5 min, 1000 rpm), washed with PBS and treated with 1 ml of trypsin for 3 min. We added 10 ml of complete media, filtered again through 100 μm strainers, spun and resuspended cells in 10 ml of complete DMEM 10% FBS plus normocin (50 μg/ml; ant-nr-1 Invivogen). Tumor cells were selected by positive GFP expression using a BD Influx cell sorter.

#### Generation of 5555 cells resistant to anti-PD-1

Anti-PD-1-resistant 5555 cells were generated as described in (Orgaz et al, 2020). Briefly, 1 million 5555 cells were subcutaneously injected into the right flank of 5–7-week-old female C57BL/6J mice. After 7–14 days, mice with tumors (50–80 mm^3^) were randomly allocated into groups of 6–7 animals and treated every 3 days with anti-PD-1 monoclonal antibody (InVivoPlus clone RMP1-14, BioXCell #BE0146) (10 mg/kg, intraperitoneally (i.p.)) or rat IgG2a isotype control (clone 2A3 BioXCell # BE0089). Anti-PD-1-non-responder (NR) lines were established in culture by digesting tumors with a mixture of Liberases (TH and TM, 75 μg/ml each, Roche Diagnostics) and 1 μg/ml DNase I (Sigma) in HBSS for 1 h at 37 °C with shaking and then passed through 100 μM strainers.

### Patient samples and tissue microarray (TMA)

To analyze NGFR at the invasive front of melanoma patients’ samples and correlate it with metastatic progression, we used formalin-fixed paraffin-embedded (FFPE) of human primary melanomas (53 patients), and metastasis (45 patients) obtained from Hospital Arnau de Vilanova (HUAV), Lleida and Hospital de Bellvitge, Barcelona (Spain), see tables S2-S3 from (Rodriguez-Hernandez et al, 2020). We also analyzed another two cohorts from 45 metastatic melanoma patients categorized as responders and non-responders to immunotherapy with a clinical history of relapse after the first IT treatment and 46 primary melanoma samples. The clinical information is summarized in Tables EV1 and EV2. Patients were staged and classified according to the American Joint Committee on Cancer Melanoma Staging and Classification criteria (Balch et al, 2009). A tissue arrayer device (Beecher Instruments, Silver Spring, MD, USA) was used to construct the tissue microarrays (TMA). The IF was defined histologically by board-certified pathologists based on H&E and/or MLANA staining. Cores were annotated as TB (central tumor mass) or IF (peripheral region at the tumor–stroma interface where tumor cells infiltrate the surrounding tissue).

### Multiparameter immunohistochemistry staining of TMAs

All immunohistochemistry staining procedures were conducted on a Bond RXm Autostainer (Leica Biosystems) to ensure consistency and reproducibility. After deparaffinization and rehydration, antigen retrieval was carried out by heating the slides in citrate buffer ER2 for 40 min. Slides were incubated with the following primary antibodies: NGFR (1:100 cat.nr. MA5-13314 Invitrogen), pMLC2 (1:100 cat.nr. #3671 CST), Mel A (1:200 cat.nr. NBP1-30151 Novus Biologicals), and S100 (1:600 cat.nr. NCL-L-S100p Novocastra) at 37 °C. The primary antibodies were visualized using the Opal™ staining system (cat.nr NEL871001KT Akoya Biosciences), which involves tyramide signal amplification (TSA) for enhanced detection. After incubation with secondary antibodies conjugated to horseradish peroxidase (HRP), tyramide-conjugated fluorophores were applied to detect specific markers. The channels used for the experiment are Opal 520/CD3, Opal 570/NGFR, Opal 620/pMLC2 and Opal 690/Mel A and S100. Stained TMAs were imaged using a Vectra® Polaris™ Automated Quantitative Pathology Imaging System (Akoya Biosciences). Multispectral images were analyzed with inForm® software to quantify marker expression and assess co-localization.

### Image processing and analysis of the TMAs

QuPath 0.4.4 was used for NGFR analysis in the TMAs. Cores (1 mm) were identified with the TMA grid, and the de-arrayed command and tissue detection excluded background and artifact. Cell detection used Watershed Cell Detection. Color deconvolution (H-DAB) was applied, and per-cell OD intensities were measured. A single threshold for binary categorization of positive or negative cells for the staining of interest was chosen. Cells were then classified as tumor, immune, or stroma using intensity thresholds and morphology features (Random Forest trained on pathologist-labeled ROIs), and per-core counts/densities were exported for downstream analyses. The number of positive cells was normalized by the number of cell detections per mm^2^.

In the TMA from Responders and non-Responders, consecutively cut slides were stained with one staining at a time as follows: NGFR, MelanA and S100. To account for variable tumor content across cores, we computed a tumor-content proxy as the mean fraction of S100⁺ cells and MelanA⁺ cells per core (S100_MelanA_mean). NGFR positivity was then adjusted as: NGFR_percent_positive × S100_MelanA_mean. These adjusted NGFR fractions were then correlated with the time from patients’ first PD-1 infusion until the clinical evaluation of progression (TTP, time-to-progression). Because multiple TMA spots were prepared per patient (range 1 to 4), we analyzed the effect of NGFR on TTP using a mixed-effects Cox proportional hazard model with patient ID as a random intercept, using the R package “coxme” version 2.2-17.

To analyze multiplex IHC of TMAs, the Inform® software was trained on 6 ROIs, each corresponding to 1 TMA core, to develop a tissue and cell segmentation algorithm to be applied to all samples (Fig. EV5E). Images were spectrally unmixed, then ROIs were segmented into tumor/stromal compartment (positive or negative for S100 and Mel A staining and CD3 staining (1:200 cat.nr. A0452 Dako), respectively and positive/negative for NGFR and/or pMLC2), and background compartment (negative for S100, Mel A, NGFR, and pMLC2 staining). Next, ROIs underwent a cell segmentation procedure, which was based on nuclear recognition with positive DAPI staining. The Inform® algorithm was trained, and tissue and cell segmentation of the remaining cores was performed. Each core was manually checked for the accuracy of the tissue and cell segmentation procedure and compared with IHC as a reference. Each cell was called positive/negative for each marker, by fitting a two-component additive mixture model to a marker intensity histogram. To render the marker intensity distribution parametric, intensity was first transformed by taking the logarithm, the square root, or the hyperbolic arc-sine. Each cell is thus assigned a probability of being positive for a specific cell marker. Cells with a probability ≥0.99 of positivity were assigned as positive.

### Quantitative qRT-PCR

Total RNA was extracted from cells using the RNeasy kit (Qiagen), and reverse transcription (1 mg) was applied using the QuantiTect Reverse Transcription kit (Qiagen). A total of 20 ng of total cDNA were then subjected to Quantitative real-time PCR (qRT-PCR) using TaqMan Gene Expression Master Mix and predesigned TaqMan probes for *NGFR* (*Mm00446296_m1*, Applied Biosystems). Assays were performed in triplicate on a QuantStudio 6 Flex System (Applied Biosystems). Relative expression was calculated following the delta delta Ct (DDCt) method. *HPRT* was used as the housekeeping gene.

### Immunoblotting

Cells were lysed in RIPA buffer supplemented with protease/phosphatase inhibitors (Roche). For p-Myosin and Myosin detection, cells were lysed in Laemmli buffer (60 mM Tris-HCl, pH 6.8, 10% Glycerol, 2% SDS) and frozen on ice immediately. Then, samples were boiled 5–8 min at 95 °C, followed by 20 s of sonication in a cold-water bath. Clear supernatants were obtained after 20 min centrifugation at 4 °C. Protein concentration was determined by Pierce BCA assay (Thermo Fisher Scientific). Equal amounts of protein were resolved by SDS-PAGE and probed with the following primary antibodies: anti-β-ACTIN (dil: 1/10,000; #2228, Sigma-Aldrich), GAPDH (dil: 1/10,000, ab8245, Abcam), anti-phospho ERK1/2 (p42/p44MAPK) (dil: 1/1000, #9101, Cell signaling), anti-ERK1/2 (p44 MAPK) (dil: 1/1000, #9102, Cell signaling); anti-human/mouse NGFR (dil:1/1000; ab52987, Abcam), anti-human NGFR nori138B (dil: 1/500 ab271289, Abcam), anti-mouse NGFR nori146C (dil 1:500, ab271290, Abcam); anti-phospho pThr18/S19 pMLC2 (dil 1/1000, #3674, cell signaling), anti-MLC2 (dil 1/1000, #3672, cell signaling). The secondary antibodies used were Amersham™ ECL™ peroxidase-linked anti-rabbit IgG (dil: 1/5000, NA934V), anti-Mouse (dil: 1/5000, NA931V) and anti-Rat IgG (dil: 1/5000, #31470, Thermo Fisher) or the fluorescent-probed antibodies IRDye® 800CW anti-Rabbit (dil:1/5000, #926-32211, LI-COR), Alexa Fluor™ 680 anti-Mouse (dil: 1/5000, #A21057, Licor) and IRDye® 680RD anti-Rat (dil: 1/5000, #925-68076, LI-COR). Signals were detected using the ECL Western Blotting Substrate kit (GE Healthcare) or the Odyssey CLX system (Licor). The intensities of immunoreactive bands were quantified by densitometry using ImageJ software (ImageJ, RRID:SCR_003070) or Image Studio Lite Ver 5.2 (LI-COR).

### Histological analyses

Tissue samples were fixed in 10% neutral buffered formalin (4% formaldehyde in solution) and embedded in paraffin. Tissue samples were cut into 2.5-µm-thick sections, mounted on Superfrost®Plus slides and dried overnight. Several immunohistochemistry reactions were performed in an automated immunostaining platform (Discovery ULTRA, Ventana-Roche or Autostainer Link 48, Dako). Antigen retrieval was first performed with the appropriate method, and endogenous peroxidase activity was quenched with 3% hydrogen peroxide. Then, slides were incubated with the appropriate primary antibodies: human anti-HMB45 (Dako, Agilent (IR052)); human NGFR nori138B (Dil 1:50, Monoclonal Antibody Core Unit, CNIO (AM138)), human/mouse anti-pMLC2 (Dil 1:100, #3671, Cell Signaling) and mouse anti-HMB45-Mart1 (ab732, abcam), mouse anti-PD-L1 (Dil 1:2000, ab213480, Abcam); mouse anti-NGFR (NGFR nori146C, dil 1:5, Monoclonal Antibody Core Unit, CNIO (AM146)) and mouse anti CD8 (Dil 1:200, clone OTO94A, Monoclonal Antibody Core Unit, CNIO (AM94A)). After that, they were incubated with the corresponding secondary antibodies anti-rat, anti-rabbit (BA-9400-1.5, AI-5000-1.5, Vector Laboratories, respectively) or anti-mouse (ab6789, Abcam) conjugated with horseradish peroxidase. Immunohistochemical reaction was developed using 30- diaminobenzidine tetrahydrochloride, magenta or purple chromogen, using Envision FLEX, Novolink polymer or OmniMap Discovery Ultra platforms following the manufacturer’s protocols. Nuclei were counterstained with Harrys’s hematoxylin. Positive control sections known to be primary antibody positive were included for each staining run.

Four slides of each tumor were acquired with a slide scanner (AxioScan Z1, Zeiss) and Image analysis was performed with QuPath-0.5.1 and ZEN software (Zeiss). For CD8 and PD-L1 analysis, a binary positivity value was assigned. First, we set the color deconvolution stains based on the level of hematoxylin and DAB/purple staining. An object classifier was trained to distinguish cell detection based on hematoxylin presence. Then, we generated a pixel classifier to select the whole tumor, which we called “Tissue detection” to be able to automate the process of tissue selection. Finally, we ran the script fusing these parts, obtaining the number of CD8^+^ T cells, as well as other data such as the total number of cells or area which we used to relativize. Expression values were quantified based on intensity thresholds: 0 for negative, 1 for low, 2 for moderate, and 3 for high intensity. The H-score was calculated as: H-score = (0 × % negative) + (1 × % low positive) + (2 × % moderate positive) + (3 × % high positive); the final score ranges from a minimum of 0 (negative) to a maximum 300 (100% high). In cases requiring evaluation of differences between the IF and tumor core, an outer 200 µm diameter was applied within the tumor (IF), and expression values were computed accordingly in both scenarios. The quantification of pMLC2 in resistant tumors was performed in the same manner as the initial steps for detecting CD8 and PD-L1. However, we use an additional step to quantify the differences of pMLC2^+^ areas in the IF. For this purpose, we considered the larger size of the analyzed tumors, which in this case measured 1000 µm.

### Immunofluorescence

After fixation with PFA 4% and washing three times with PBS, cells were permeabilized with PBS 0.1% Triton X-100 for 10 min. Non-specific sites were blocked by incubation in a blocking solution containing 1% BSA 5% Donkey/Goat Serum in PBS for 1 h at RT. Cells were then incubated 1 h or overnight with primary antibodies (anti-human NGFR NORI138B dil1:100 ab271289, anti-human pS19 pMLC2 dil1:200 #3671, Cell Signaling) prepared in PBS 1% BSA. After three washes with PBS-0.01% Tween, sections were incubated for 1 h with secondary antibodies from the Alexa Fluor series from Molecular Probes (Thermo Fisher, dil1/500) and washed again. Finally, samples were stained with DAPI (5μg/ml, 20 min) and mounted with Prolong Gold (Thermo Fisher). Fluorescent images were obtained using a TCS-SP8 STED 3X confocal microscope (Leica Microsystems) equipped with a 63X/NA1.4 oil immersion objective, AOBS, a tunable white laser and LAS-X software v3.75. A z-stack was acquired, and then the maximum intensity projection (MIP) was calculated with Fiji software v1.54f (Schindelin et al, 2012) for each field of view (FOV). MIP images were then analyzed with CellProfiler software v4.2.6 (Stirling et al, 2021), and the data obtained were processed and plotted with Excel software (Microsoft).

For cell morphology, the shape descriptor “roundness” in ImageJ was used after manually drawing around the cell shape using F-actin-staining images (Orgaz et al, 2020). Phospho-MLC2 fluorescence signal was quantified by calculating the pixel intensity in single cells relative to the cell area (Orgaz et al, 2020).

### 2D migration and invasion experiments

After 6 h of 1% serum-starvation, a serum-free cell suspension (40,000/100,000) SK-MEL-147 cells were seeded in duplicate into Boyden-chamber wells 6.5-mm Transwell filters with 8-µm pores (#3422, Costar or Biocoat Matrigel Invasion Chamber #354480, Corning) and exposed to medium containing complete medium in the presence or absence of THX-B (20μM). After 24 h of incubation at 37 °C and 5% CO_2_, we fixed the cells with 4% paraformaldehyde (PFA) and stained with DAPI for 15 min (5 µg/ml, D9542, Merck). Baseline migration (toward a starving medium, no chemoattraction) was included as a negative control. Samples were captured with a TCS-SP5 confocal microscope (Leica Microsystems) equipped with a 20X/NA0.7 air objective, AOBS and LAS AF v2.73 software. A z-stack of four representative FOV were captured for each replicate and analyzed with Imaris v7.3 software. The movement of individual SK-MEL-147 cancer cells towards the membrane was analyzed by detecting the nuclei of the cells, localizing the transwell membrane and the positions of each cell in each frame (before or after migration). % of migrated cells was defined by the number of cells crossing the membrane / total number of cells identified.

### Cell culture on thick layers of collagen I

About 5μg/ml of Rat Tail type I collagen (Millipore 08-115) was placed onto 12 mm diameter cover slips for 1 h at 37 °C. After washing with PBS, 40,000–70,000 cells (SK-MEL-147 and A375M2, respectively) were seeded for another 24 h. Cells were fixed in PFA 4% and stained and imaged for Immunofluorescence as indicated before.

Fibrillar bovine dermal collagen (no. 5005-B; PureCol, Advanced BioMatrix) was prepared at 1.7 mg/ml in DMEM; 100 μl/well in 96-well plates; 700 μl/well in 12-well plates. After collagen gel polymerization (4 h), cells were seeded on top of collagen in medium containing 10% FBS, allowed to adhere for 24 h, treatments added (where appropriate), imaged and fixed in PFA 4% and stained and imaged for Immunofluorescence as indicated before.

### Spheroids formation and 3D collagen I invasion assay

Multicellular spheroids of A375P cells were prepared with the hanging droplet method (Kelm et al, 2003), using 3 × 10^3^ cells in a 20 μl droplet in complete DMEM media. After 3 days, spheroids were embedded in Rat Tail type I collagen (Millipore 08-115) diluted to a final concentration of 2.2 mg/ml in 25 mM HEPES and PBS. NaOH was added till pH reached 7.5. After polymerization, spheroids were covered with complete media. When indicated, 1 μM of ROCKi was added to the covered media. Spheroids were fixed in 4% paraformaldehyde immediately after polymerization of the matrix (T0) or after 3 days of invasion (T2). After fixation, cells in spheroids were permeabilized 15 min in 0.1% Triton X-100/PBS and labeled with human anti-NGFR (dil 1:50 Nori 138B, ab271289, Abcam), pMLC2 (anti-human pS19 pMLC2 dil 1:100 #3671 Cell Signaling), and DAPI (5μg/ml, 15 min) as indicated above. Spheroids were imaged with a bright-field microscope or a Thunder Imaging System (Leica Microsystems) equipped with a 5X/NA0.15 air objective and a TCS-SP5 confocal Microscope (Leica Microsystems) equipped with a 10X/NA0.4 air objective, AOBS and LAS AF v2.75 software, collecting a stack of images along the z-axis with a 10–15 μm interval between optical sections. Spheroid area was measured using ImageJ software. T2 (72 h) data were normalized with their corresponding T0, and statistical analyses were performed between the different normalized-T2 conditions. In A375P pLV-mCherry-GFP cells, NGFR-GFP induction was calculated by performing the ratio between the intensity of GFP vs mCherry per cell at the core and distal site (IF) of the spheroids using DefiniensXD v2.5 software. NGFR-GFP/mCherry ratios at the Invasive Front were normalized with the ratios at the core of the spheroids at both T0 and T2. To analyze NGFR and pMLC2 expression at the invasive front of the spheroids, a selection of 5 spheroids per condition were imaged using a TCS-SP5 confocal microscope (Leica Microsystems) equipped with a 40X objective with an AOBS and LAS AF v2.73 software. A z-stack was acquired, and then the maximum intensity projection (MIP) was calculated with Fiji software v1.54 f. Invasive front, measured as the cells detached from the spheroid core, like in solid tumors, was manually defined for each spheroid based on the nuclei, and the mean intensity was calculated for each channel using FIJI. The mean intensity for NFGR and pMLC2 was normalized by DAPI mean intensity.

### RhoA-GTP pulldown assay

RhoA-GTP pulldown assay was performed as previously described (Samain et al, 2023). Briefly, 300,000 cells/well were seeded on six-well plates and serum-starved (1% FBS; 24 h). Cells were lysed in pull-down lysis buffer (50 mM tris (pH 7.4), 1% Triton, 10 mM MgCl_2_, 200 mM NaCl, 1 mM dithiothreitol, 0.1 mM phenylmethylsulphonyl fluoride, and EDTA-free protease inhibitor cocktail); sonicated (15 s twice in a water sonication bath) and centrifuged at 13,000 rpm for 25 min). 30μl of the supernatant was separated for the determination of total RhoA levels. The remaining protein lysate was incubated for 1 h at 4 °C with 18 μl of glutathione *S*-transferase–conjugated Rhotekin RBD beads (Cytoskeleton, #RT02). Beads were centrifuged (4 min at 700 × *g*), washed and resuspended in loading buffer. All samples were boiled for 5 min and resolved by SDS–polyacrylamide gel electrophoresis. RhoA levels were detected by immunoblot (1:1000; Cell Signaling Technology, #2117).

### Statistical analysis

The error bars in the graphical data represent the means + SEM. For in vivo and in vitro assays, specifics regarding biological replicates are stated in the figure legends. When appropriate, the statistical significance was determined by applying the two-tailed unpaired Student’s *t*-test or one-way/two-way ANOVA with appropriate correction for multiple comparison (Dunnett, Tukey, Bonferroni) using GraphPad Prism software version 5.03. The presence of NGFR-positive cells on non-invasive vs invasive 3D A375P spheroids and the Melanoma patients with high NGFR expression (Q1) vs Q2.4 in responders (R) and non-responders (NR) patients were analyzed using a chi-square Fisher exact test.

### Ethical compliance

This research complies with all relevant ethical regulations. Human samples were collected with informed written consent, in accordance with the Helsinki Declaration, and the study design was approved by the Guy’s Research Ethics Committee and Ethics Committee of Guy’s and St Thomas’ NHS Foundation Trust and the Ethics Committee of the IRBLleida (PT17/0015/0027) and HUB-ICO-IDIBELL (PT17/0015/0024) Biobanks, in accordance with the Human Tissue Act, 2004. Compensation was not provided. Human cell experiments from biopsies of metastatic melanomas were approved by the ethical review board (BASEC-2017–0494, KEK No. 2014–0425) at the University Hospital Zurich as previously described in (Raaijmakers et al, 2015).

All animals were housed according to institutional guidelines, and all procedures were approved by the ISCIII Ethical Committee and the Comunidad de Madrid (PROEX225/17 and PROEX82.7/23) in Spain, the Lady Davis Institute Animal Care Committee (Canada) and/or the Ethical Review Process Committees at Barts Cancer Institute, King’s College London and The Francis Crick Institute. The experiments were performed in accordance with the guidelines for Ethical Conduct in the Care and Use of Animals as stated in The International Guiding Principles for Biomedical Research involving Animals, developed by the Council for International Organizations of Medical Sciences (CIOMS), and the guidelines of the Canadian Council on Animal Care. All animals were maintained under specific pathogen-free conditions and/or the Institutional Committees on Animal Welfare of the UK Home Office (The Home Office Animals Scientific Procedures Act, 1986) and the Committee of the National Cancer Research Institute.

## Supplementary information

Table EV1

Table EV2

Peer Review File

Source data Fig. 1

Source data Fig. 2

Source data Fig. 3

Source data Fig. 4

Source data Fig. 5

Source data Fig. 6

Source data Fig. 7

Figure EV1 Source Data

Figure EV2 Source Data

Figure EV3 Source Data

Figure EV4 Source Data

Figure EV5 Source Data

Expanded View Figures

## Data Availability

No novel large-scale data amenable to data repository deposition were generated in this study. Public transcriptomic analyses: Bulk RNA-seq expression data from GSE78220 and GSE91061 (Hugo et al, 2016; Riaz et al, 2017) were downloaded from the processed expression matrix that is deposited. NGFR expression values (FPKM) were extracted for each of the samples from the cohort, restricting to baseline and pretreatment biopsies and correlated with clinical responses (partial and complete, PR + CR) or non-response (permanent or stable disease, PD + SD). Fisher’s exact chi-squared test has been applied. The ameboid signature comprised 20 genes (RHOA, ROCK1, ROCK2, MYL9, MYL12A, MYL12B, MYH9, MYH10, ACTA2, TAGLN, ITGA5, ITGB1, VCL, PXN, TLN1, FLNA, FLNB, CFL1, MYH11, and MYL6), that were selected because they are well-established components of actomyosin contractility or cytoskeletal remodeling that associate with ameboid motility. Expression values were log-transformed (log1p), and an ameboid score was computed per patient as the mean of gene-wise z-scores for the signature genes. Associations between NGFR and the ameboid score were quantified using Spearman’s rank correlation (*R*) with corresponding *p* values. Results were visualized using scatter plots with a fitted linear trend line and annotation of *n*, *R*, and *p* value. The source data of this paper are collected in the following database record: biostudies:S-SCDT-10_1038-S44318-026-00803-2.
