# Supplementary material for: NGFR induces melanoma invasion and immunotherapy resistance through myosin light chain 2 modulation
Source: EMBO J. 2026 May 26;45(14):4988–5023. doi: 10.1038/s44318-026-00803-2 (PMC13373201; doi:10.1038/s44318-026-00803-2)
Supplement: Supplementary file 1 — Table EV1 [file 44318_2026_803_MOESM1_ESM.pdf]

Table EV1. Clinical information for melanoma patients under IT (TMAs 257 and 258)

	high (N=14)	low (N=18)	Medium (N=13)	Overall (N=45)
**Gender**				
f	4 (28.6%)	7 (38.9%)	7 (53.8%)	18 (40%)
m	10 (71.4%)	11 (61.1%)	6 (46.2%)	27 (60%)
**Age.Range**				
≥60	13 (92.9%)	13 (72.2%)	4 (30.8%)	30(66,7%)
45-60	1 (7.1%)	2 (11.1%)	4 (30.8%)	7 (15,5%)
<45	0 (0%)	1 (5.6%)	1 (7.7%)	2 (4,5%)
45-59	0 (0%)	2 (11.1%)	4 (30.8%)	6 (13,3%)
**Anatomical.location**				
brain	2 (14.3%)	0 (0%)	2 (15.4%)	4 (8,9%)
LN	6 (42.9%)	6 (33.3%)	3 (23.1%)	15(33,3%)
skin	6 (42.9%)	7 (38.9%)	7 (53.8%)	20(44,5%)
lung	0 (0%)	0 (0%)	1 (7,7%)	1 (2,2%)
others	0 (0%)	5 (5.6%)	0 (0%)	5 (11,1%)
**Melanoma.Subtype**				
acral lentiginous	1 (7.1%)	1 (5.6%)	0 (0%)	2 (4.5%)
cutaneous	11 (73.3%)	14 (77.8%)	13 (100%)	38(84.4%)
mucosal	0 (7.1%)	2 (11,1%)	0 (0%)	2 (4.5%)
unknown	2 (14.3%)	1 (5.6%)	0 (0%)	3 (6.6%)
**Stage**				
III	10 (71.4%)	11 (61.1%)	8 (61.5%)	29(64.5%)
IV	4 (28.6%)	6 (33.3%)	5 (38.5%)	15(33.3%)
III or IV	0 (0%)	1 (5.6%)	0 (0%)	1 (2.2%)
**Mutation.status**				
BRAF	8 (57.1%)	12 (66.7%)	8 (61.5%)	28(62.3%)
KRAS	1 (7.1%)	2 (11.1%)	0 (0%)	3 (6.6%)
NRAS	5 (35.7%)	4 (22.2%)	5 (38.5%)	14(31.1%)
**Treatment**				
Nivo	10 (71.4%)	7 (38.9%)	7 (53.8%)	24(53.3%)
Pembro	4 (28.6%)	5 (27.8%)	2 (15.4%)	11(24.5%)
Ipi	0 (0%)	3 (16.7%)	1 (7.7%)	4 (8.9%)
Ipi+Nivo	0 (0%)	2 (11.1%)	2 (15.4%)	4 (8.9%)
Ipi+Nivo or Nivo	0 (0%)	1 (5.6%)	0 (0%)	1 (2.2%)
Nivo+LAG3	0 (0%)	0 (0%)	1 (7.7%)	1 (2.2%)
