# Supplementary material for: NGFR induces melanoma invasion and immunotherapy resistance through myosin light chain 2 modulation
Source: EMBO J. 2026 May 26;45(14):4988–5023. doi: 10.1038/s44318-026-00803-2 (PMC13373201; doi:10.1038/s44318-026-00803-2)
Supplement: Supplementary file 2 — Table EV2 [file 44318_2026_803_MOESM2_ESM.pdf]

Table EV2. Clinical information for melanoma patients (TMAs 18 and 19)

| | High (N=16) | Low (N=15) | medium (N=15) | Overall (N=46) |
|---|---|---|---|---|
| **Gender** | | | | |
| F | 4 (25.0%) | 6 (40.0%) | 6 (40.0%) | 16 (34.8%) |
| M | 12 (75.0%) | 9 (60.0%) | 9 (60.0%) | 30 (65.2%) |
| **Age.Range** | | | | |
| >= 60 | 10 (62.5%) | 12 (80.0%) | 9 (60.0%) | 31 (67.4%) |
| 45 - 60 | 6 (37.5%) | 3 (20.0%) | 4 (26.7%) | 13 (28.3%) |
| < 45 | 0 (0%) | 0 (0%) | 2 (13.3%) | 2 (4.3%) |
| **Melanoma.Subtype** | | | | |
| acral | 1 (6.3%) | 1 (6.7%) | 0 (0%) | 2 (4.3%) |
| nodular | 6 (37.5%) | 8 (53.3%) | 6 (40.0%) | 20 (43.5%) |
| SSM | 6 (37.5%) | 6 (40.0%) | 5 (33.3%) | 17 (37.0%) |
| Missing | 3 (18.8%) | 0 (0%) | 4 (26.7%) | 7 (15.2%) |
| **Stage** | | | | |
| IIIB | 1 (6.3%) | 1 (6.7%) | 1 (6.7%) | 3 (6.5%) |
| IIIC | 3 (18.8%) | 2 (13.3%) | 1 (6.7%) | 6 (13.0%) |
| IV | 1 (6.3%) | 0 (0%) | 0 (0%) | 1 (2.2%) |
| M1c | 1 (6.3%) | 2 (13.3%) | 2 (13.3%) | 5 (10.9%) |
| M1a | 0 (0%) | 0 (0%) | 1 (6.7%) | 1 (2.2%) |
| M1b | 0 (0%) | 0 (0%) | 1 (6.7%) | 1 (2.2%) |
| Missing | 10 (62.5%) | 10 (66.7%) | 9 (60.0%) | 29 (63.0%) |
