# Supplementary figures and images for: NGFR induces melanoma invasion and immunotherapy resistance through myosin light chain 2 modulation (part 1 of 3)
Source: EMBO J. 2026 May 26;45(14):4988–5023. doi: 10.1038/s44318-026-00803-2 (PMC13373201; doi:10.1038/s44318-026-00803-2)

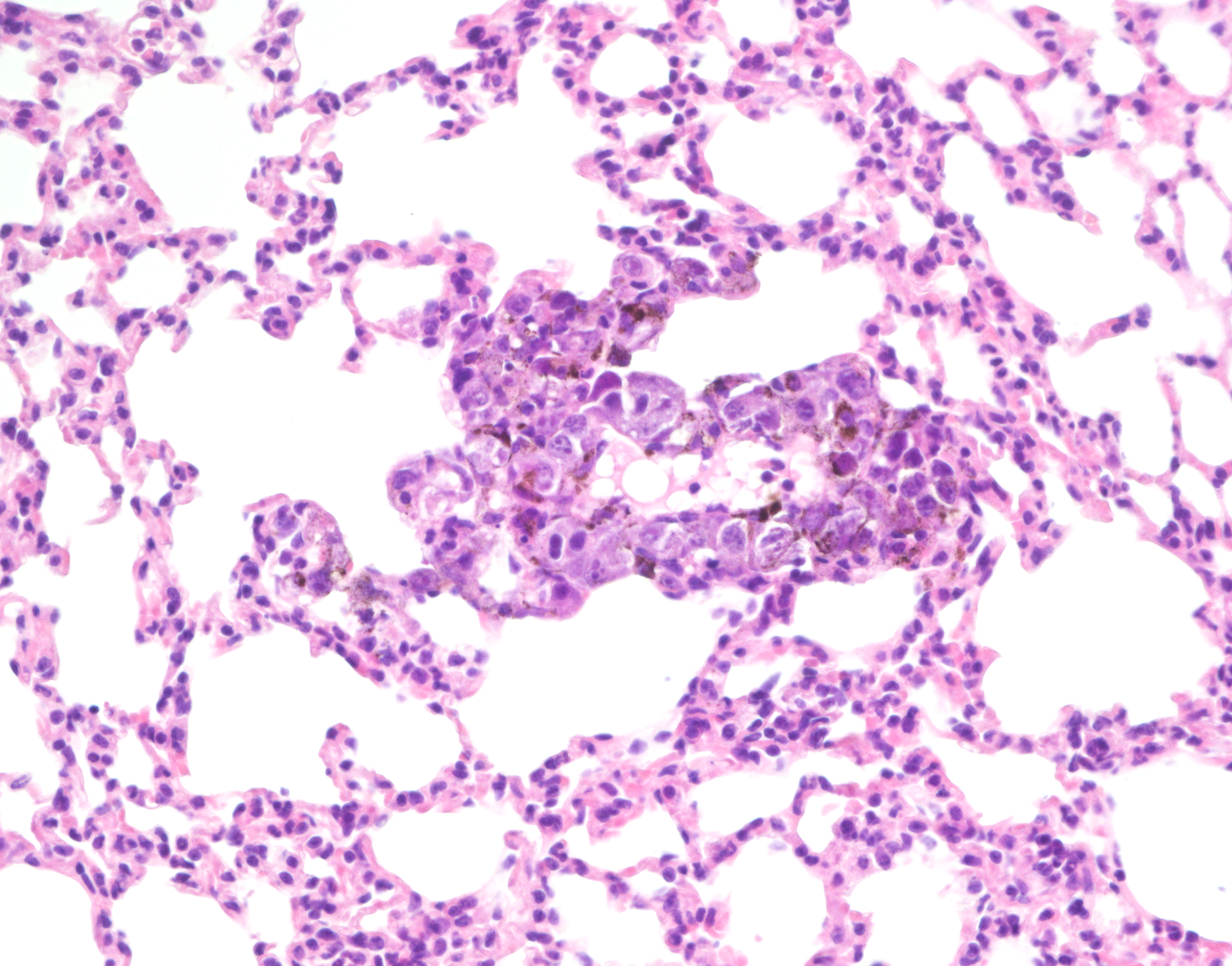

Supplement: Supplementary file 4 — Source data Fig. 1 [file 44318_2026_803_MOESM4_ESM.zip › Fig 1/1C/Copia de gN1 CWS194 LUNG 40X 1.tif]

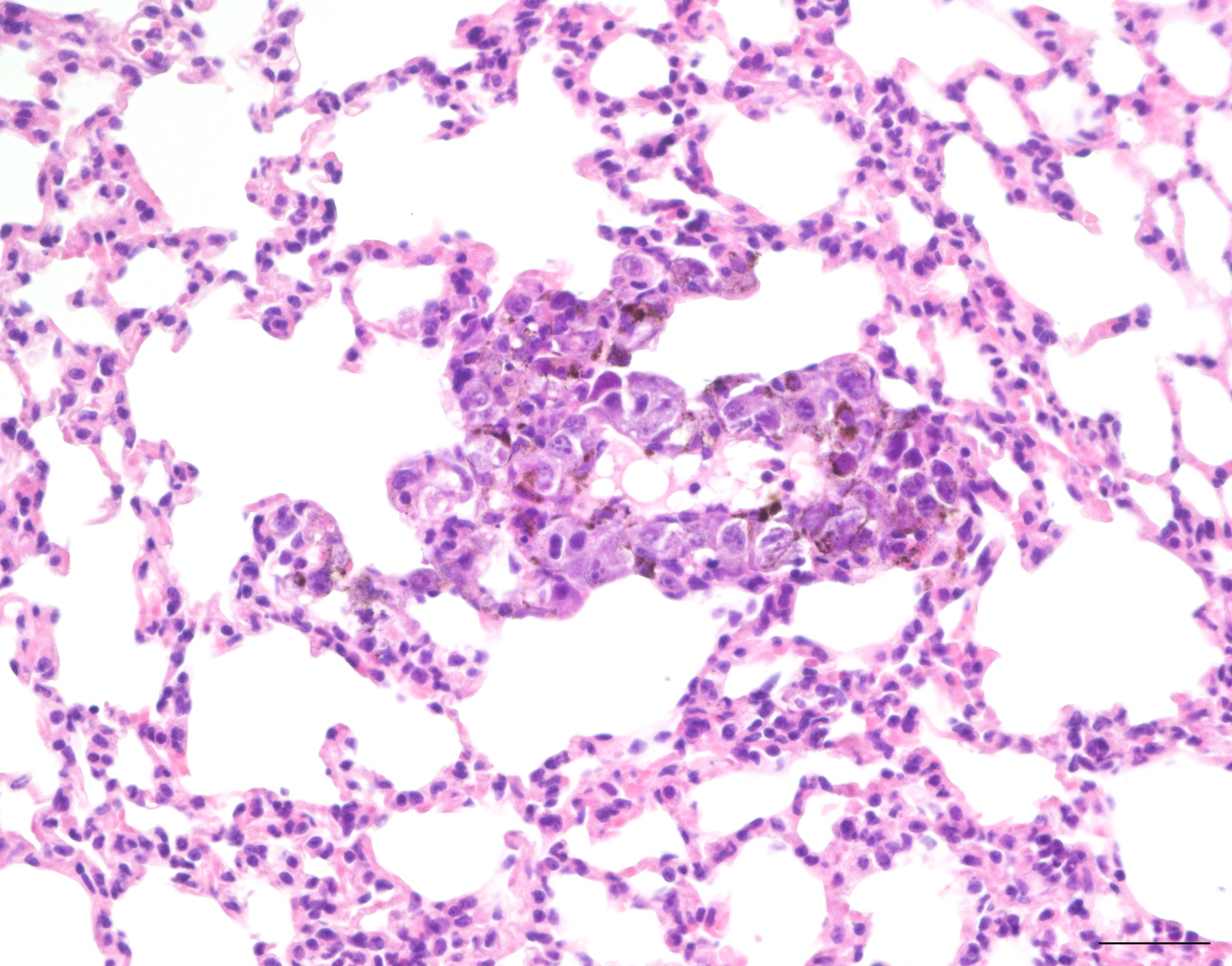

Supplement: Supplementary file 4 — Source data Fig. 1 [file 44318_2026_803_MOESM4_ESM.zip › Fig 1/1C/Copia de gN1 CWS194 LUNG 40X 1_scala.jpg]

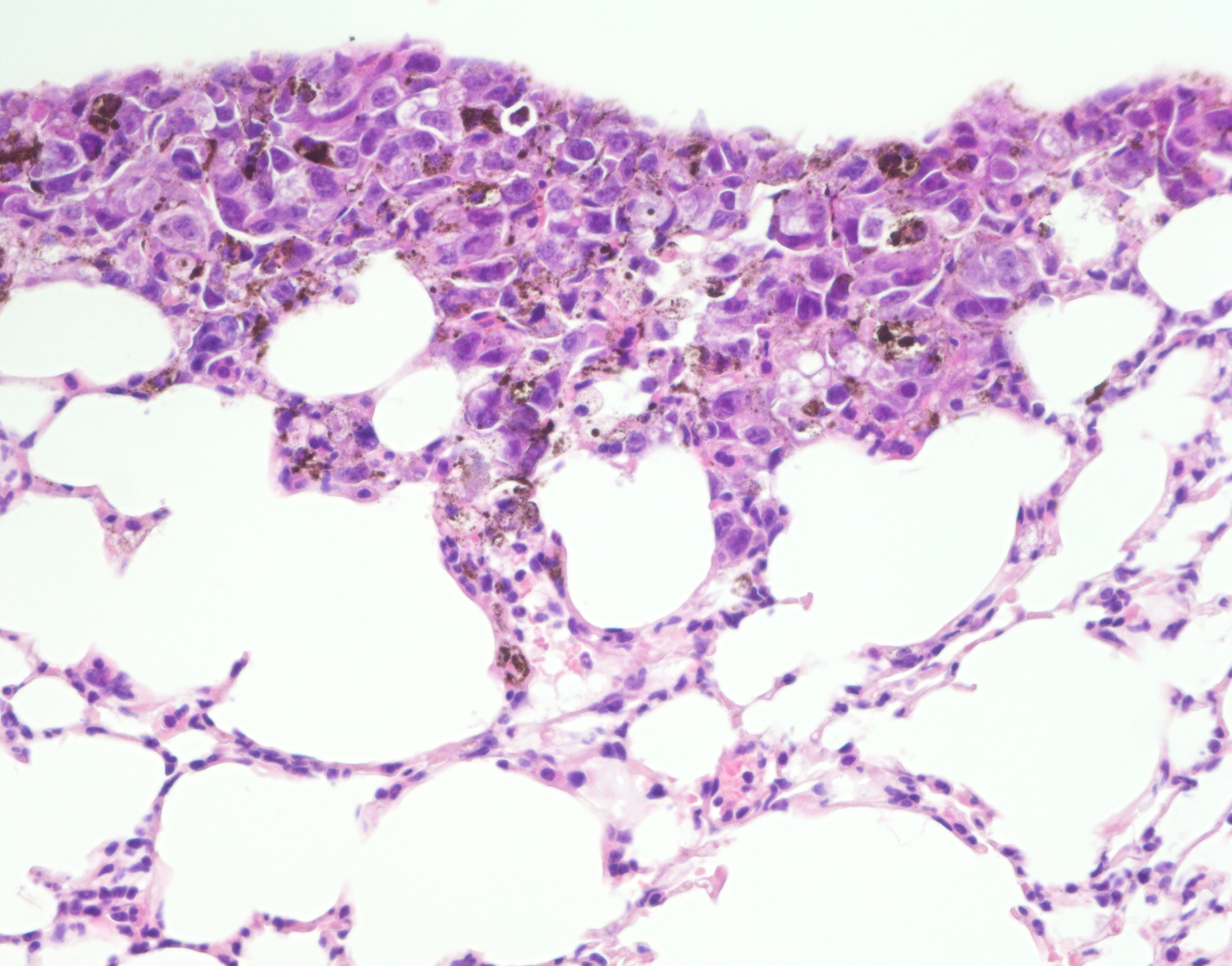

Supplement: Supplementary file 4 — Source data Fig. 1 [file 44318_2026_803_MOESM4_ESM.zip › Fig 1/1C/Copia de WT CWS208 LUNG 40X 1.tif]

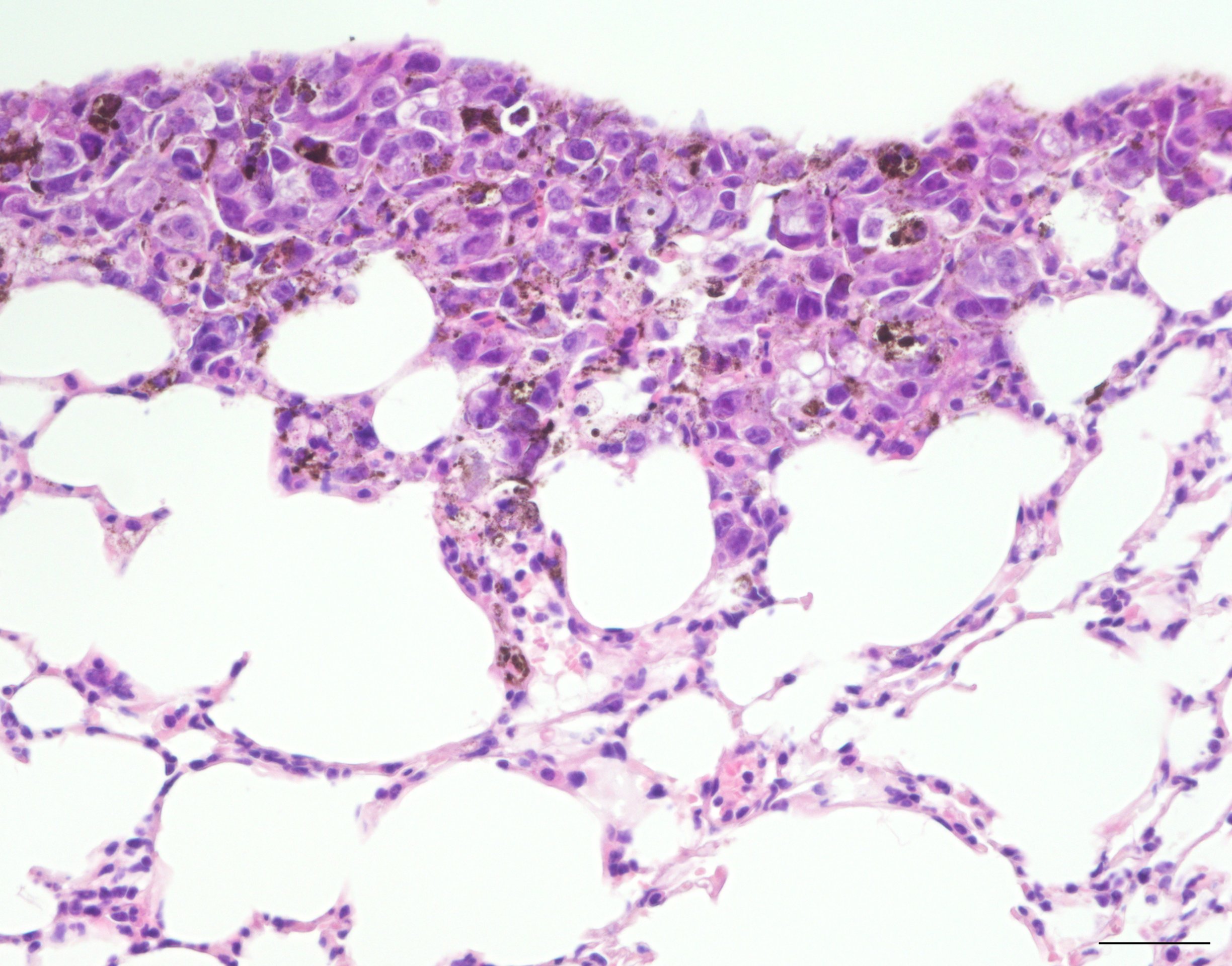

Supplement: Supplementary file 4 — Source data Fig. 1 [file 44318_2026_803_MOESM4_ESM.zip › Fig 1/1C/Copia de WT CWS208 LUNG 40X 1_escala.jpg]

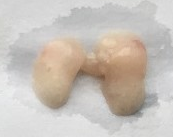

Supplement: Supplementary file 4 — Source data Fig. 1 [file 44318_2026_803_MOESM4_ESM.zip › Fig 1/1F/F1-GFP.tif]

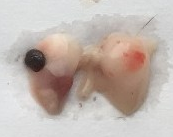

Supplement: Supplementary file 4 — Source data Fig. 1 [file 44318_2026_803_MOESM4_ESM.zip › Fig 1/1F/F1-NGFR-GFP.tif]

SK-MEL-147 GFP luc

cpC

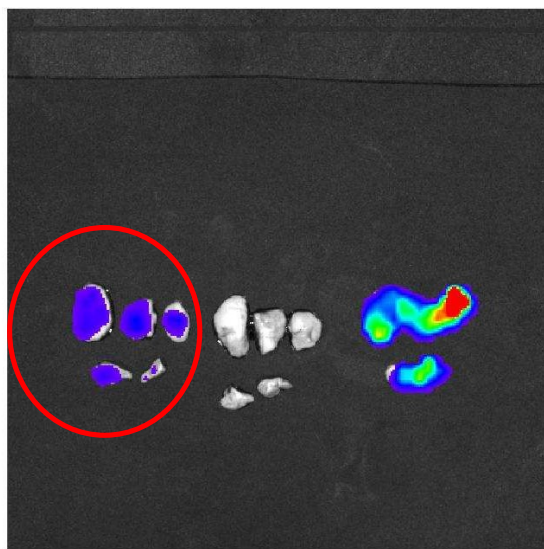

cpNGFR1

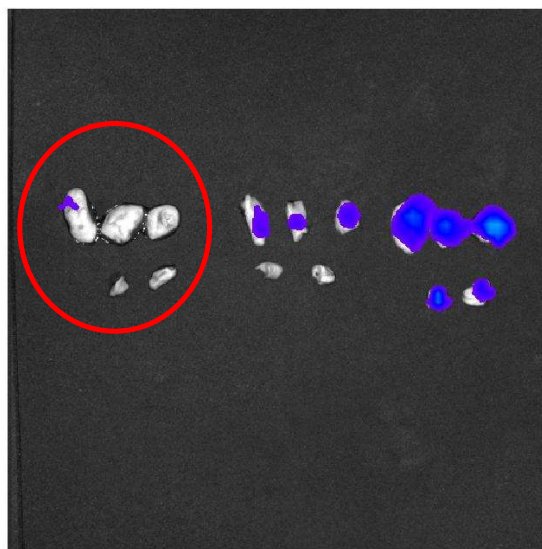

cpNGFR2

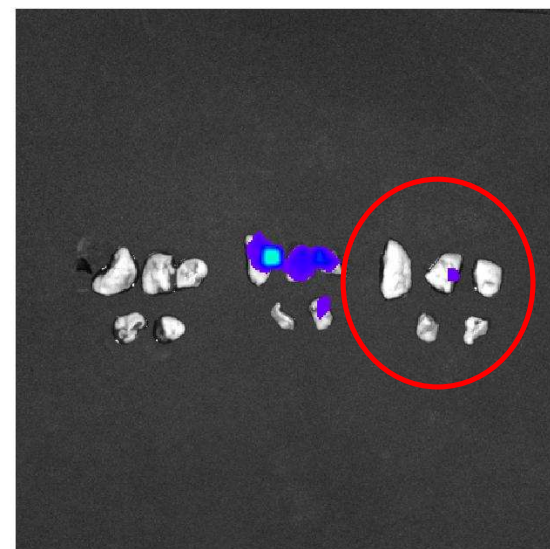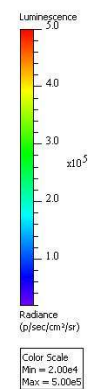

Supplement: Supplementary file 4 — Source data Fig. 1 [file 44318_2026_803_MOESM4_ESM.zip › Fig 1/1I/1I-Readme.pdf]

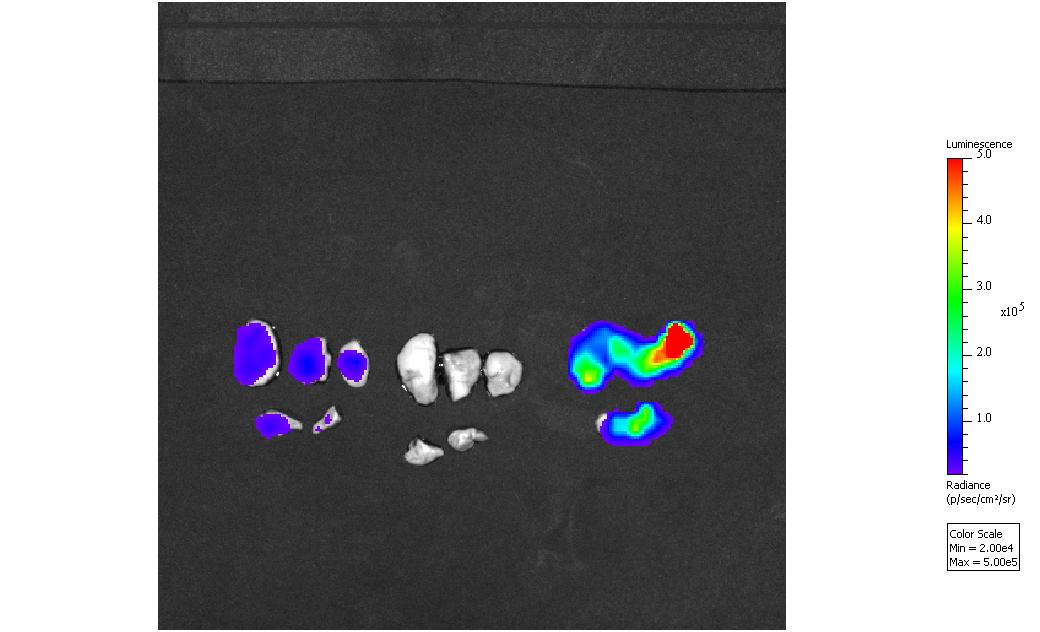

Supplement: Supplementary file 4 — Source data Fig. 1 [file 44318_2026_803_MOESM4_ESM.zip › Fig 1/1I/Control 39-40-31-2.jpg]

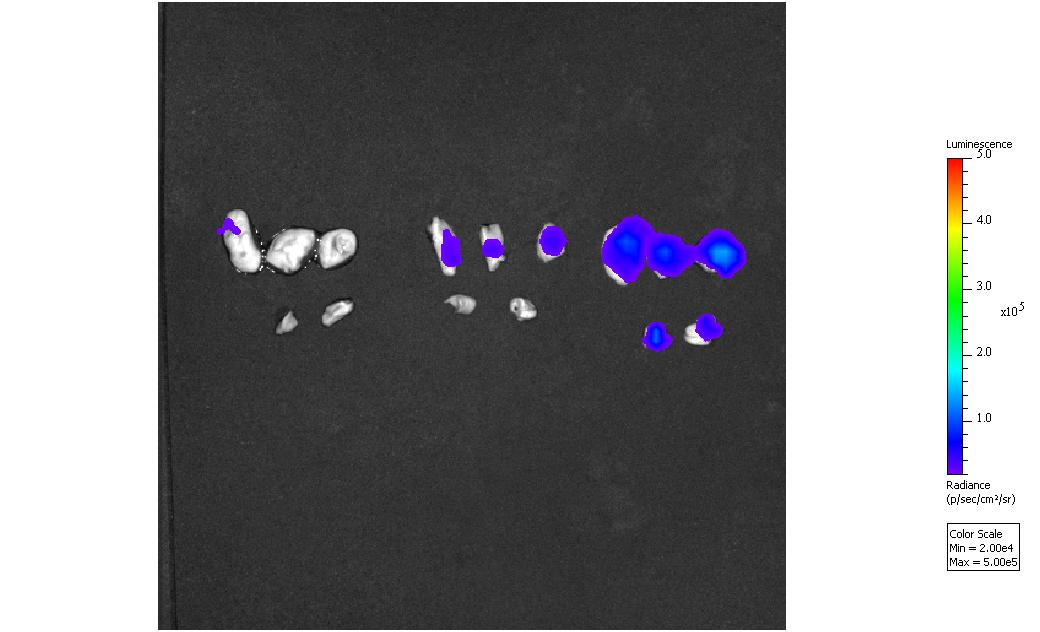

Supplement: Supplementary file 4 — Source data Fig. 1 [file 44318_2026_803_MOESM4_ESM.zip › Fig 1/1I/CP NGFR1 44-32-33-2.jpg]

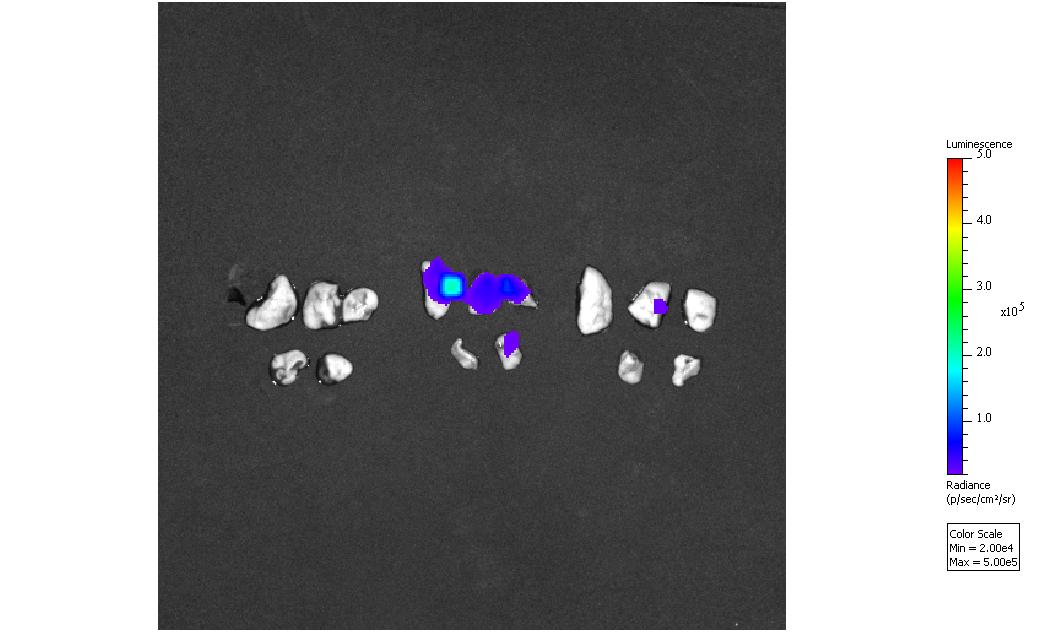

Supplement: Supplementary file 4 — Source data Fig. 1 [file 44318_2026_803_MOESM4_ESM.zip › Fig 1/1I/CP NGFR2 45-46-47-2.jpg]

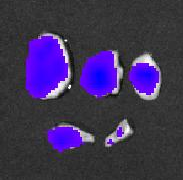

Supplement: Supplementary file 4 — Source data Fig. 1 [file 44318_2026_803_MOESM4_ESM.zip › Fig 1/1I/cpControl.tif]

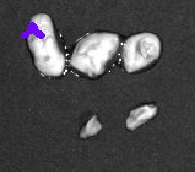

Supplement: Supplementary file 4 — Source data Fig. 1 [file 44318_2026_803_MOESM4_ESM.zip › Fig 1/1I/cpNGFR1.tif]

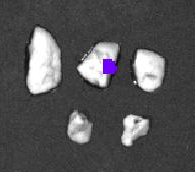

Supplement: Supplementary file 4 — Source data Fig. 1 [file 44318_2026_803_MOESM4_ESM.zip › Fig 1/1I/cpNGFR2.tif]

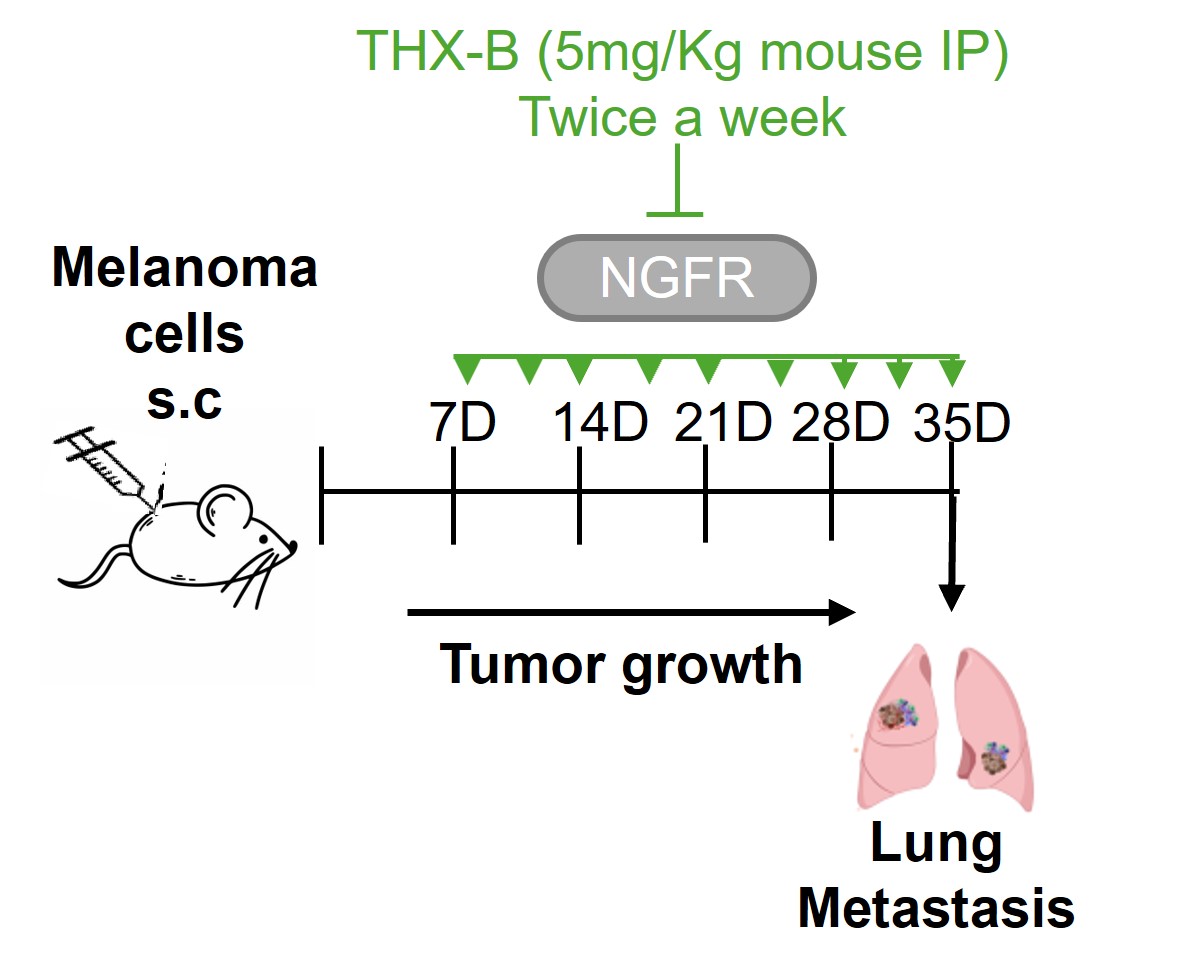

Supplement: Supplementary file 4 — Source data Fig. 1 [file 44318_2026_803_MOESM4_ESM.zip › Fig 1/1K.jpg]

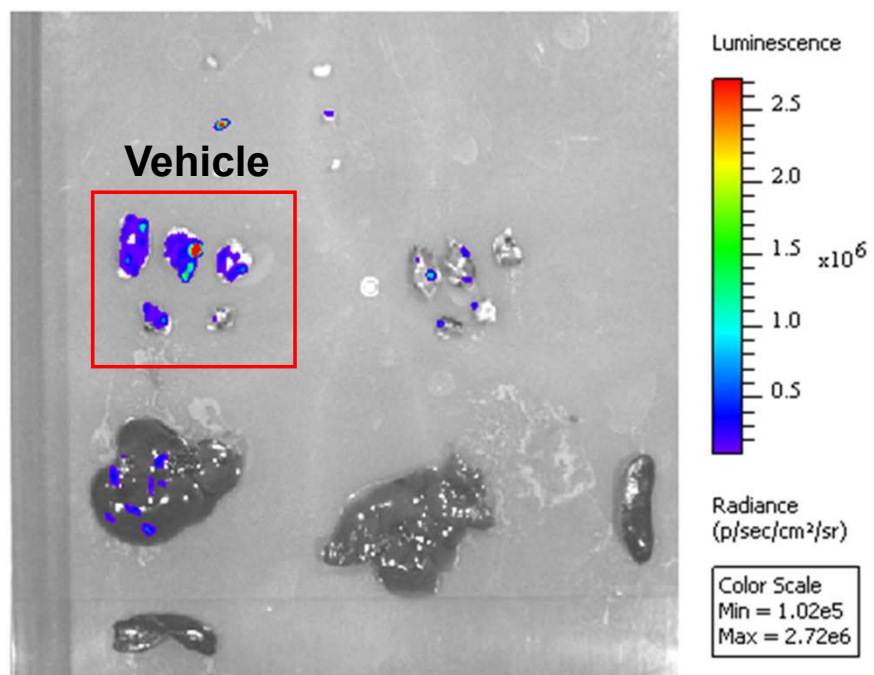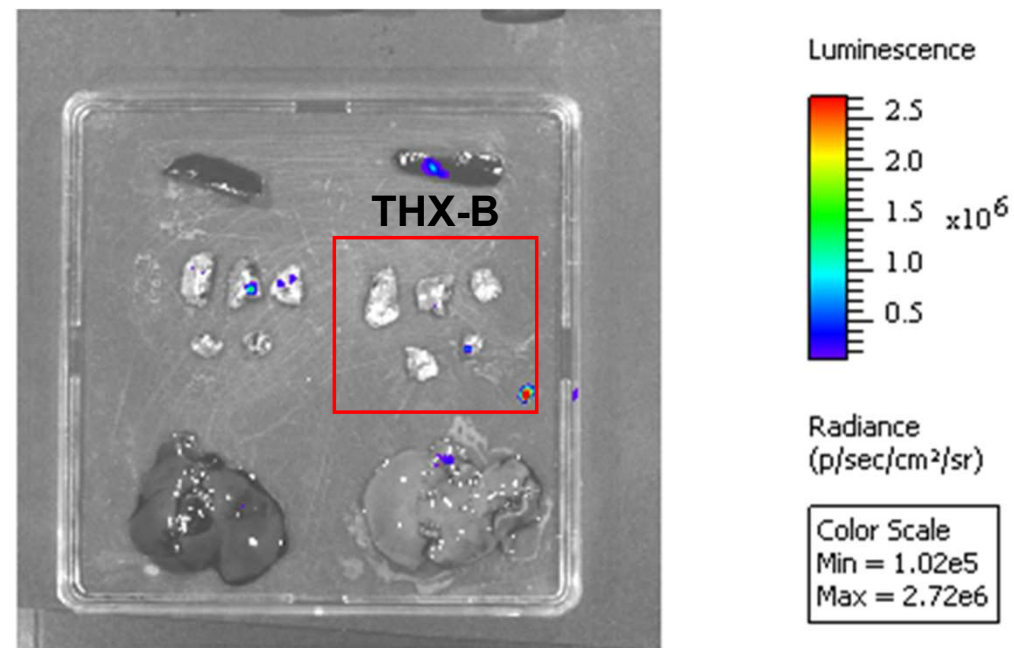

Supplement: Supplementary file 4 — Source data Fig. 1 [file 44318_2026_803_MOESM4_ESM.zip › Fig 1/1N/1N-Readme.pdf]

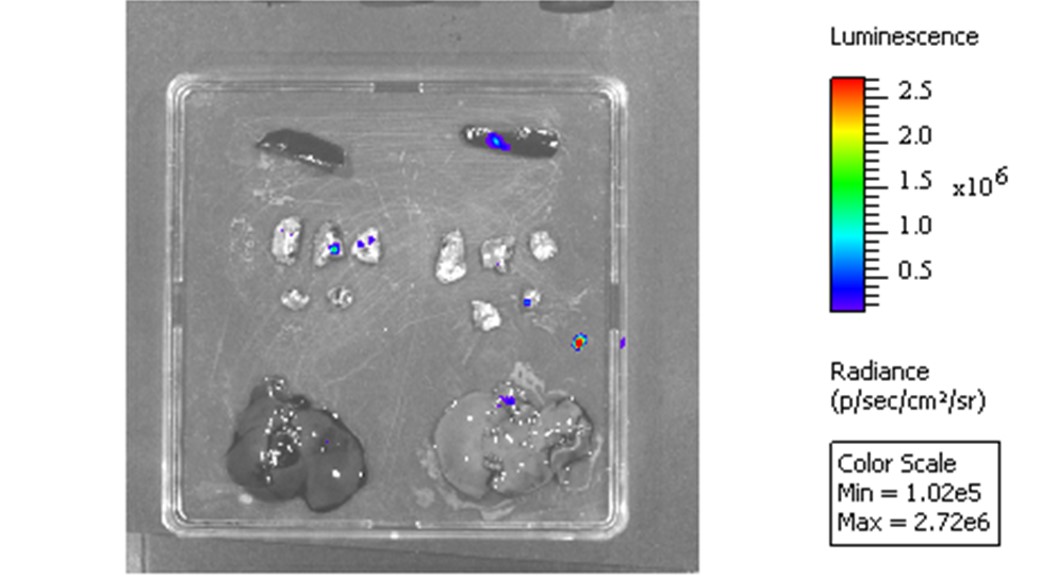

Supplement: Supplementary file 4 — Source data Fig. 1 [file 44318_2026_803_MOESM4_ESM.zip › Fig 1/1N/THX-B.jpg]

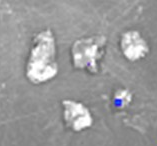

Supplement: Supplementary file 4 — Source data Fig. 1 [file 44318_2026_803_MOESM4_ESM.zip › Fig 1/1N/THX-B.tif]

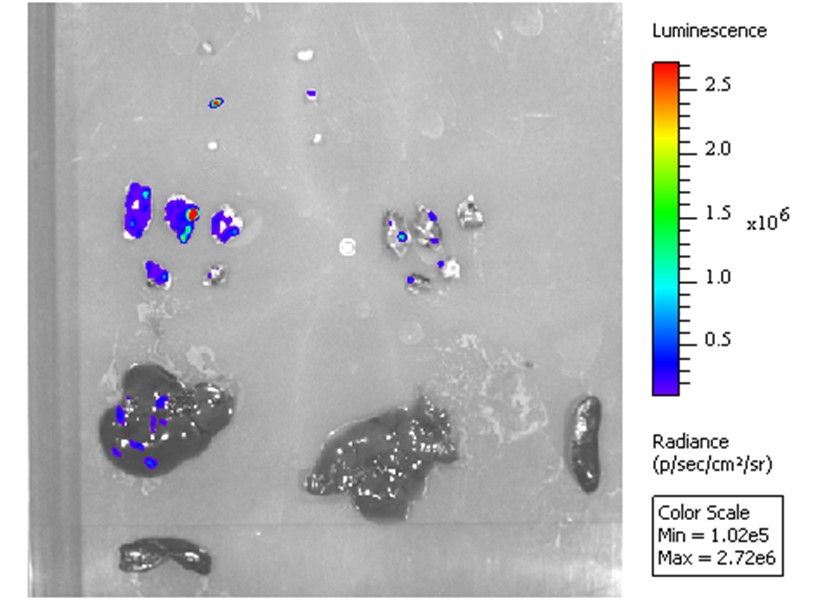

Supplement: Supplementary file 4 — Source data Fig. 1 [file 44318_2026_803_MOESM4_ESM.zip › Fig 1/1N/Vehicle + THX-B.jpg]

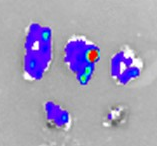

Supplement: Supplementary file 4 — Source data Fig. 1 [file 44318_2026_803_MOESM4_ESM.zip › Fig 1/1N/Vehicle.tif]

Vehicle

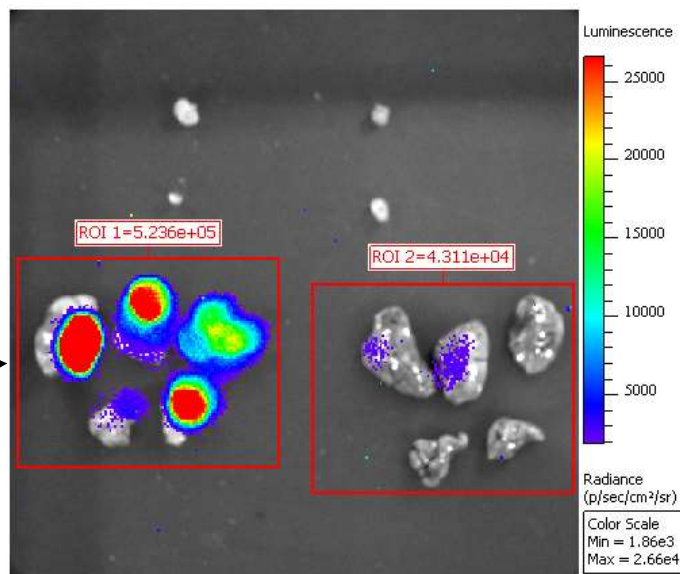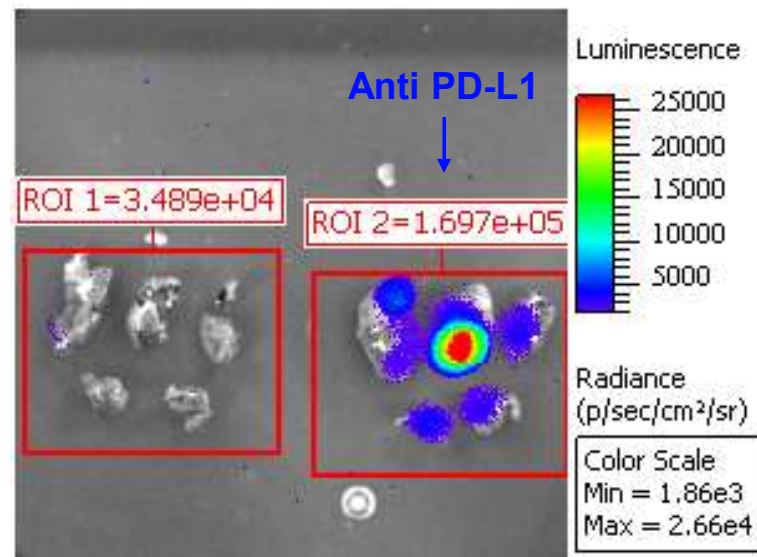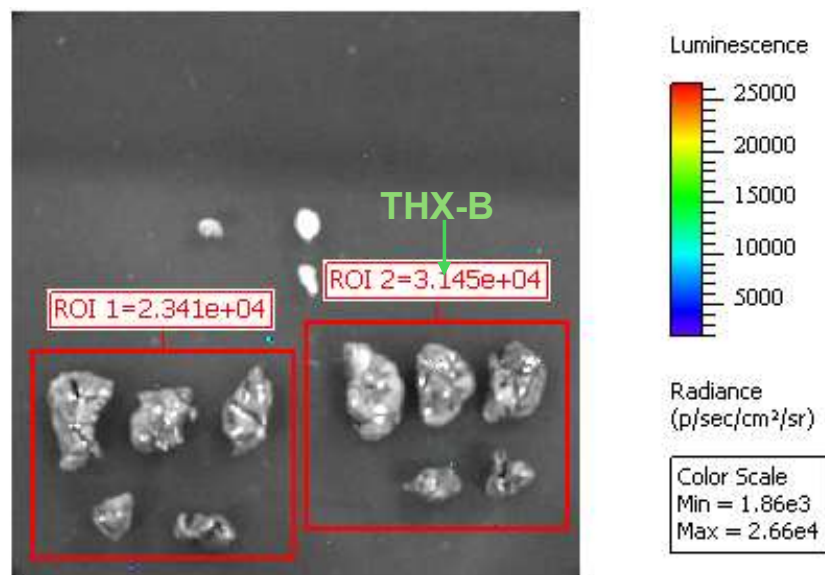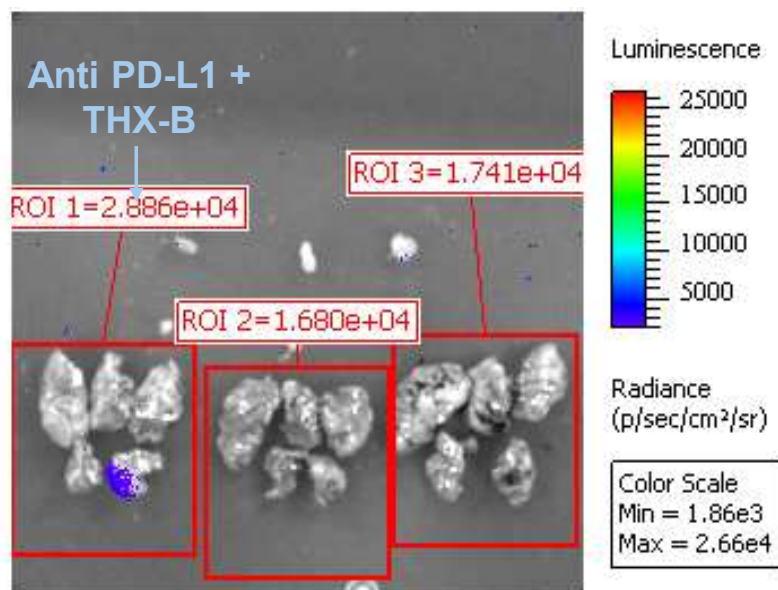

Supplement: Supplementary file 5 — Source data Fig. 2 [file 44318_2026_803_MOESM5_ESM.zip › Fig 2/2C/2C-Readme.pdf]

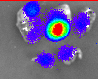

Supplement: Supplementary file 5 — Source data Fig. 2 [file 44318_2026_803_MOESM5_ESM.zip › Fig 2/2C/anti - PD-L1-1.tif]

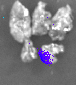

Supplement: Supplementary file 5 — Source data Fig. 2 [file 44318_2026_803_MOESM5_ESM.zip › Fig 2/2C/Combo-1.tif]

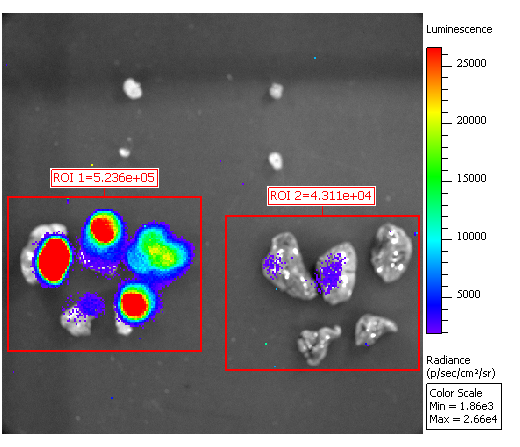

Supplement: Supplementary file 5 — Source data Fig. 2 [file 44318_2026_803_MOESM5_ESM.zip › Fig 2/2C/lungs_group1_1049-1050.tif]

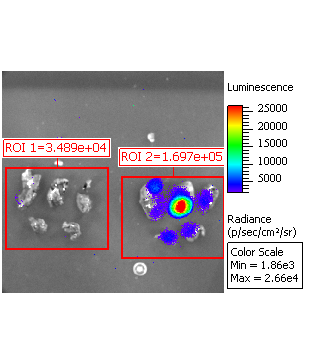

Supplement: Supplementary file 5 — Source data Fig. 2 [file 44318_2026_803_MOESM5_ESM.zip › Fig 2/2C/lungs_group2_1058-1059.tif]

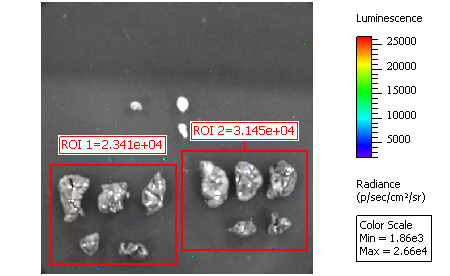

Supplement: Supplementary file 5 — Source data Fig. 2 [file 44318_2026_803_MOESM5_ESM.zip › Fig 2/2C/lungs_group3_1064-1067.tif]

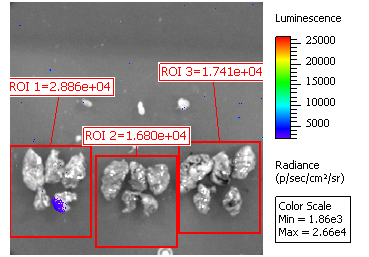

Supplement: Supplementary file 5 — Source data Fig. 2 [file 44318_2026_803_MOESM5_ESM.zip › Fig 2/2C/lungs_group4_1071-1072.tif]

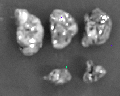

Supplement: Supplementary file 5 — Source data Fig. 2 [file 44318_2026_803_MOESM5_ESM.zip › Fig 2/2C/THX-B-1.tif]

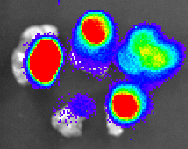

Supplement: Supplementary file 5 — Source data Fig. 2 [file 44318_2026_803_MOESM5_ESM.zip › Fig 2/2C/Vehicle-1.tif]

2g)

### B16-F10-R

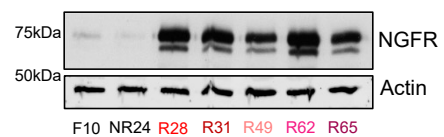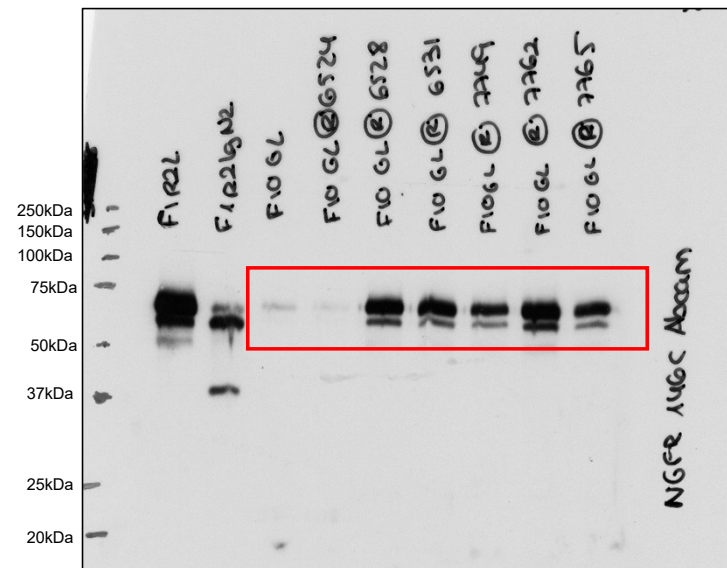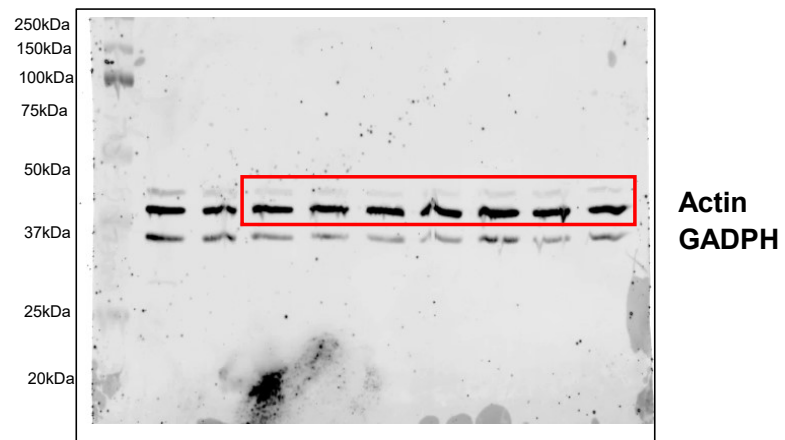

Supplement: Supplementary file 5 — Source data Fig. 2 [file 44318_2026_803_MOESM5_ESM.zip › Fig 2/2G/2G-Readme.pdf]

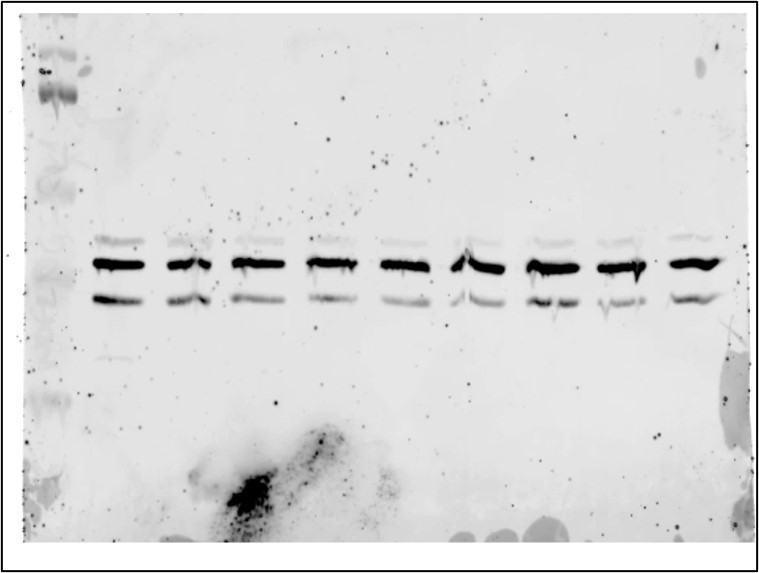

Supplement: Supplementary file 5 — Source data Fig. 2 [file 44318_2026_803_MOESM5_ESM.zip › Fig 2/2G/Actin.tif]

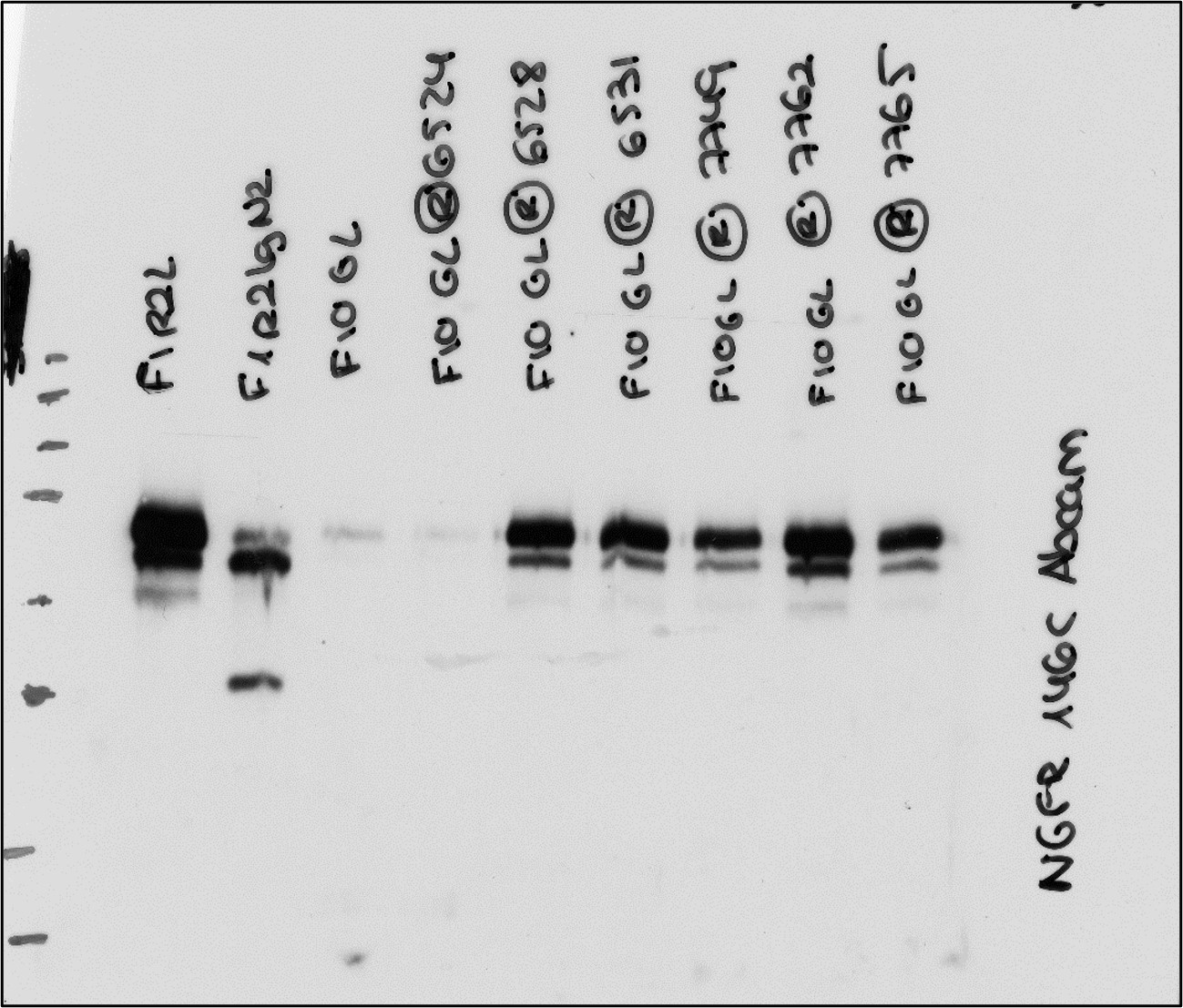

Supplement: Supplementary file 5 — Source data Fig. 2 [file 44318_2026_803_MOESM5_ESM.zip › Fig 2/2G/NGFR.tif]

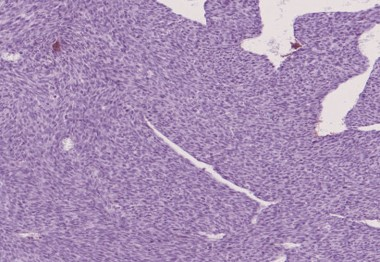

Supplement: Supplementary file 5 — Source data Fig. 2 [file 44318_2026_803_MOESM5_ESM.zip › Fig 2/2H/5555-Anti PD-L1.tif]

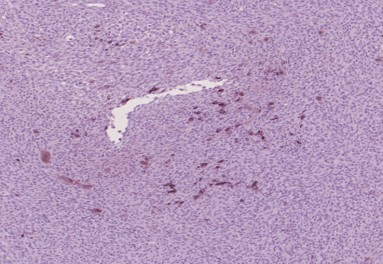

Supplement: Supplementary file 5 — Source data Fig. 2 [file 44318_2026_803_MOESM5_ESM.zip › Fig 2/2H/5555-Control.tif]

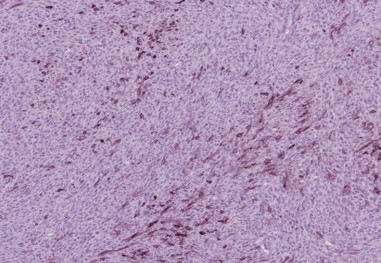

Supplement: Supplementary file 5 — Source data Fig. 2 [file 44318_2026_803_MOESM5_ESM.zip › Fig 2/2H/5555-PD1R-anti PD-1.tif]

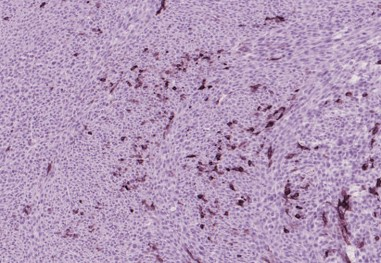

Supplement: Supplementary file 5 — Source data Fig. 2 [file 44318_2026_803_MOESM5_ESM.zip › Fig 2/2H/5555-PD1R-Vehicle.tif]

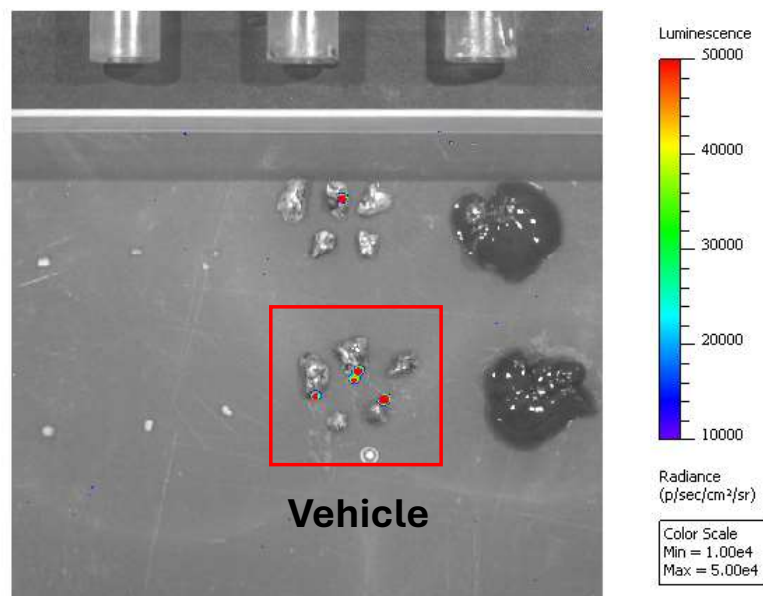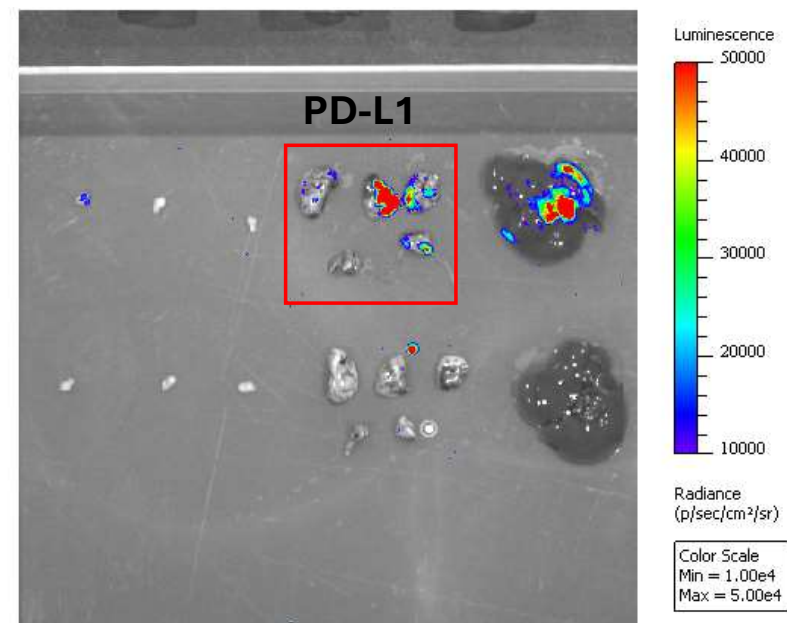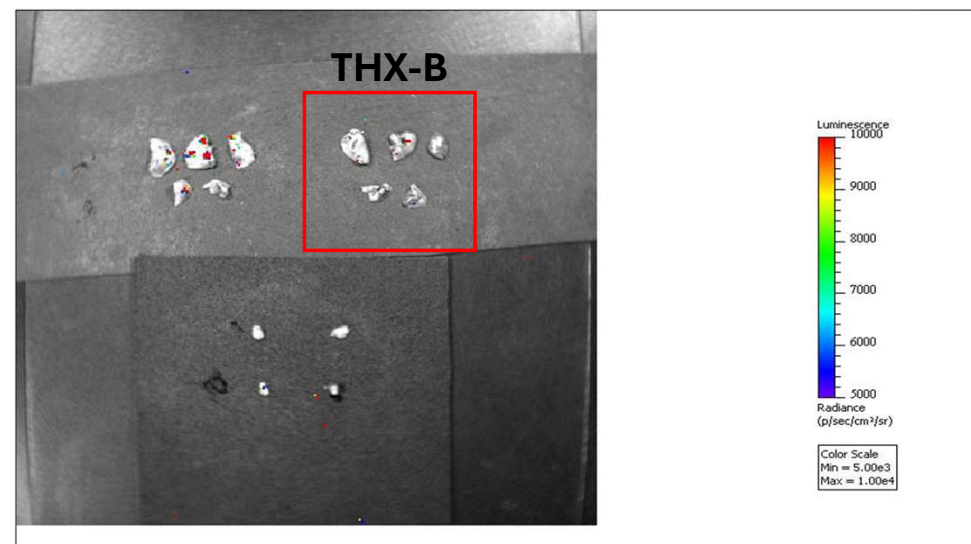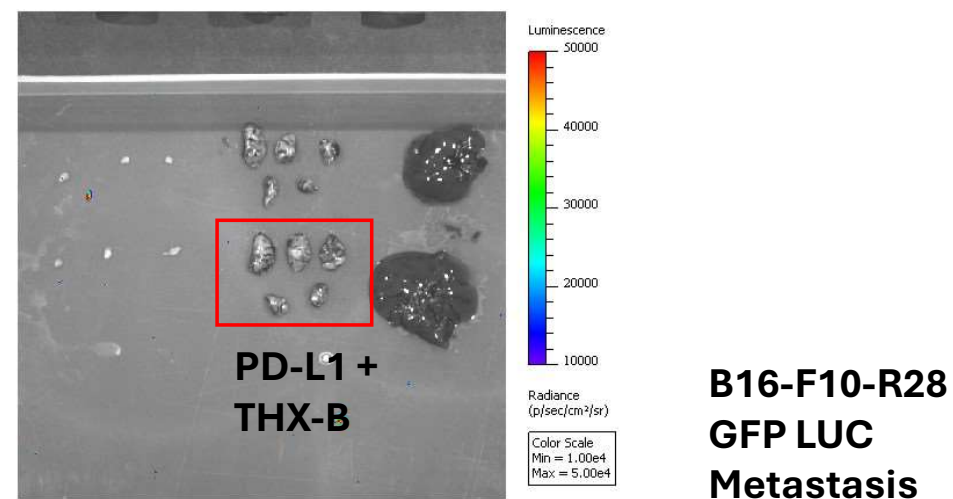

Supplement: Supplementary file 5 — Source data Fig. 2 [file 44318_2026_803_MOESM5_ESM.zip › Fig 2/2L/2L-Readme.pdf]

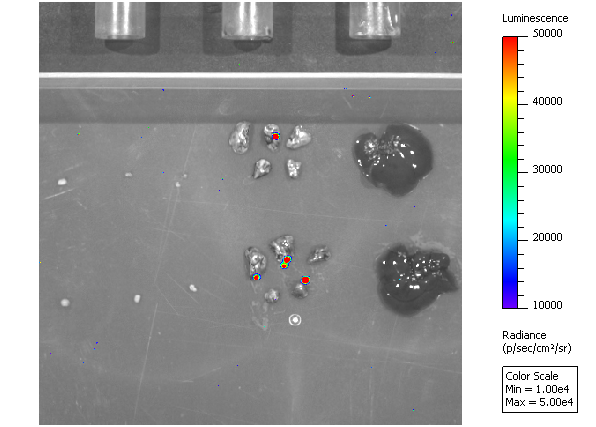

Supplement: Supplementary file 5 — Source data Fig. 2 [file 44318_2026_803_MOESM5_ESM.zip › Fig 2/2L/F10 gfp luc RESISTANT Control XBS 7761-63.tif]

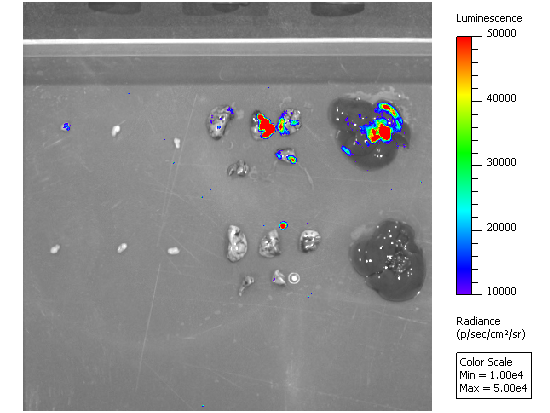

Supplement: Supplementary file 5 — Source data Fig. 2 [file 44318_2026_803_MOESM5_ESM.zip › Fig 2/2L/F10 gfp luc RESISTANTxbs6528 200pdl1 XBS 7757-59.tif]

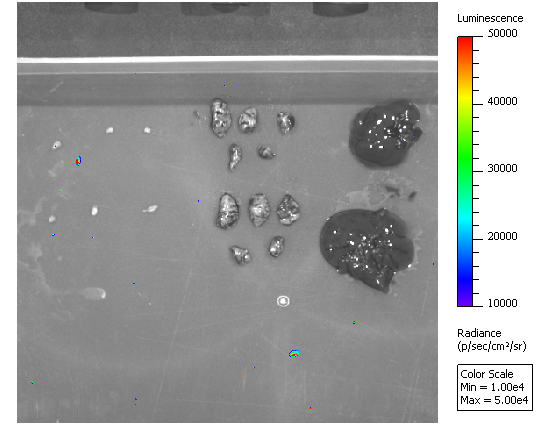

Supplement: Supplementary file 5 — Source data Fig. 2 [file 44318_2026_803_MOESM5_ESM.zip › Fig 2/2L/F10 gfp luc RESISTANTxbs6528 200pdl1+thxb XBS 7767-69.tif]

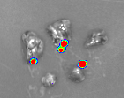

Supplement: Supplementary file 5 — Source data Fig. 2 [file 44318_2026_803_MOESM5_ESM.zip › Fig 2/2L/F10R28 Vehicle-1.tif]

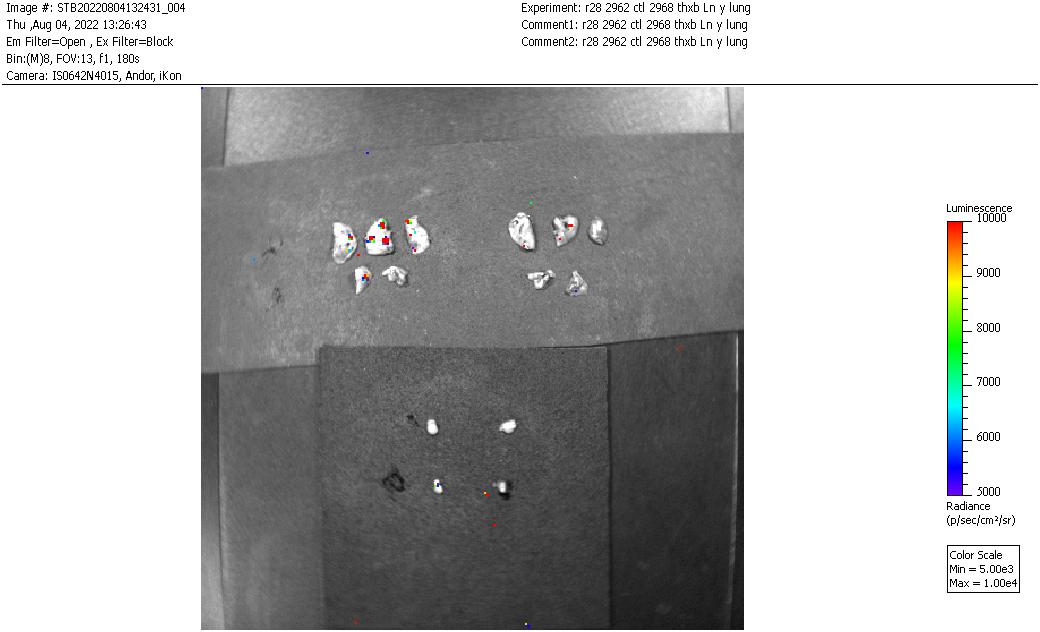

Supplement: Supplementary file 5 — Source data Fig. 2 [file 44318_2026_803_MOESM5_ESM.zip › Fig 2/2L/R28 2962control 2968 thx-b ln.jpg]

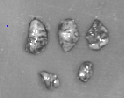

Supplement: Supplementary file 5 — Source data Fig. 2 [file 44318_2026_803_MOESM5_ESM.zip › Fig 2/2L/R28 Combo-1.tif]

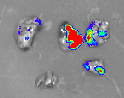

Supplement: Supplementary file 5 — Source data Fig. 2 [file 44318_2026_803_MOESM5_ESM.zip › Fig 2/2L/R28 PDL-1-1.gif]

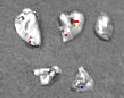

Supplement: Supplementary file 5 — Source data Fig. 2 [file 44318_2026_803_MOESM5_ESM.zip › Fig 2/2L/R28 THX-B-1.tif]

# *CD8+ IHC Yumm1.1 tumors*

IgG (22006210)

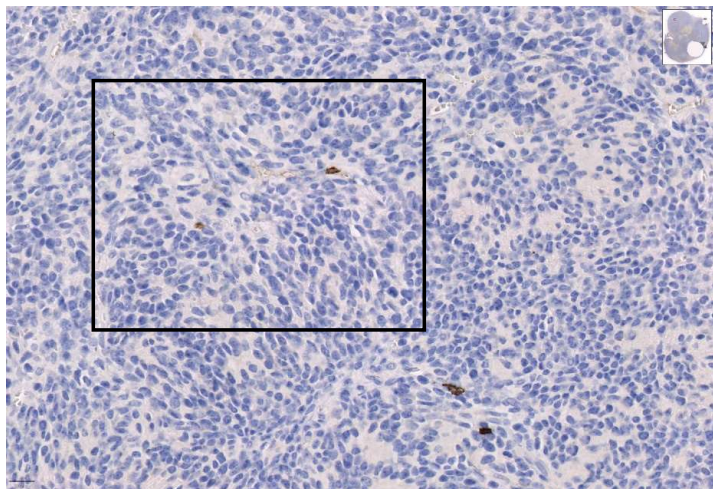

THX-B (22006221)

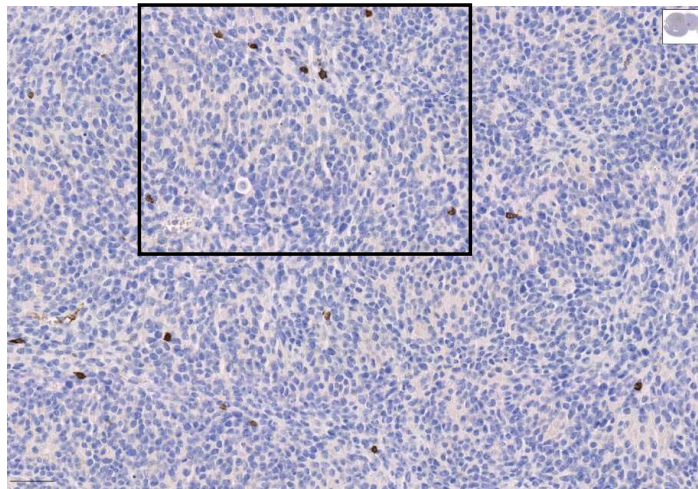

PD-L1 (22006214)

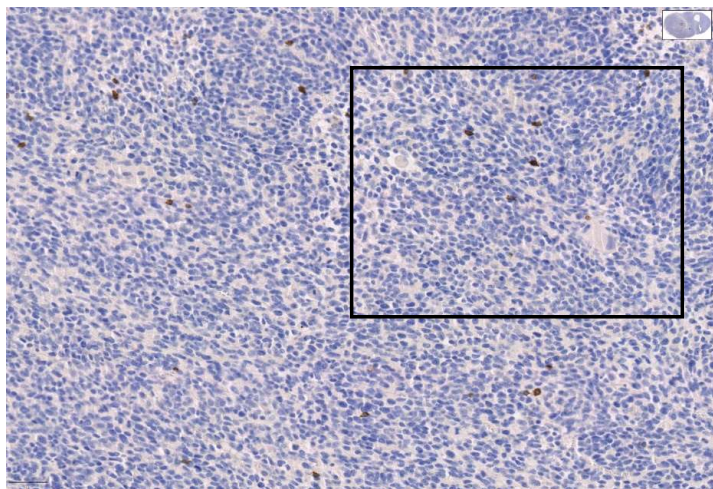

Combo (22006225)

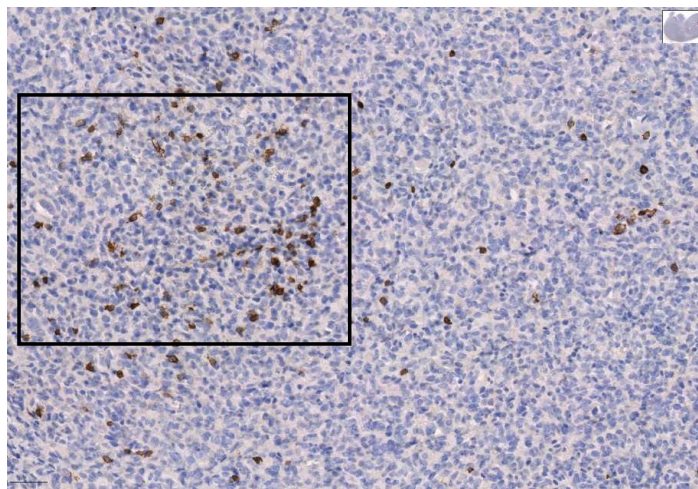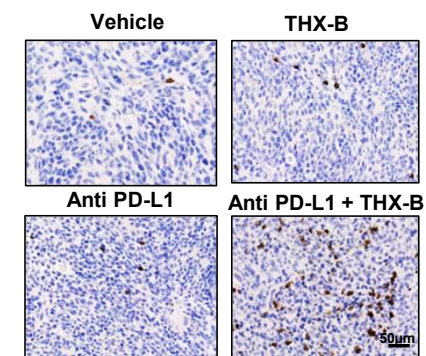

Supplement: Supplementary file 6 — Source data Fig. 3 [file 44318_2026_803_MOESM6_ESM.zip › Fig 3/3B/3B-Readme.pdf]

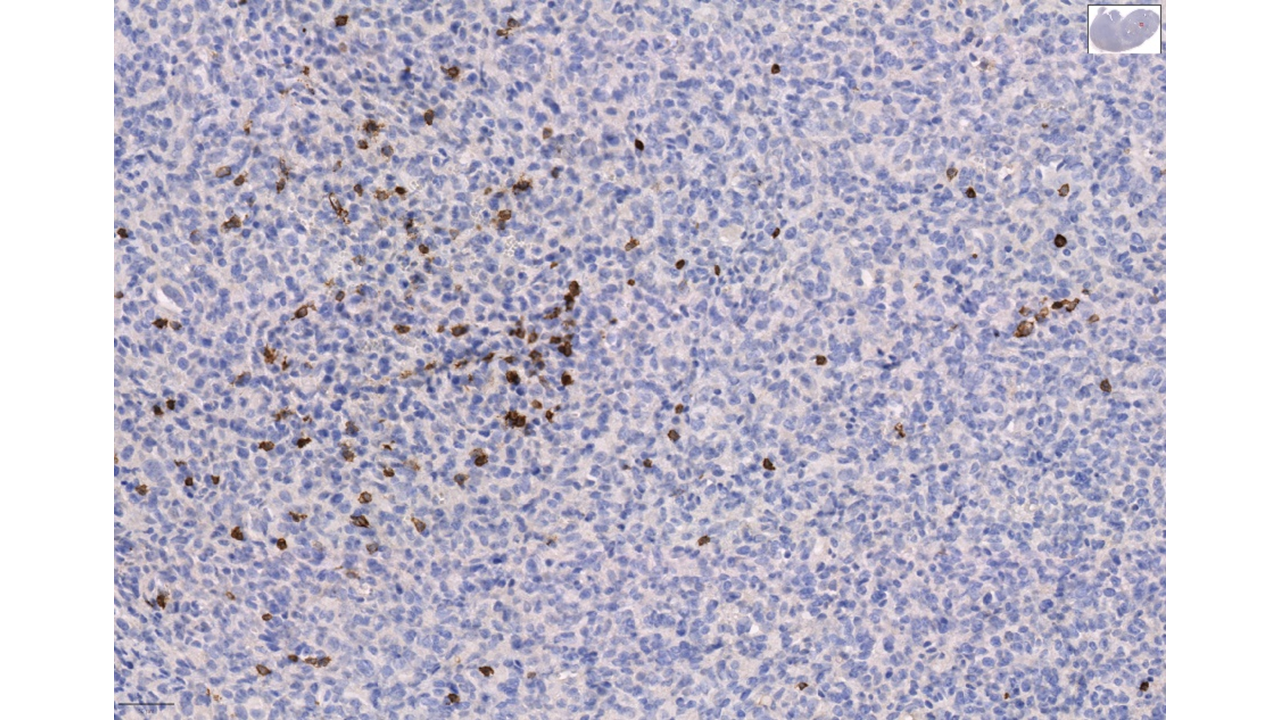

Supplement: Supplementary file 6 — Source data Fig. 3 [file 44318_2026_803_MOESM6_ESM.zip › Fig 3/3B/Combo.tif]

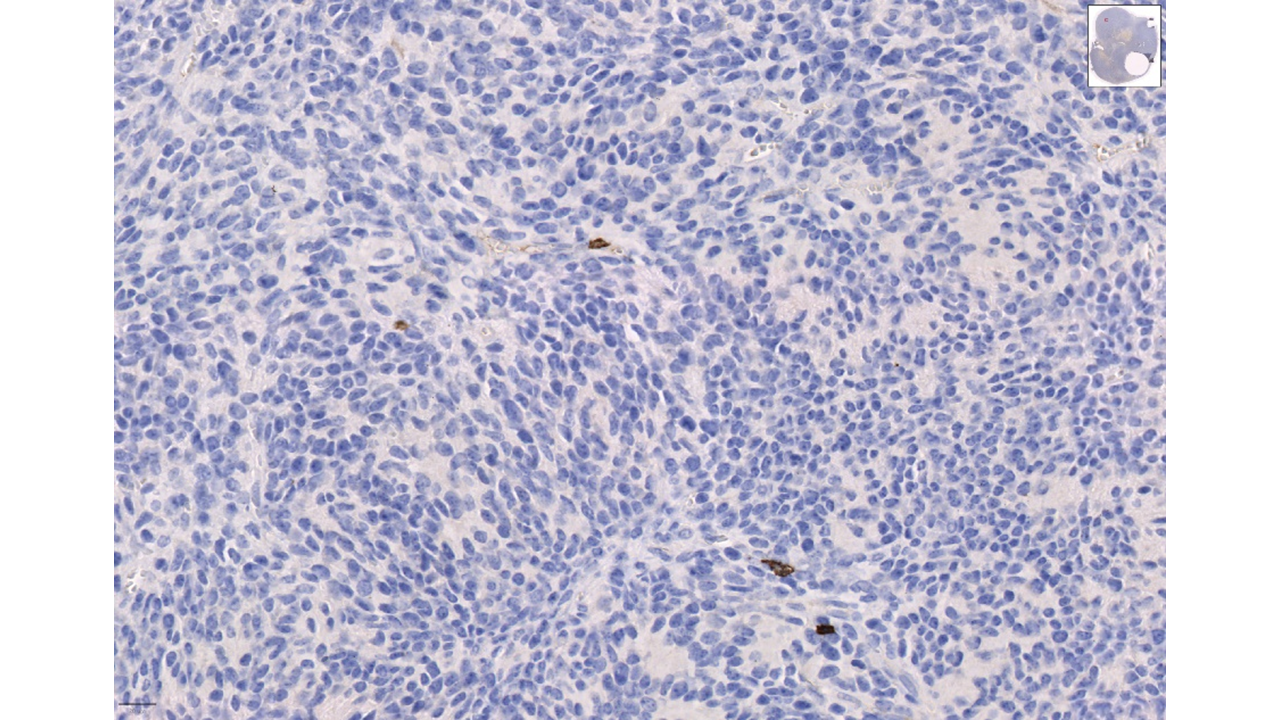

Supplement: Supplementary file 6 — Source data Fig. 3 [file 44318_2026_803_MOESM6_ESM.zip › Fig 3/3B/IgG.tif]

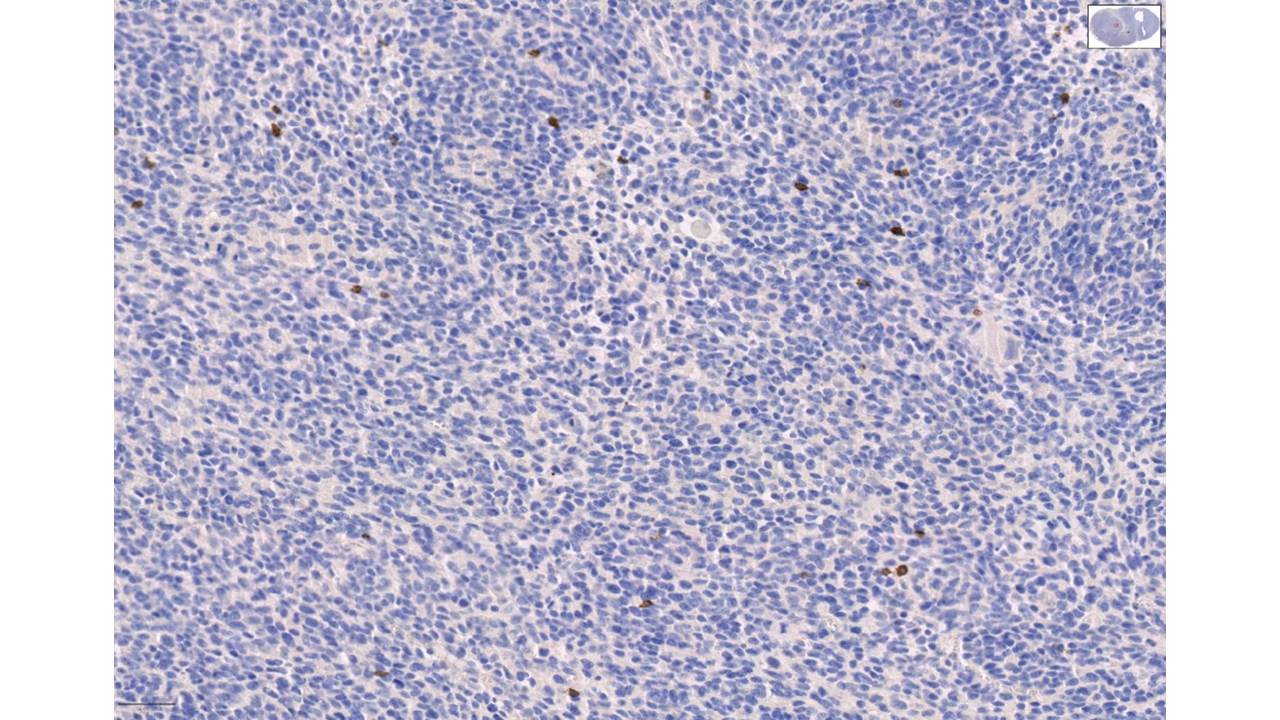

Supplement: Supplementary file 6 — Source data Fig. 3 [file 44318_2026_803_MOESM6_ESM.zip › Fig 3/3B/PD-L1.tif]

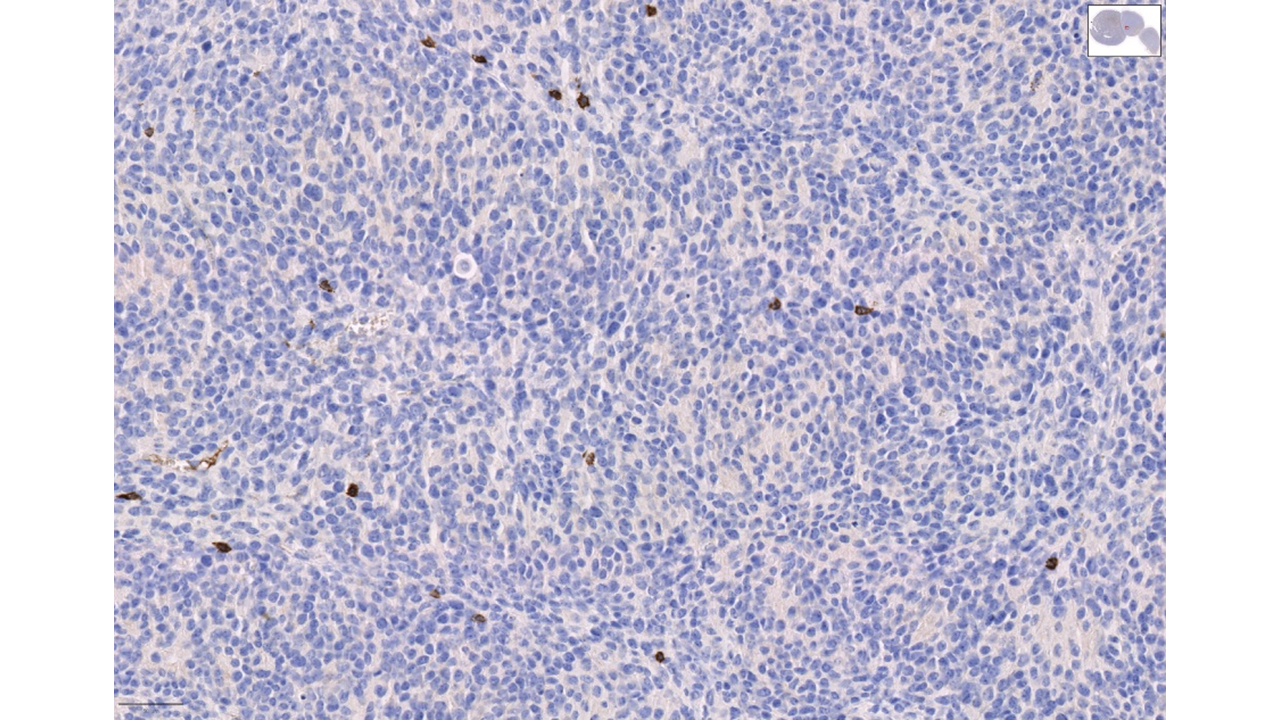

Supplement: Supplementary file 6 — Source data Fig. 3 [file 44318_2026_803_MOESM6_ESM.zip › Fig 3/3B/THX-B.tif]

# B16-F10

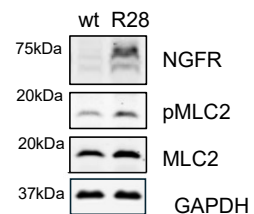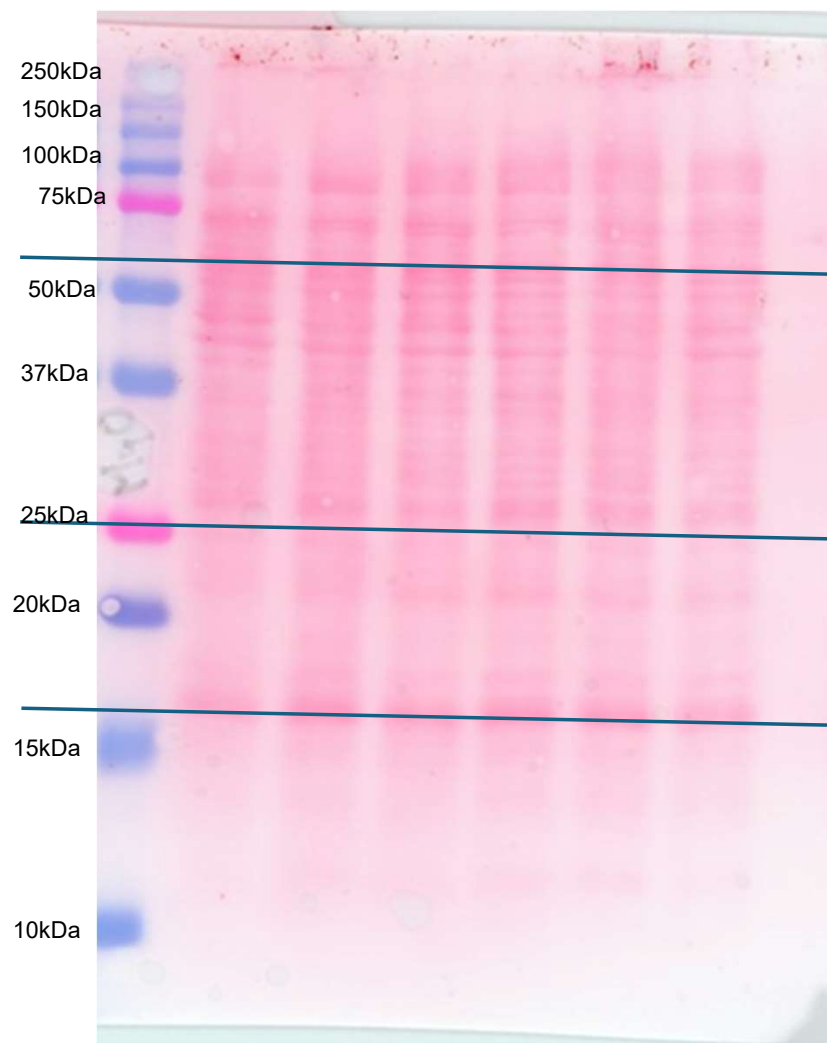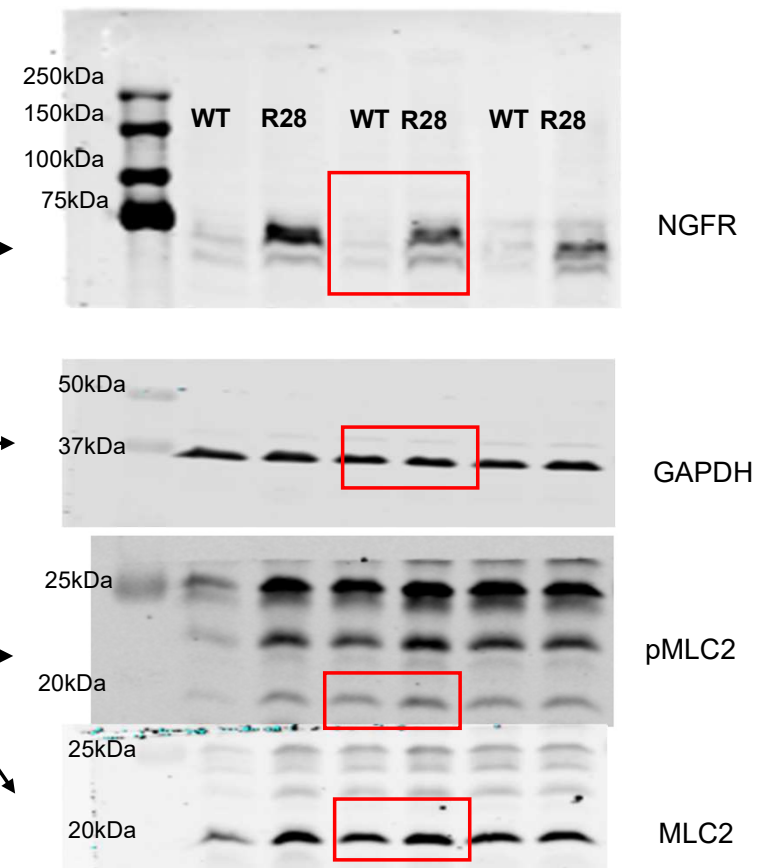

Supplement: Supplementary file 7 — Source data Fig. 4 [file 44318_2026_803_MOESM7_ESM.zip › Fig 4/4A/4A-Readme.pdf]

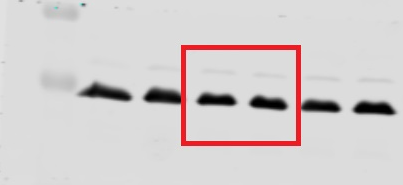

Supplement: Supplementary file 7 — Source data Fig. 4 [file 44318_2026_803_MOESM7_ESM.zip › Fig 4/4A/GAPDH.tif]

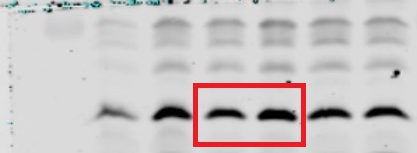

Supplement: Supplementary file 7 — Source data Fig. 4 [file 44318_2026_803_MOESM7_ESM.zip › Fig 4/4A/MLC2.tif]

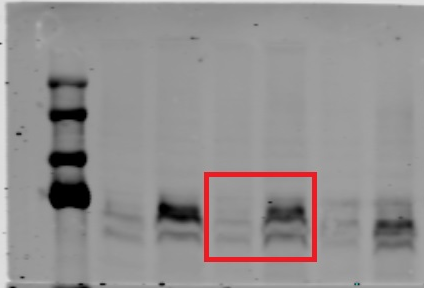

Supplement: Supplementary file 7 — Source data Fig. 4 [file 44318_2026_803_MOESM7_ESM.zip › Fig 4/4A/NGFR.tif]

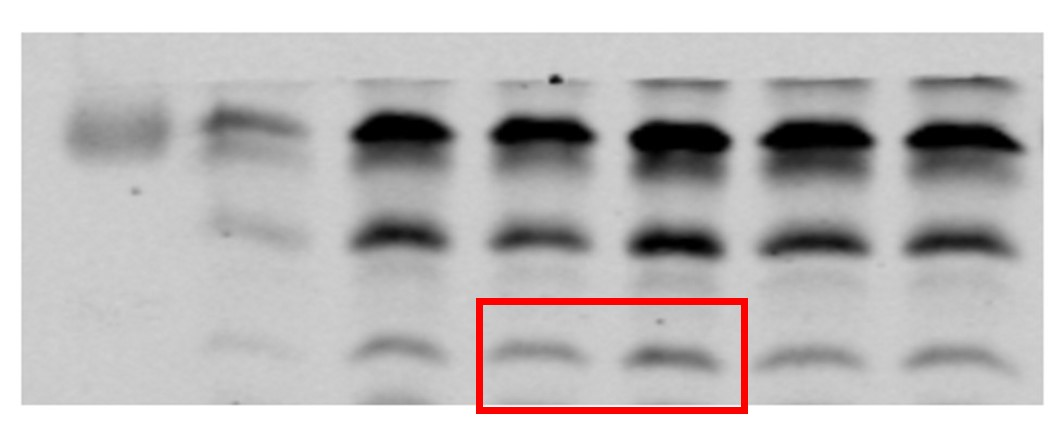

Supplement: Supplementary file 7 — Source data Fig. 4 [file 44318_2026_803_MOESM7_ESM.zip › Fig 4/4A/pMCL2.tif]

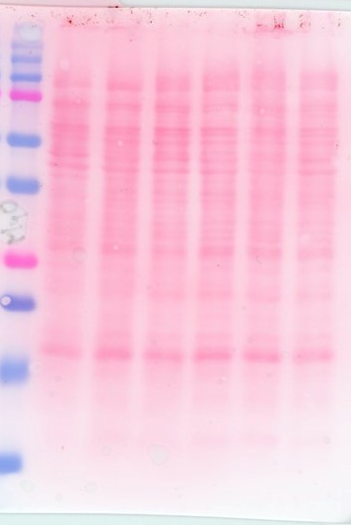

Supplement: Supplementary file 7 — Source data Fig. 4 [file 44318_2026_803_MOESM7_ESM.zip › Fig 4/4A/Ponceau.tif]

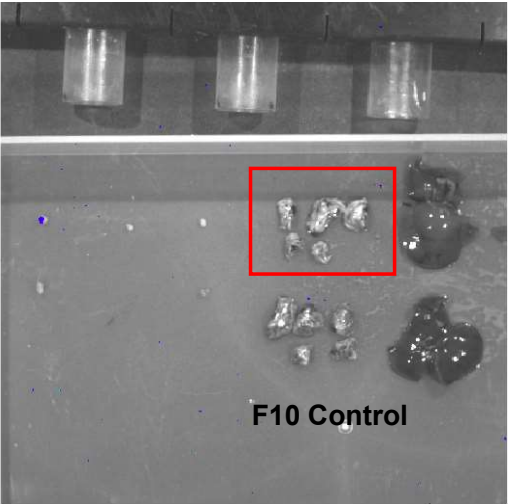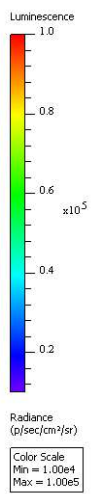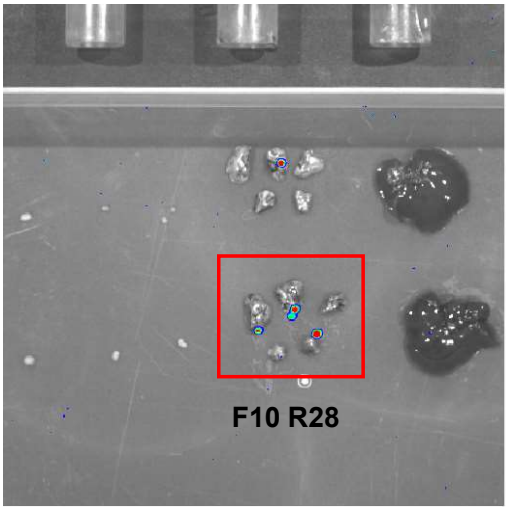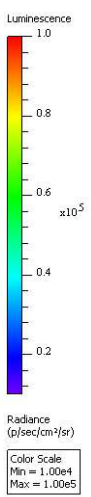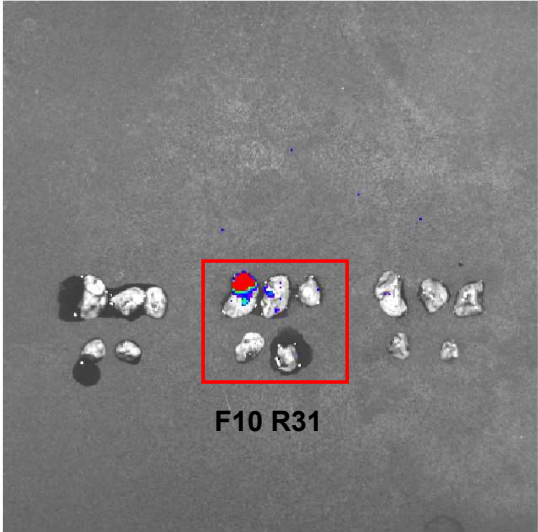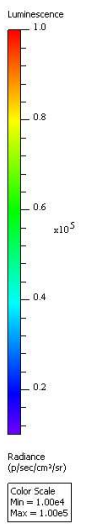

Supplement: Supplementary file 7 — Source data Fig. 4 [file 44318_2026_803_MOESM7_ESM.zip › Fig 4/4F/4F-Readme.pdf]

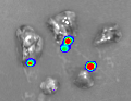

Supplement: Supplementary file 7 — Source data Fig. 4 [file 44318_2026_803_MOESM7_ESM.zip › Fig 4/4F/F10 GL R28-1.tif]

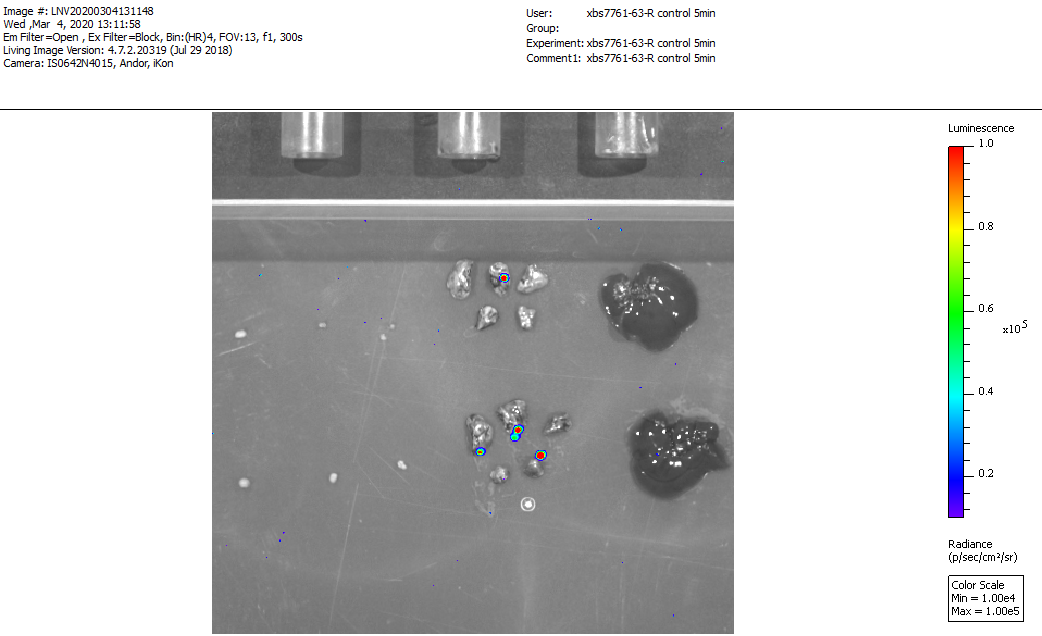

Supplement: Supplementary file 7 — Source data Fig. 4 [file 44318_2026_803_MOESM7_ESM.zip › Fig 4/4F/F10 GL R28.tif]

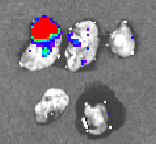

Supplement: Supplementary file 7 — Source data Fig. 4 [file 44318_2026_803_MOESM7_ESM.zip › Fig 4/4F/F10 GL R31-1.tif]

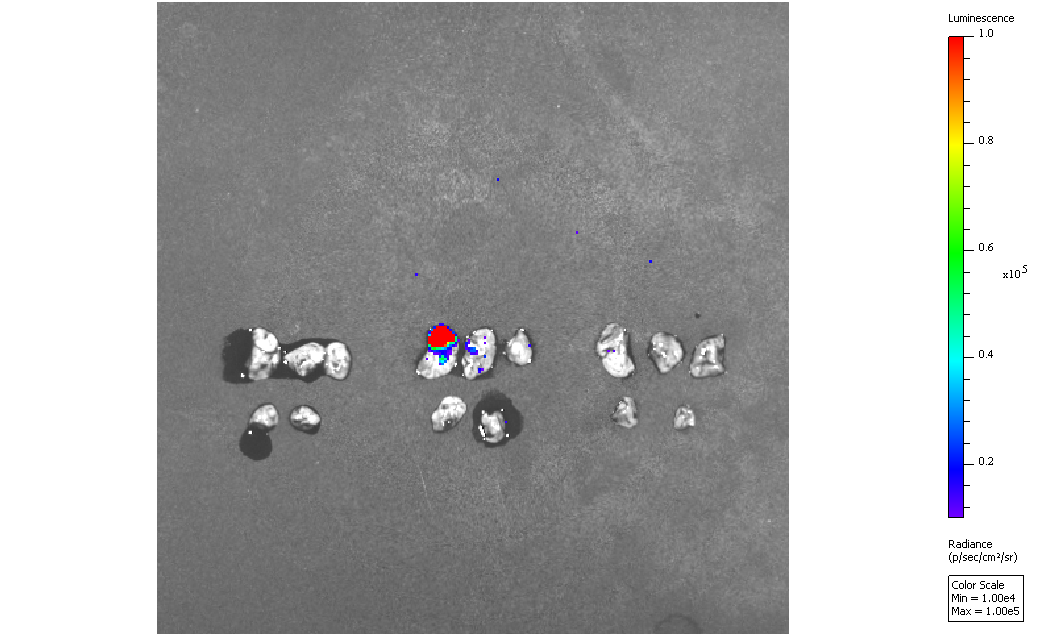

Supplement: Supplementary file 7 — Source data Fig. 4 [file 44318_2026_803_MOESM7_ESM.zip › Fig 4/4F/F10 GL R31.tif]

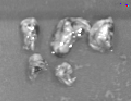

Supplement: Supplementary file 7 — Source data Fig. 4 [file 44318_2026_803_MOESM7_ESM.zip › Fig 4/4F/F10 GL-1.tif]

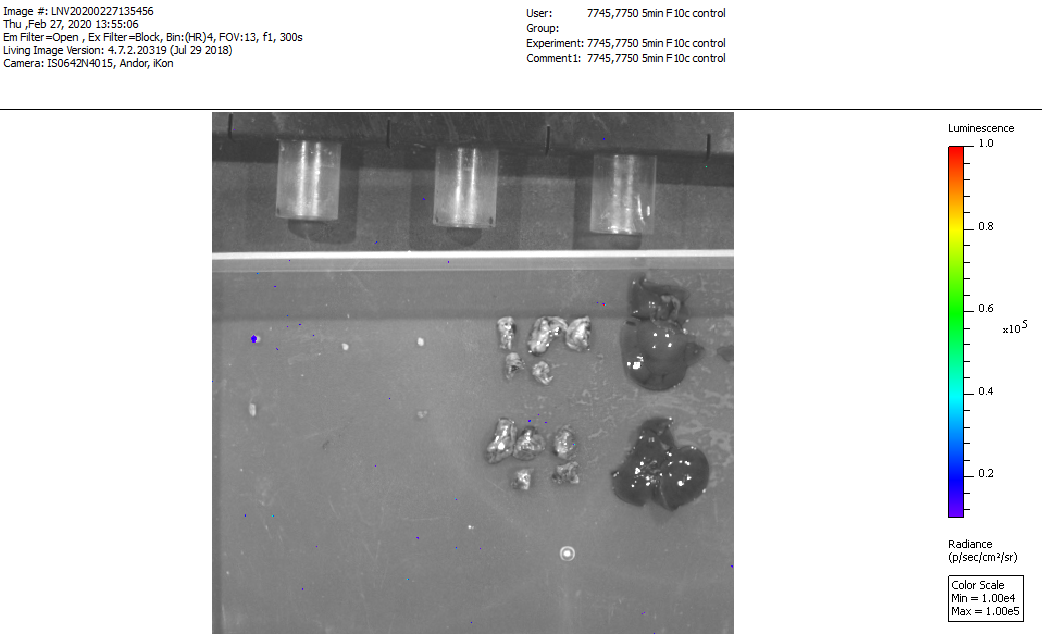

Supplement: Supplementary file 7 — Source data Fig. 4 [file 44318_2026_803_MOESM7_ESM.zip › Fig 4/4F/F10 GL.tif]

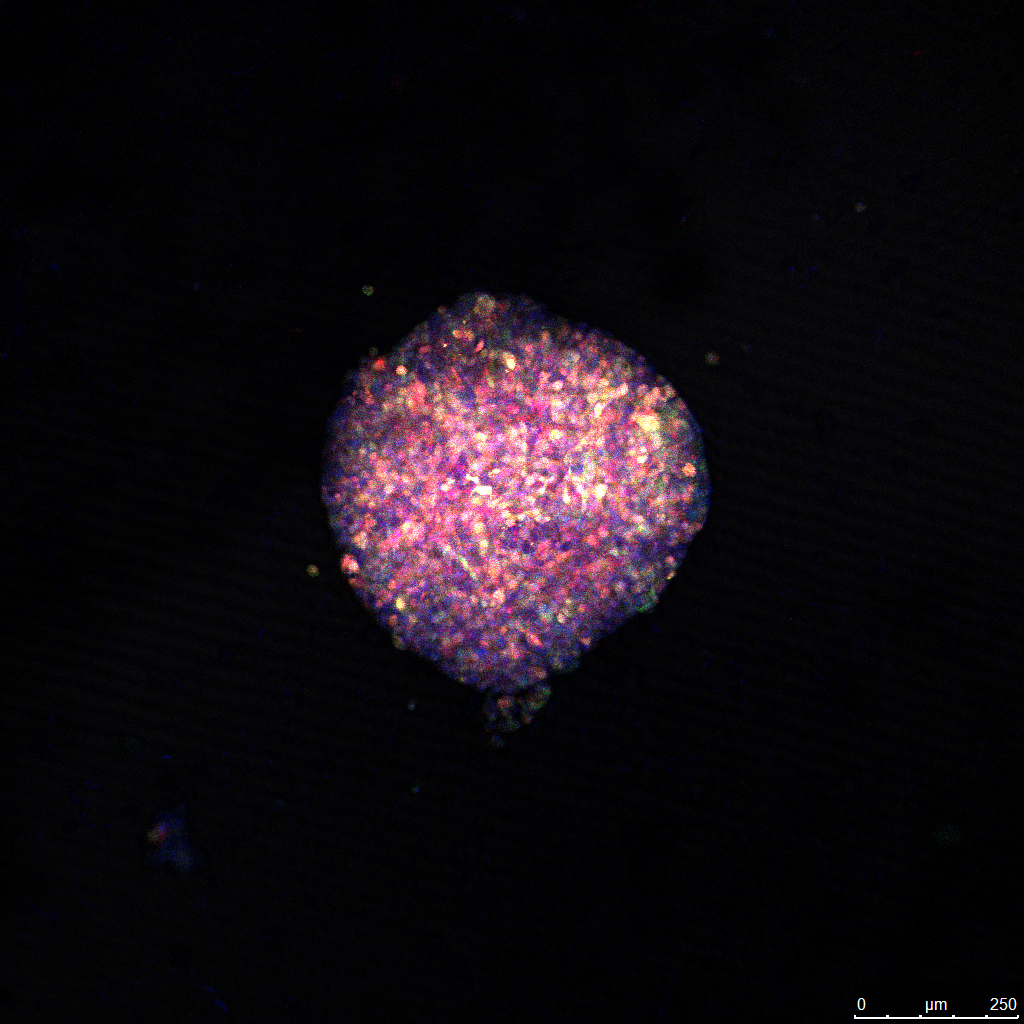

Supplement: Supplementary file 7 — Source data Fig. 4 [file 44318_2026_803_MOESM7_ESM.zip › Fig 4/4G/T0-Series044_z30 merge.tif]

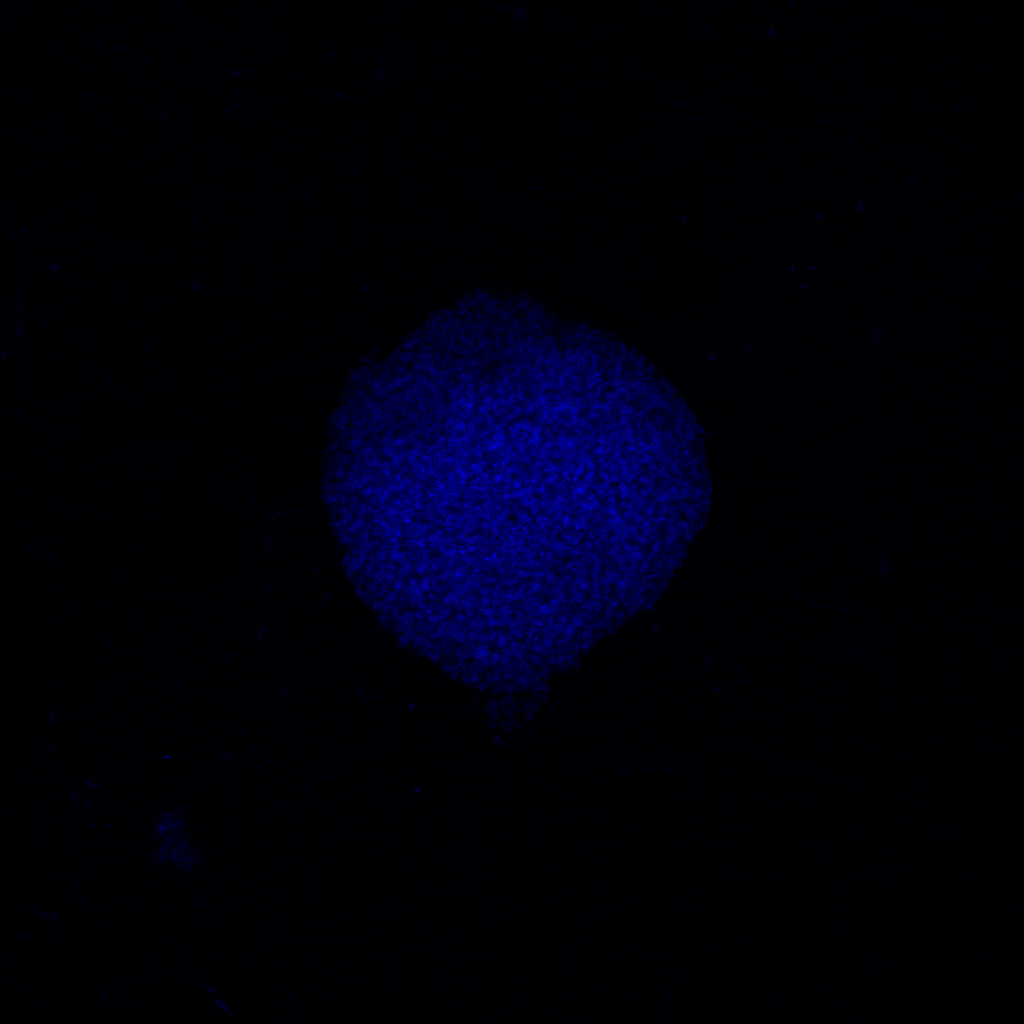

Supplement: Supplementary file 7 — Source data Fig. 4 [file 44318_2026_803_MOESM7_ESM.zip › Fig 4/4G/T0-Series044_z30_ch00 DAPI.tif]

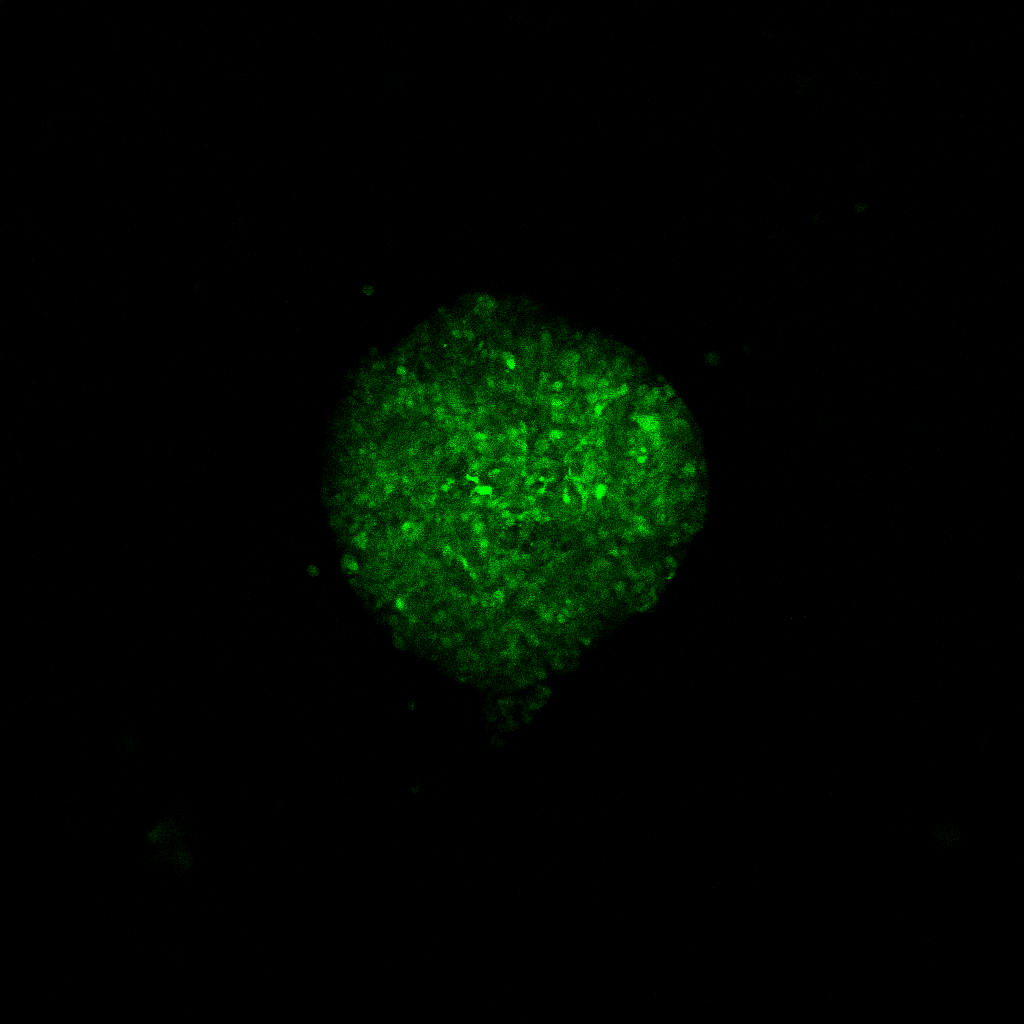

Supplement: Supplementary file 7 — Source data Fig. 4 [file 44318_2026_803_MOESM7_ESM.zip › Fig 4/4G/T0-Series044_z30_ch02-NGFR-GFP.tif]

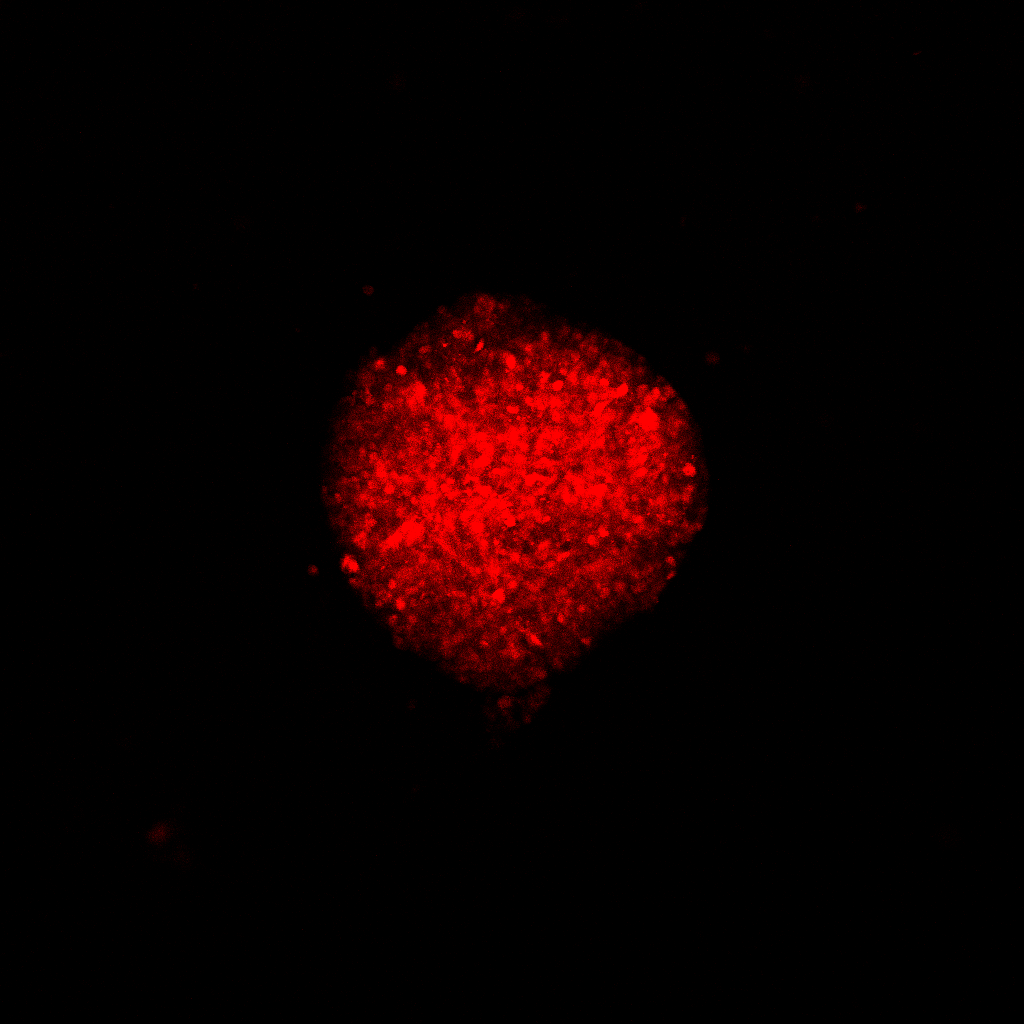

Supplement: Supplementary file 7 — Source data Fig. 4 [file 44318_2026_803_MOESM7_ESM.zip › Fig 4/4G/T0-Series044_z30_ch04-mcherry.tif]

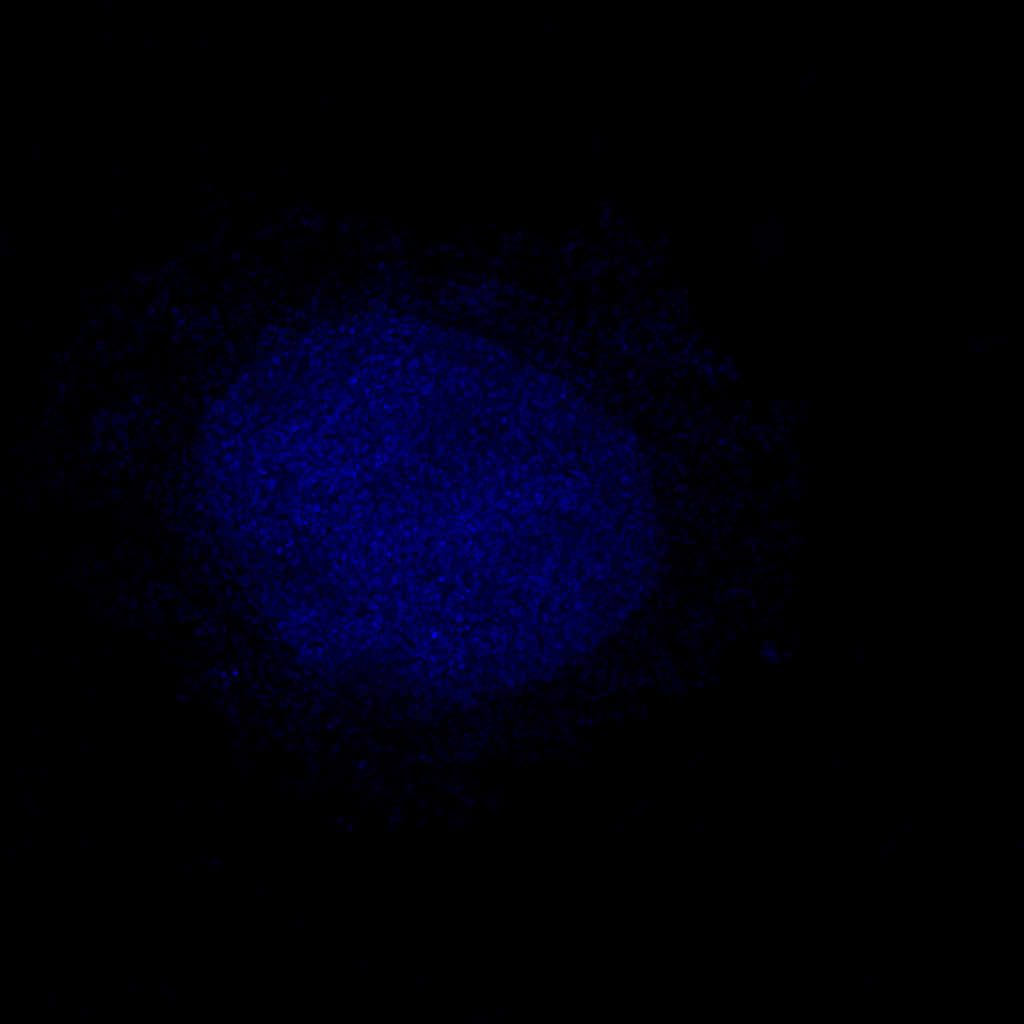

Supplement: Supplementary file 7 — Source data Fig. 4 [file 44318_2026_803_MOESM7_ESM.zip › Fig 4/4G/T2-Series040_z30_ch00 DAPI.tif]

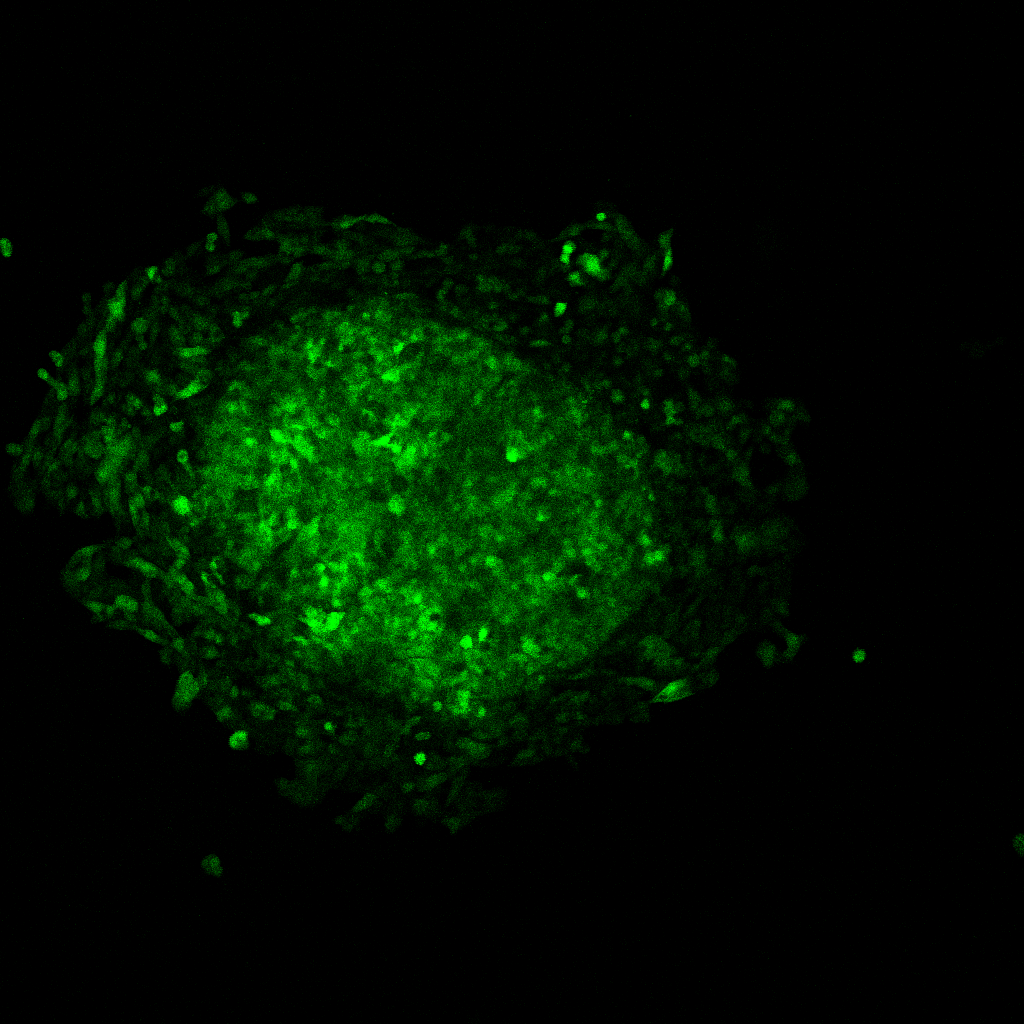

Supplement: Supplementary file 7 — Source data Fig. 4 [file 44318_2026_803_MOESM7_ESM.zip › Fig 4/4G/T2-Series040_z30_ch02-NGFR-GFP.tif]

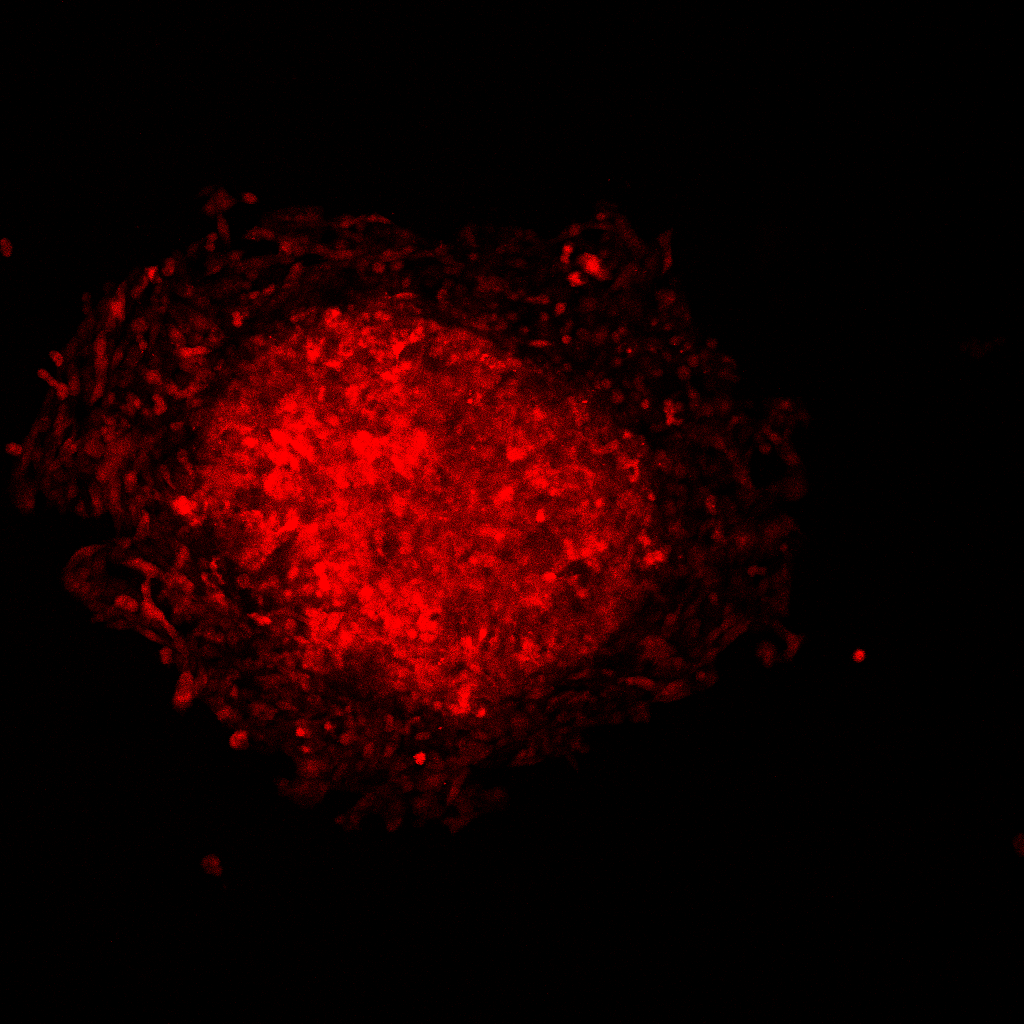

Supplement: Supplementary file 7 — Source data Fig. 4 [file 44318_2026_803_MOESM7_ESM.zip › Fig 4/4G/T2-Series040_z30_ch04-mcherry.tif]

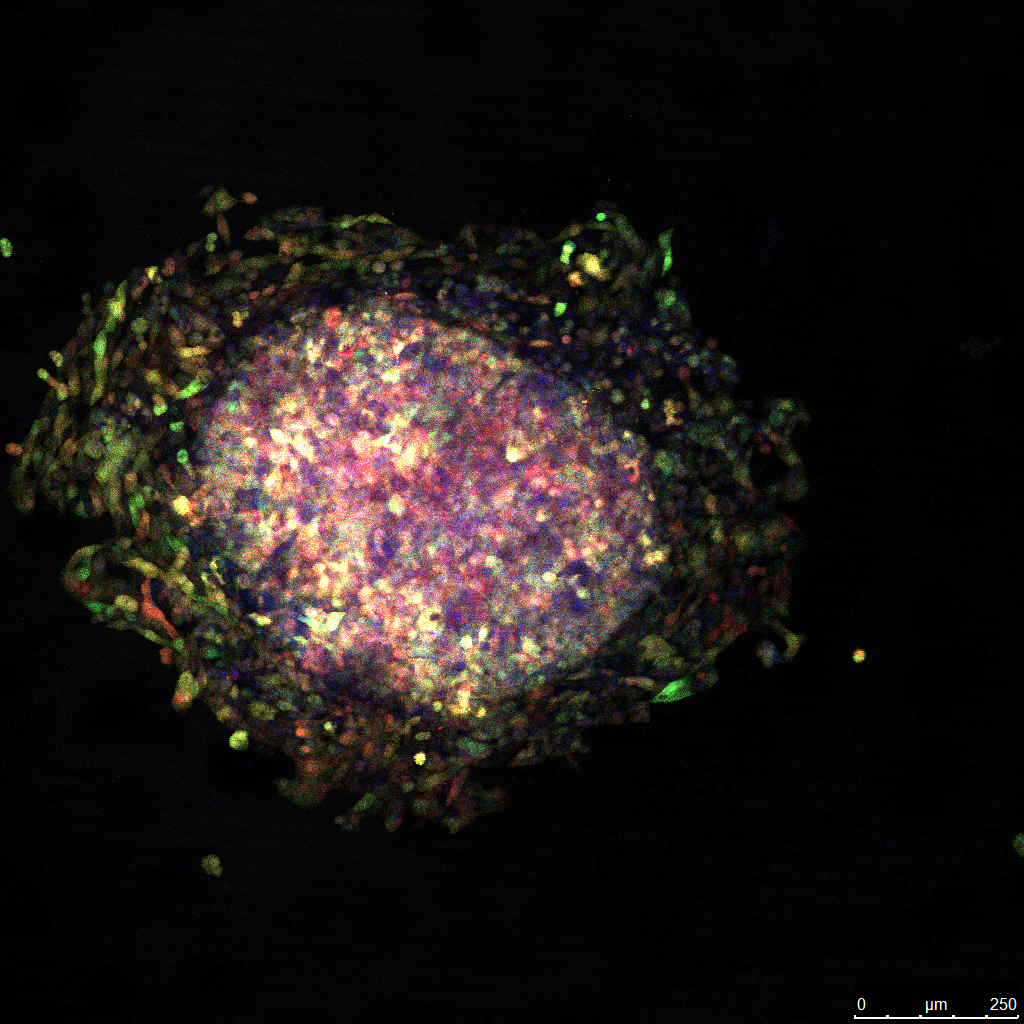

Supplement: Supplementary file 7 — Source data Fig. 4 [file 44318_2026_803_MOESM7_ESM.zip › Fig 4/4G/T2_z30 merge.tif]

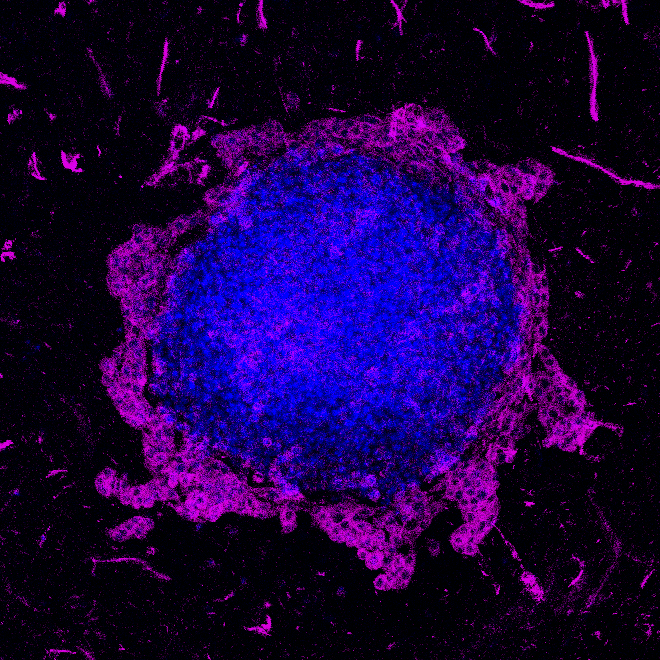

Supplement: Supplementary file 7 — Source data Fig. 4 [file 44318_2026_803_MOESM7_ESM.zip › Fig 4/4I/Invasive T2 E2_14 DAPI and NGFR.tif]

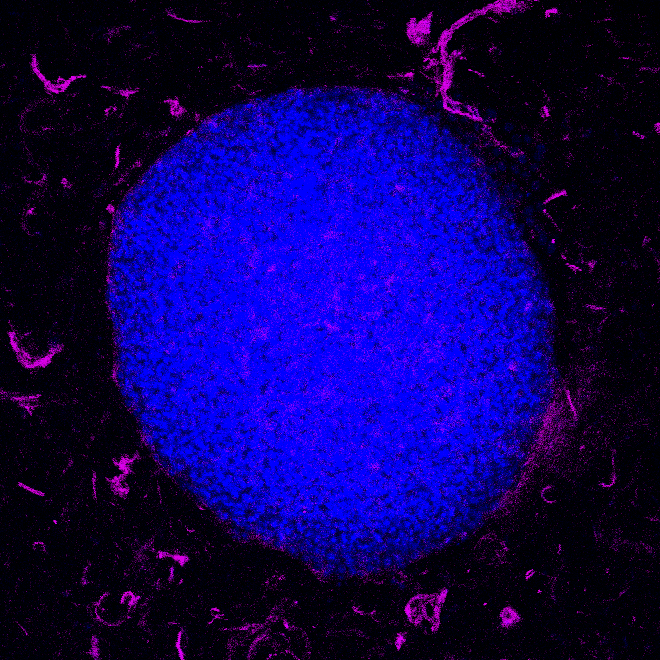

Supplement: Supplementary file 7 — Source data Fig. 4 [file 44318_2026_803_MOESM7_ESM.zip › Fig 4/4I/Non-Invasive T2 E2_12 DAPI-NGFR.tif]

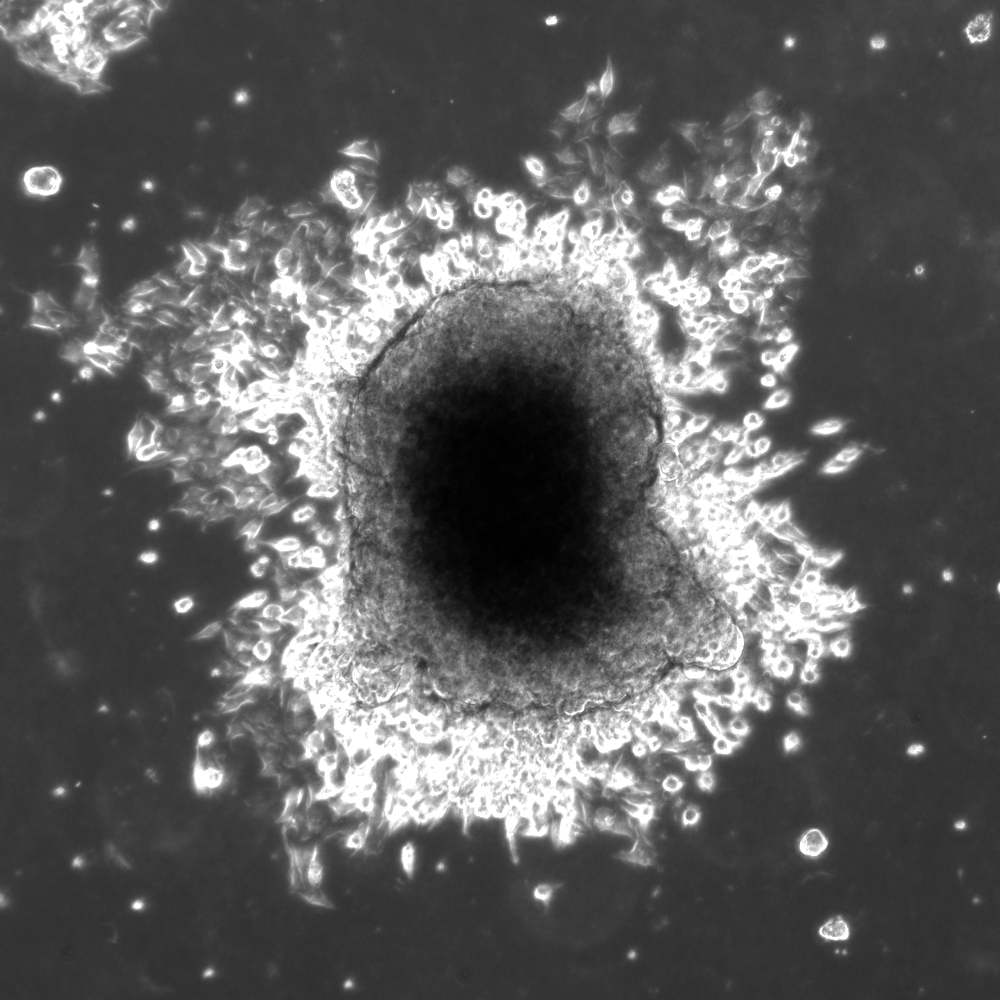

Supplement: Supplementary file 7 — Source data Fig. 4 [file 44318_2026_803_MOESM7_ESM.zip › Fig 4/4K/A3750 CP Control T2.tif]

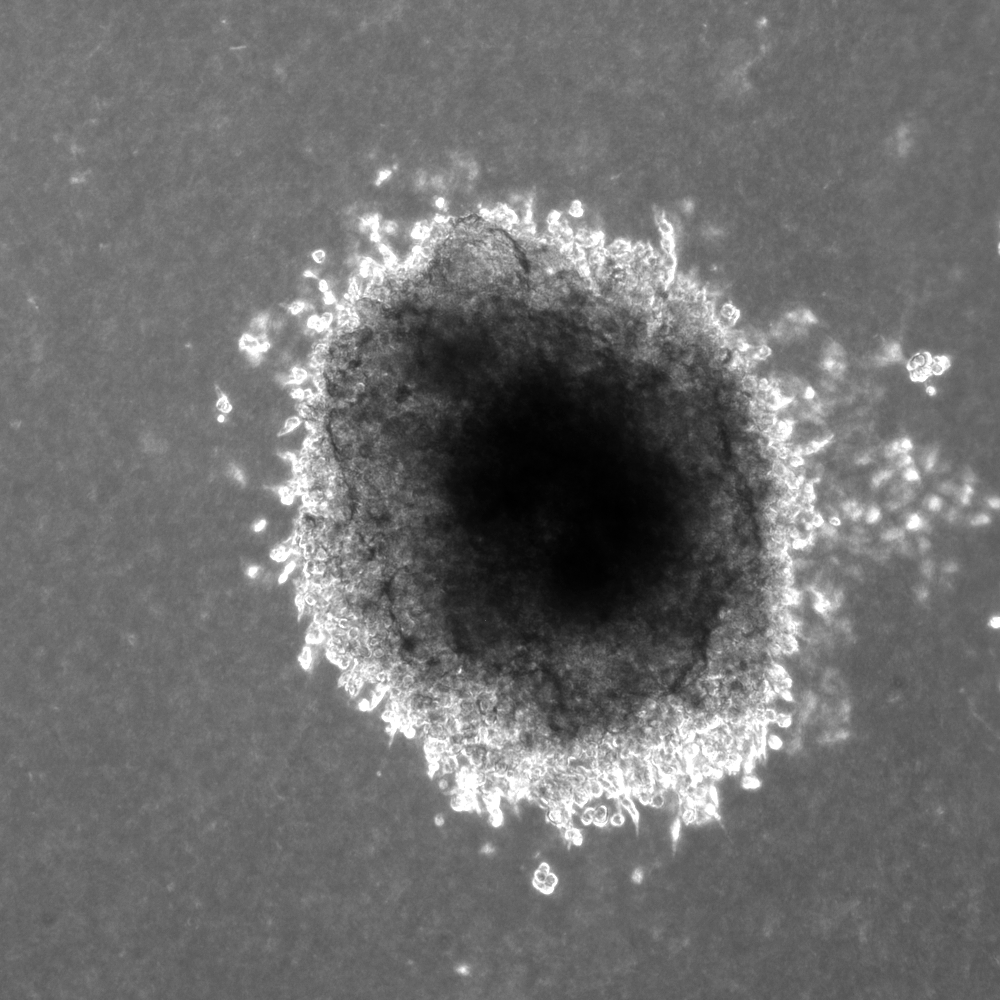

Supplement: Supplementary file 7 — Source data Fig. 4 [file 44318_2026_803_MOESM7_ESM.zip › Fig 4/4K/A3750 CP NGFR1 T2.tif]

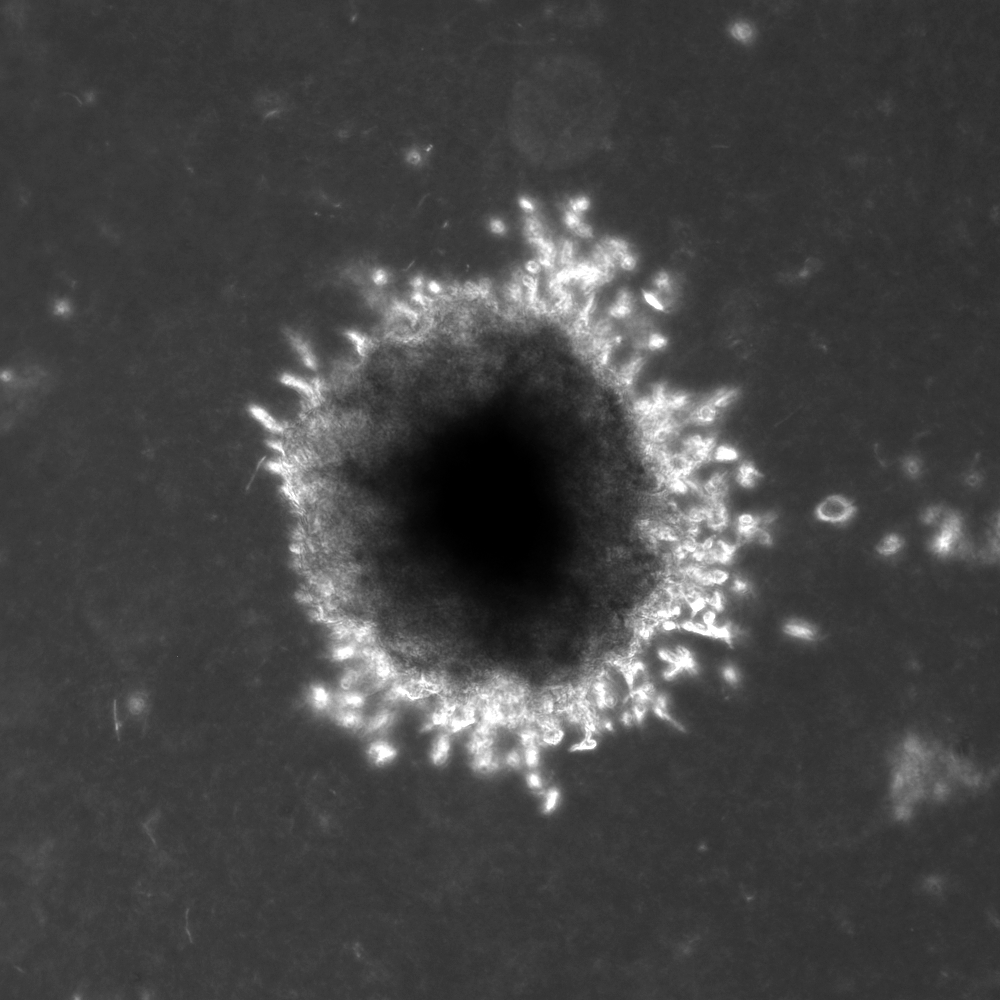

Supplement: Supplementary file 7 — Source data Fig. 4 [file 44318_2026_803_MOESM7_ESM.zip › Fig 4/4K/A3750 CP NGFR2 T2.tif]

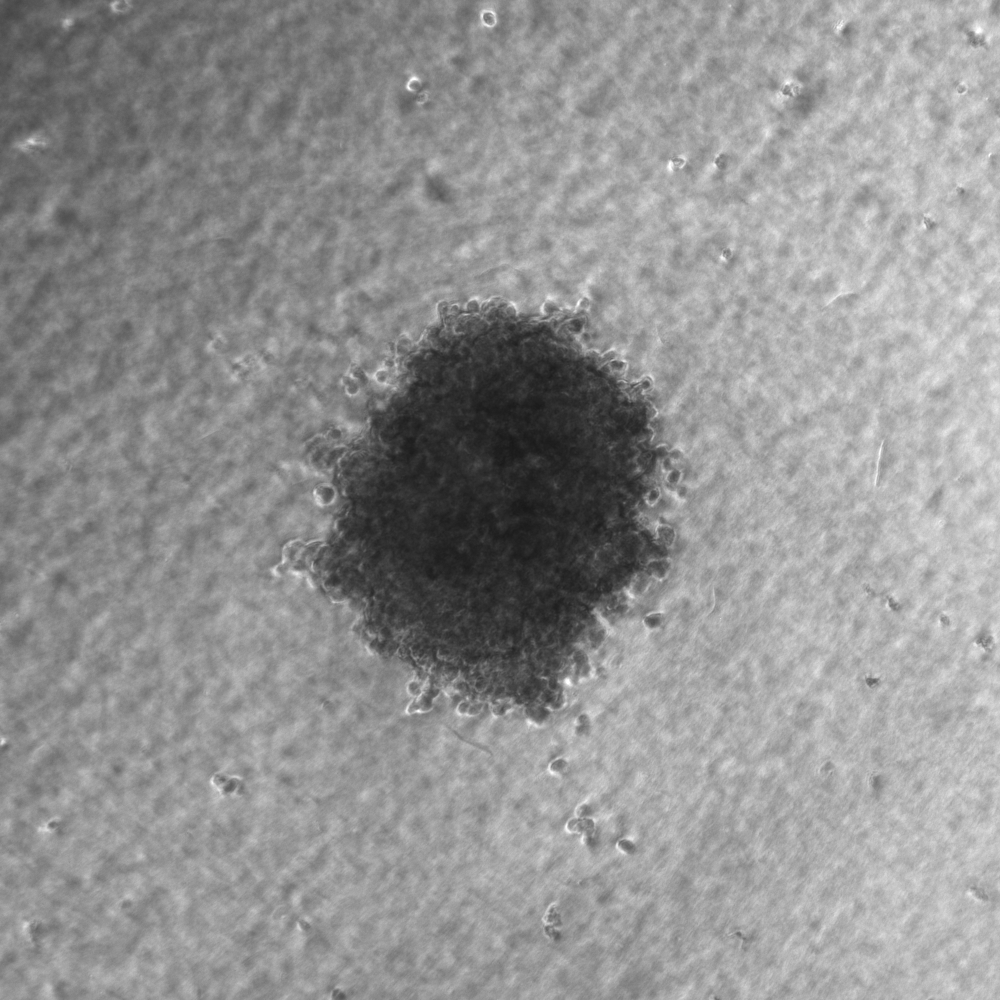

Supplement: Supplementary file 7 — Source data Fig. 4 [file 44318_2026_803_MOESM7_ESM.zip › Fig 4/4K/a375p CP Control t0.tif]

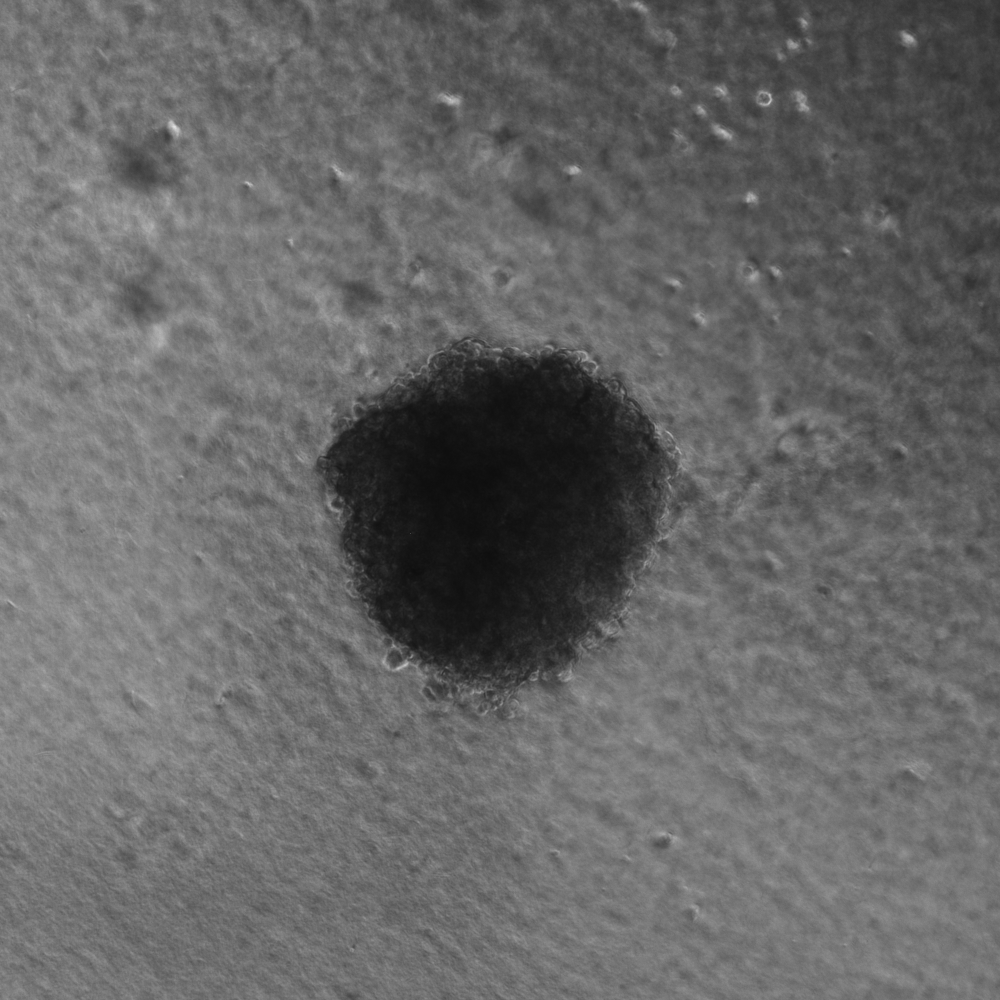

Supplement: Supplementary file 7 — Source data Fig. 4 [file 44318_2026_803_MOESM7_ESM.zip › Fig 4/4K/a375p CP NGFR1 t0.tif]

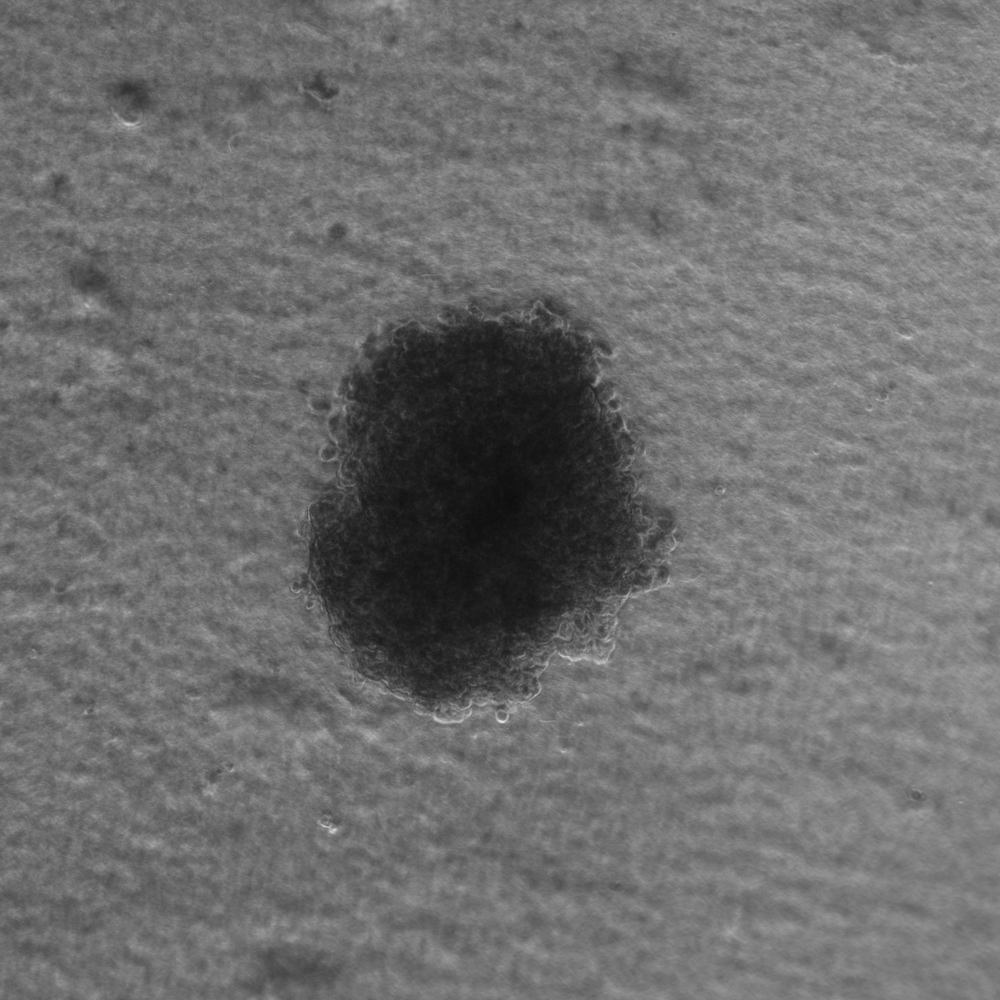

Supplement: Supplementary file 7 — Source data Fig. 4 [file 44318_2026_803_MOESM7_ESM.zip › Fig 4/4K/a375p CP NGFR2 T0.tif]

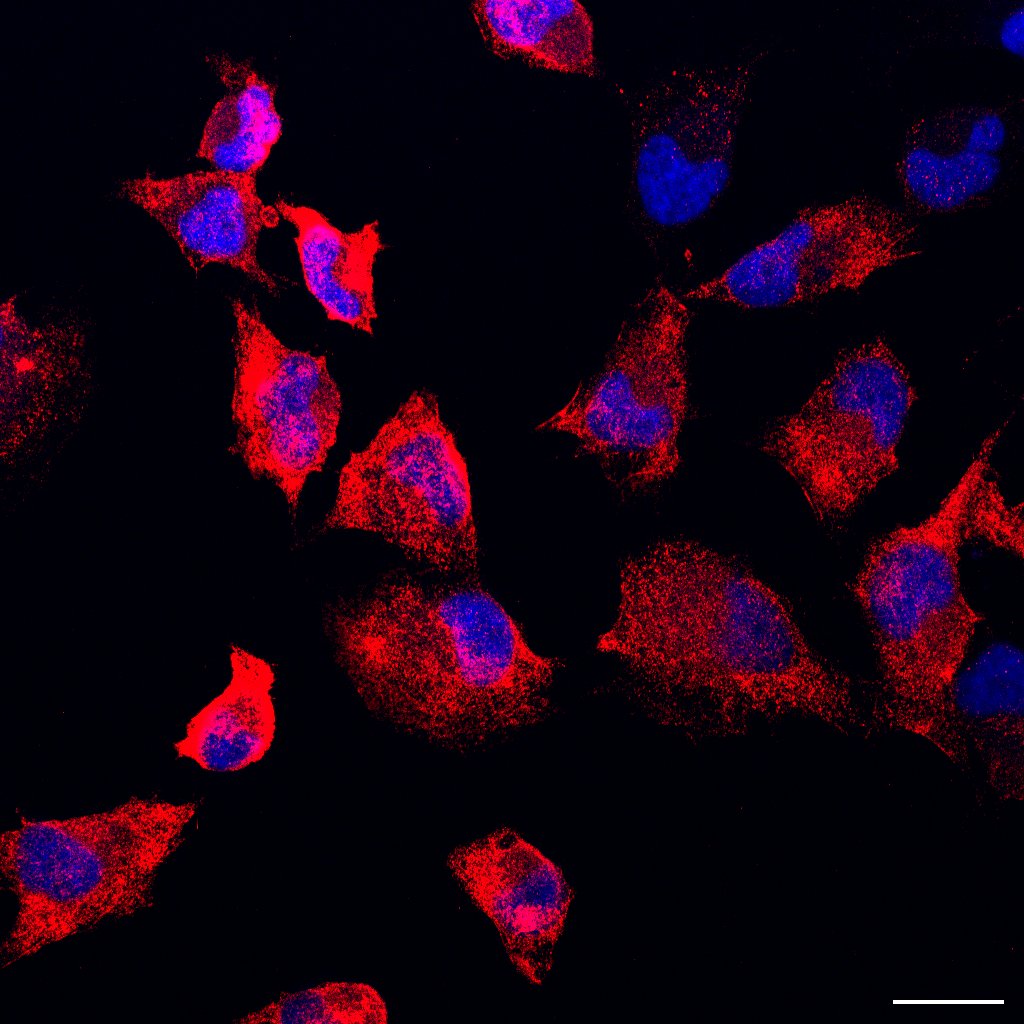

Supplement: Supplementary file 8 — Source data Fig. 5 [file 44318_2026_803_MOESM8_ESM.zip › Fig 5/5A/Control_composite escala.jpg]

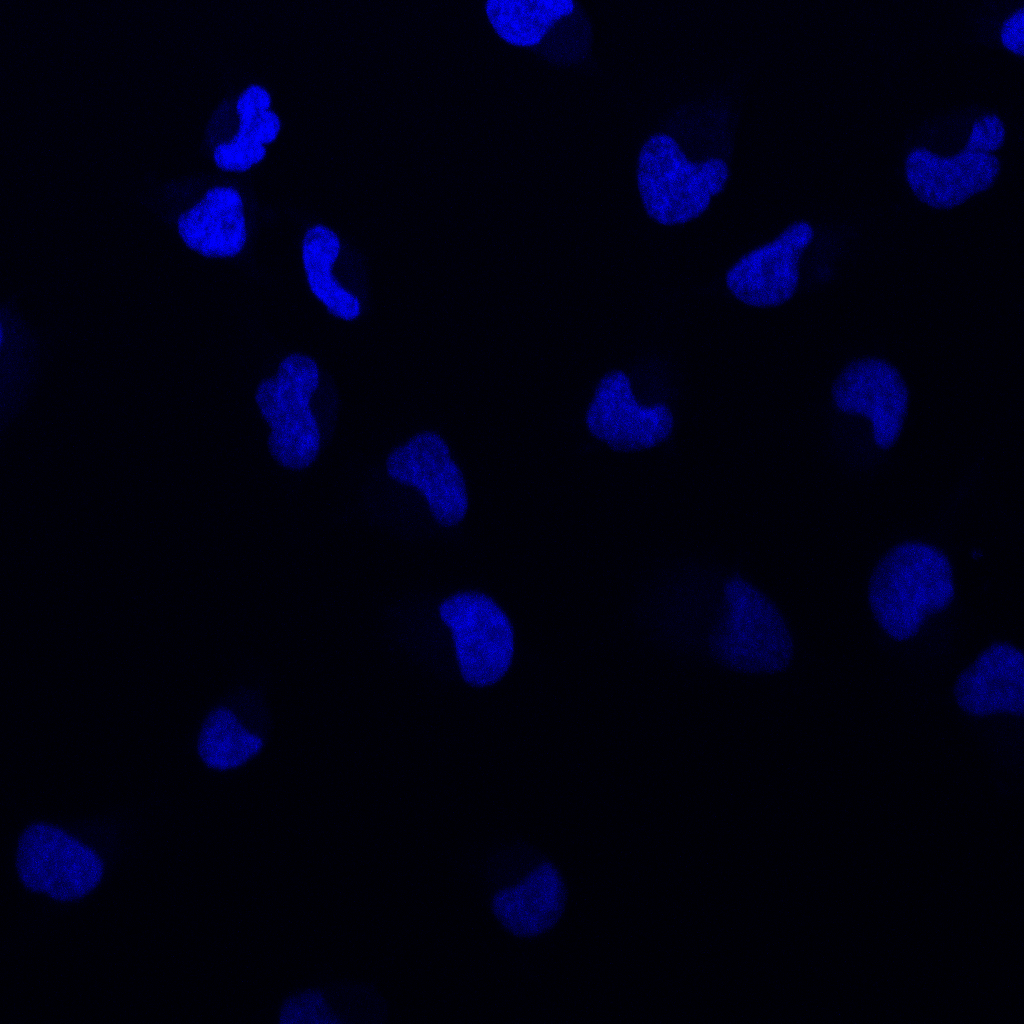

Supplement: Supplementary file 8 — Source data Fig. 5 [file 44318_2026_803_MOESM8_ESM.zip › Fig 5/5A/Control_DAPI.tif]

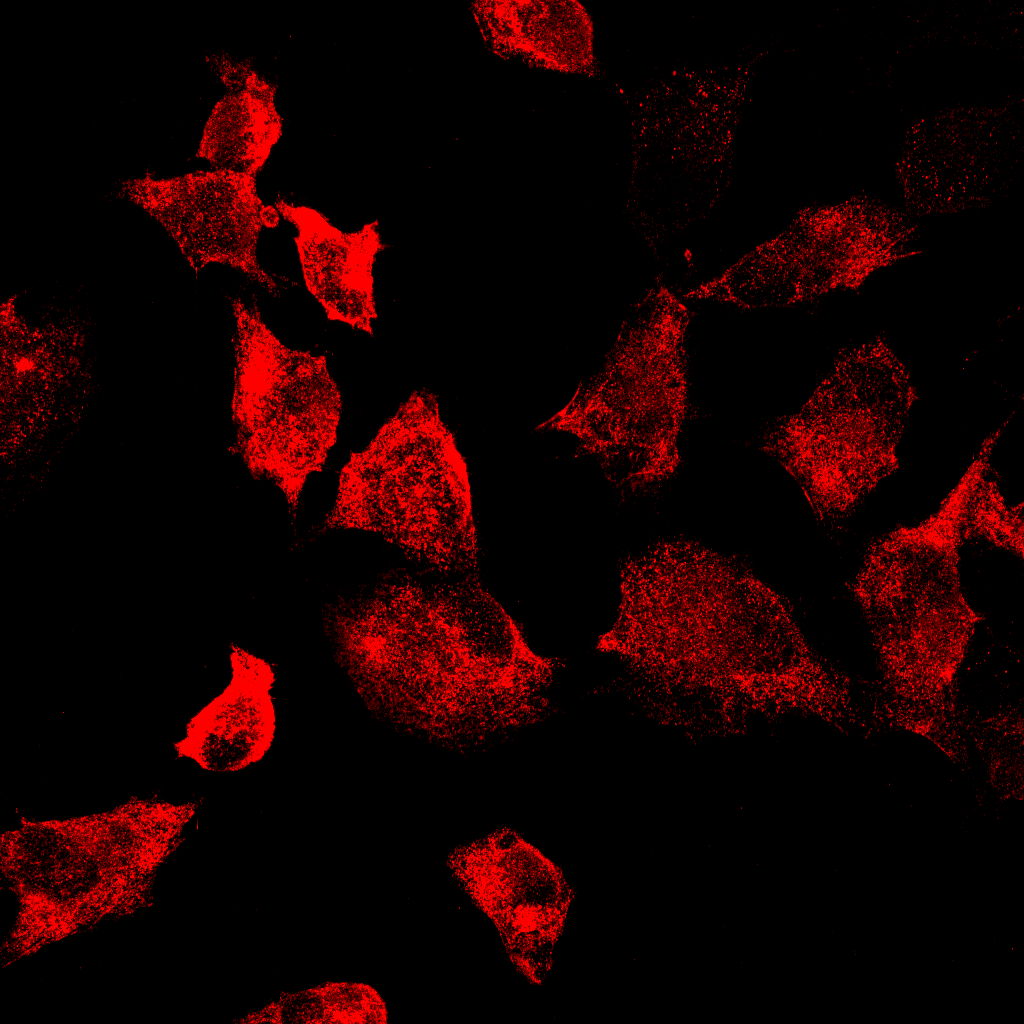

Supplement: Supplementary file 8 — Source data Fig. 5 [file 44318_2026_803_MOESM8_ESM.zip › Fig 5/5A/Control_pMLC2.tif]

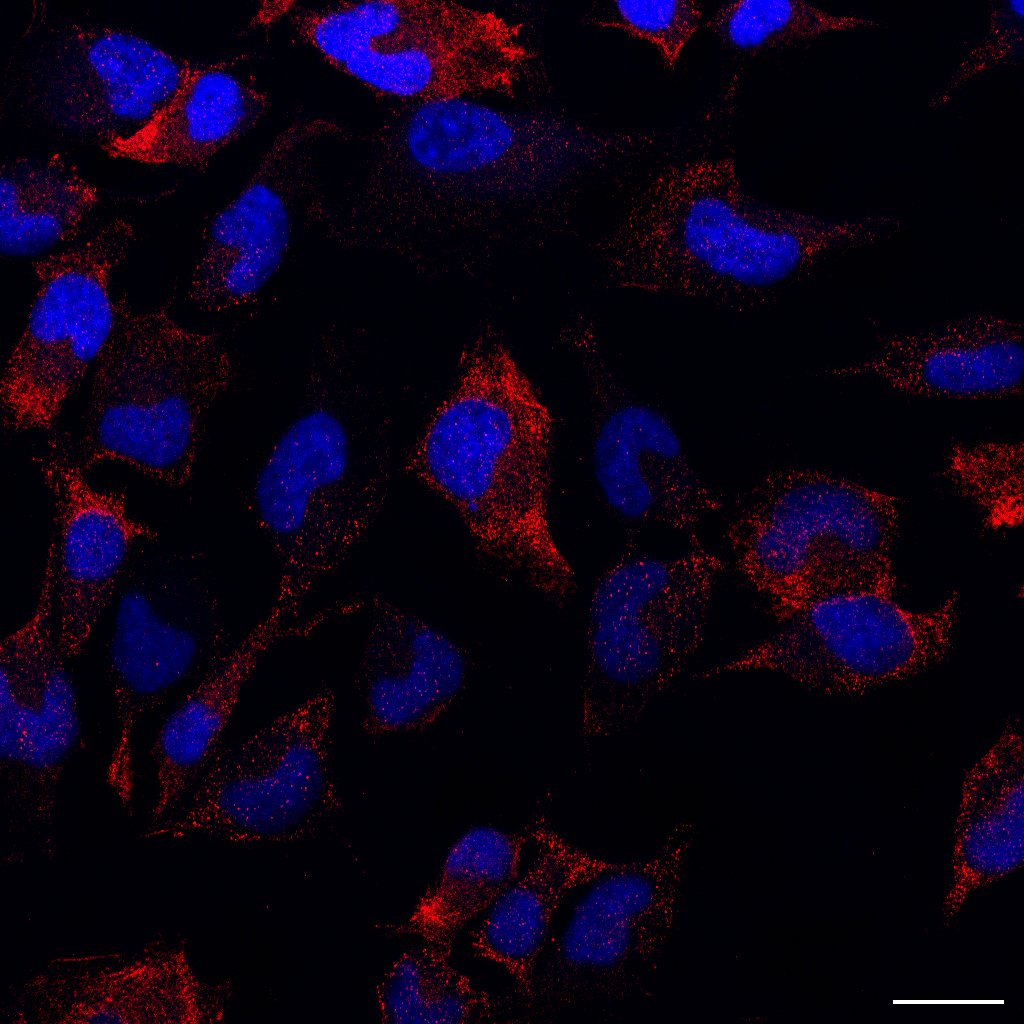

Supplement: Supplementary file 8 — Source data Fig. 5 [file 44318_2026_803_MOESM8_ESM.zip › Fig 5/5A/THX-B_composite escala.jpg]

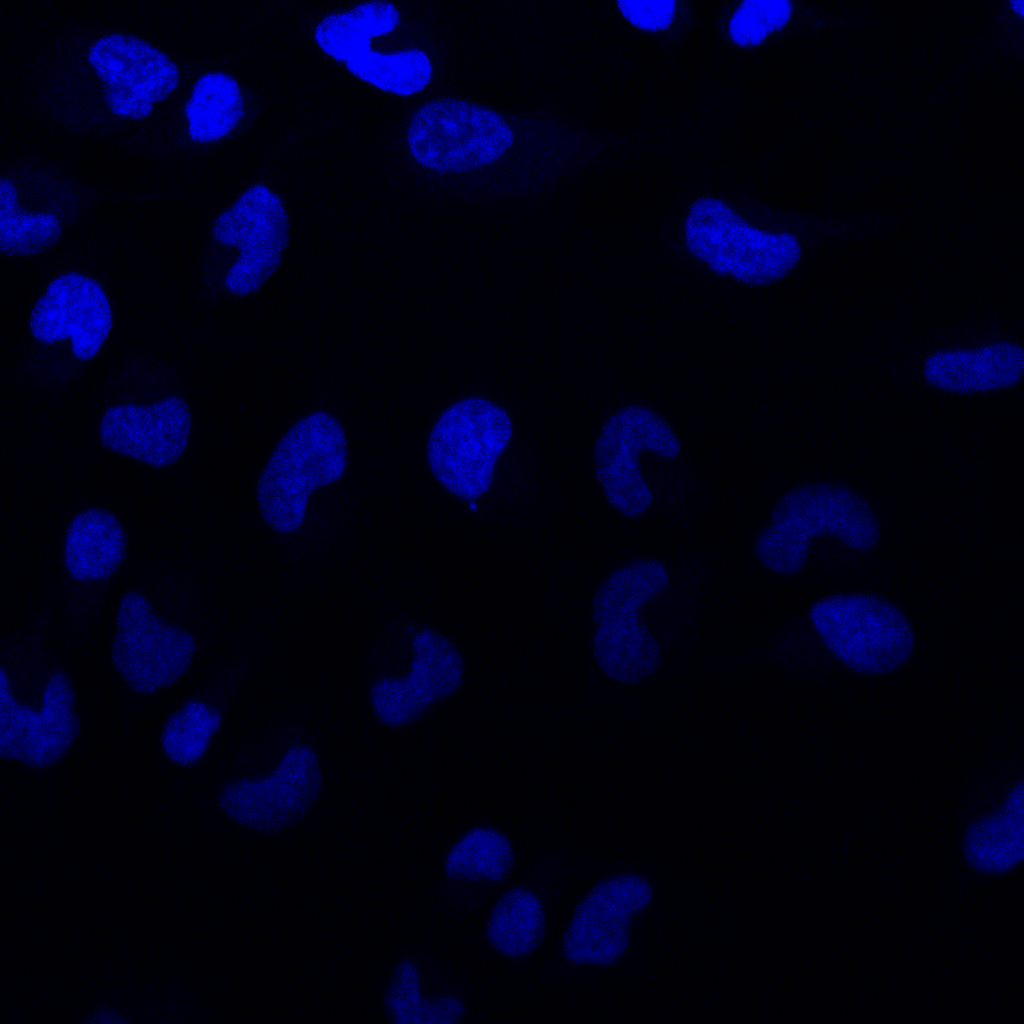

Supplement: Supplementary file 8 — Source data Fig. 5 [file 44318_2026_803_MOESM8_ESM.zip › Fig 5/5A/THX-B_DAPI.tif]

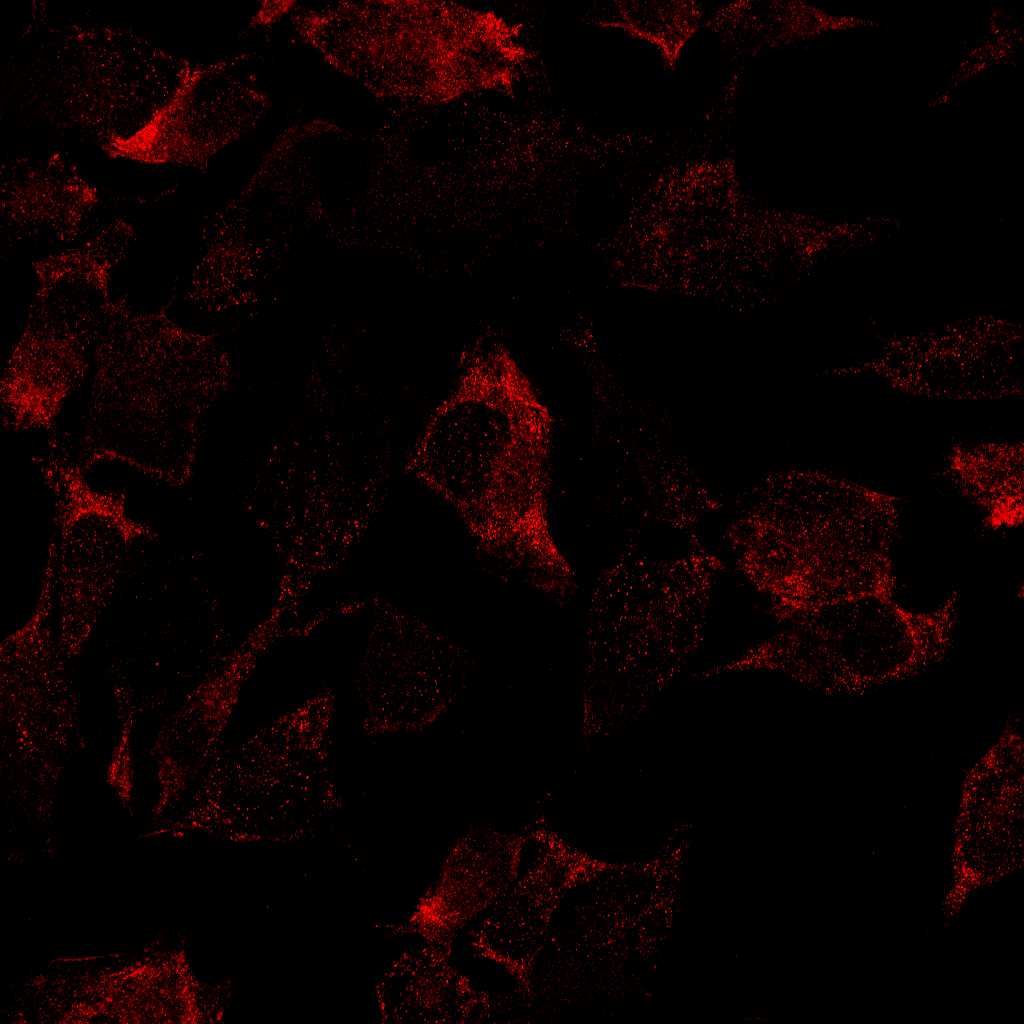

Supplement: Supplementary file 8 — Source data Fig. 5 [file 44318_2026_803_MOESM8_ESM.zip › Fig 5/5A/THX-B_pMLC2.tif]

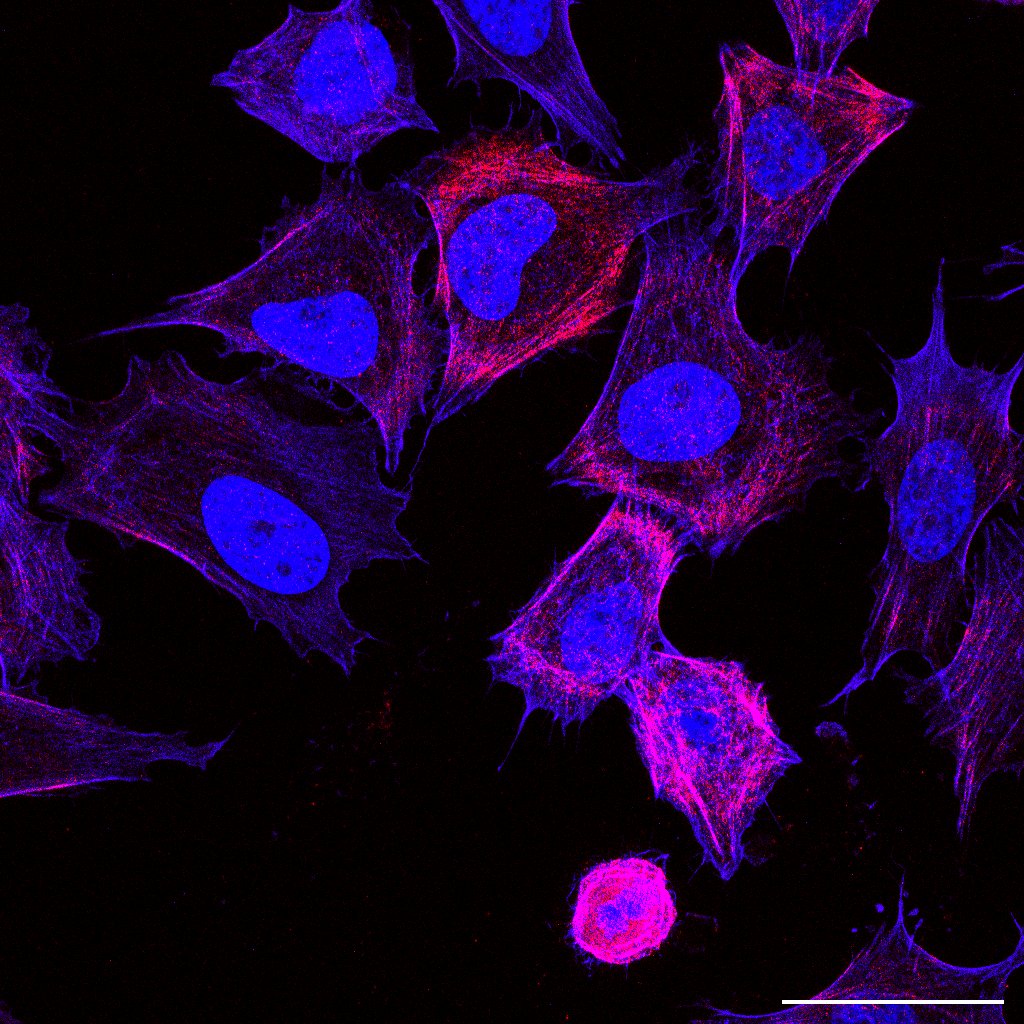

Supplement: Supplementary file 8 — Source data Fig. 5 [file 44318_2026_803_MOESM8_ESM.zip › Fig 5/5C/cpC composite.jpg]

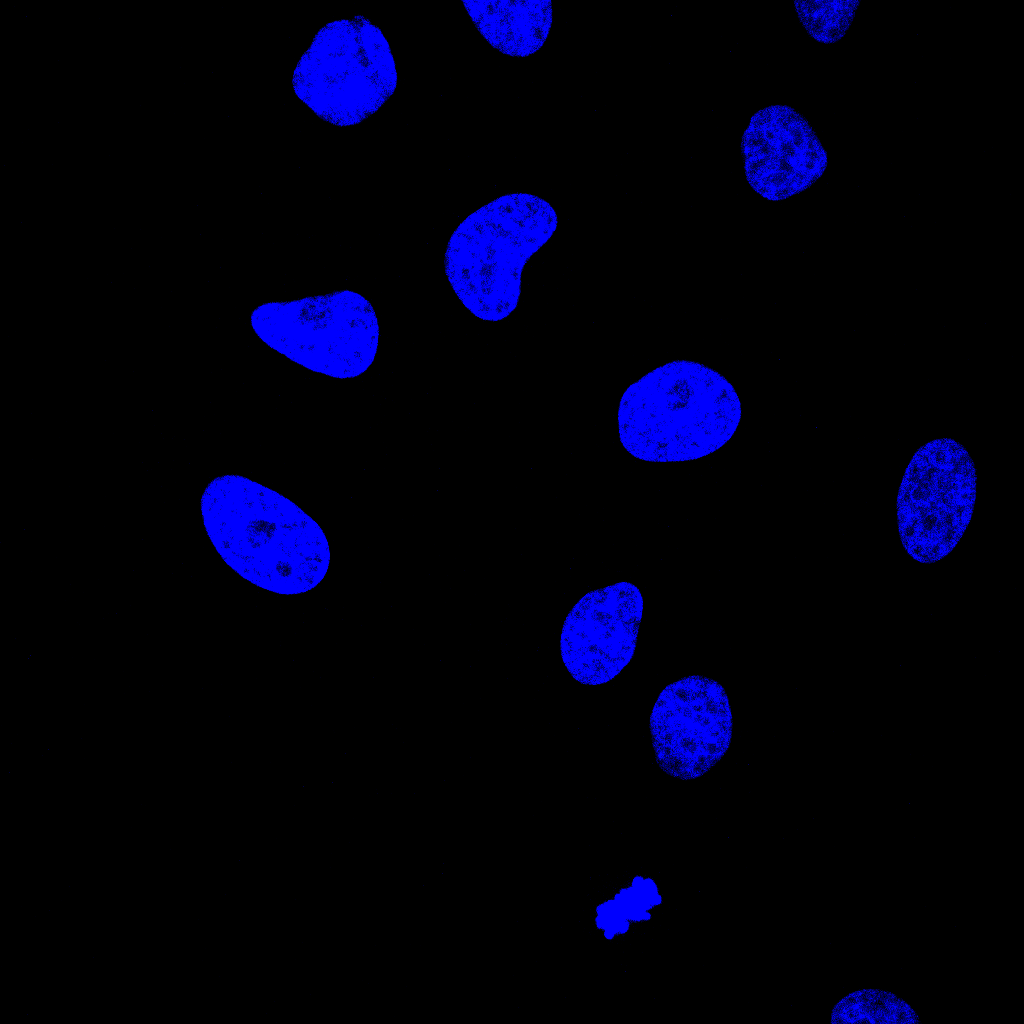

Supplement: Supplementary file 8 — Source data Fig. 5 [file 44318_2026_803_MOESM8_ESM.zip › Fig 5/5C/cpC_DAPI.tif]

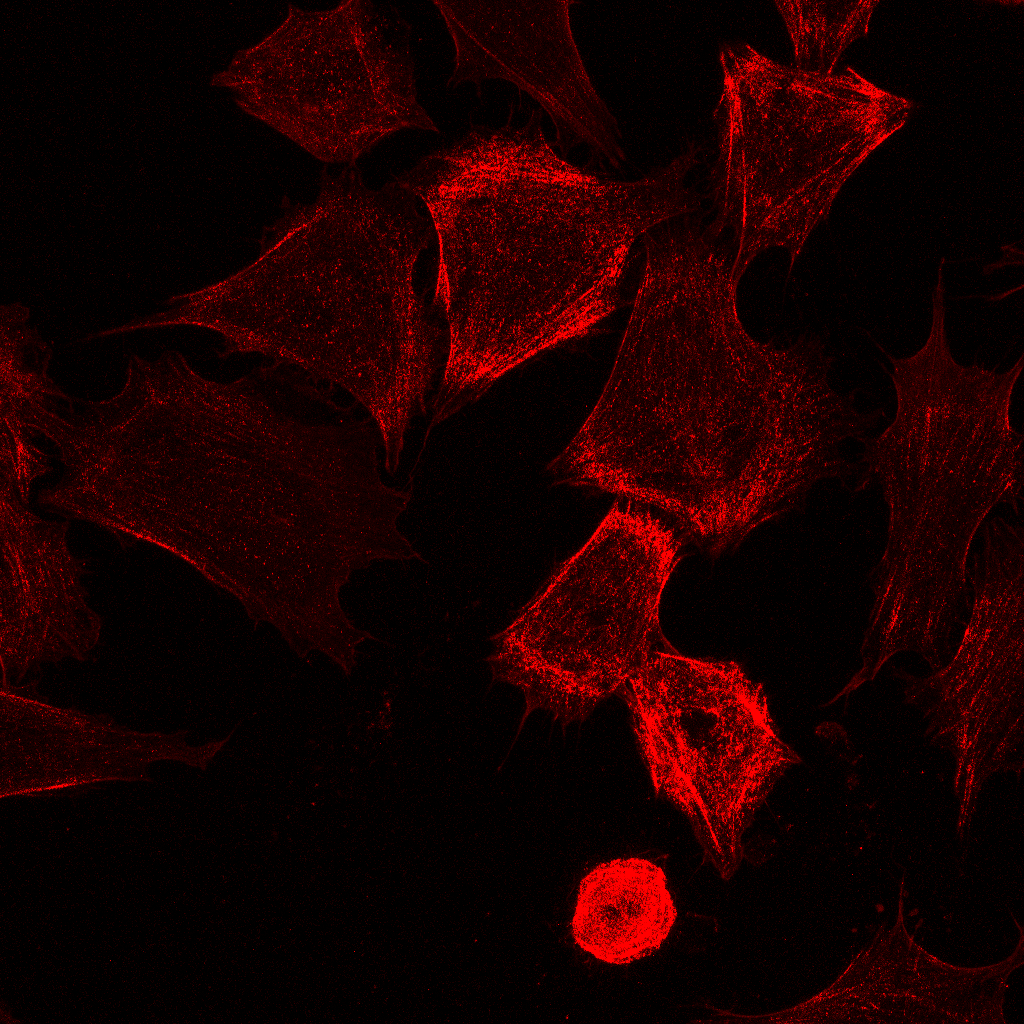

Supplement: Supplementary file 8 — Source data Fig. 5 [file 44318_2026_803_MOESM8_ESM.zip › Fig 5/5C/cpC_pMLC2.tif]

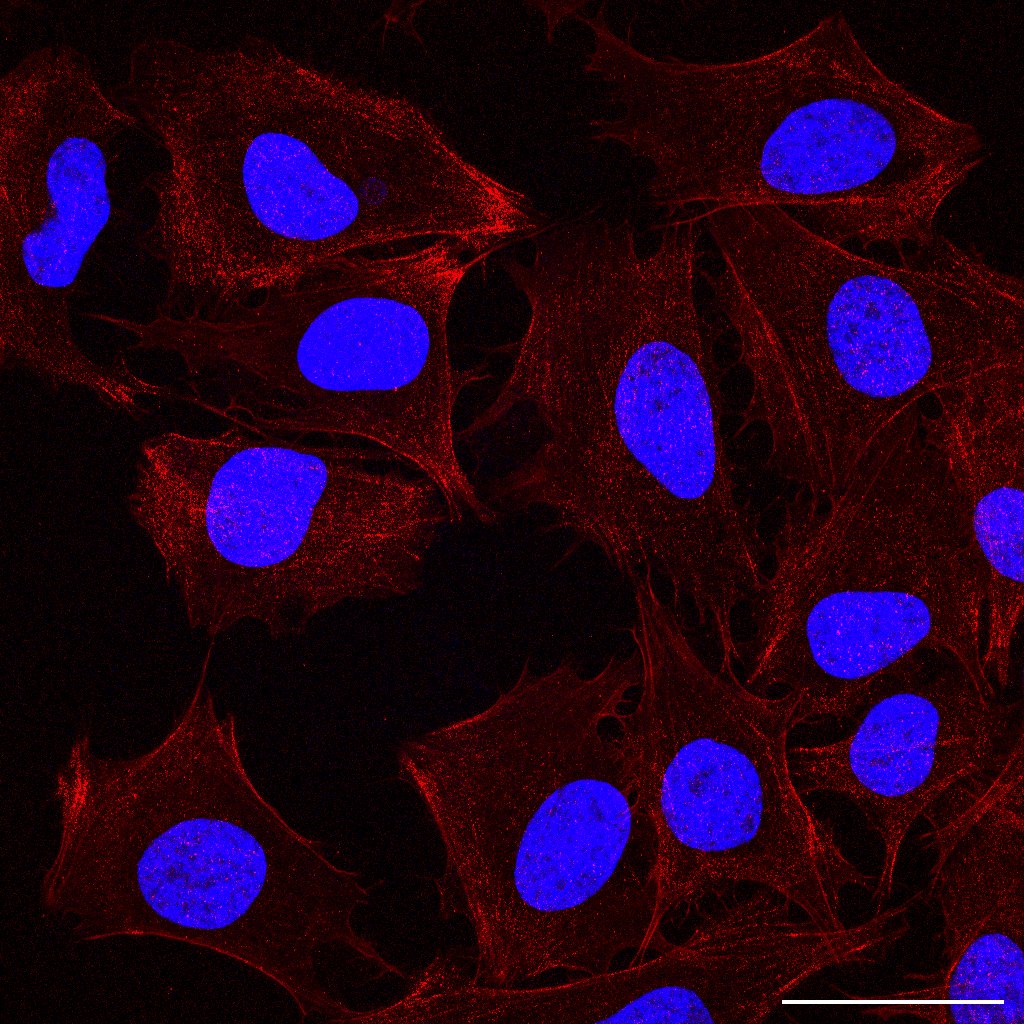

Supplement: Supplementary file 8 — Source data Fig. 5 [file 44318_2026_803_MOESM8_ESM.zip › Fig 5/5C/CPN1 composite.jpg]

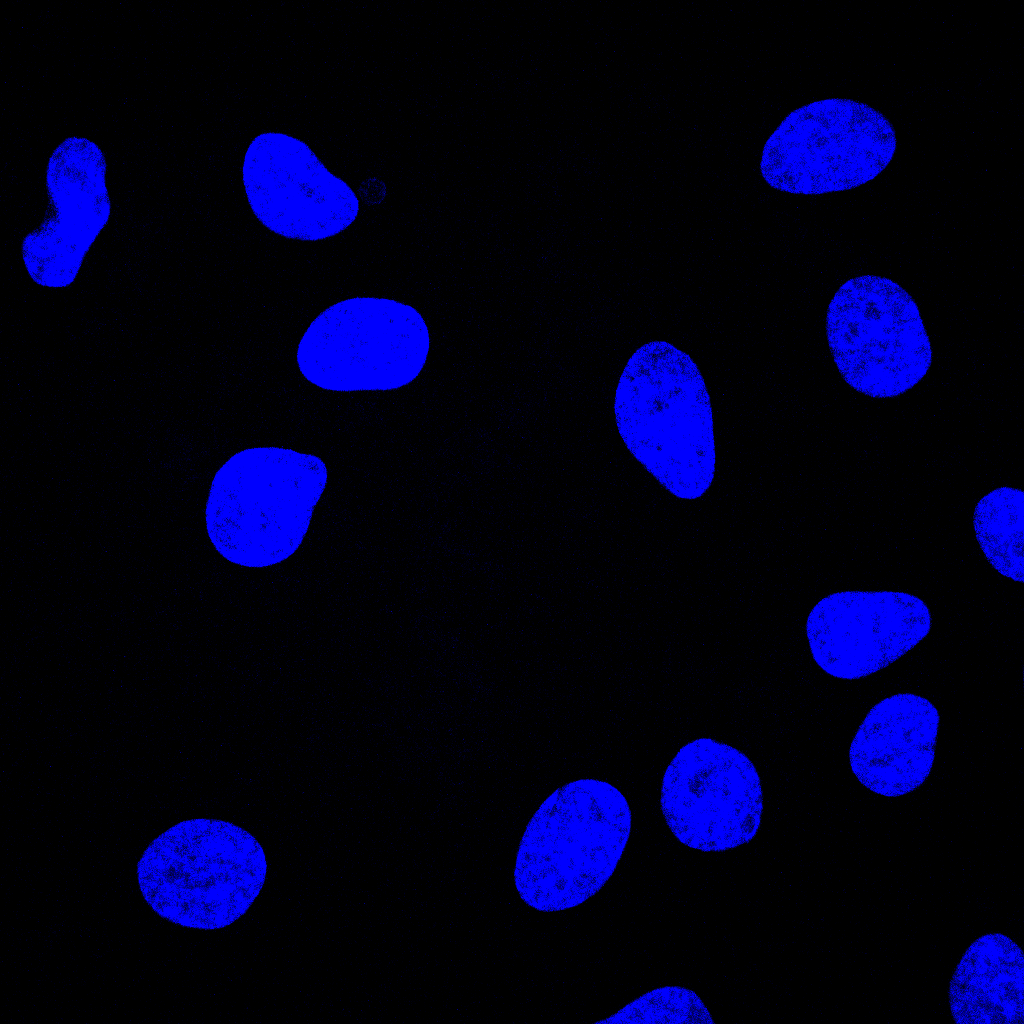

Supplement: Supplementary file 8 — Source data Fig. 5 [file 44318_2026_803_MOESM8_ESM.zip › Fig 5/5C/cpN1_DAPI.tif]

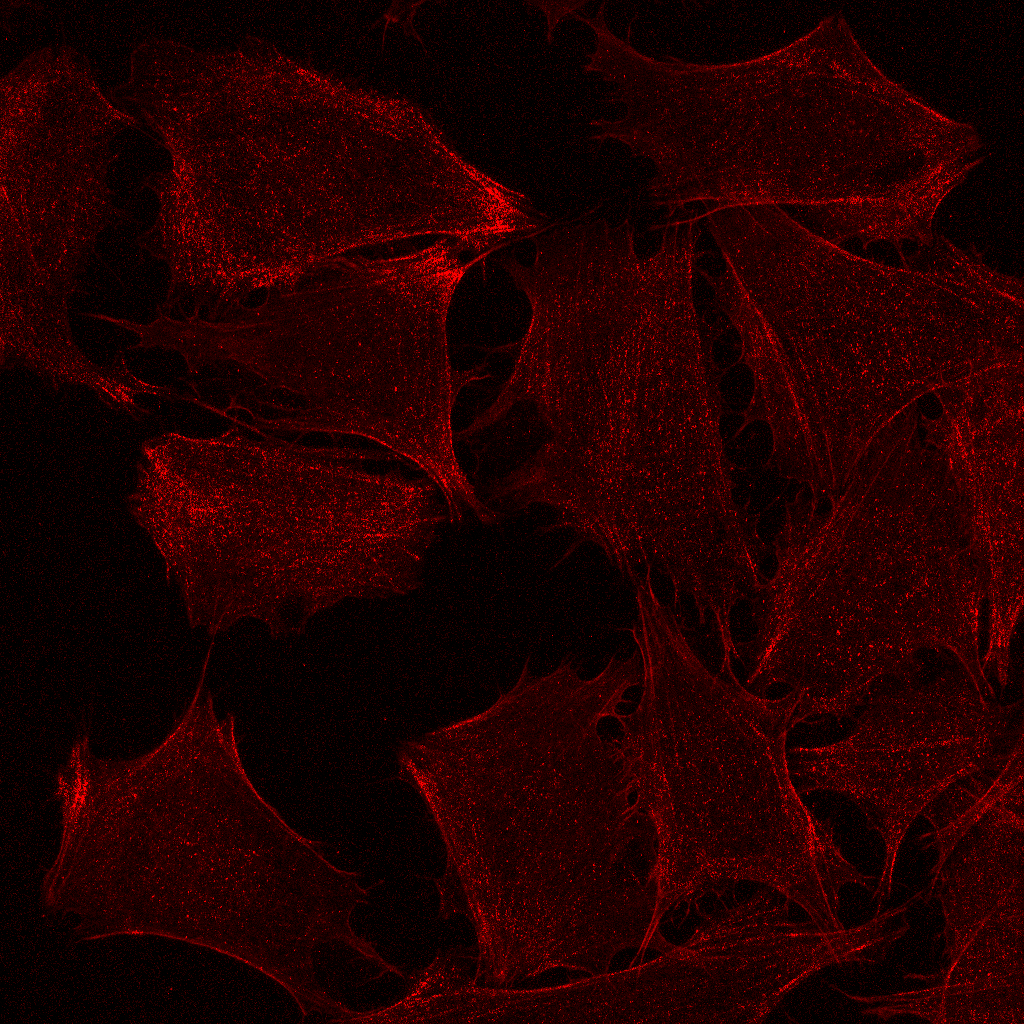

Supplement: Supplementary file 8 — Source data Fig. 5 [file 44318_2026_803_MOESM8_ESM.zip › Fig 5/5C/cpN1_pMLC2.tif]

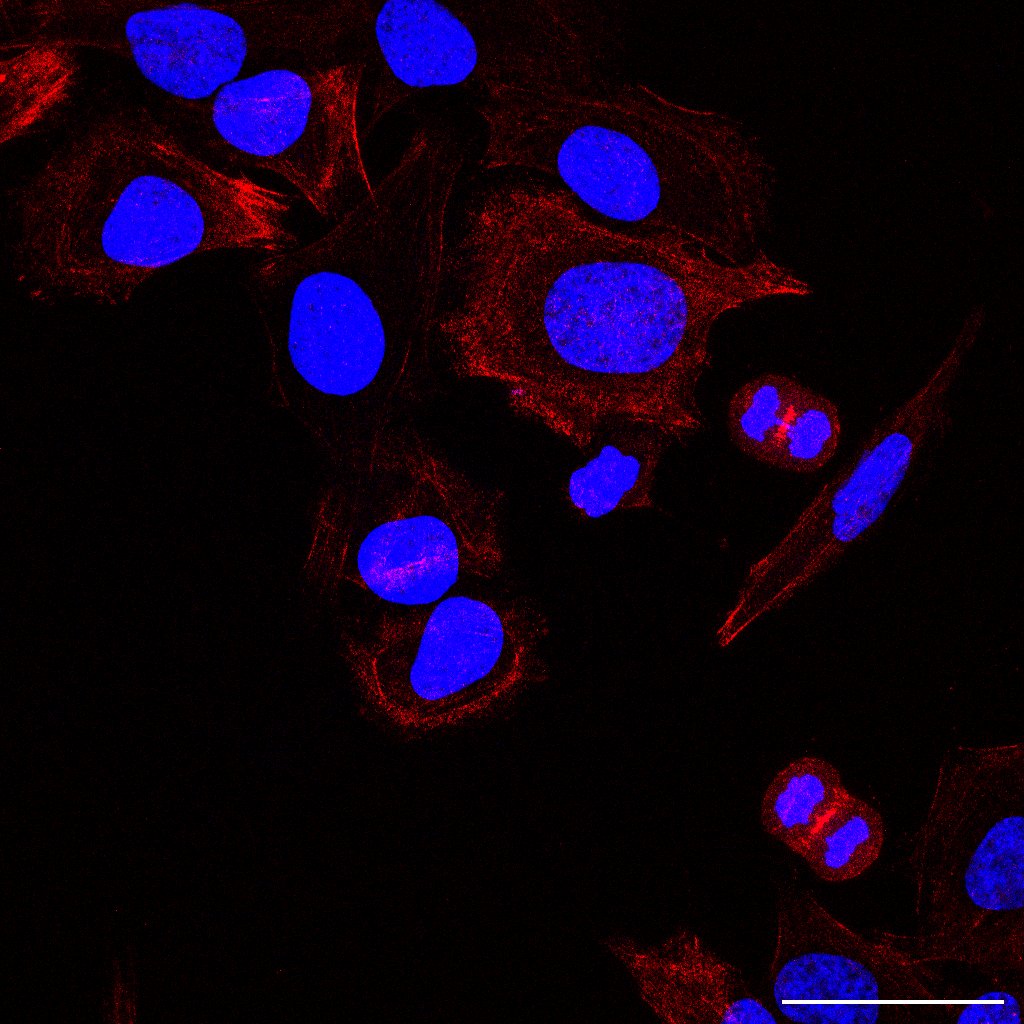

Supplement: Supplementary file 8 — Source data Fig. 5 [file 44318_2026_803_MOESM8_ESM.zip › Fig 5/5C/cpN2 composite.jpg]

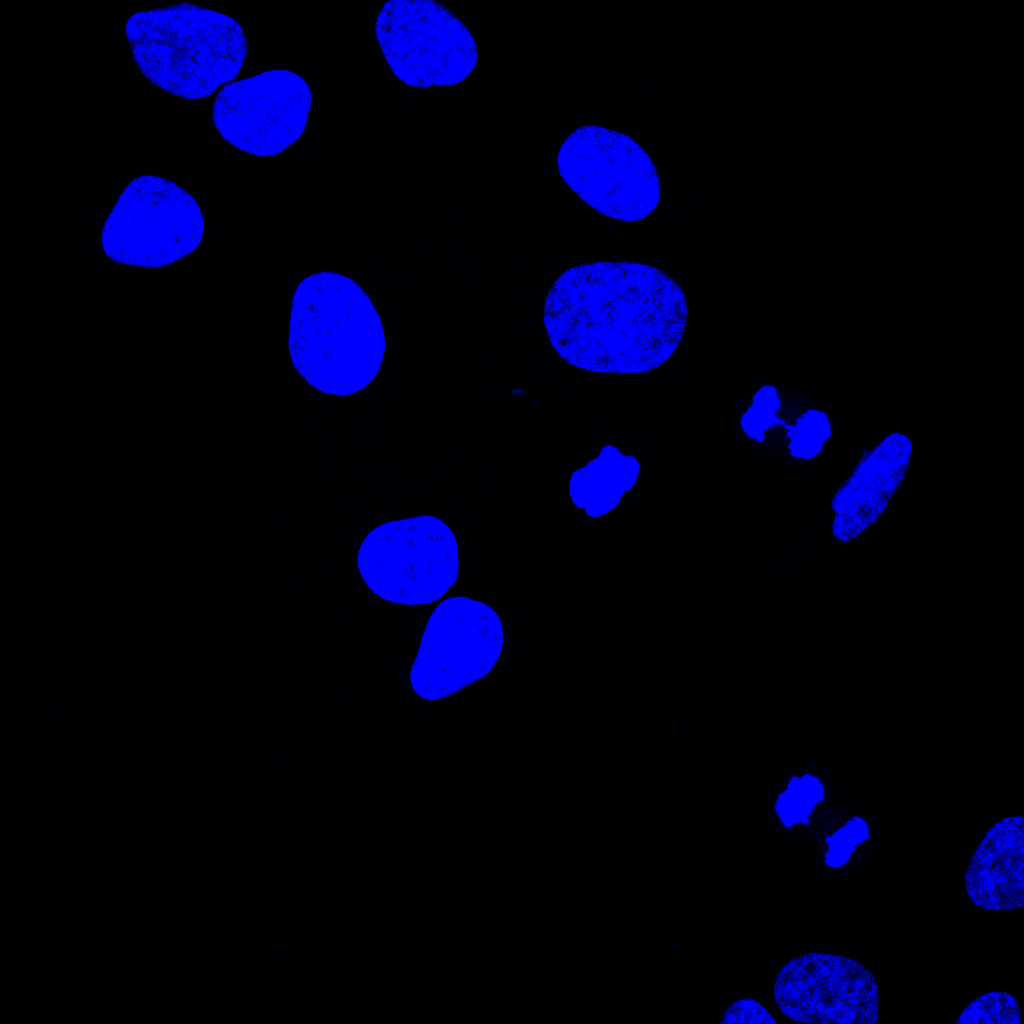

Supplement: Supplementary file 8 — Source data Fig. 5 [file 44318_2026_803_MOESM8_ESM.zip › Fig 5/5C/cpN2_DAPI.tif]

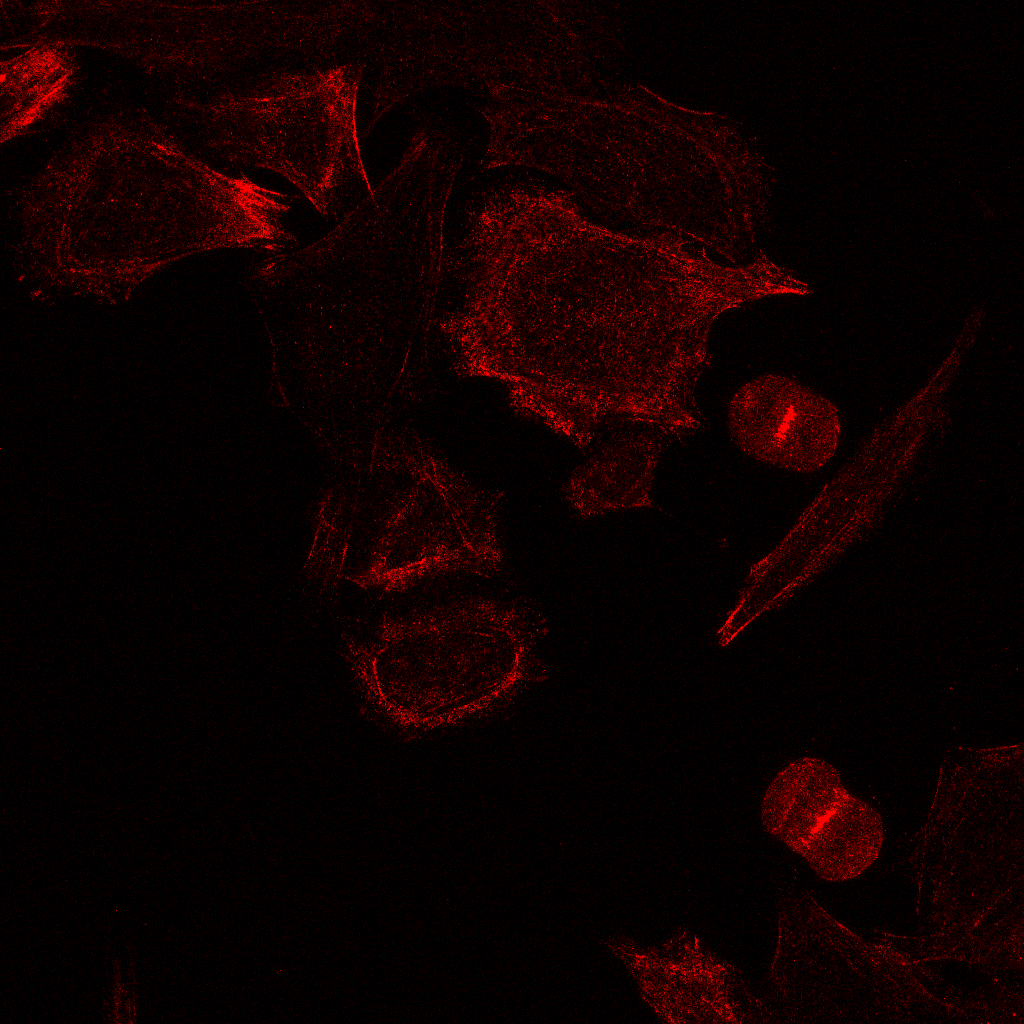

Supplement: Supplementary file 8 — Source data Fig. 5 [file 44318_2026_803_MOESM8_ESM.zip › Fig 5/5C/cpN2_pMLC2.tif]

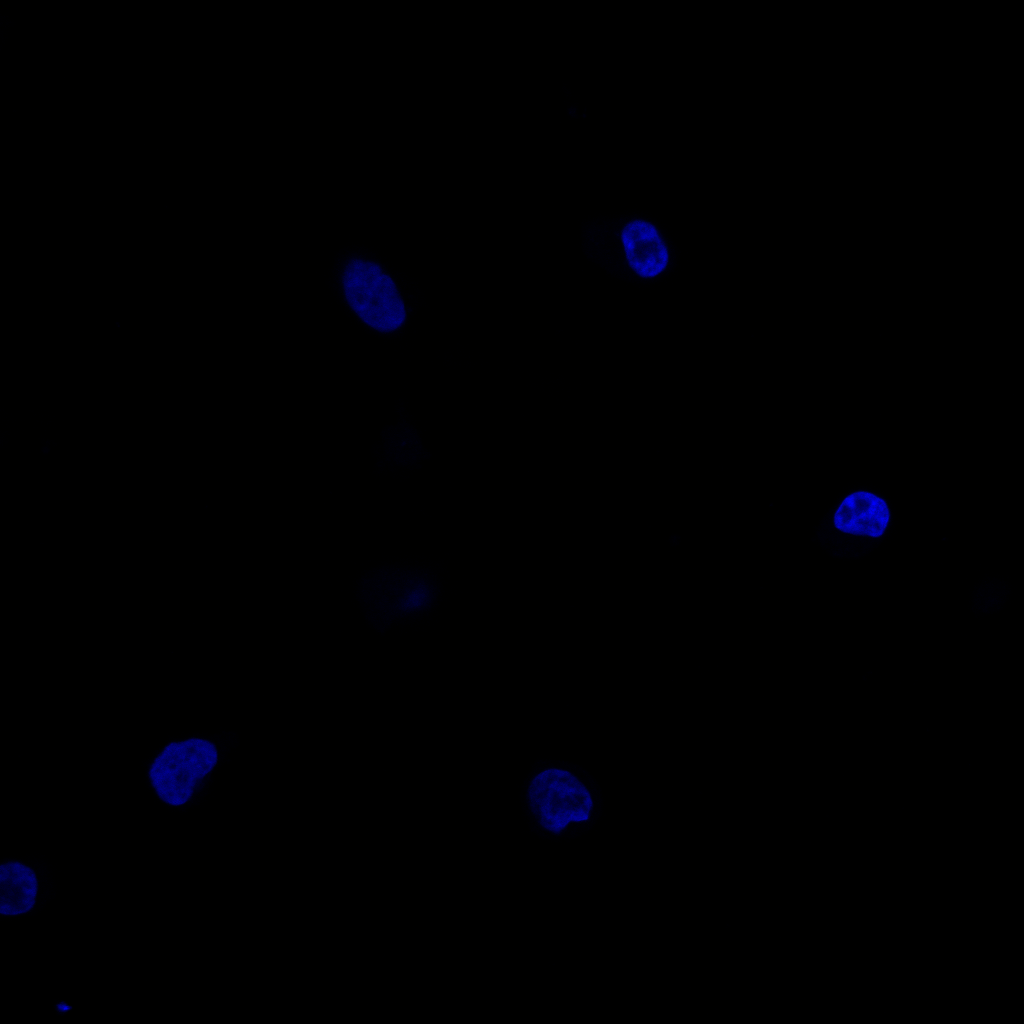

Supplement: Supplementary file 8 — Source data Fig. 5 [file 44318_2026_803_MOESM8_ESM.zip › Fig 5/5E/C1-40x_sk103_control3_Dapi.tif]

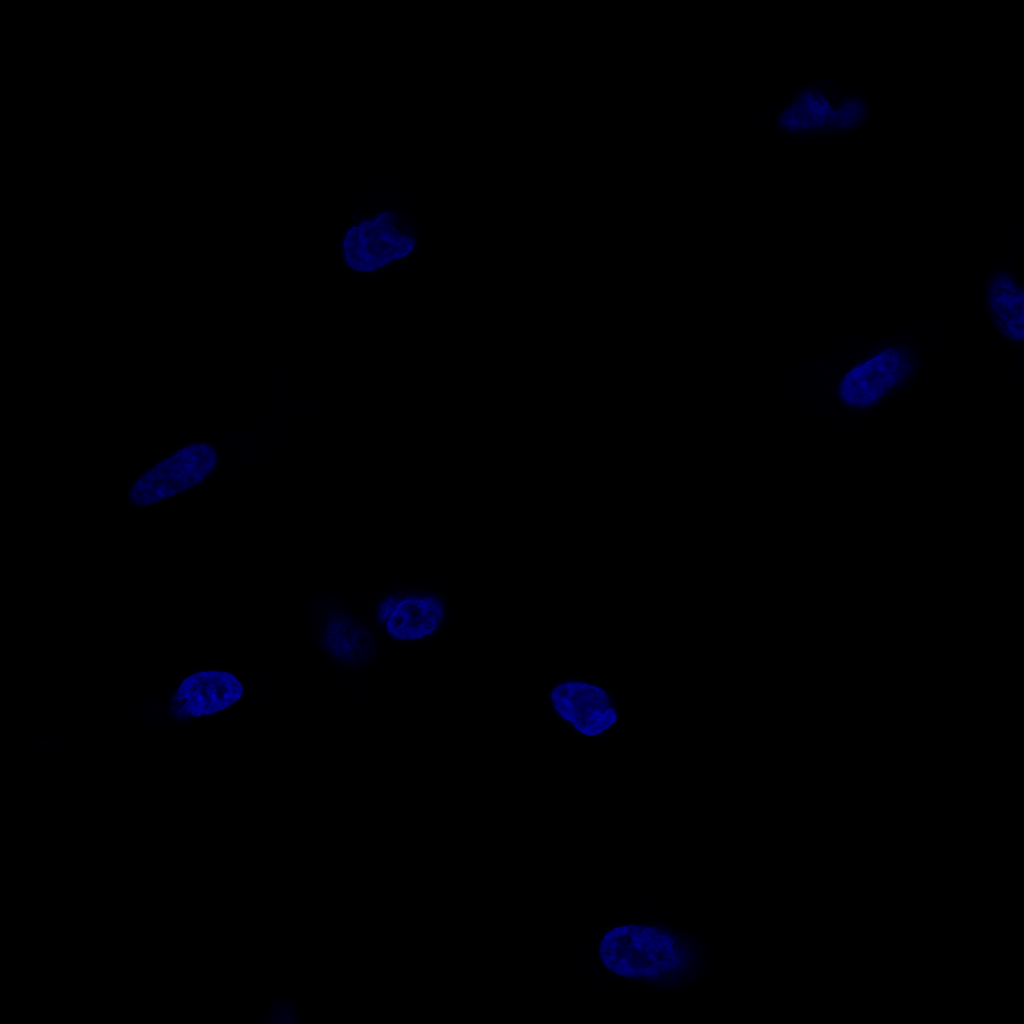

Supplement: Supplementary file 8 — Source data Fig. 5 [file 44318_2026_803_MOESM8_ESM.zip › Fig 5/5E/C1-40x_sk103_ngfr1_1_Dapi.tif]

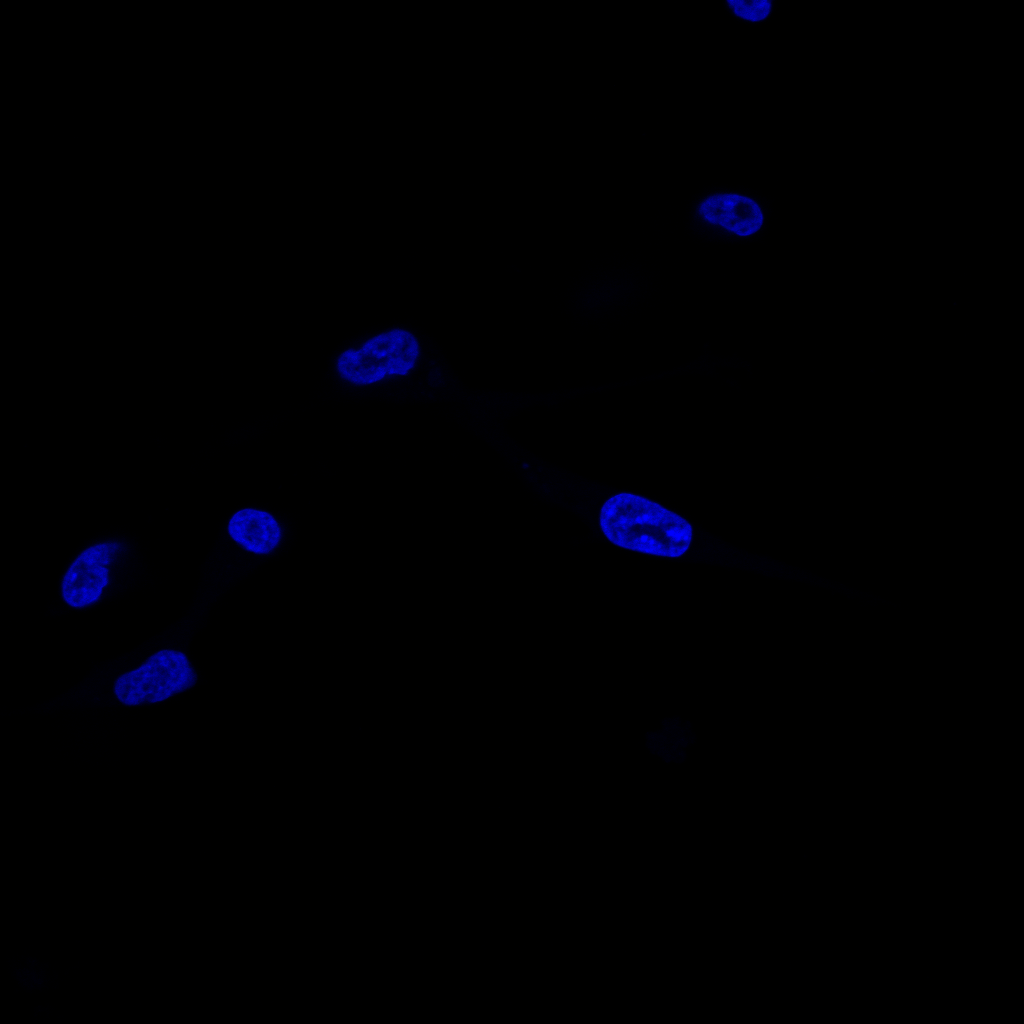

Supplement: Supplementary file 8 — Source data Fig. 5 [file 44318_2026_803_MOESM8_ESM.zip › Fig 5/5E/C1-40x_sk103_ngfr2_3_Dapi.tif]

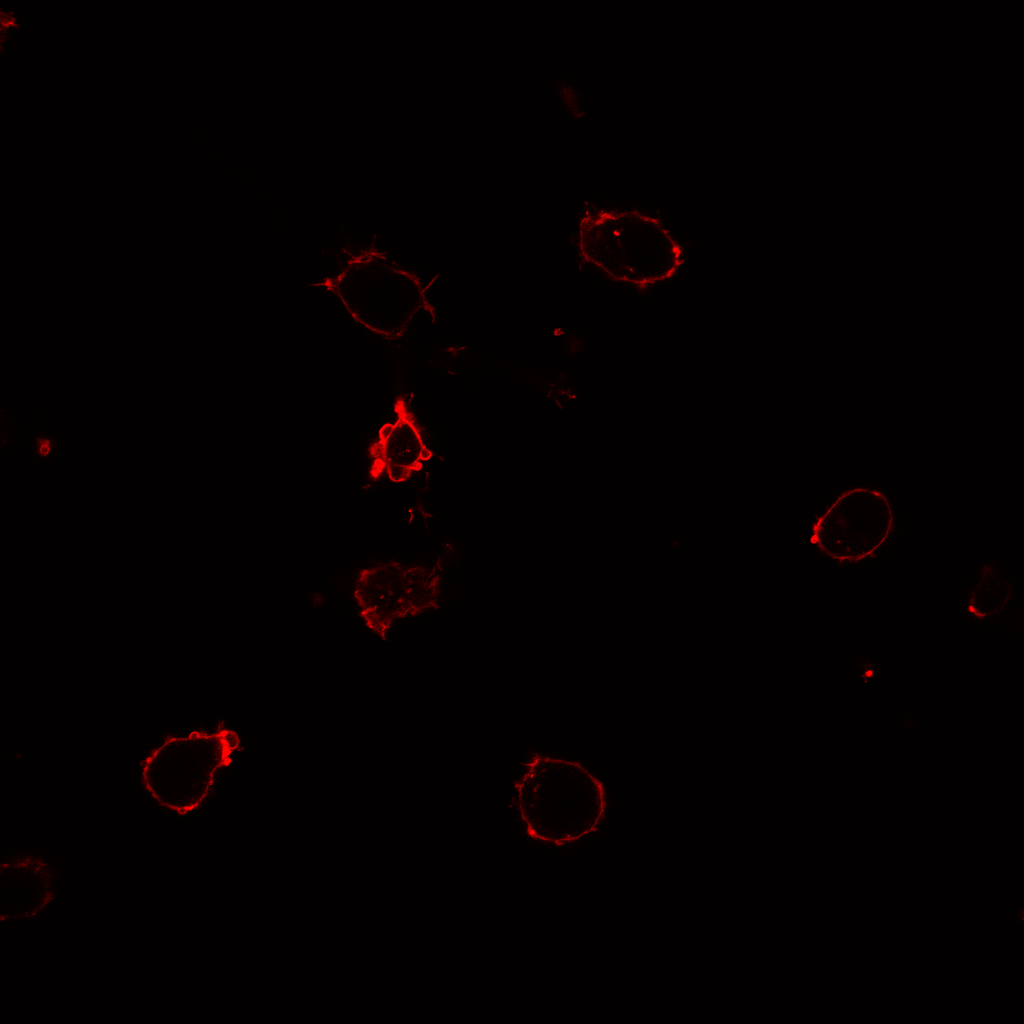

Supplement: Supplementary file 8 — Source data Fig. 5 [file 44318_2026_803_MOESM8_ESM.zip › Fig 5/5E/C3-40x_sk103_control3_actin.tif]

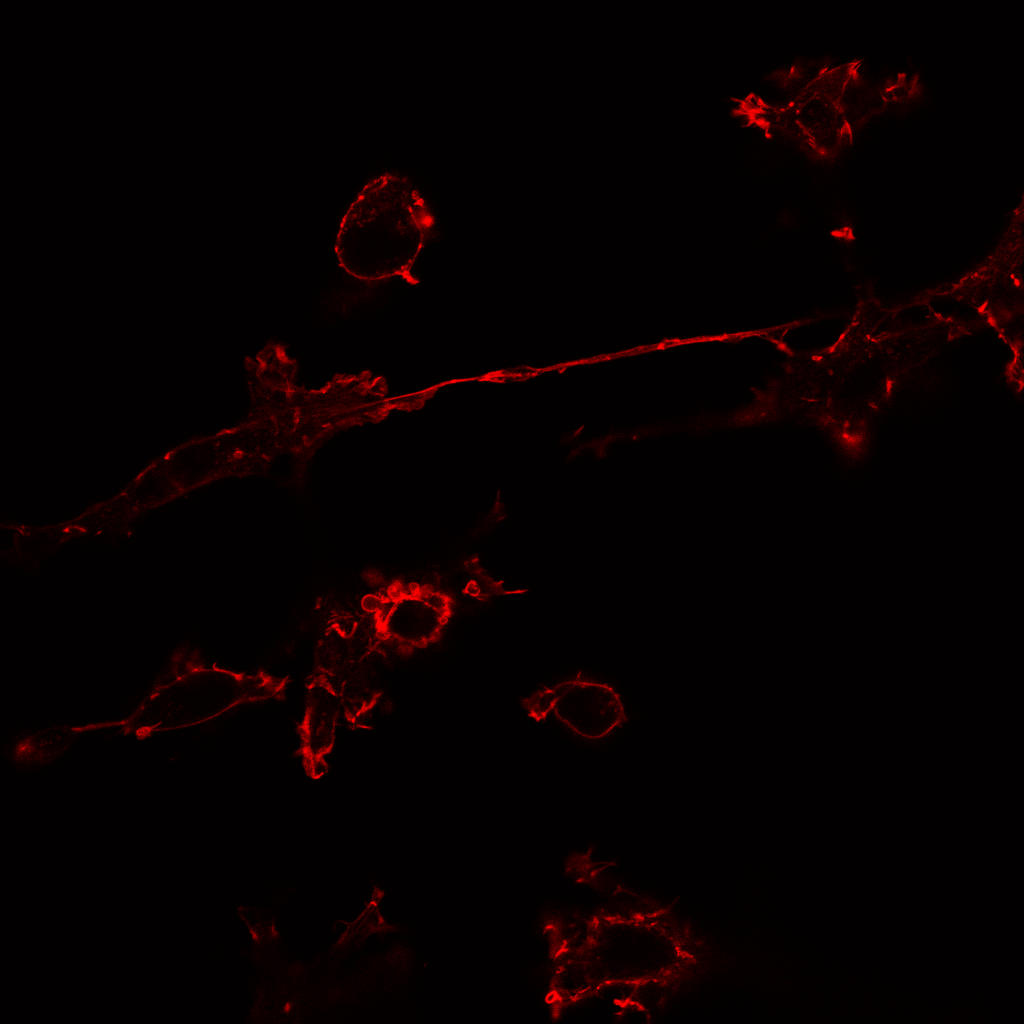

Supplement: Supplementary file 8 — Source data Fig. 5 [file 44318_2026_803_MOESM8_ESM.zip › Fig 5/5E/C3-40x_sk103_ngfr1_1_actin.tif]

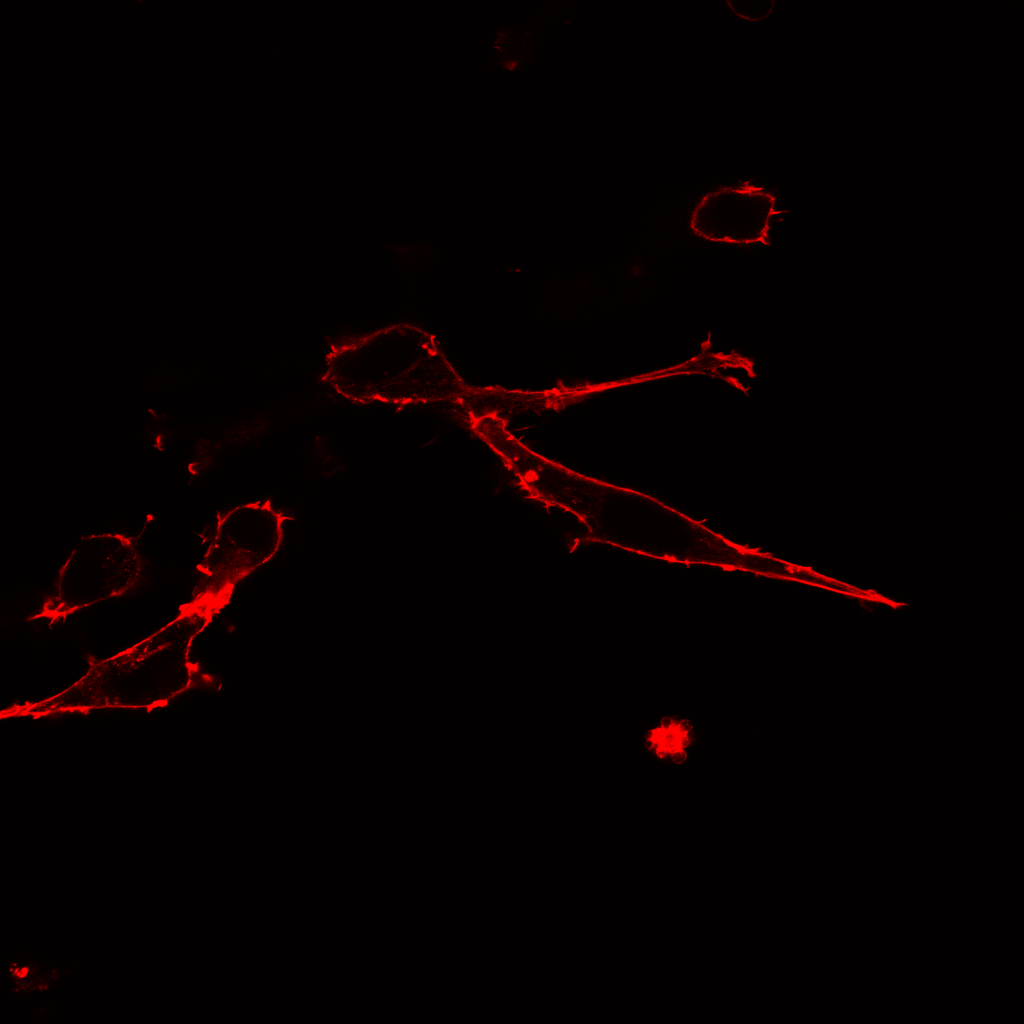

Supplement: Supplementary file 8 — Source data Fig. 5 [file 44318_2026_803_MOESM8_ESM.zip › Fig 5/5E/C3-40x_sk103_ngfr2_3_actin.tif]

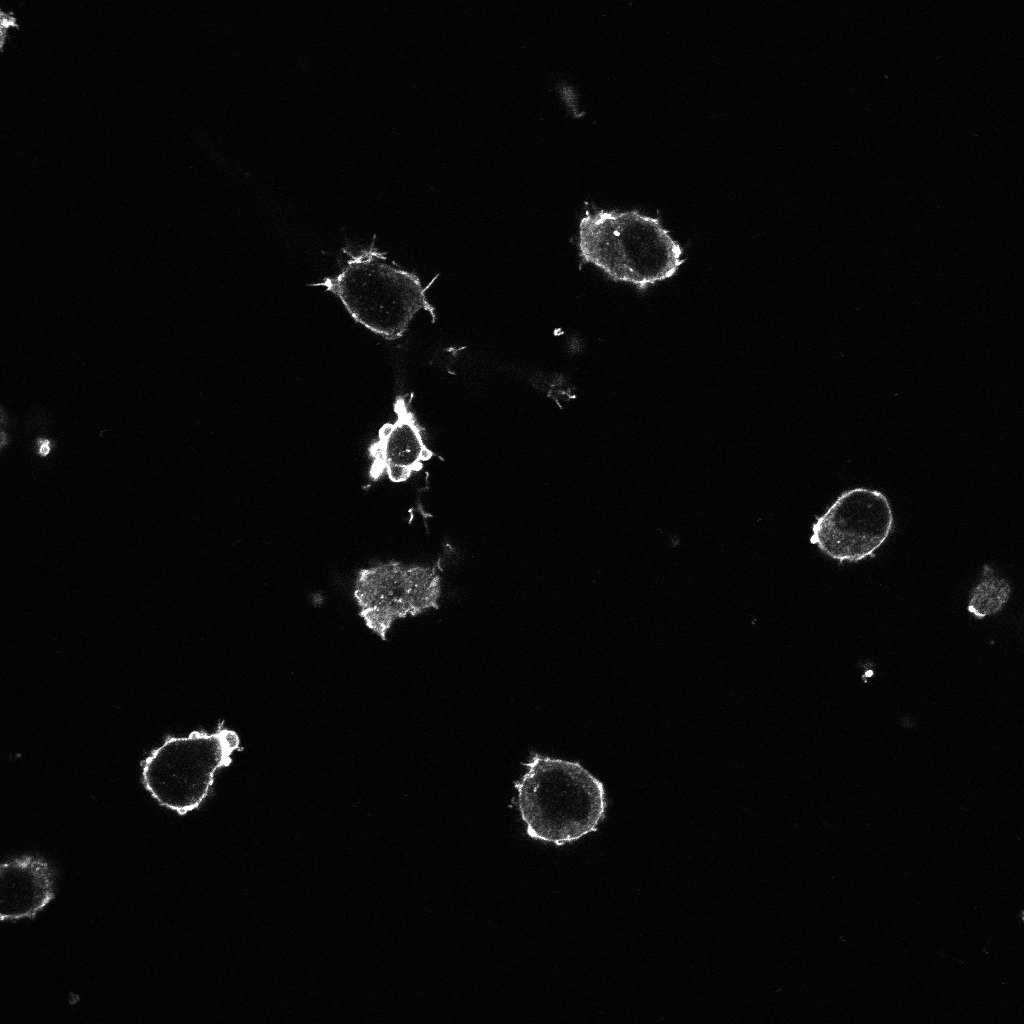

Supplement: Supplementary file 8 — Source data Fig. 5 [file 44318_2026_803_MOESM8_ESM.zip › Fig 5/5E/Composite-SKMEL-CpC.tif]
