# Supplementary figures and images for: NGFR induces melanoma invasion and immunotherapy resistance through myosin light chain 2 modulation (part 2 of 3)
Source: EMBO J. 2026 May 26;45(14):4988–5023. doi: 10.1038/s44318-026-00803-2 (PMC13373201; doi:10.1038/s44318-026-00803-2)

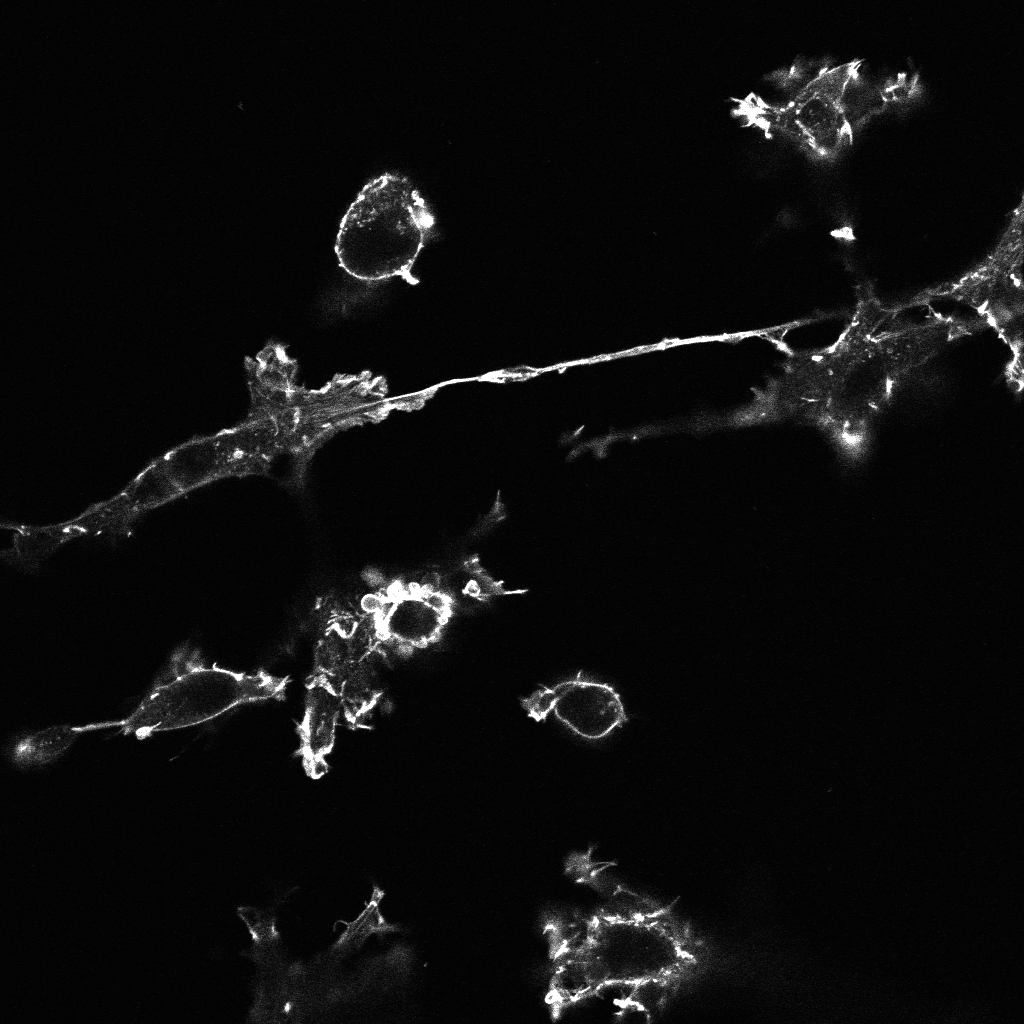

Supplement: Supplementary file 8 — Source data Fig. 5 [file 44318_2026_803_MOESM8_ESM.zip › Fig 5/5E/Composite-SKMEL-CpNGFR1.tif]

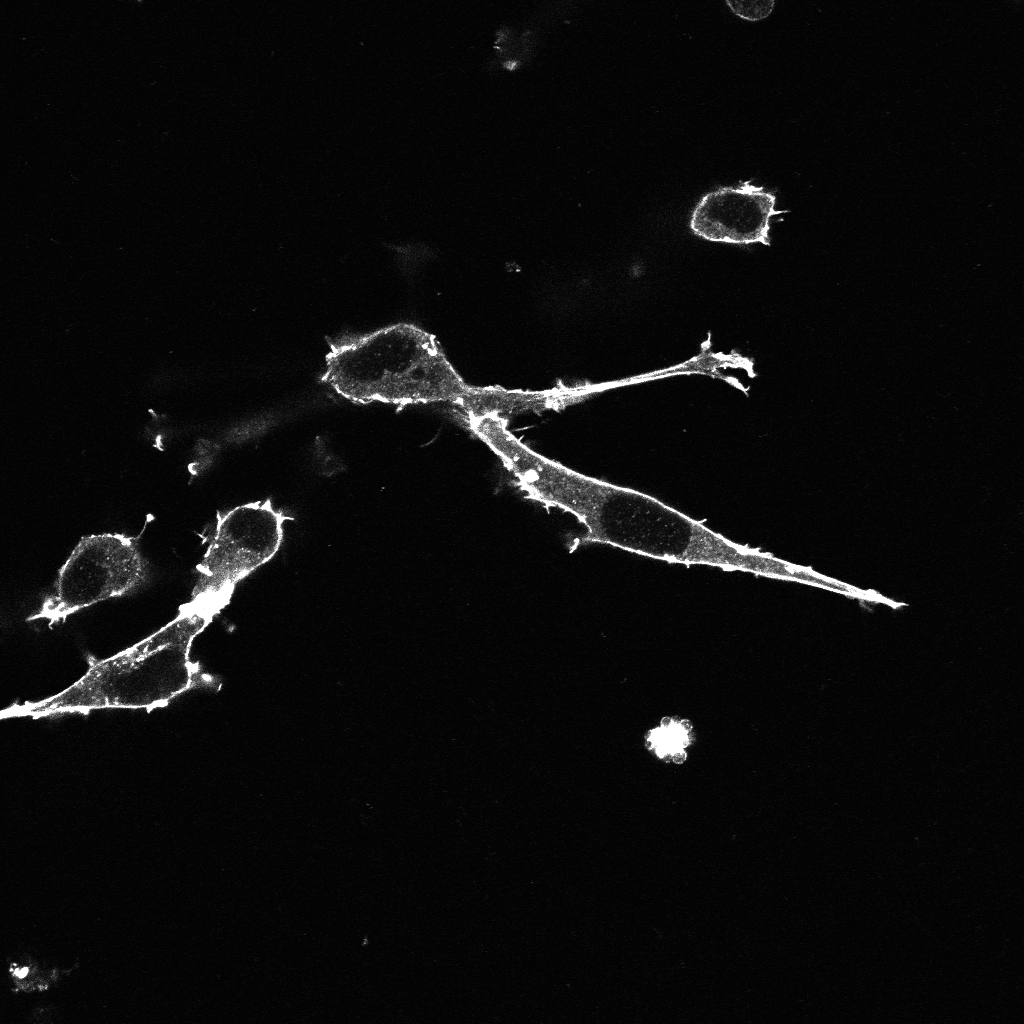

Supplement: Supplementary file 8 — Source data Fig. 5 [file 44318_2026_803_MOESM8_ESM.zip › Fig 5/5E/Composite_SKMEL_cpNGFR2.tif]

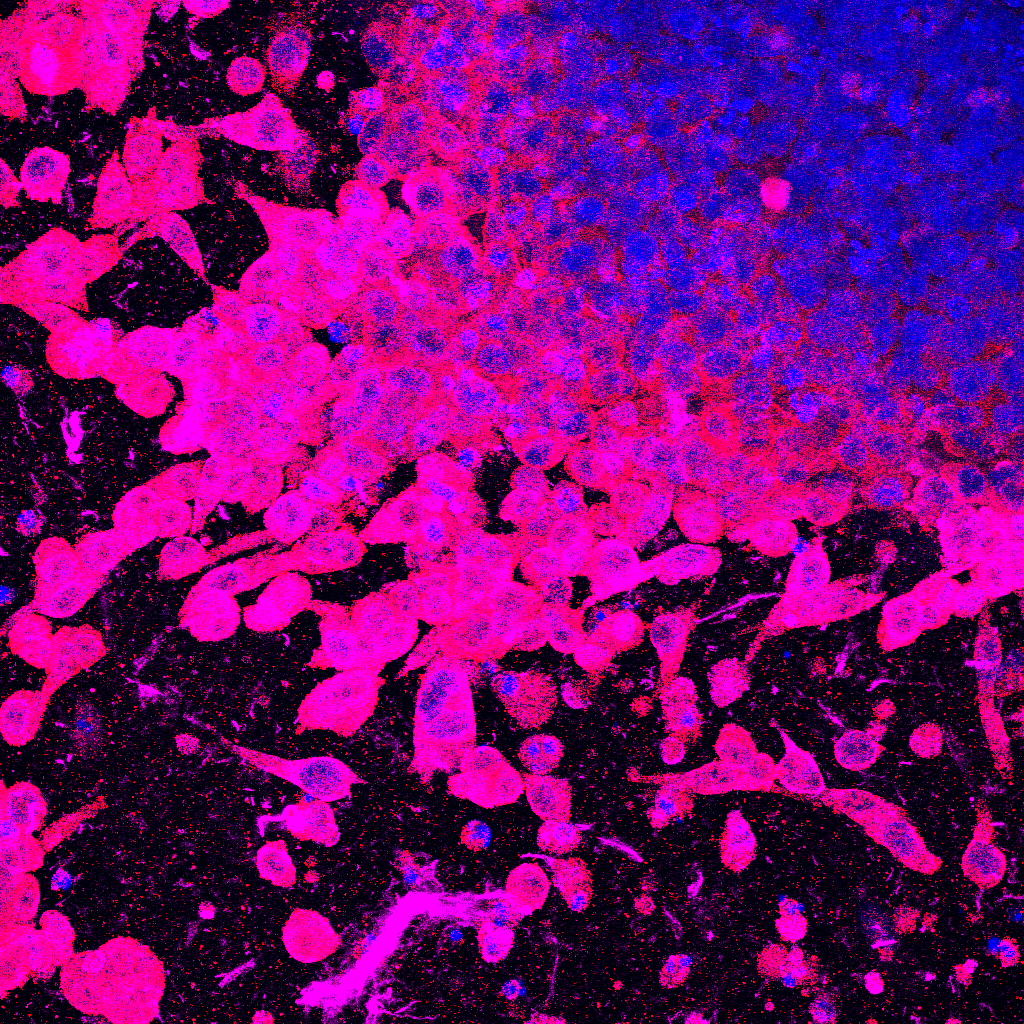

Supplement: Supplementary file 8 — Source data Fig. 5 [file 44318_2026_803_MOESM8_ESM.zip › Fig 5/5H/Composite_cpC.tif]

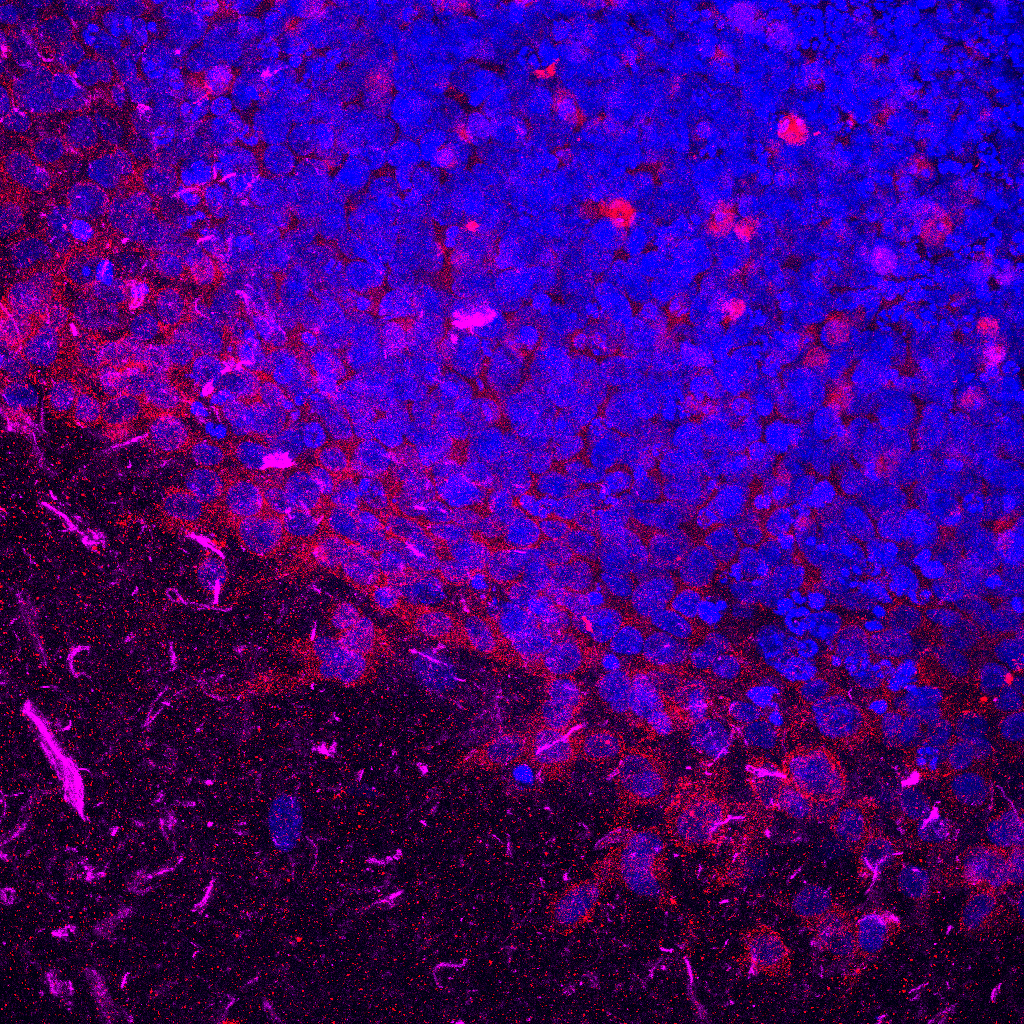

Supplement: Supplementary file 8 — Source data Fig. 5 [file 44318_2026_803_MOESM8_ESM.zip › Fig 5/5H/Composite_CPN1.tif]

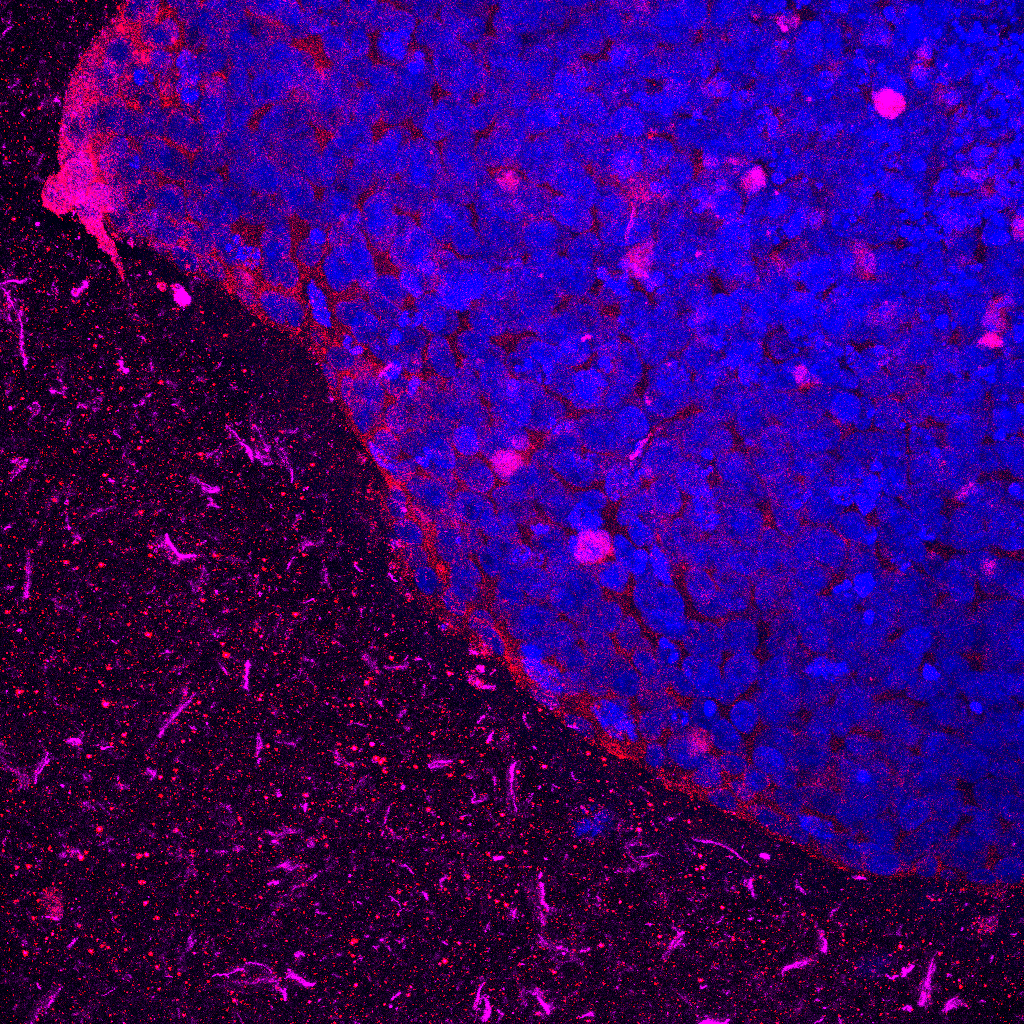

Supplement: Supplementary file 8 — Source data Fig. 5 [file 44318_2026_803_MOESM8_ESM.zip › Fig 5/5H/Composite_CPN2.tif]

j)

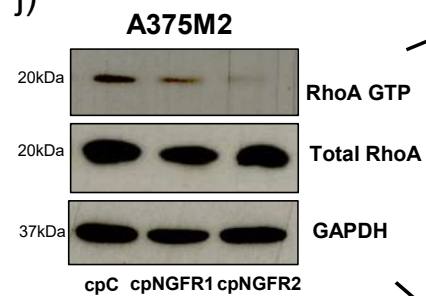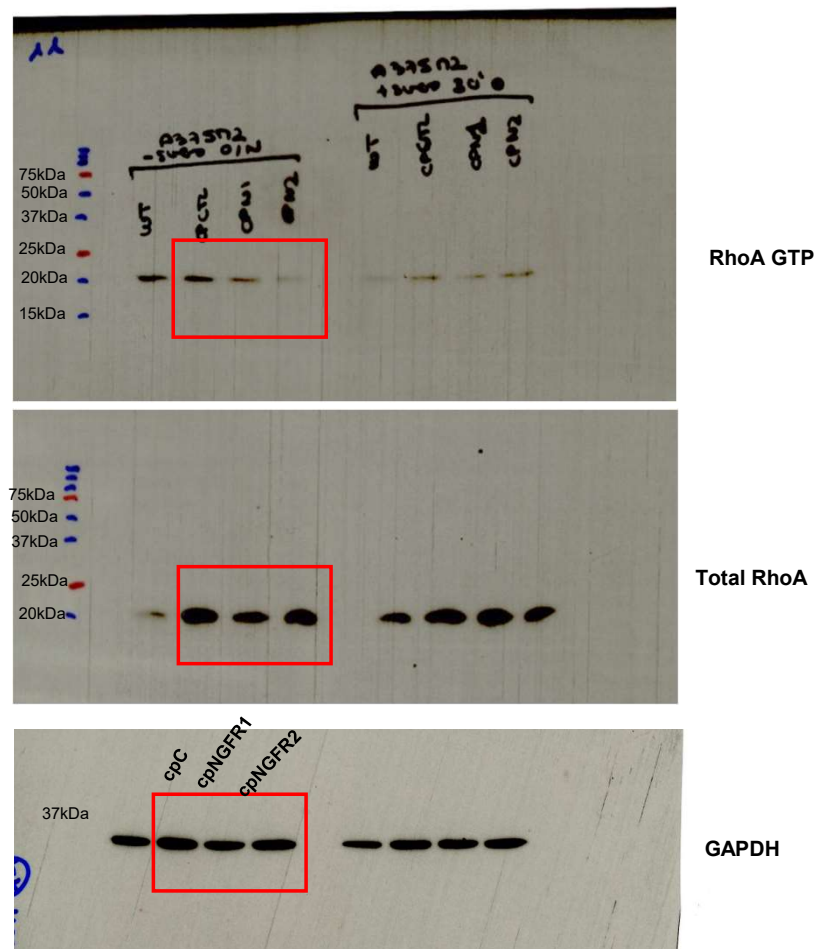

Supplement: Supplementary file 8 — Source data Fig. 5 [file 44318_2026_803_MOESM8_ESM.zip › Fig 5/5J/5J-Readme.pdf]

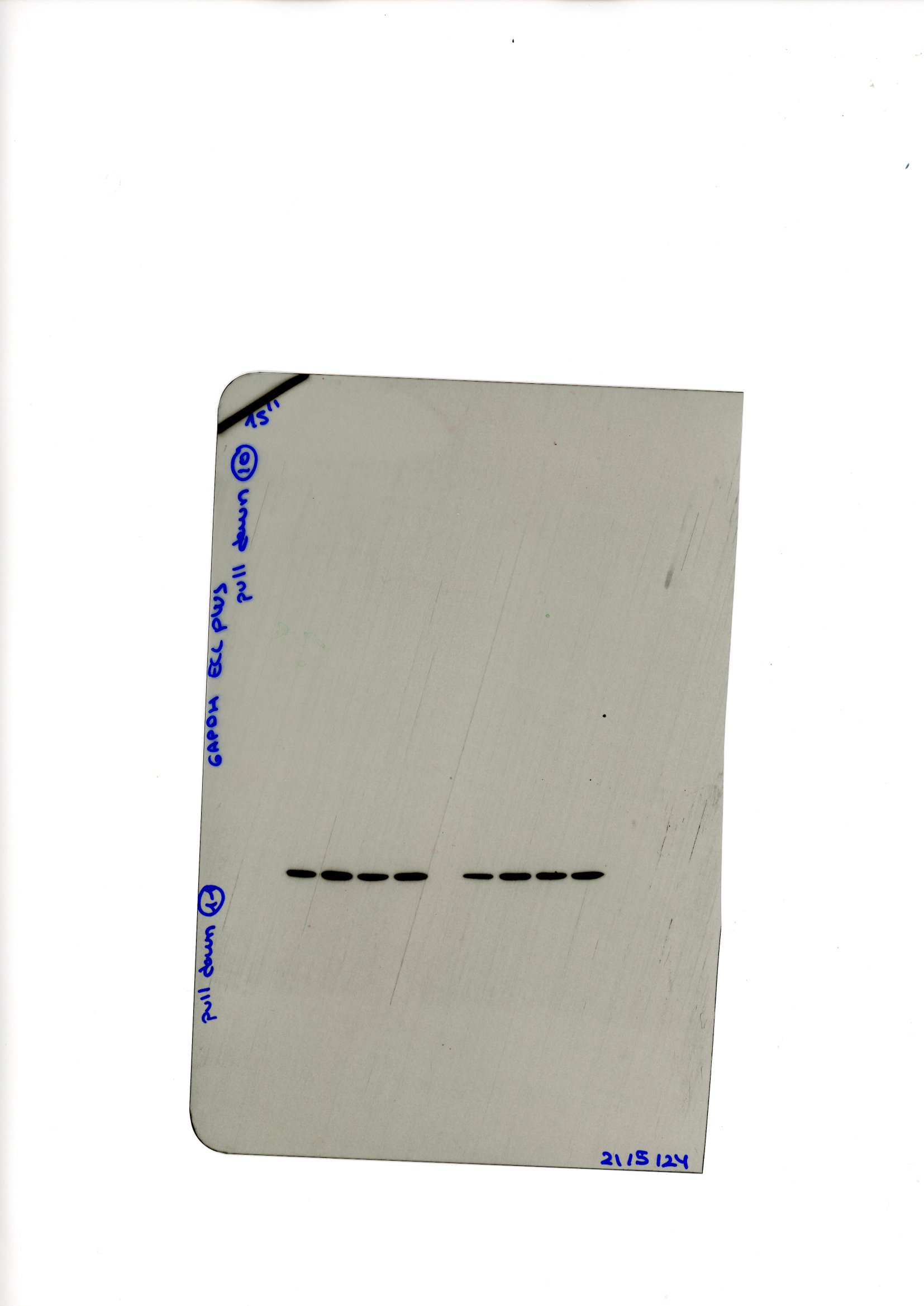

Supplement: Supplementary file 8 — Source data Fig. 5 [file 44318_2026_803_MOESM8_ESM.zip › Fig 5/5J/GAPDH.jpg]

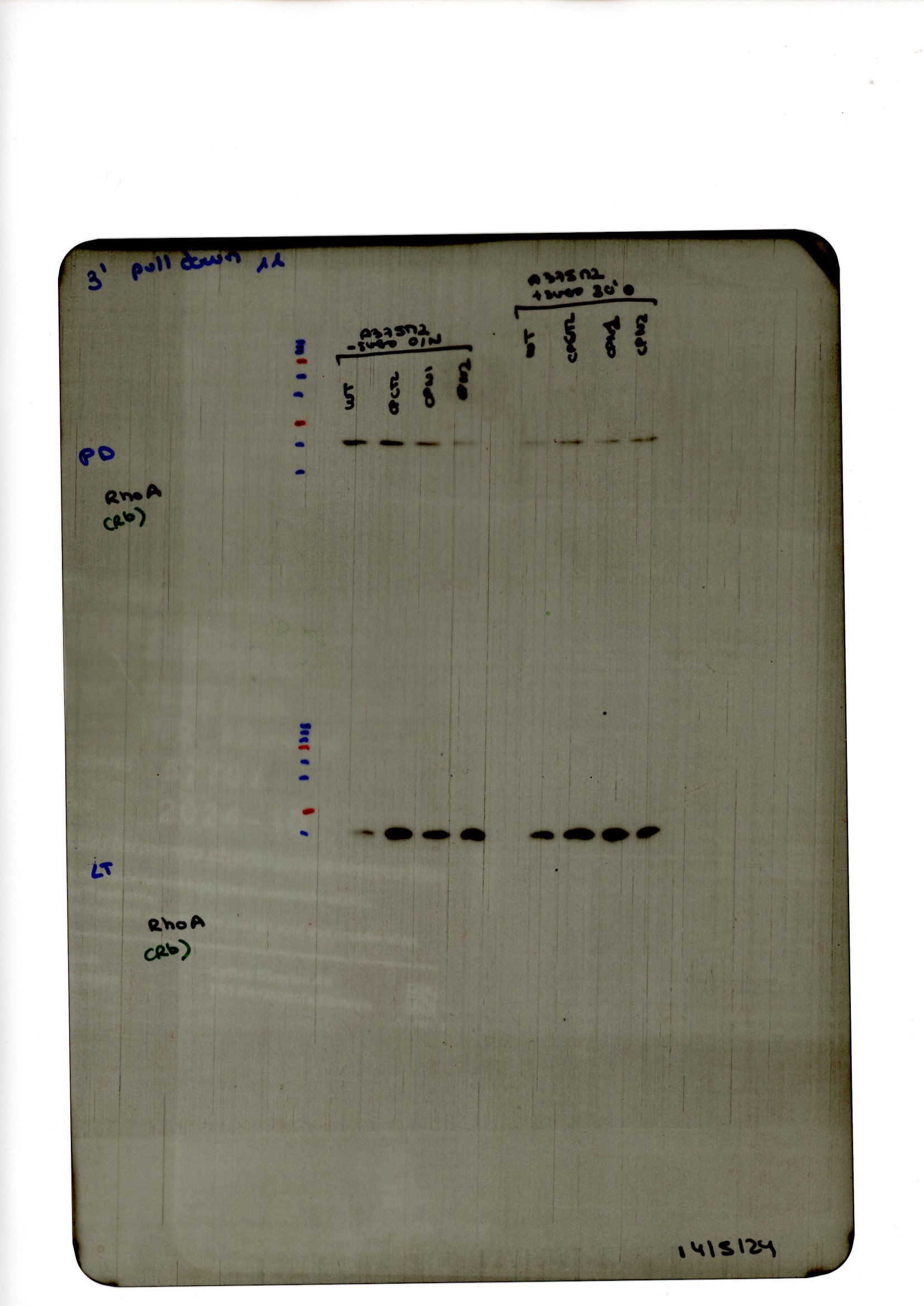

Supplement: Supplementary file 8 — Source data Fig. 5 [file 44318_2026_803_MOESM8_ESM.zip › Fig 5/5J/RhoA PullDown up and Lysates Down.jpg]

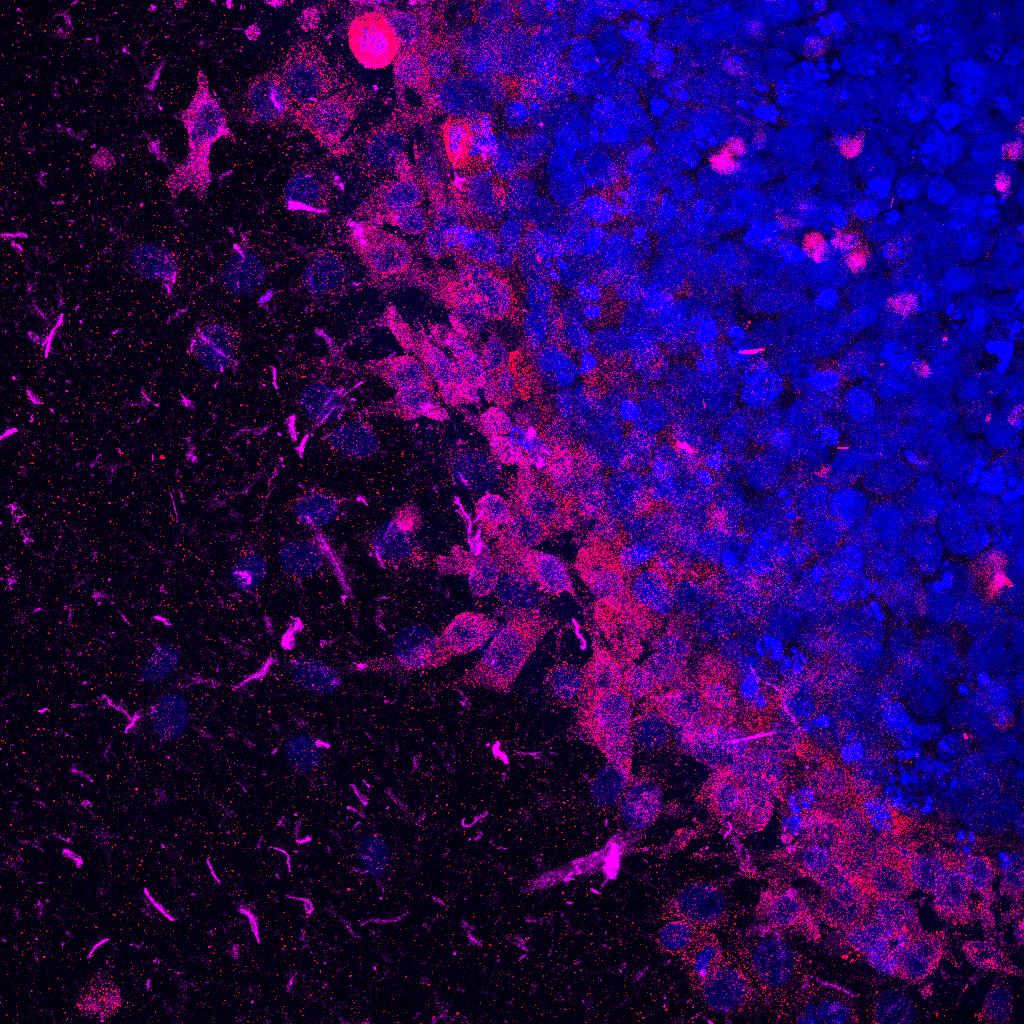

Supplement: Supplementary file 9 — Source data Fig. 6 [file 44318_2026_803_MOESM9_ESM.zip › Fig 6/6E/Composite A375P cpC ROCKi.tif]

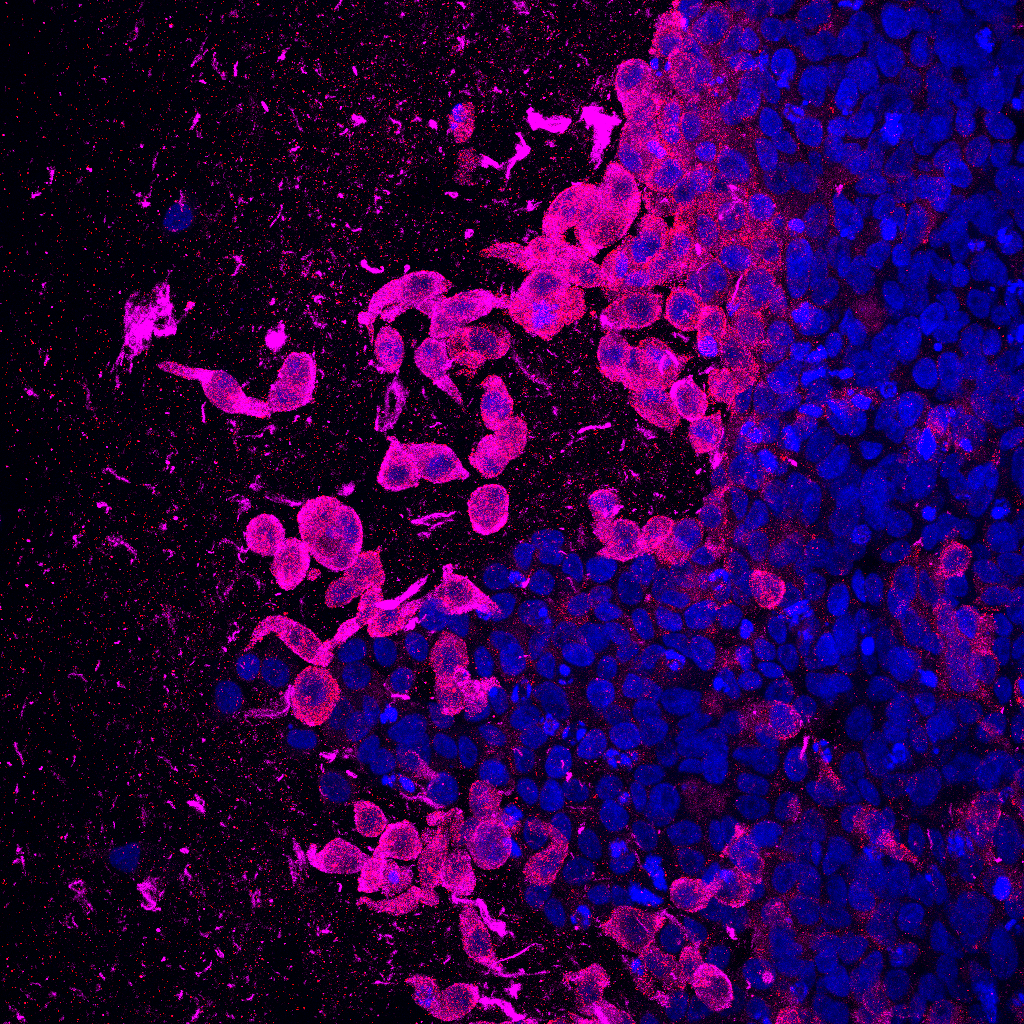

Supplement: Supplementary file 9 — Source data Fig. 6 [file 44318_2026_803_MOESM9_ESM.zip › Fig 6/6E/Composite_A375P_cpC Vehicle.tif]

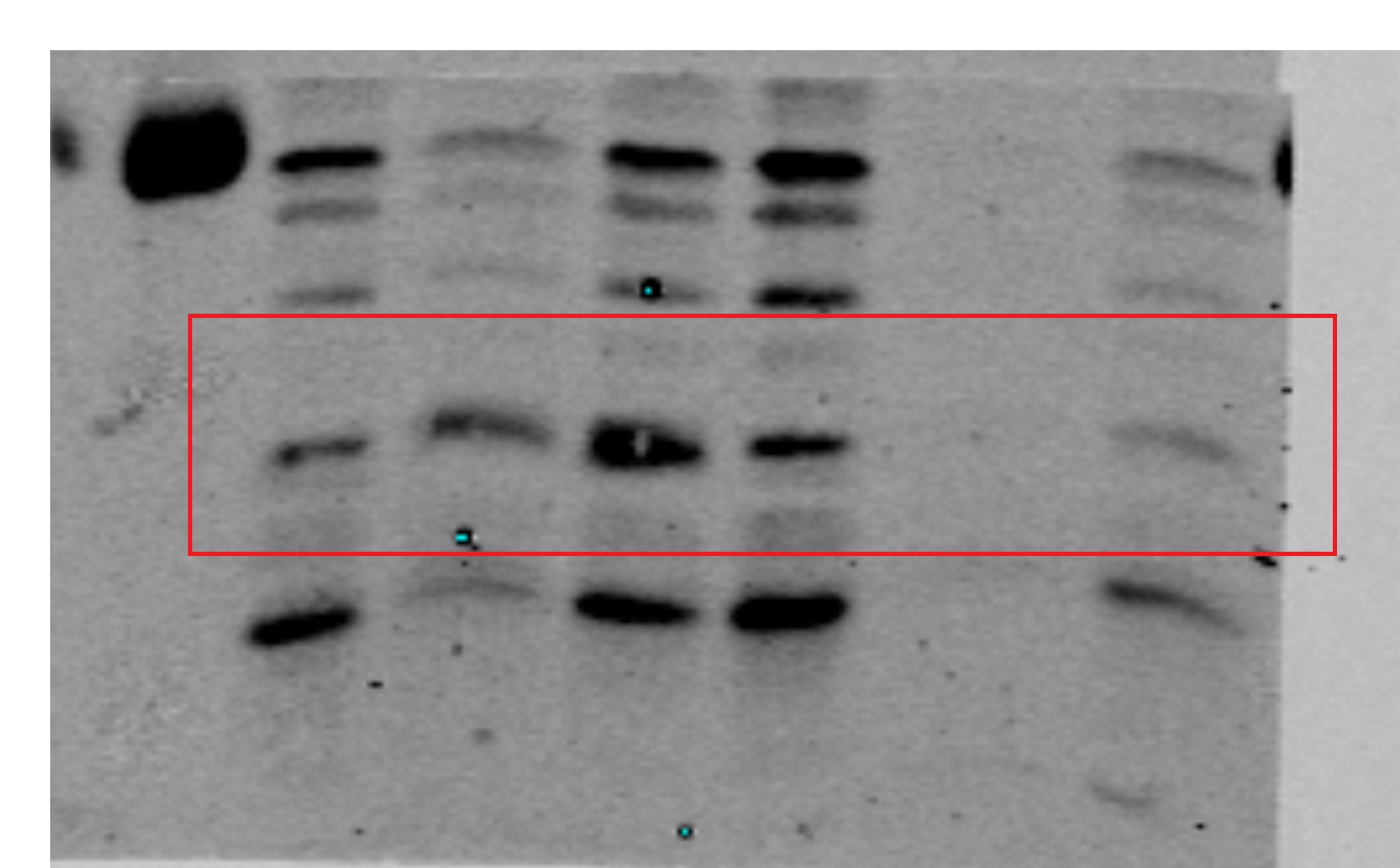

Supplement: Supplementary file 9 — Source data Fig. 6 [file 44318_2026_803_MOESM9_ESM.zip › Fig 6/6H/220311 MLC2 total.tif]

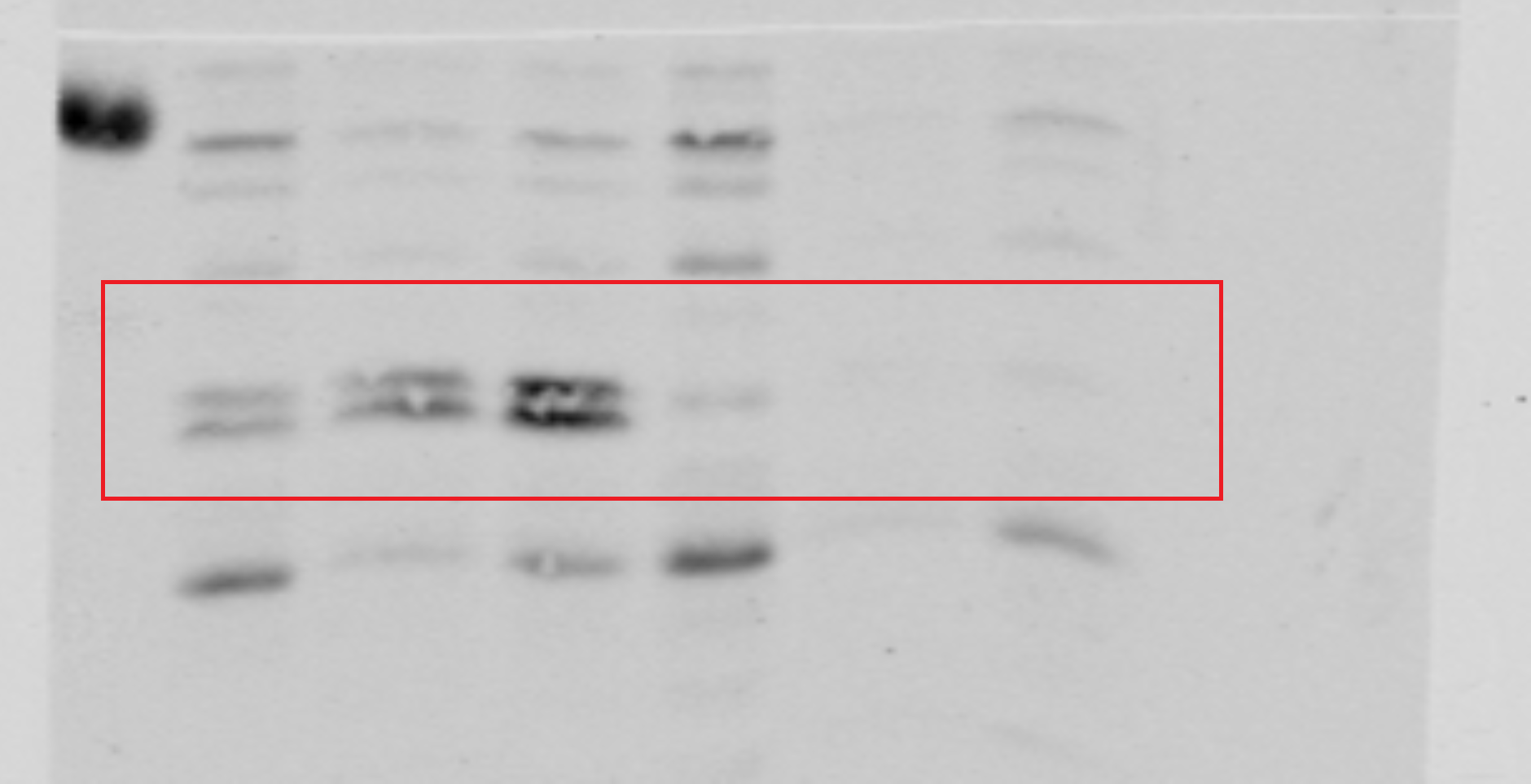

Supplement: Supplementary file 9 — Source data Fig. 6 [file 44318_2026_803_MOESM9_ESM.zip › Fig 6/6H/220311 pMLC2.tif]

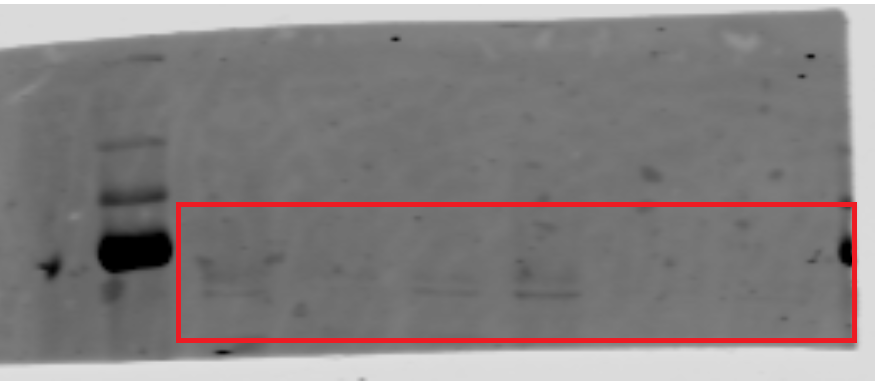

Supplement: Supplementary file 9 — Source data Fig. 6 [file 44318_2026_803_MOESM9_ESM.zip › Fig 6/6H/220314 NGFR.tif]

# A375M2 - ROCKi

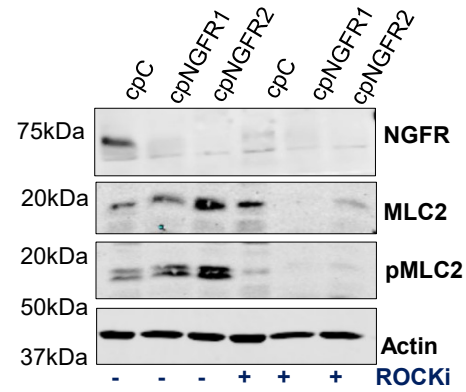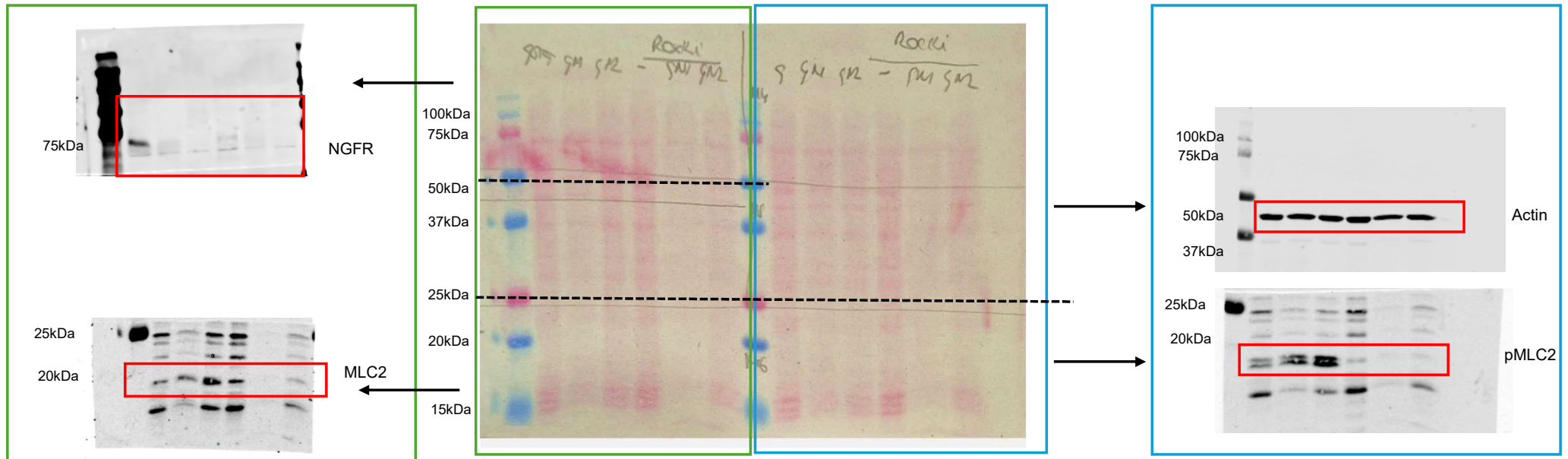

Supplement: Supplementary file 9 — Source data Fig. 6 [file 44318_2026_803_MOESM9_ESM.zip › Fig 6/6H/6H-Readme.pdf]

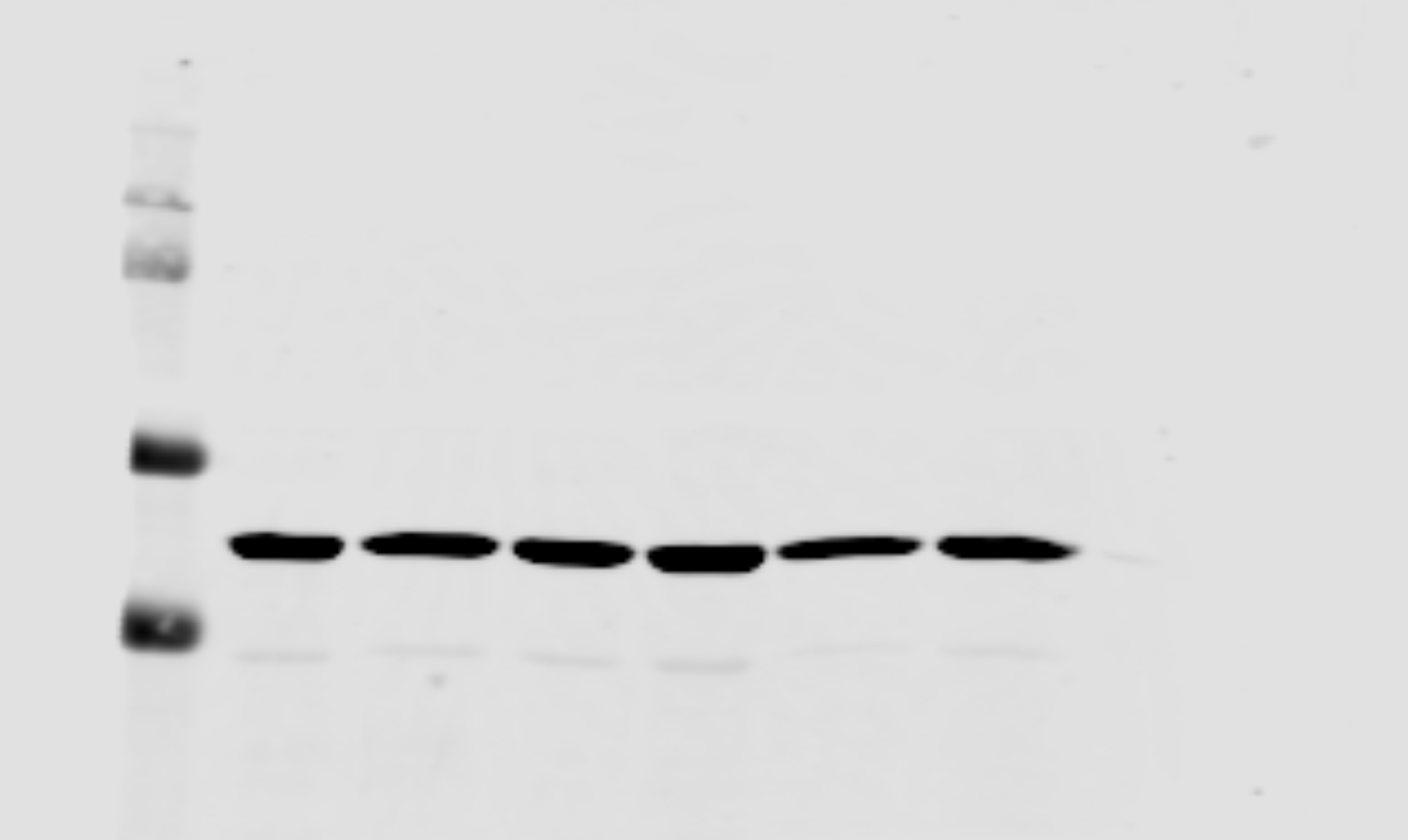

Supplement: Supplementary file 9 — Source data Fig. 6 [file 44318_2026_803_MOESM9_ESM.zip › Fig 6/6H/ACTIN.tif]

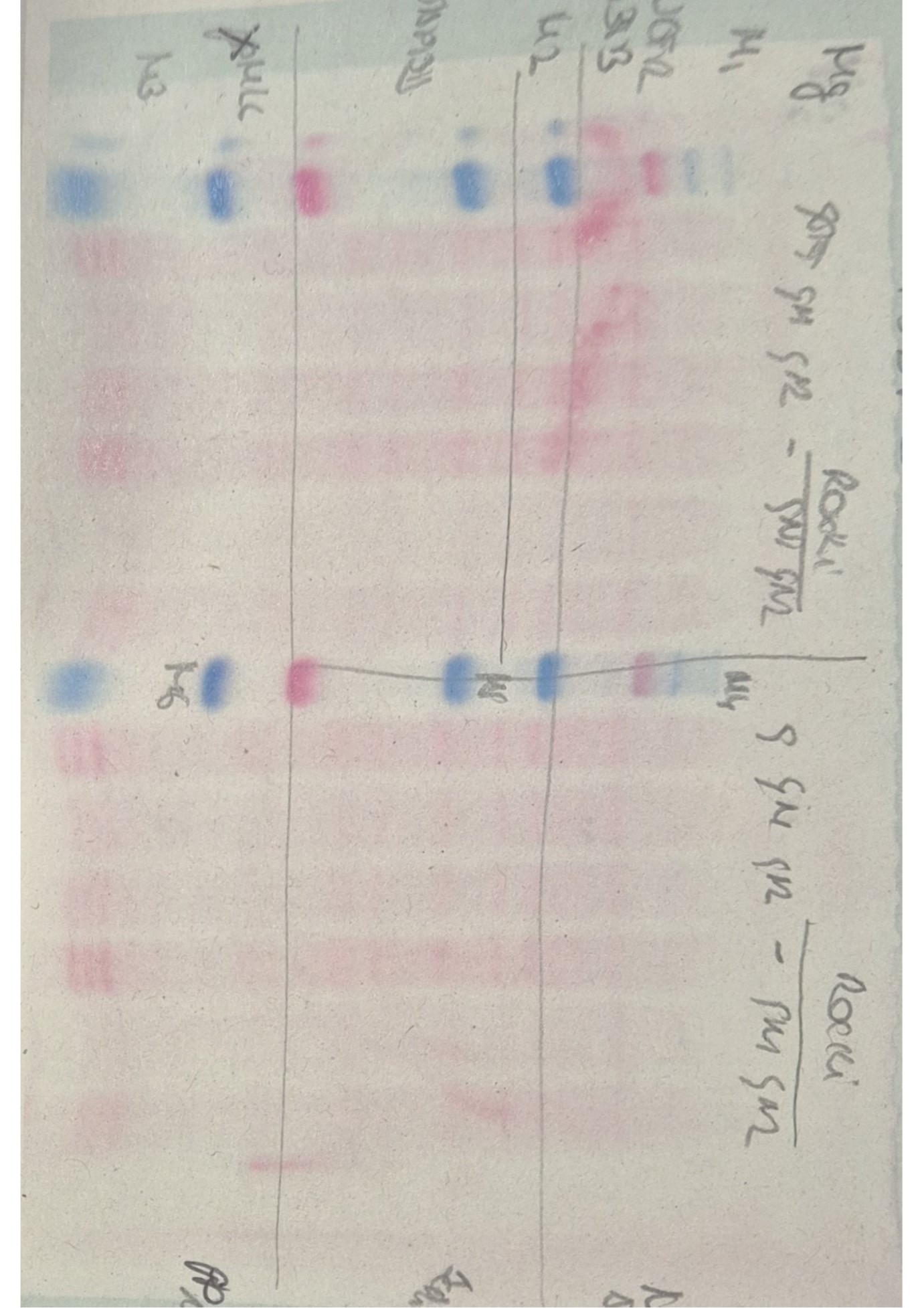

Supplement: Supplementary file 9 — Source data Fig. 6 [file 44318_2026_803_MOESM9_ESM.zip › Fig 6/6H/Ponceau.jpg]

## A375M

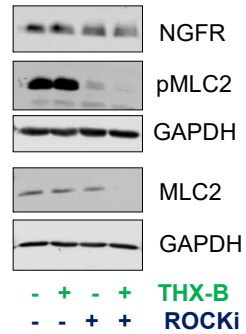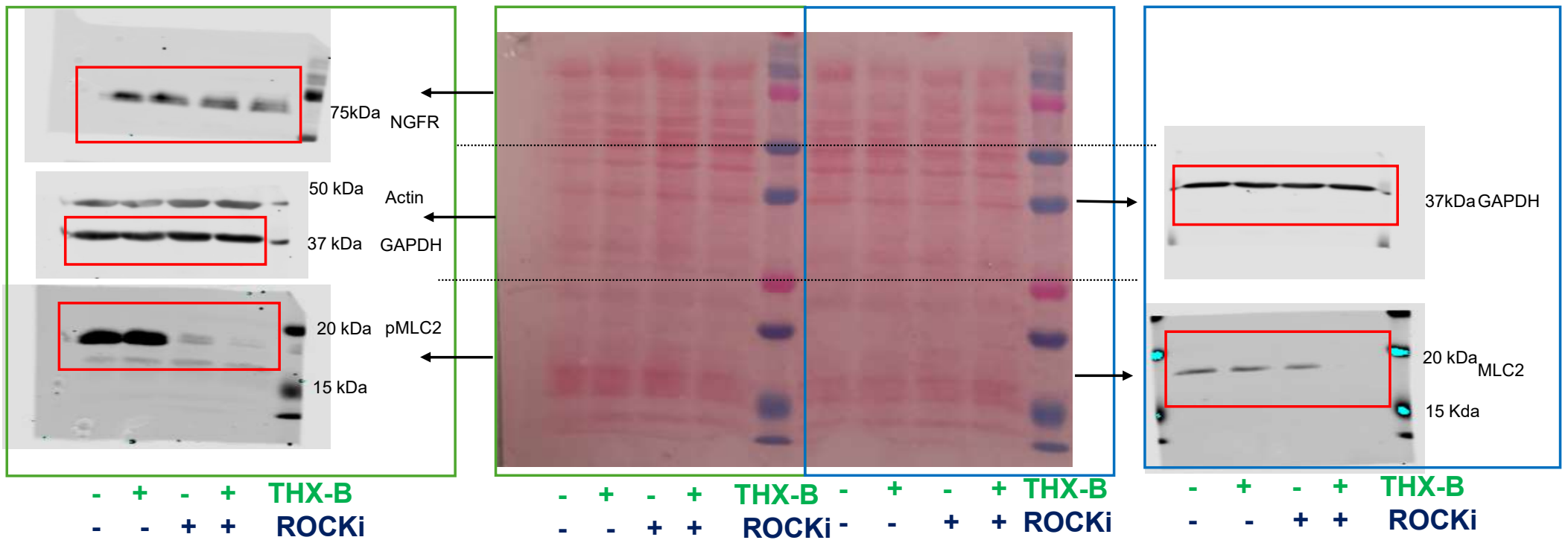

Supplement: Supplementary file 9 — Source data Fig. 6 [file 44318_2026_803_MOESM9_ESM.zip › Fig 6/6J/6j-Readme.pdf]

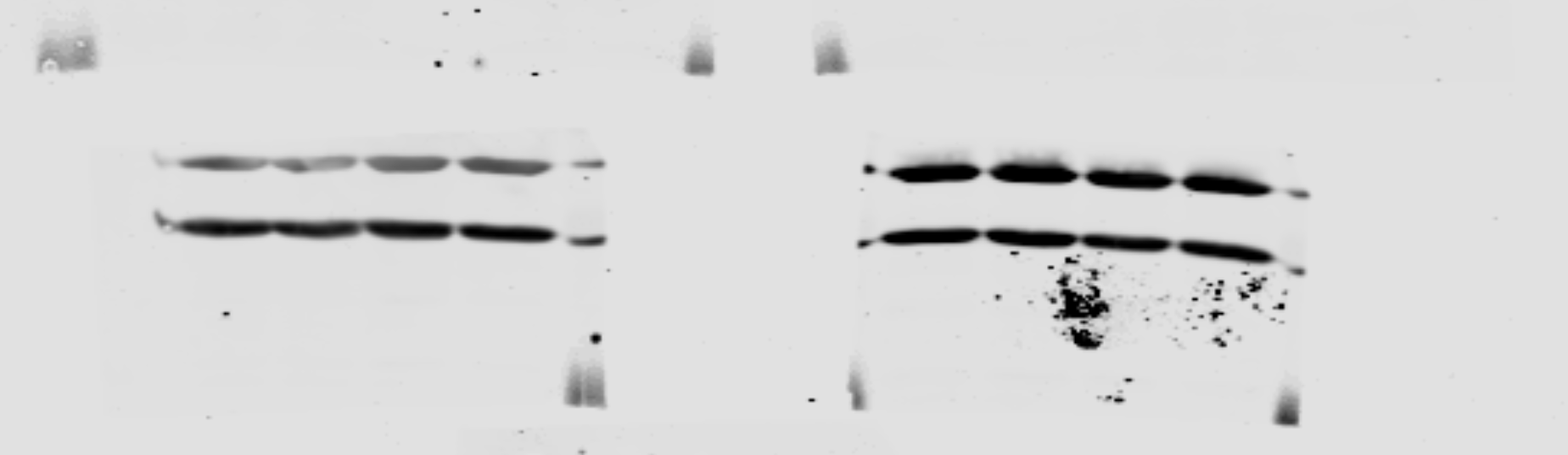

Supplement: Supplementary file 9 — Source data Fig. 6 [file 44318_2026_803_MOESM9_ESM.zip › Fig 6/6J/b-ACTIN - GAPDH-A375M.tif]

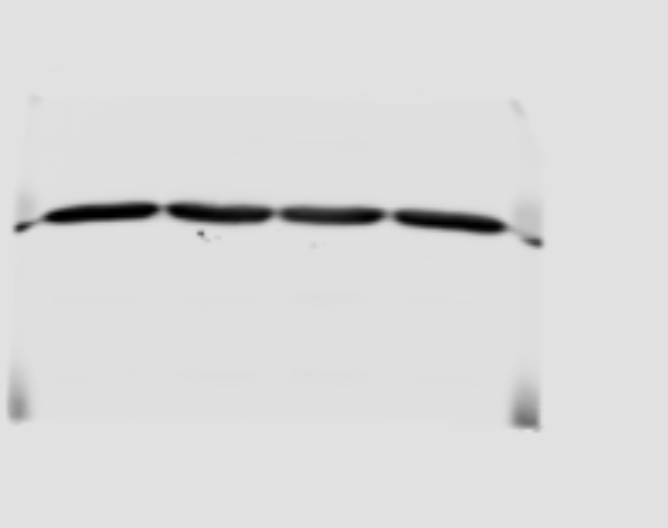

Supplement: Supplementary file 9 — Source data Fig. 6 [file 44318_2026_803_MOESM9_ESM.zip › Fig 6/6J/GAPDH_A375M.tif]

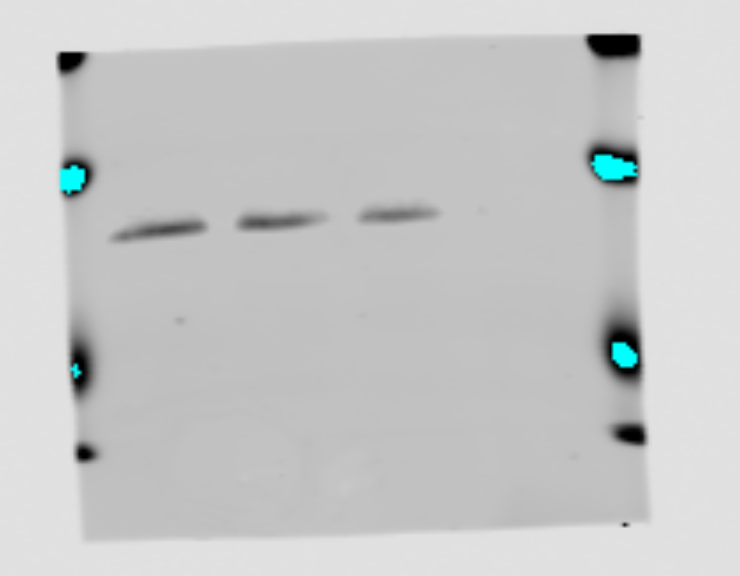

Supplement: Supplementary file 9 — Source data Fig. 6 [file 44318_2026_803_MOESM9_ESM.zip › Fig 6/6J/MLC2_A375M.tif]

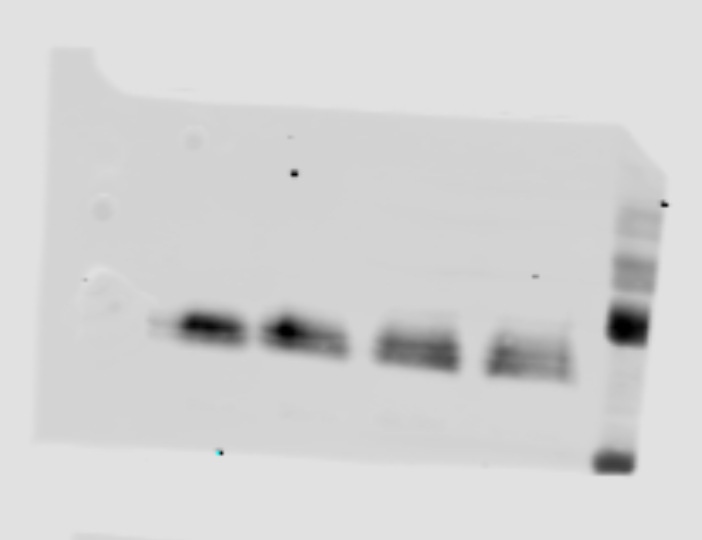

Supplement: Supplementary file 9 — Source data Fig. 6 [file 44318_2026_803_MOESM9_ESM.zip › Fig 6/6J/NGFR_A375M.tif]

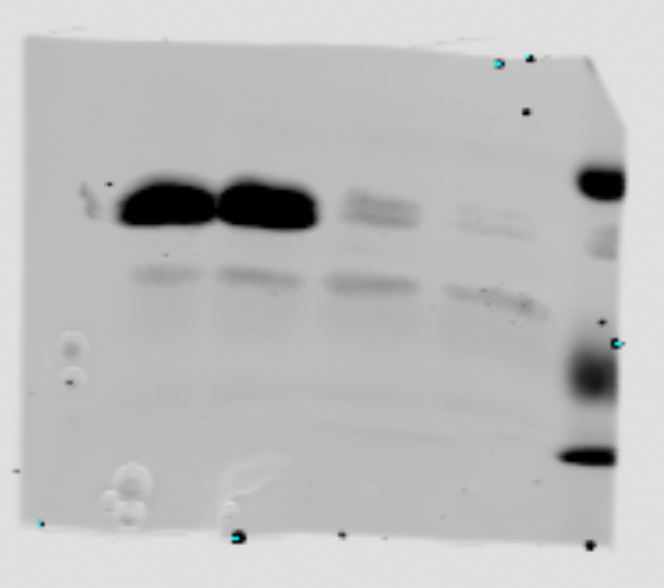

Supplement: Supplementary file 9 — Source data Fig. 6 [file 44318_2026_803_MOESM9_ESM.zip › Fig 6/6J/pMLC2_A375M.tif]

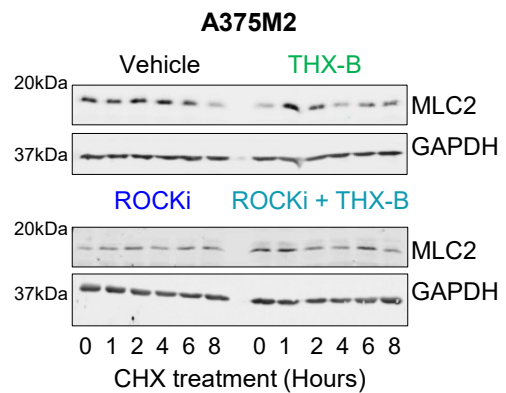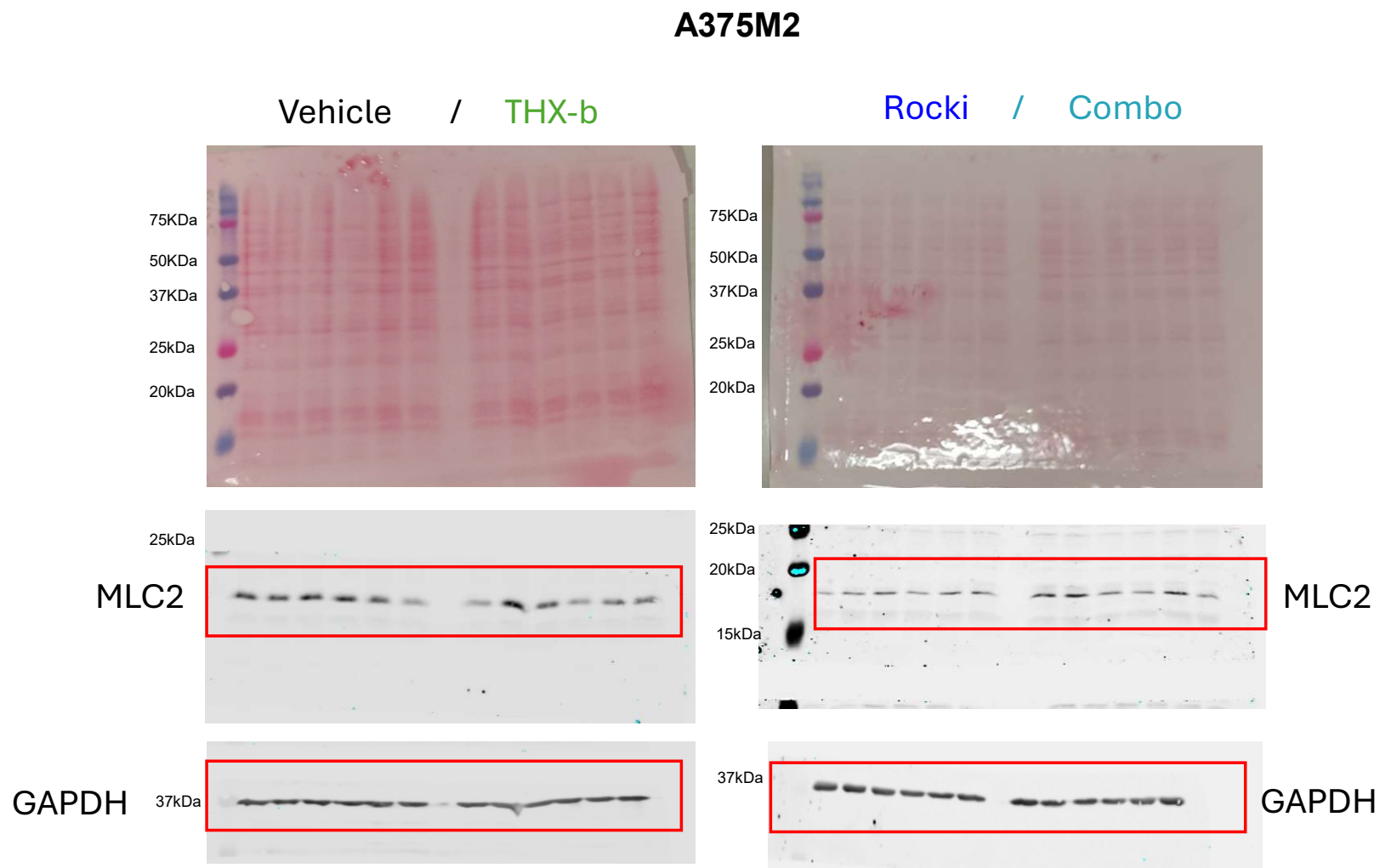

Supplement: Supplementary file 9 — Source data Fig. 6 [file 44318_2026_803_MOESM9_ESM.zip › Fig 6/6L/6L-Readme.pdf]

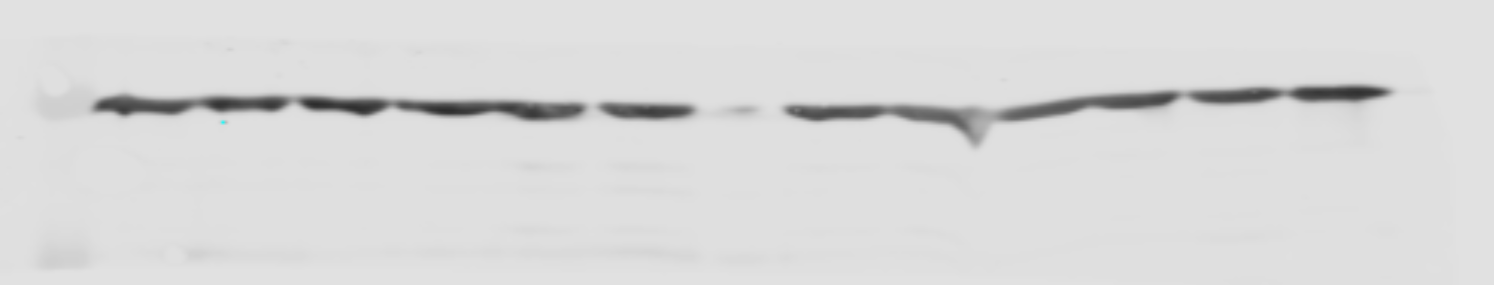

Supplement: Supplementary file 9 — Source data Fig. 6 [file 44318_2026_803_MOESM9_ESM.zip › Fig 6/6L/GAPDH_A375M_CHX_CyTHX-B.tif]

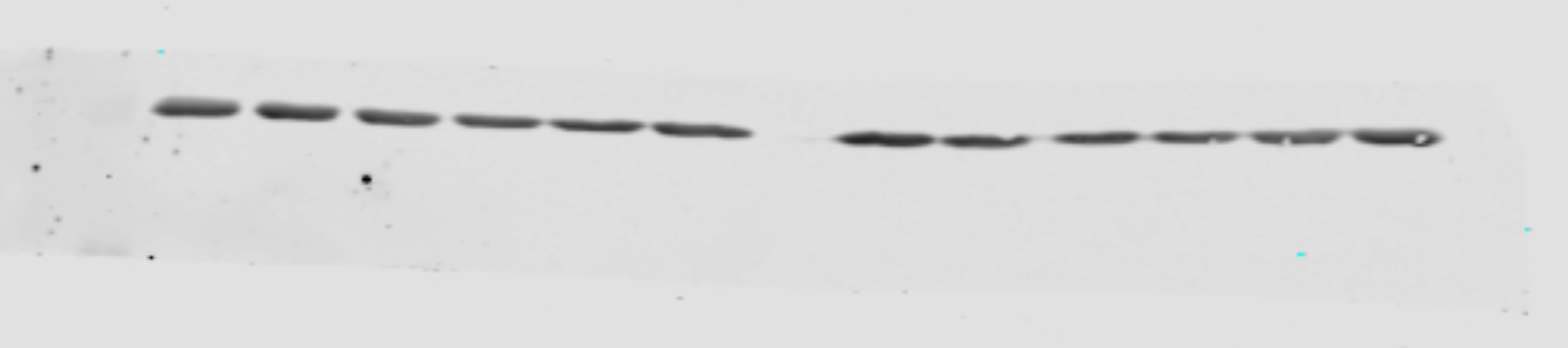

Supplement: Supplementary file 9 — Source data Fig. 6 [file 44318_2026_803_MOESM9_ESM.zip › Fig 6/6L/GAPDH_A375M_CHX_RockCombo.tif]

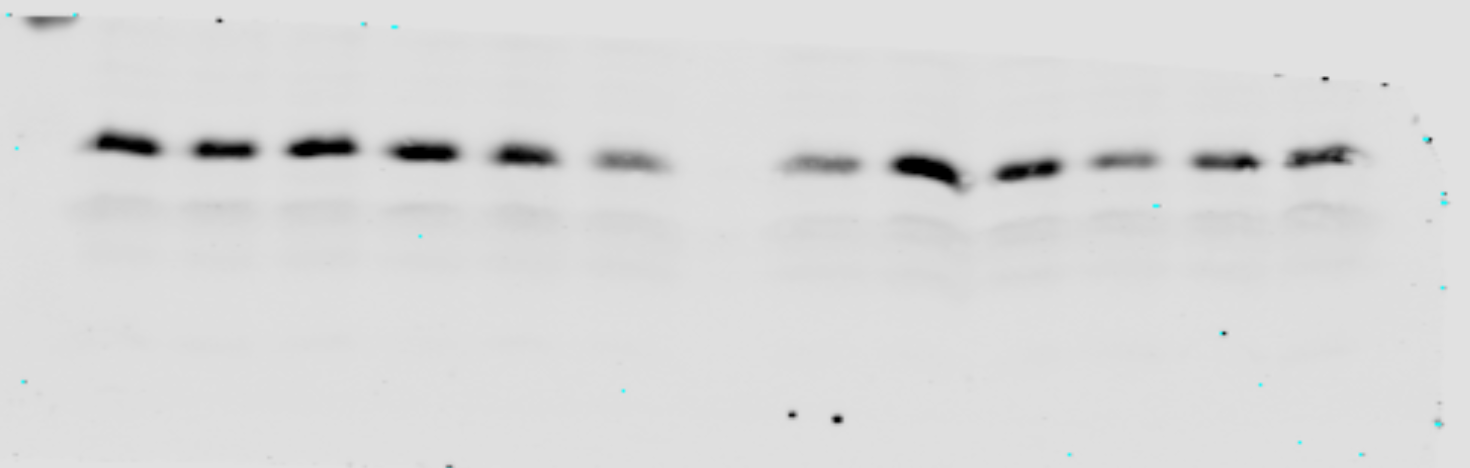

Supplement: Supplementary file 9 — Source data Fig. 6 [file 44318_2026_803_MOESM9_ESM.zip › Fig 6/6L/MLC2_A375M_CHX_CyTHX-B.tif]

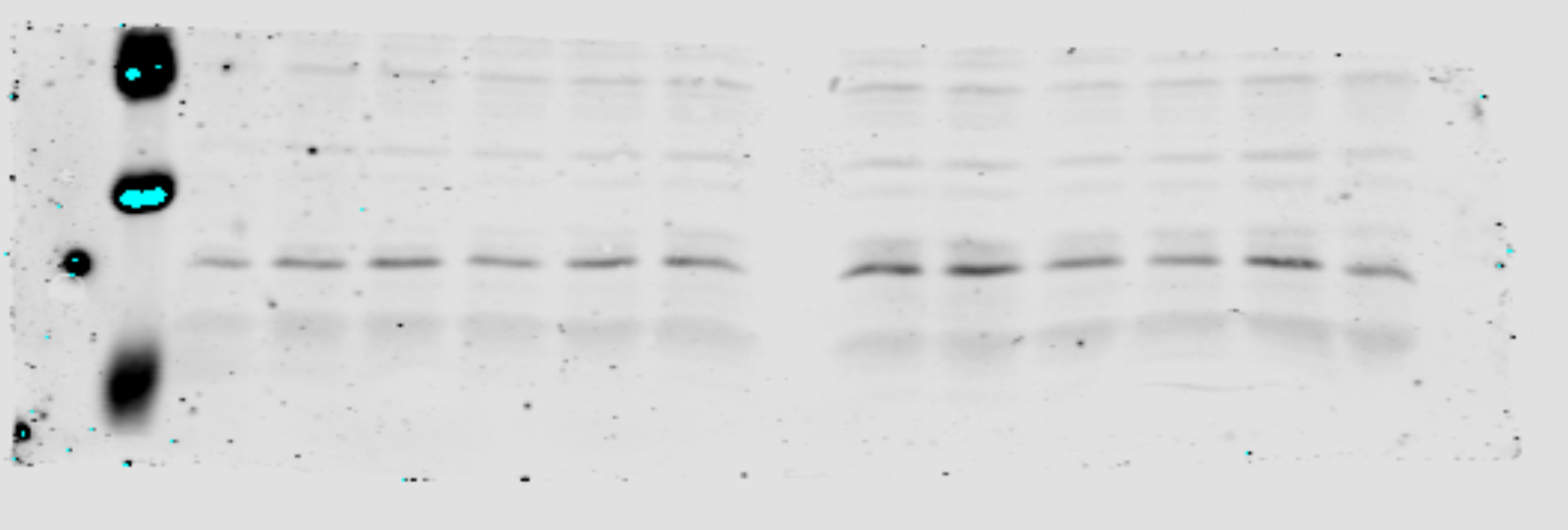

Supplement: Supplementary file 9 — Source data Fig. 6 [file 44318_2026_803_MOESM9_ESM.zip › Fig 6/6L/MLC2_A375M_CHX_RockCombo.tif]

n)

**A375M2**  
**ROCKi + THX-B**

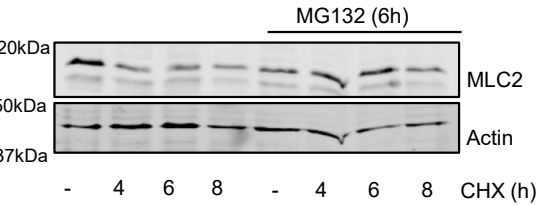

**A375M2 n=3**

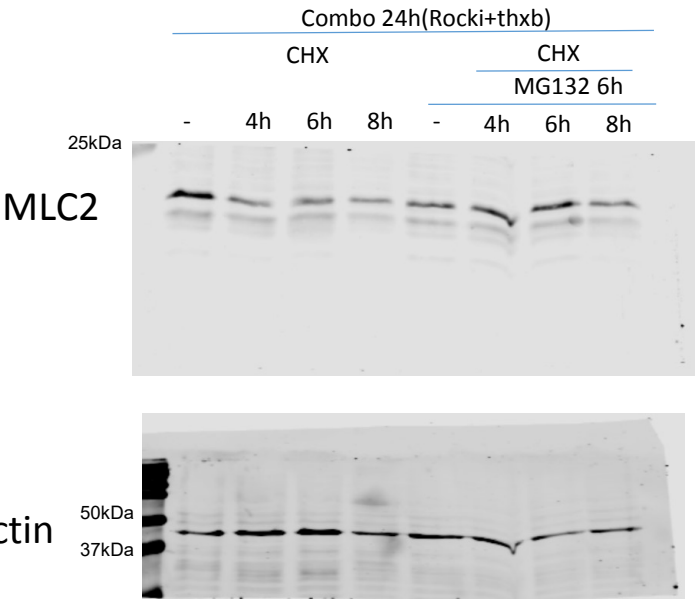

Supplement: Supplementary file 9 — Source data Fig. 6 [file 44318_2026_803_MOESM9_ESM.zip › Fig 6/6N/6N-Readme.pdf]

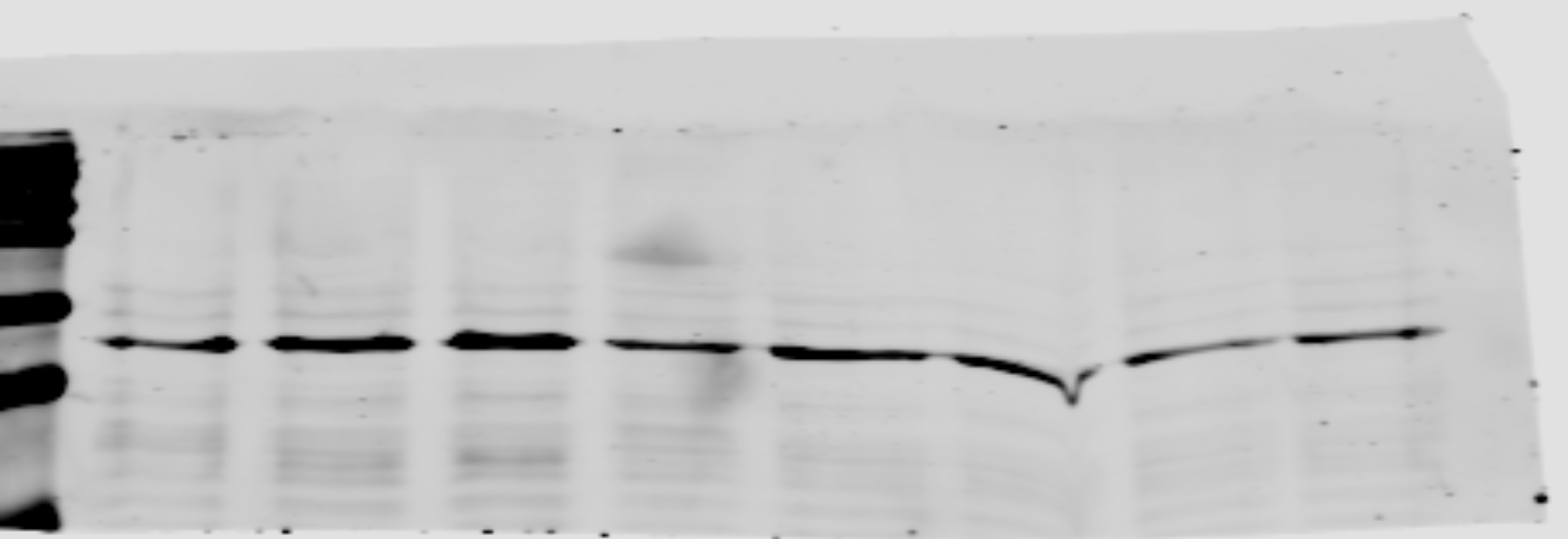

Supplement: Supplementary file 9 — Source data Fig. 6 [file 44318_2026_803_MOESM9_ESM.zip › Fig 6/6N/ACTIN.tif]

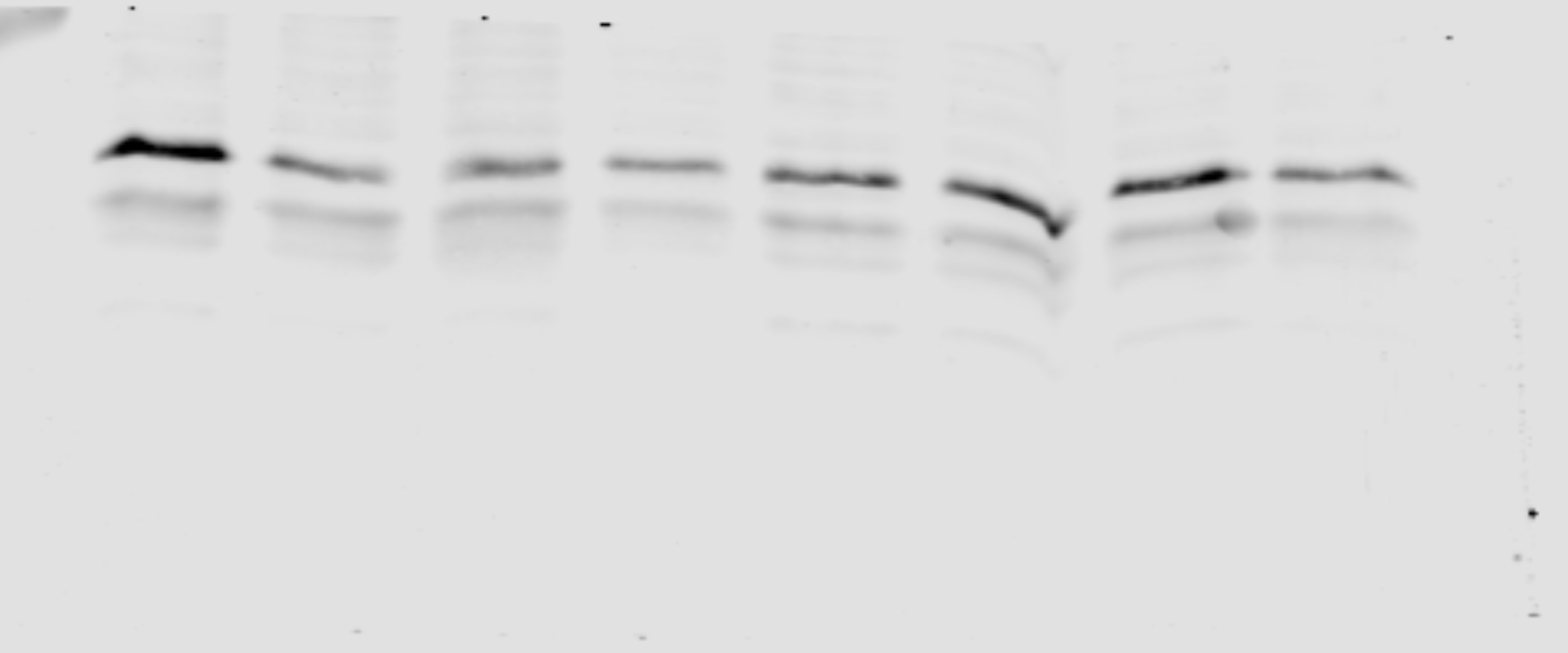

Supplement: Supplementary file 9 — Source data Fig. 6 [file 44318_2026_803_MOESM9_ESM.zip › Fig 6/6N/MLC2.tif]

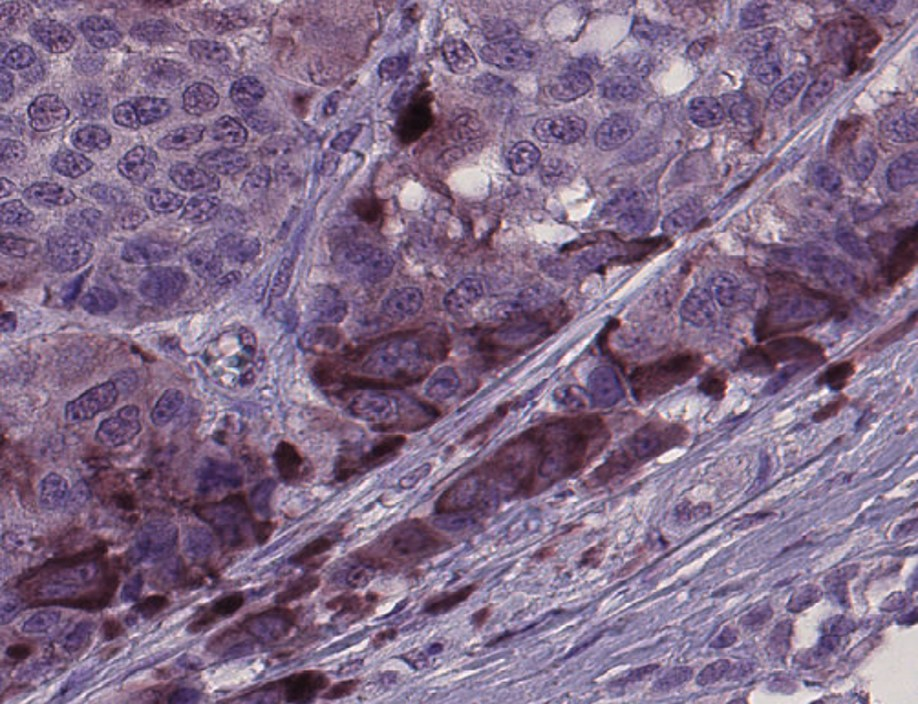

Supplement: Supplementary file 10 — Source data Fig. 7 [file 44318_2026_803_MOESM10_ESM.zip › Fig 7/7A/NGFR-IF.tif]

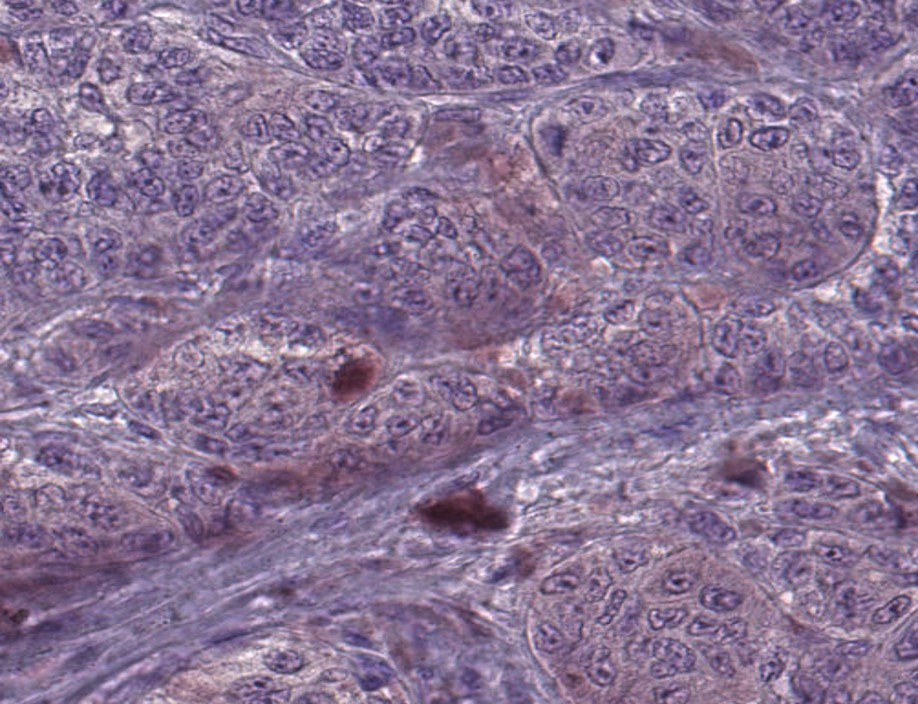

Supplement: Supplementary file 10 — Source data Fig. 7 [file 44318_2026_803_MOESM10_ESM.zip › Fig 7/7A/NGFR-TB.tif]

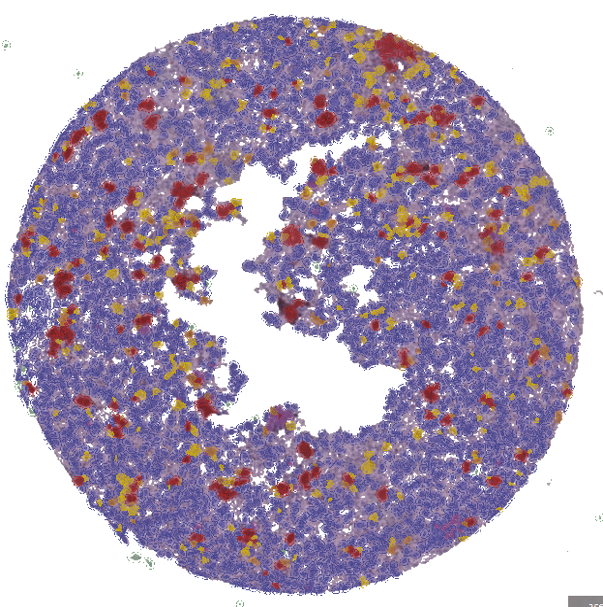

Supplement: Supplementary file 10 — Source data Fig. 7 [file 44318_2026_803_MOESM10_ESM.zip › Fig 7/7C/H-Score Metastasis.tif]

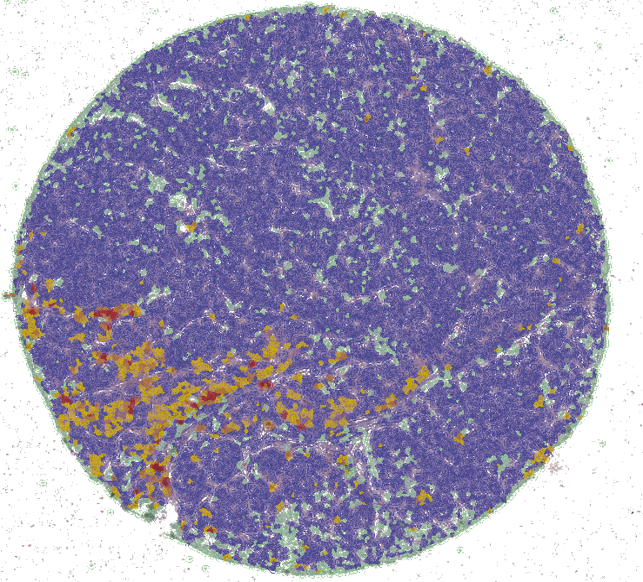

Supplement: Supplementary file 10 — Source data Fig. 7 [file 44318_2026_803_MOESM10_ESM.zip › Fig 7/7C/H-Score Primary.tif]

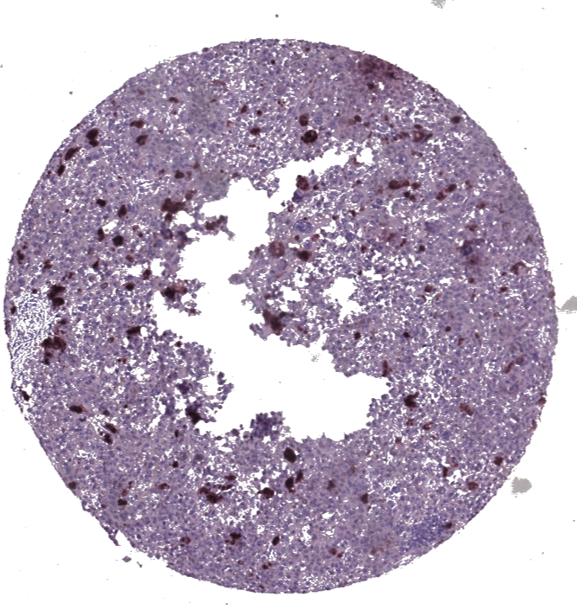

Supplement: Supplementary file 10 — Source data Fig. 7 [file 44318_2026_803_MOESM10_ESM.zip › Fig 7/7C/NGFR Metastasis.tif]

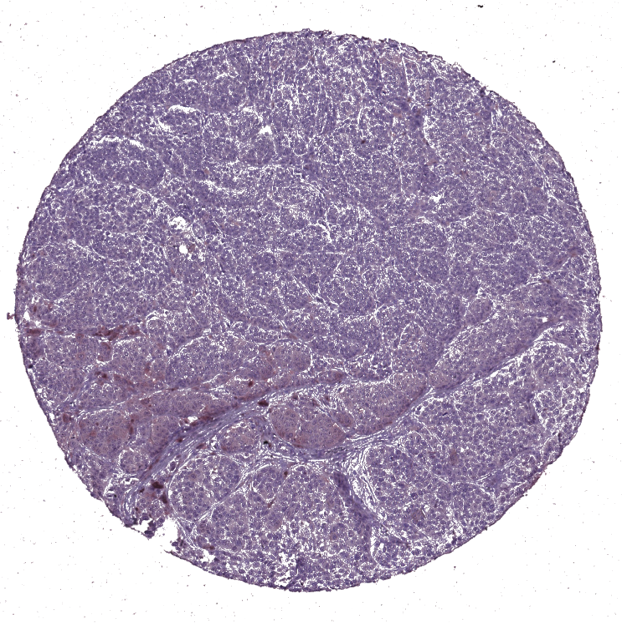

Supplement: Supplementary file 10 — Source data Fig. 7 [file 44318_2026_803_MOESM10_ESM.zip › Fig 7/7C/NGFR Primary.tif]

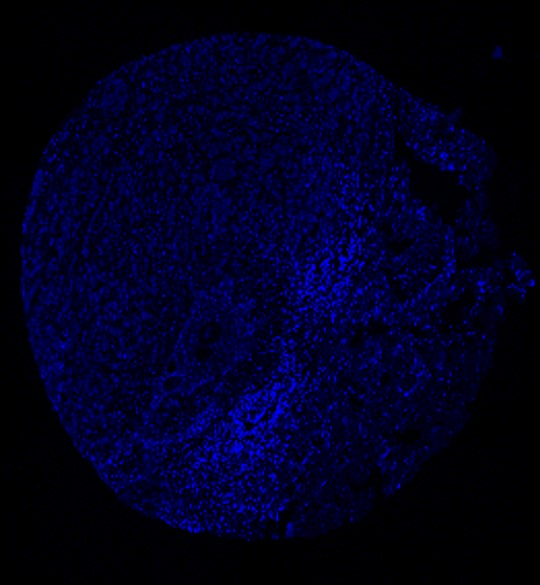

Supplement: Supplementary file 10 — Source data Fig. 7 [file 44318_2026_803_MOESM10_ESM.zip › Fig 7/7G/IF-DAPI.tif]

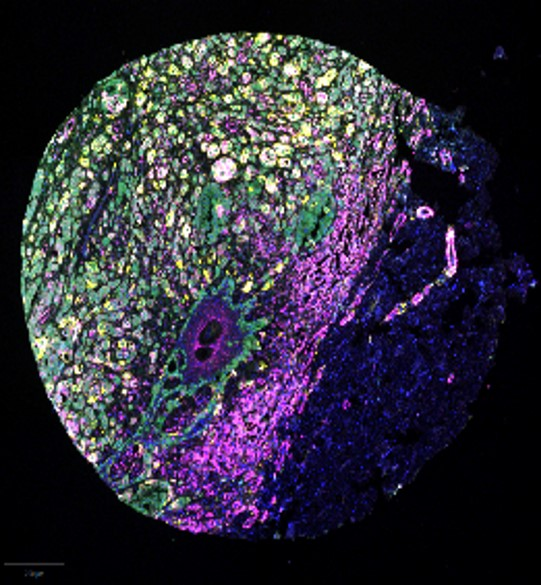

Supplement: Supplementary file 10 — Source data Fig. 7 [file 44318_2026_803_MOESM10_ESM.zip › Fig 7/7G/IF-Merge.tif]

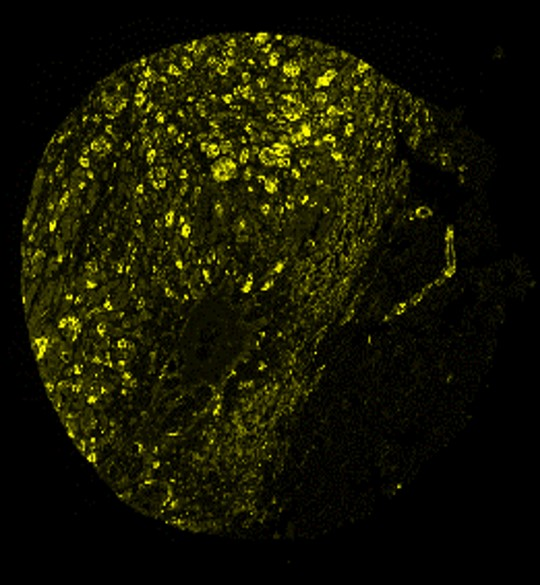

Supplement: Supplementary file 10 — Source data Fig. 7 [file 44318_2026_803_MOESM10_ESM.zip › Fig 7/7G/IF-NGFR.tif]

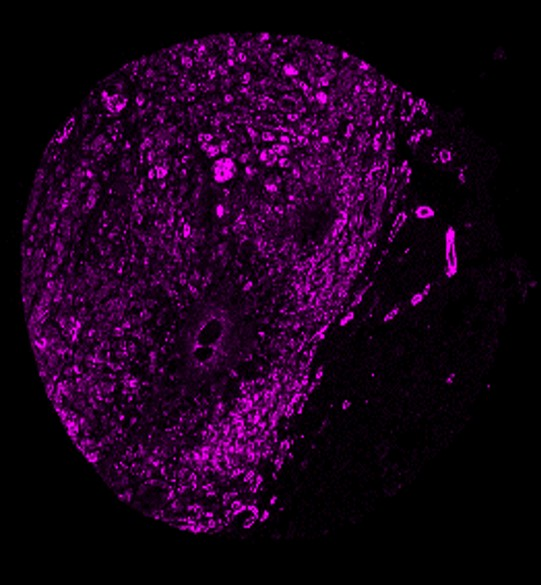

Supplement: Supplementary file 10 — Source data Fig. 7 [file 44318_2026_803_MOESM10_ESM.zip › Fig 7/7G/IF-pMLC2.tif]

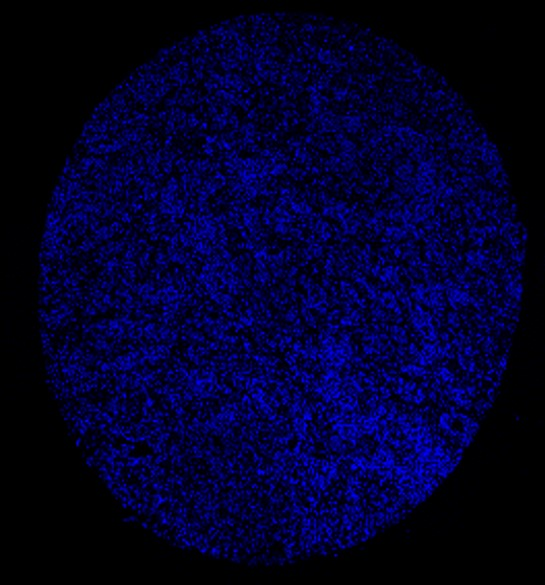

Supplement: Supplementary file 10 — Source data Fig. 7 [file 44318_2026_803_MOESM10_ESM.zip › Fig 7/7G/TB-Dapi.tif]

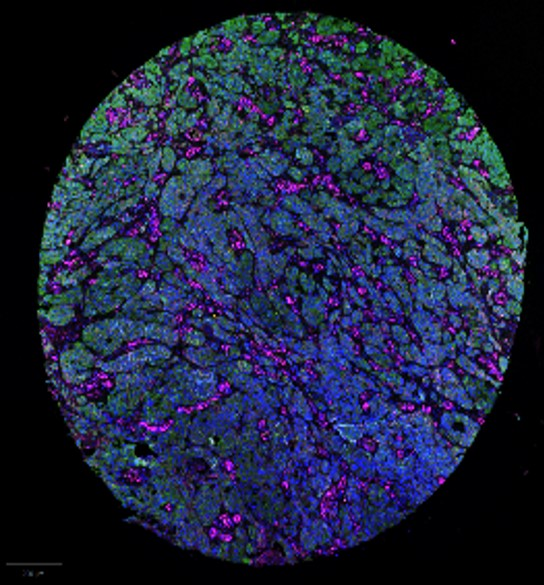

Supplement: Supplementary file 10 — Source data Fig. 7 [file 44318_2026_803_MOESM10_ESM.zip › Fig 7/7G/TB-Merge.tif]

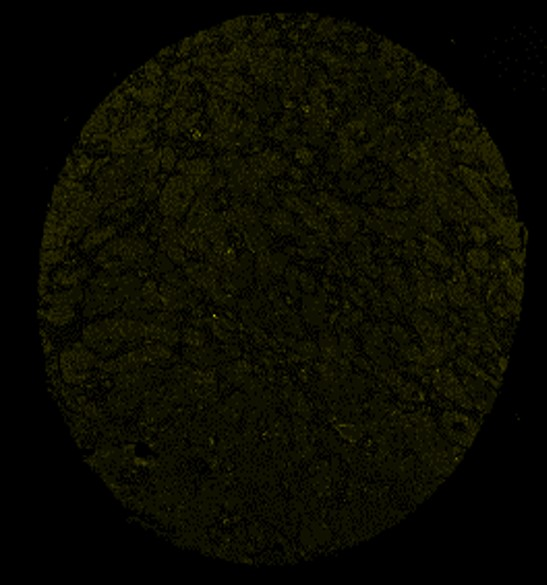

Supplement: Supplementary file 10 — Source data Fig. 7 [file 44318_2026_803_MOESM10_ESM.zip › Fig 7/7G/TB-NGFR.tif]

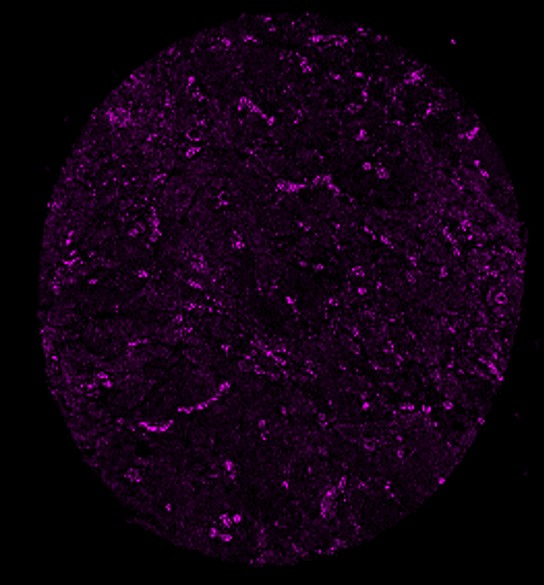

Supplement: Supplementary file 10 — Source data Fig. 7 [file 44318_2026_803_MOESM10_ESM.zip › Fig 7/7G/TB-pMLC2.tif]

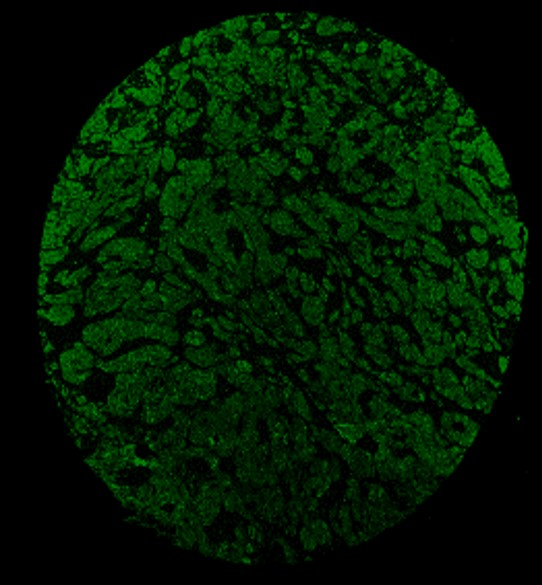

Supplement: Supplementary file 10 — Source data Fig. 7 [file 44318_2026_803_MOESM10_ESM.zip › Fig 7/7G/TB-S100-MelanA.tif]

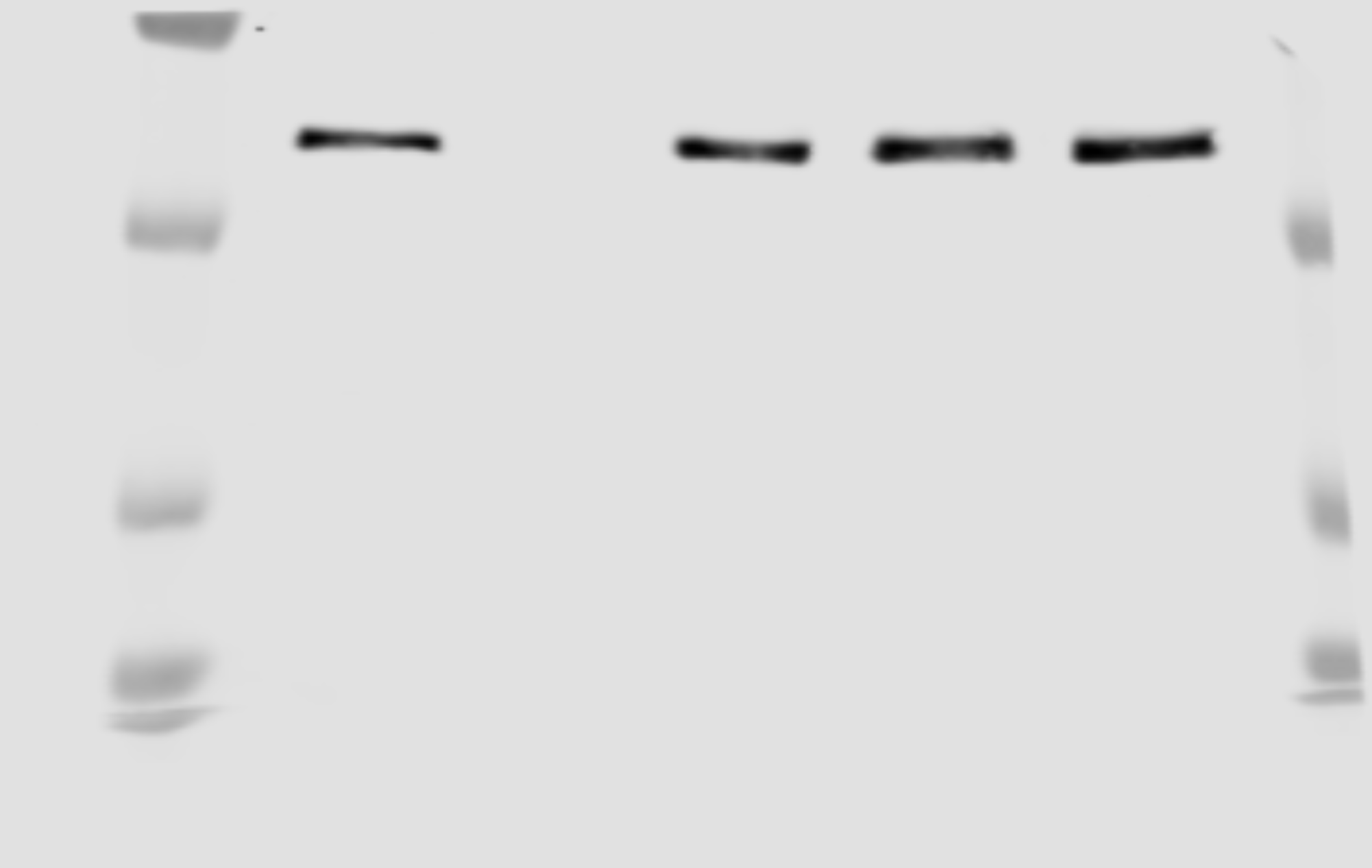

Supplement: Supplementary file 11 — Figure EV1 Source Data [file 44318_2026_803_MOESM11_ESM.zip › Fig EV1/EV1A/Actina.tif]

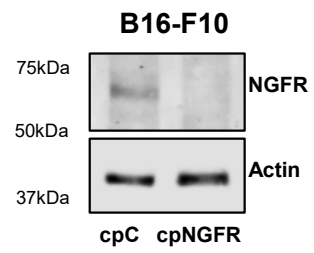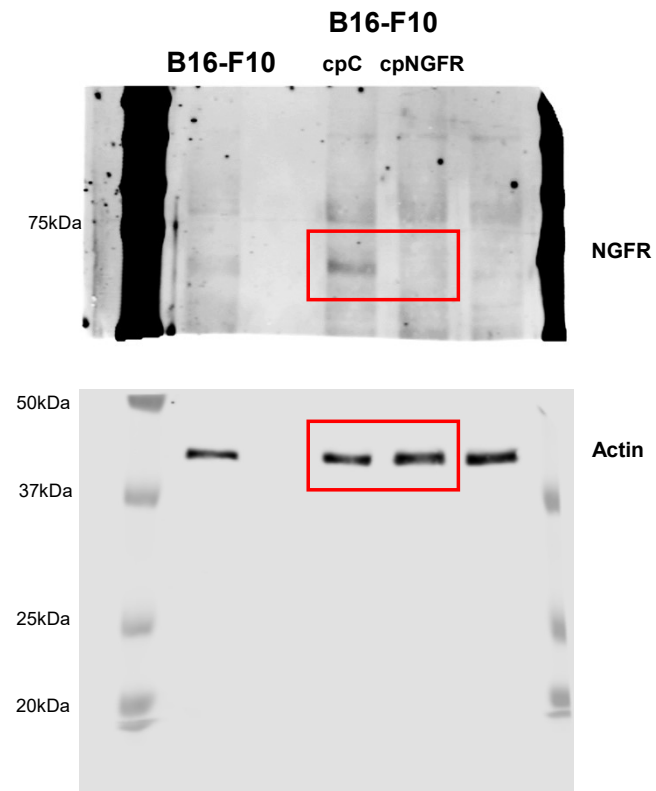

Supplement: Supplementary file 11 — Figure EV1 Source Data [file 44318_2026_803_MOESM11_ESM.zip › Fig EV1/EV1A/EV1A-Readme.pdf]

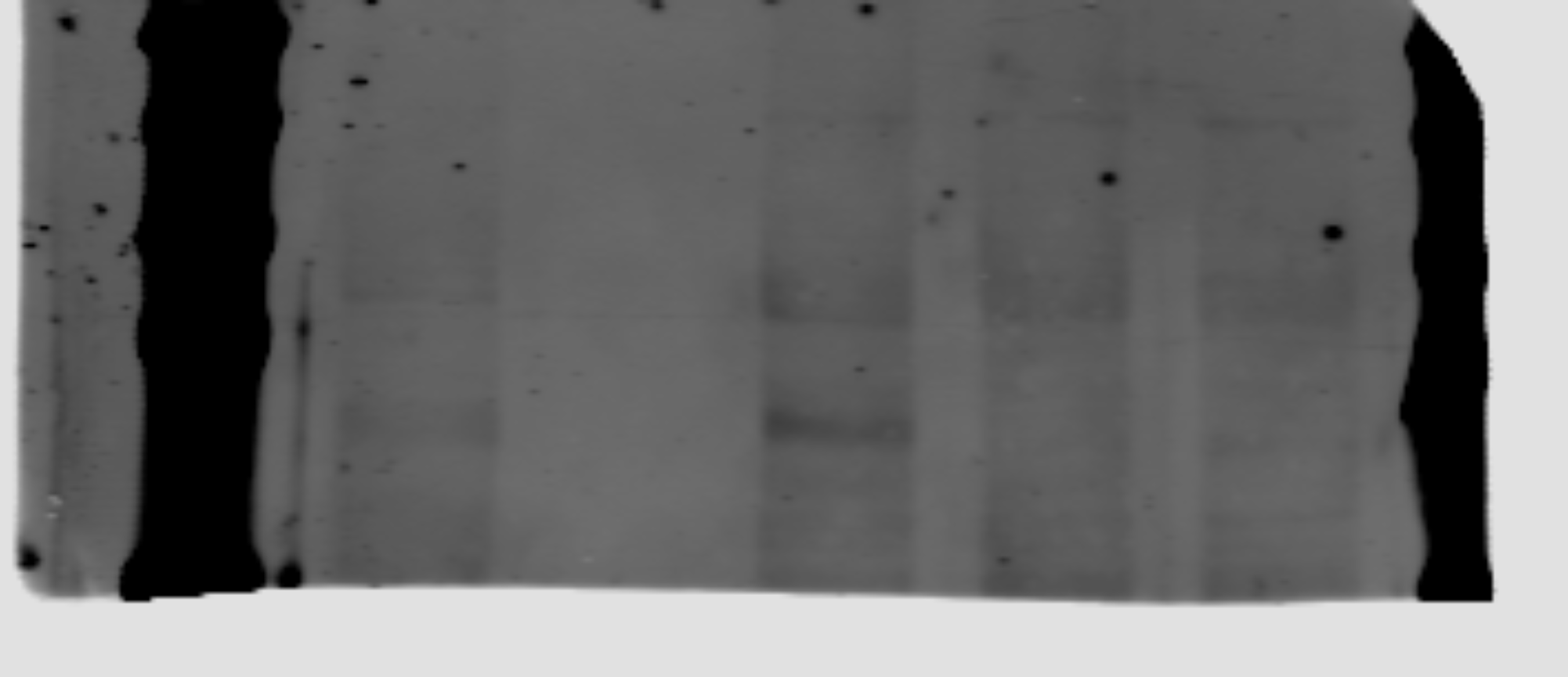

Supplement: Supplementary file 11 — Figure EV1 Source Data [file 44318_2026_803_MOESM11_ESM.zip › Fig EV1/EV1A/NGFR nori.tif]

b)

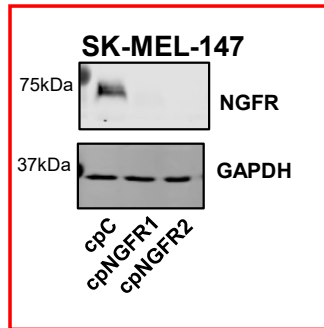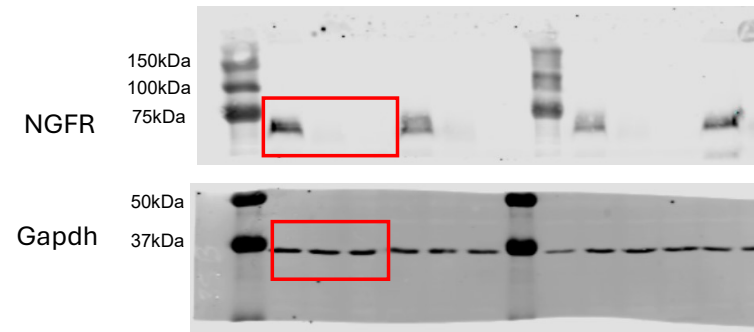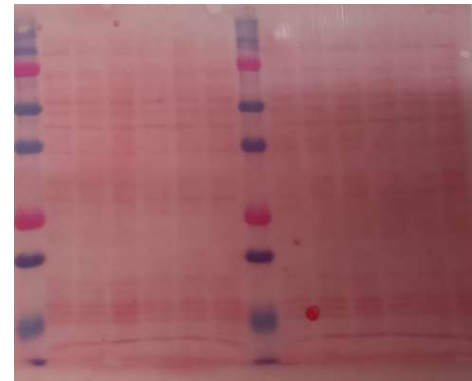

Supplement: Supplementary file 11 — Figure EV1 Source Data [file 44318_2026_803_MOESM11_ESM.zip › Fig EV1/EV1B/EV1B-Readme.pdf]

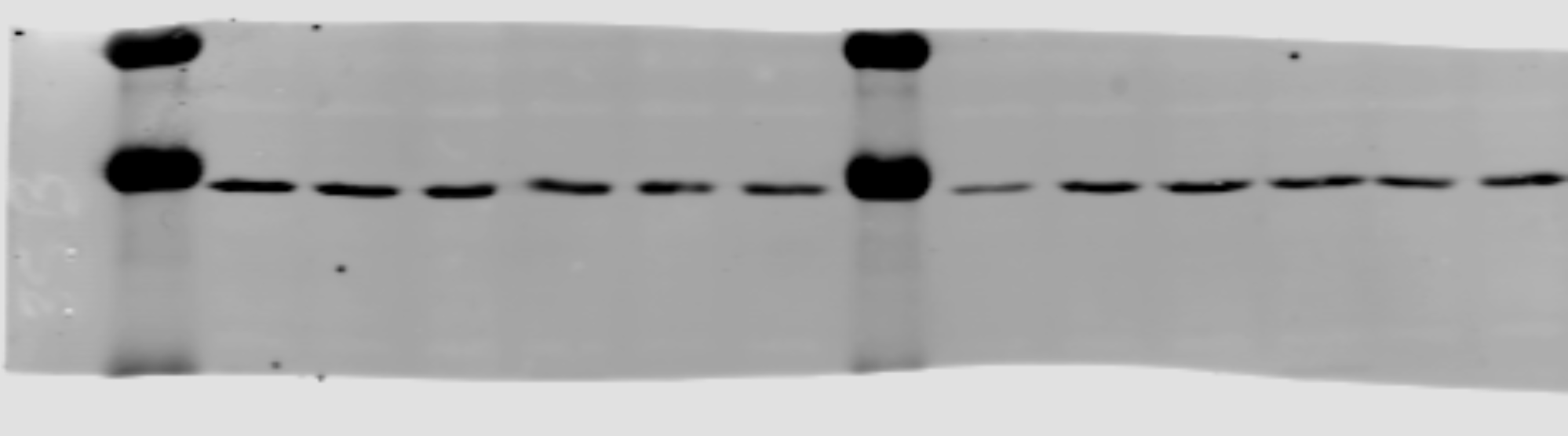

Supplement: Supplementary file 11 — Figure EV1 Source Data [file 44318_2026_803_MOESM11_ESM.zip › Fig EV1/EV1B/GAPDH-SKMEL147.tif]

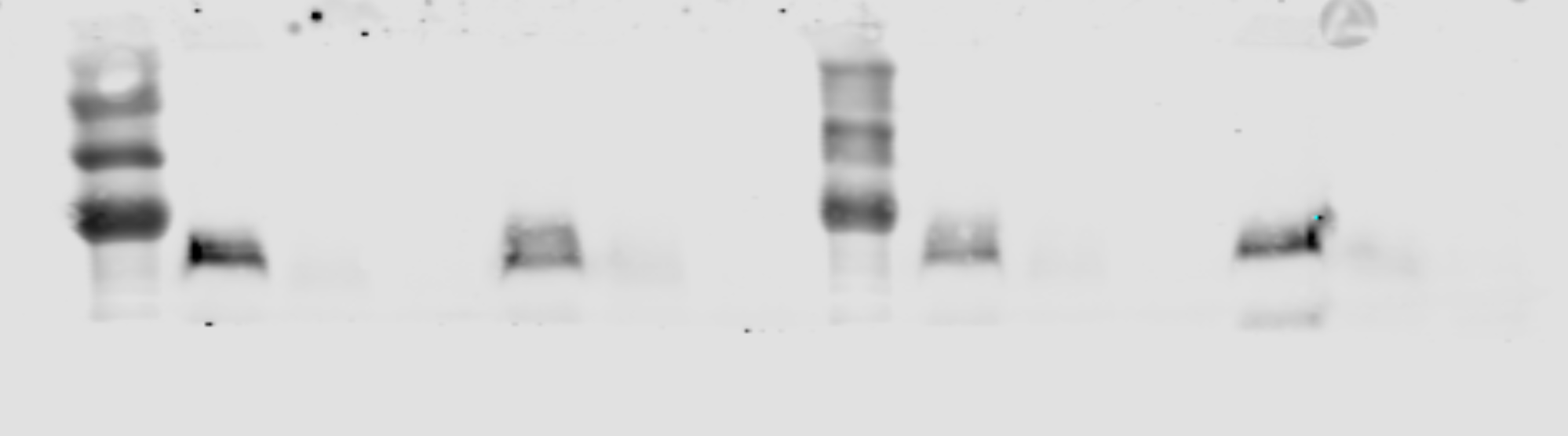

Supplement: Supplementary file 11 — Figure EV1 Source Data [file 44318_2026_803_MOESM11_ESM.zip › Fig EV1/EV1B/NGFR-SKMEL147.tif]

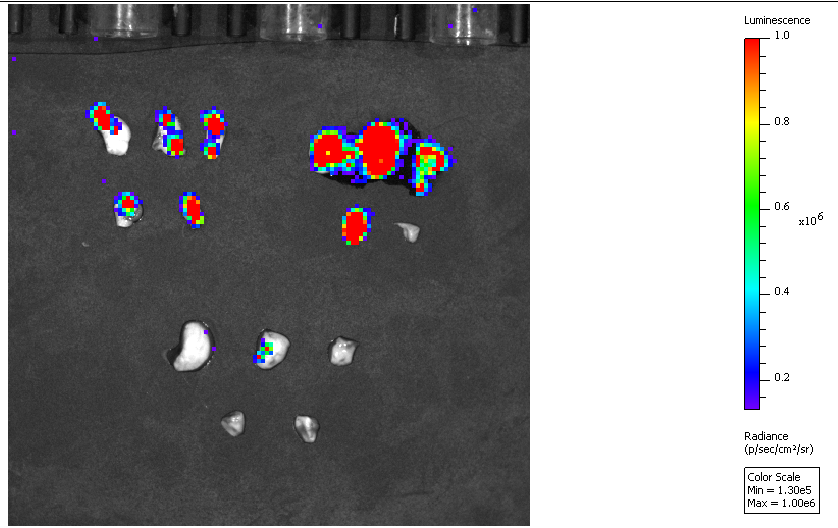

Supplement: Supplementary file 11 — Figure EV1 Source Data [file 44318_2026_803_MOESM11_ESM.zip › Fig EV1/EV1D/CP CTL_DMSO 418-419 y THXB 417.tif]

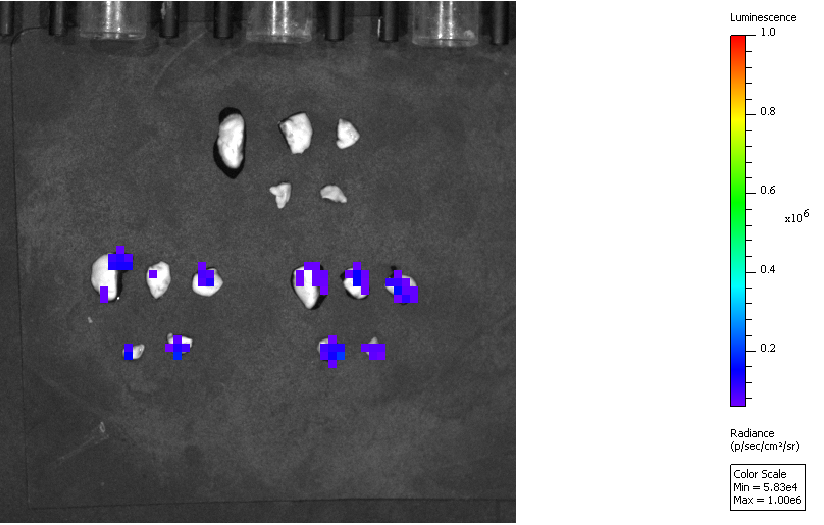

Supplement: Supplementary file 11 — Figure EV1 Source Data [file 44318_2026_803_MOESM11_ESM.zip › Fig EV1/EV1D/CP N1_DMSO 380 y THXB 381-394.tif]

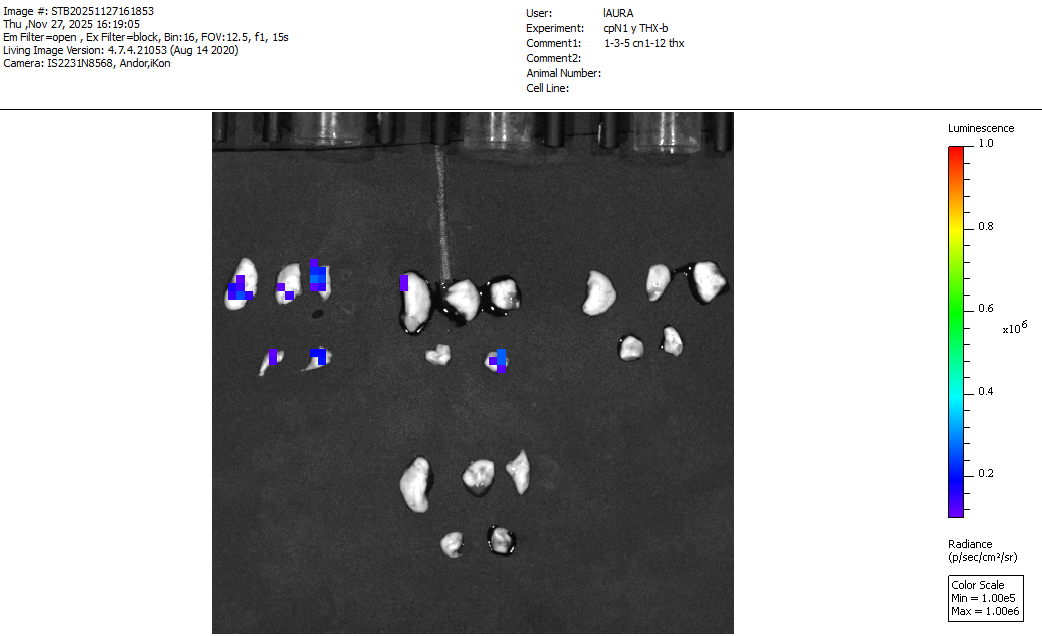

Supplement: Supplementary file 11 — Figure EV1 Source Data [file 44318_2026_803_MOESM11_ESM.zip › Fig EV1/EV1D/CP N1_DMSO 384-386-388 y THXB 413 D2.tif]

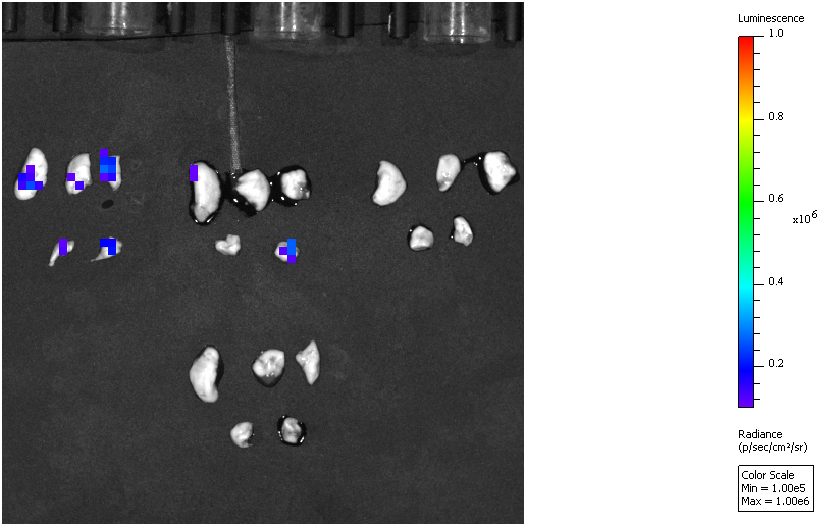

Supplement: Supplementary file 11 — Figure EV1 Source Data [file 44318_2026_803_MOESM11_ESM.zip › Fig EV1/EV1D/CP N1_DMSO 384-386-388 y THXB 413.tif]

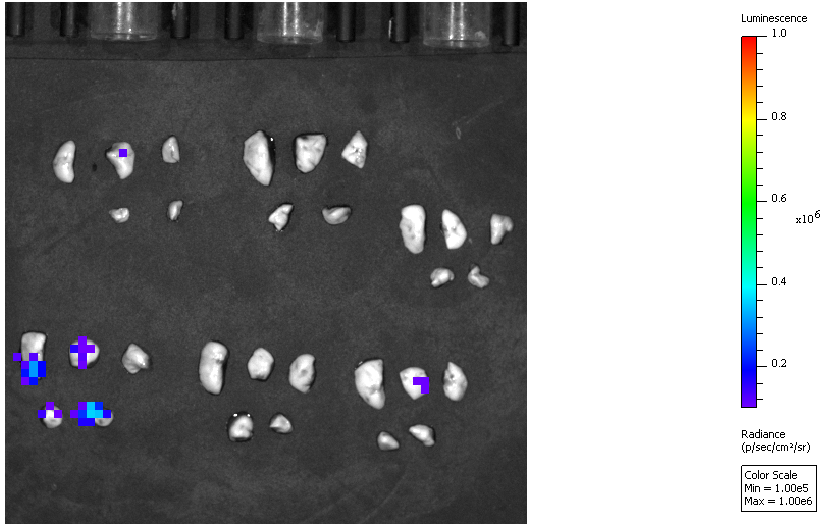

Supplement: Supplementary file 11 — Figure EV1 Source Data [file 44318_2026_803_MOESM11_ESM.zip › Fig EV1/EV1D/CP N2_DMSO 397-401 y THXB 399-389-392-393.tif]

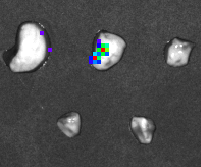

Supplement: Supplementary file 11 — Figure EV1 Source Data [file 44318_2026_803_MOESM11_ESM.zip › Fig EV1/EV1D/cpC THX-B.tif]

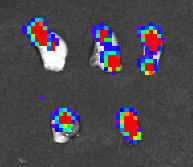

Supplement: Supplementary file 11 — Figure EV1 Source Data [file 44318_2026_803_MOESM11_ESM.zip › Fig EV1/EV1D/cpC Vehicle.tif]

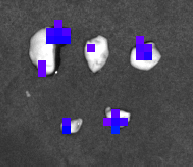

Supplement: Supplementary file 11 — Figure EV1 Source Data [file 44318_2026_803_MOESM11_ESM.zip › Fig EV1/EV1D/cpNGFR1 THX-B.tif]

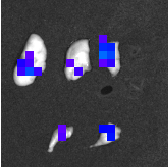

Supplement: Supplementary file 11 — Figure EV1 Source Data [file 44318_2026_803_MOESM11_ESM.zip › Fig EV1/EV1D/cpNGFR1 Vehicle.tif]

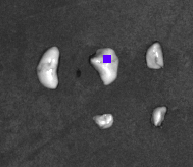

Supplement: Supplementary file 11 — Figure EV1 Source Data [file 44318_2026_803_MOESM11_ESM.zip › Fig EV1/EV1D/cpNGFR2 vehicle.tif]

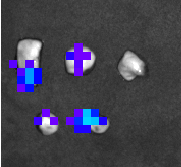

Supplement: Supplementary file 11 — Figure EV1 Source Data [file 44318_2026_803_MOESM11_ESM.zip › Fig EV1/EV1D/cpNGFR2-THX-B.tif]

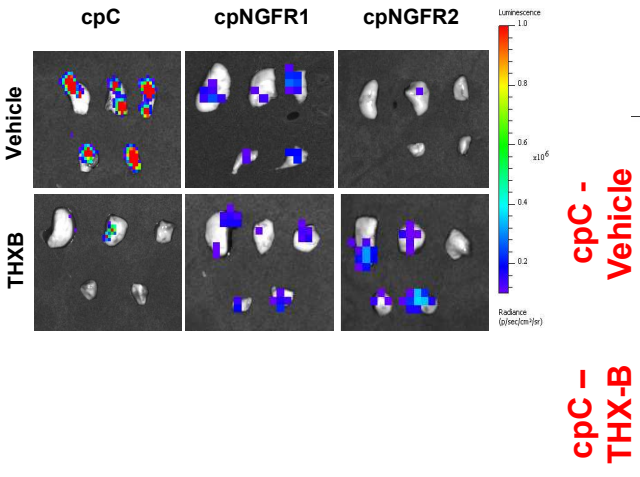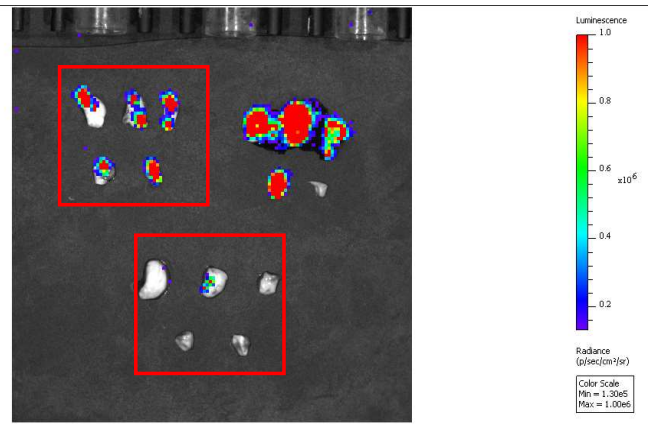

cpNGFR2 - Vehicle

cpNGFR2 - THX-B

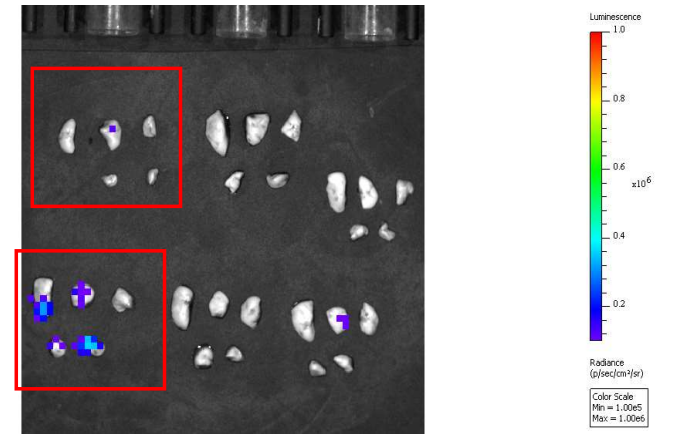

cpNGFR1 - Vehicle

cpNGFR1 - THX-B

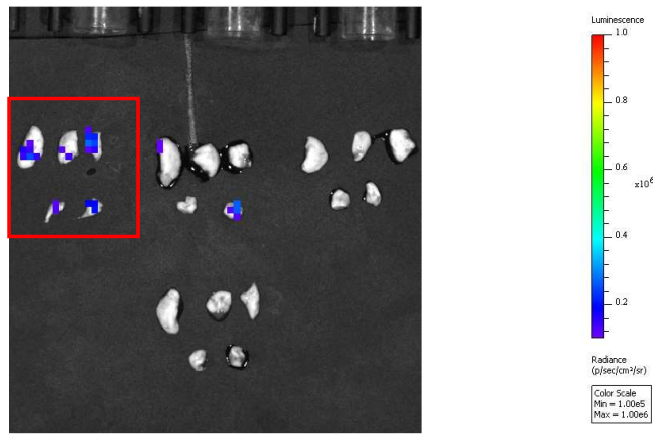

cpNGFR1 - THX-B

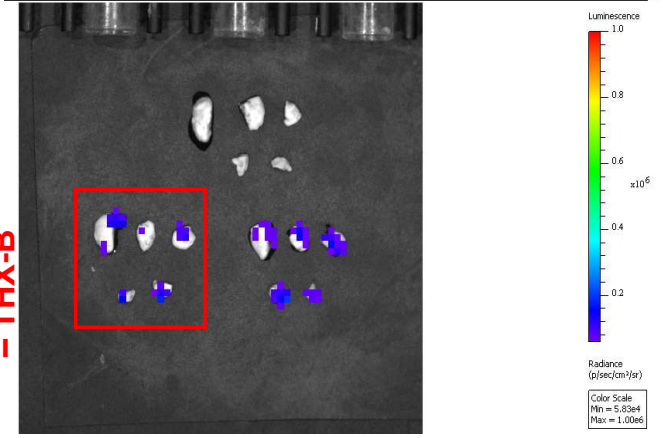

Supplement: Supplementary file 11 — Figure EV1 Source Data [file 44318_2026_803_MOESM11_ESM.zip › Fig EV1/EV1D/EV1D-Readme.pdf]

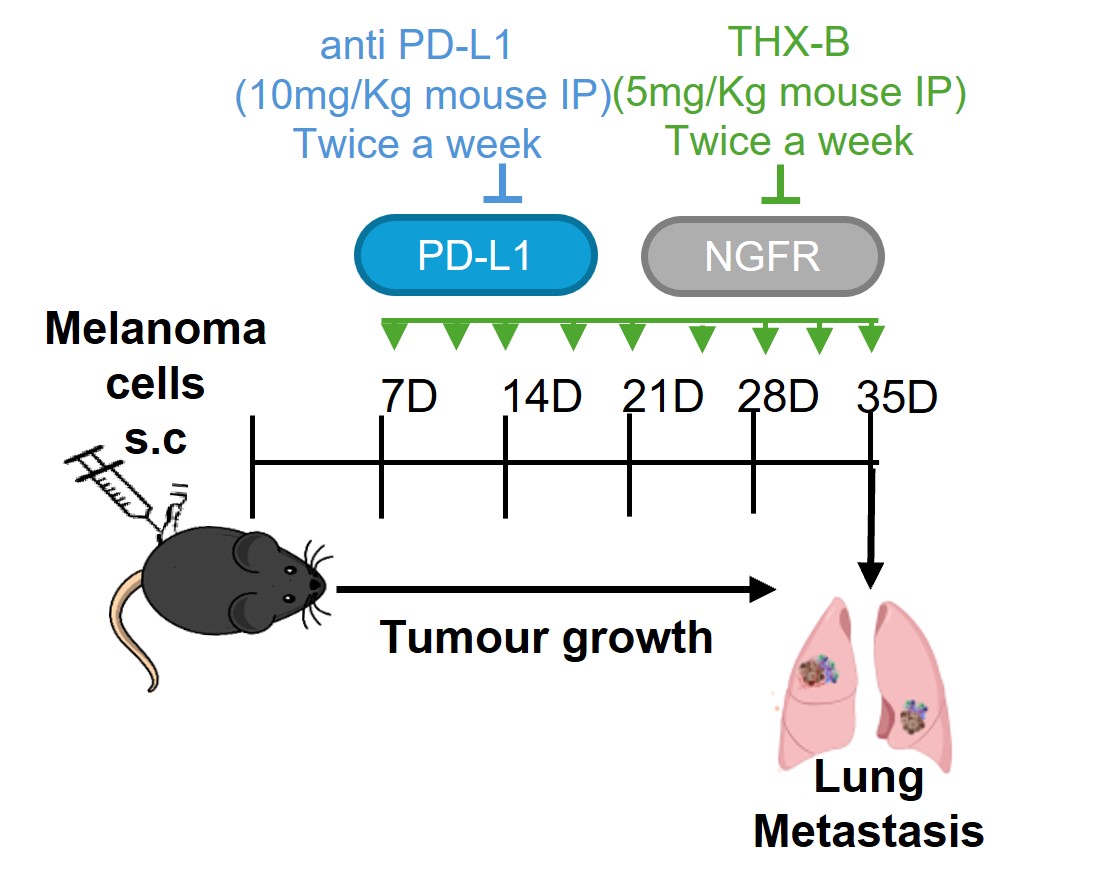

Supplement: Supplementary file 12 — Figure EV2 Source Data [file 44318_2026_803_MOESM12_ESM.zip › Fig EV2/EV2A.jpg]

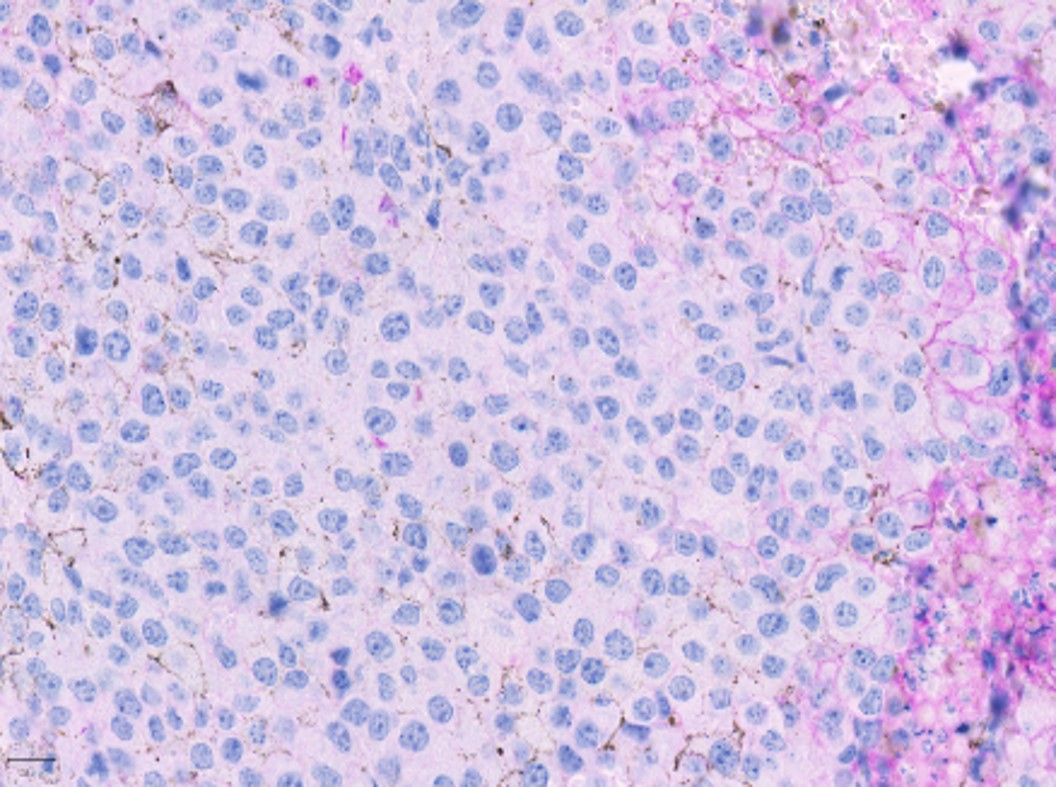

Supplement: Supplementary file 12 — Figure EV2 Source Data [file 44318_2026_803_MOESM12_ESM.zip › Fig EV2/EV2B/F10-2.tif]

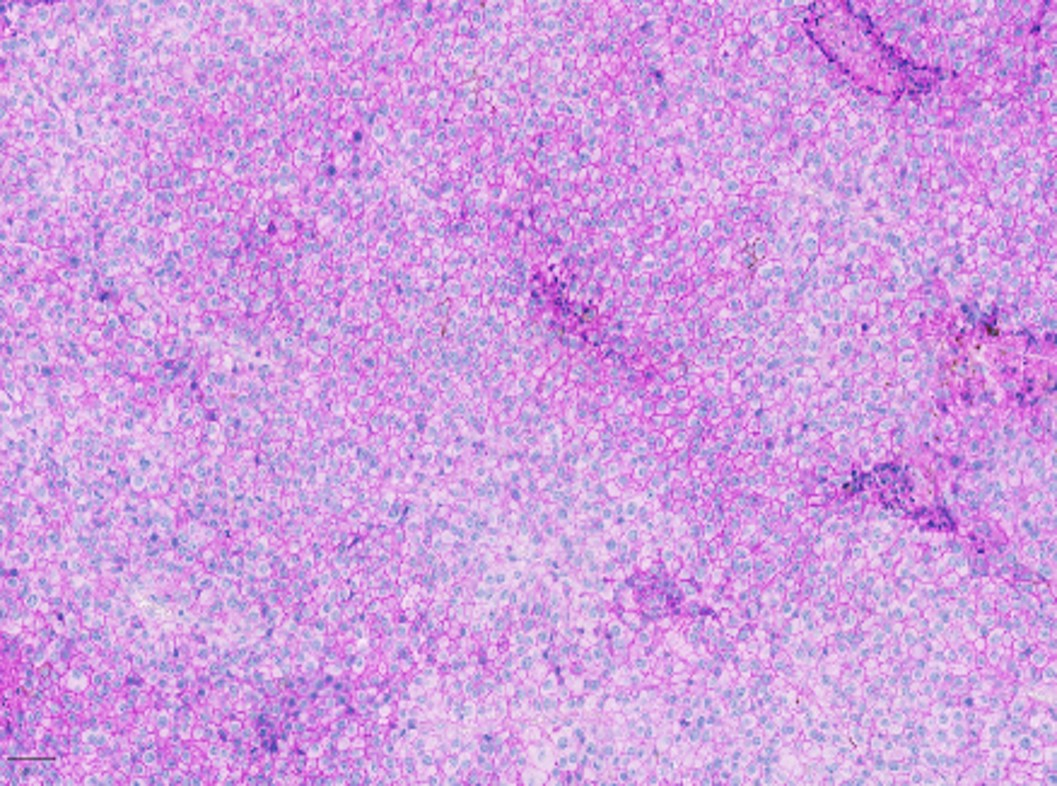

Supplement: Supplementary file 12 — Figure EV2 Source Data [file 44318_2026_803_MOESM12_ESM.zip › Fig EV2/EV2B/F10-R28-1.tif]

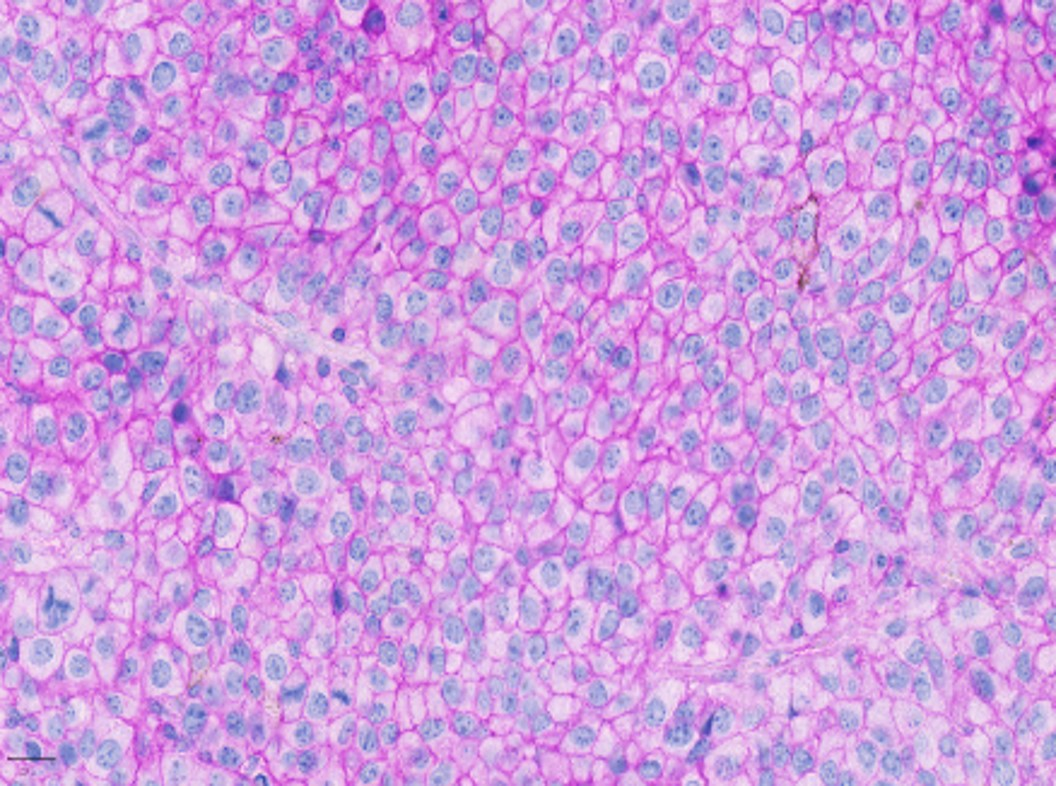

Supplement: Supplementary file 12 — Figure EV2 Source Data [file 44318_2026_803_MOESM12_ESM.zip › Fig EV2/EV2B/F10-R28-2.tif]

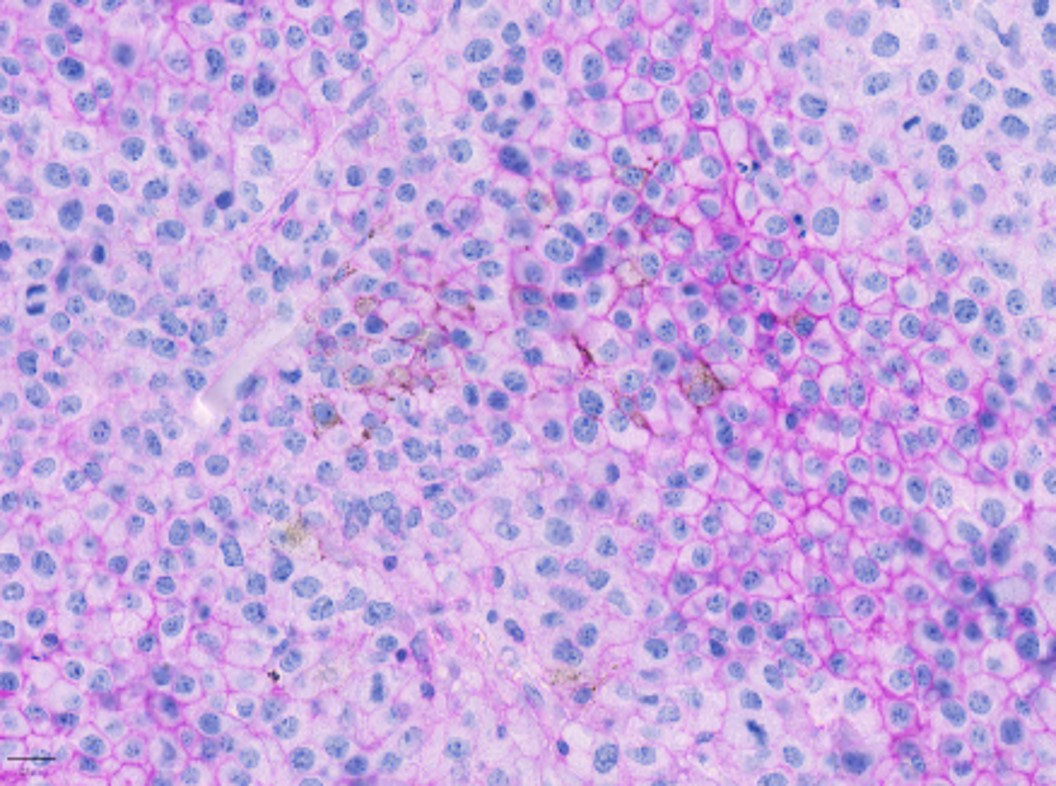

Supplement: Supplementary file 12 — Figure EV2 Source Data [file 44318_2026_803_MOESM12_ESM.zip › Fig EV2/EV2B/F10-R31-2.tif]

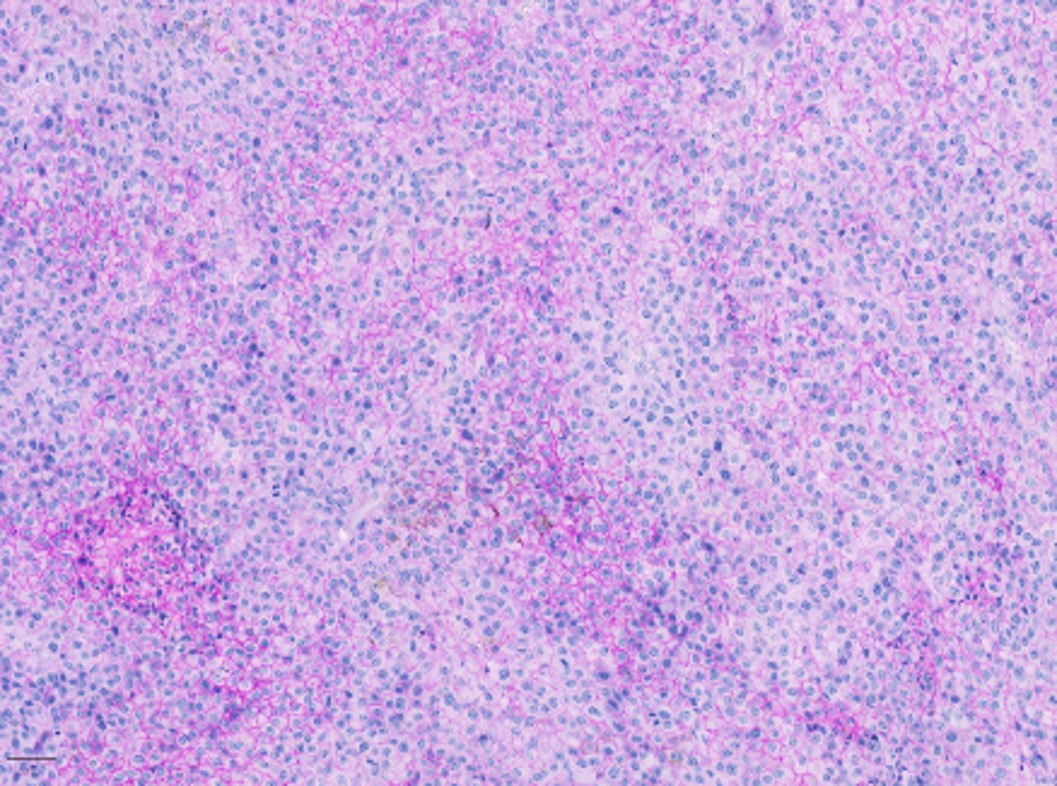

Supplement: Supplementary file 12 — Figure EV2 Source Data [file 44318_2026_803_MOESM12_ESM.zip › Fig EV2/EV2B/F10-R31.tif]

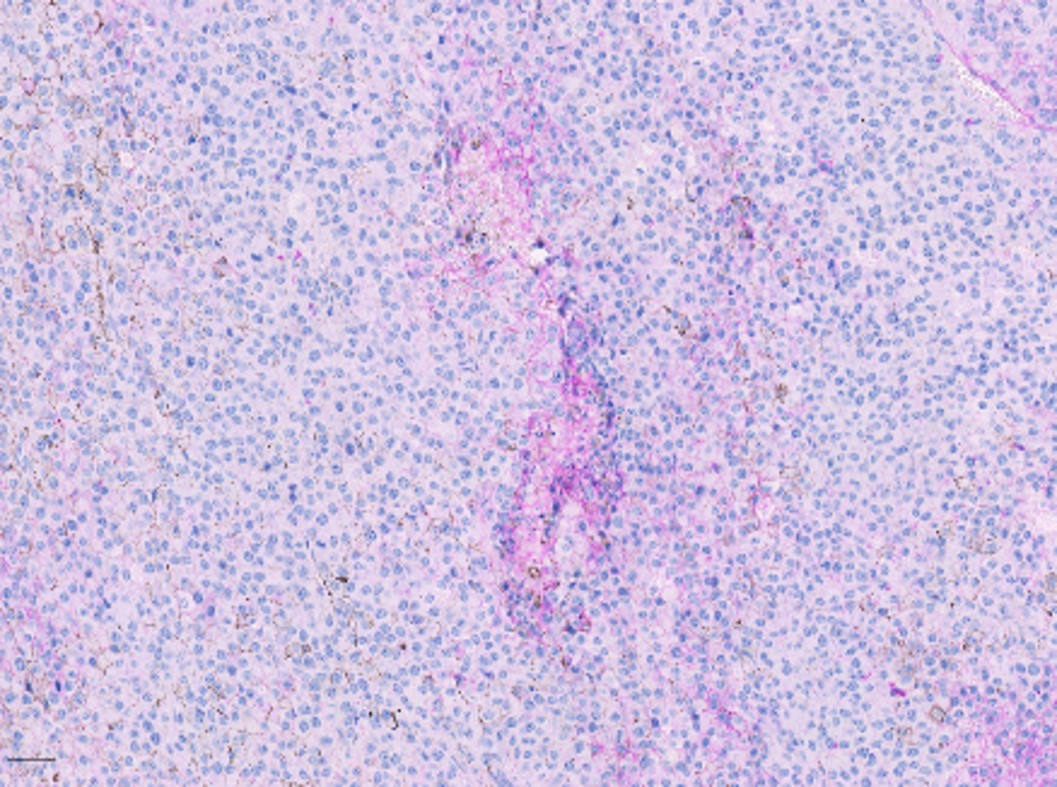

Supplement: Supplementary file 12 — Figure EV2 Source Data [file 44318_2026_803_MOESM12_ESM.zip › Fig EV2/EV2B/F10_1.tif]

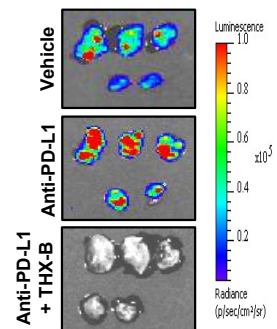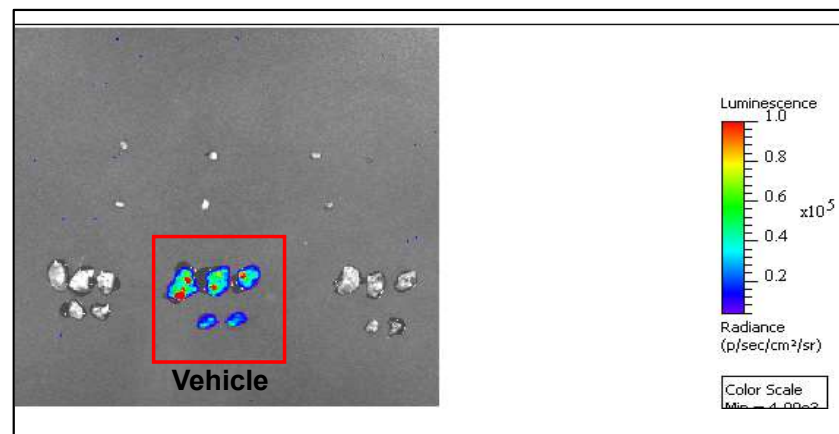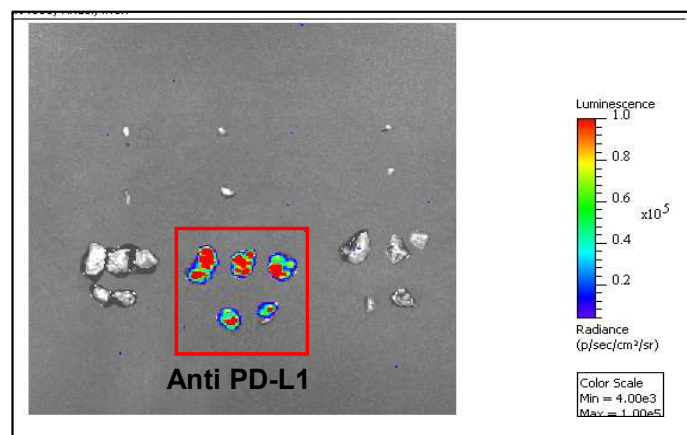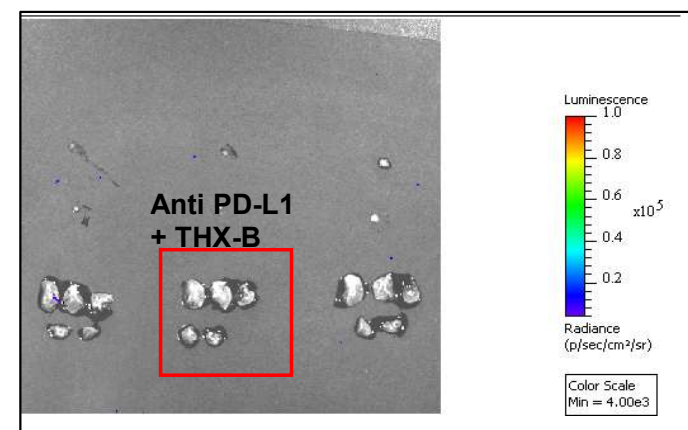

Supplement: Supplementary file 12 — Figure EV2 Source Data [file 44318_2026_803_MOESM12_ESM.zip › Fig EV2/EV2F/EV2F-Readme.pdf]

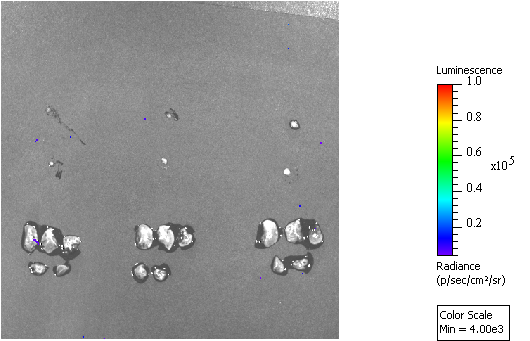

Supplement: Supplementary file 12 — Figure EV2 Source Data [file 44318_2026_803_MOESM12_ESM.zip › Fig EV2/EV2F/F10 R31 Anti PD-L1 THX-B.tif]

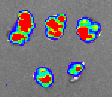

Supplement: Supplementary file 12 — Figure EV2 Source Data [file 44318_2026_803_MOESM12_ESM.zip › Fig EV2/EV2F/F10 R31 Anti PD-L1-1.tif]

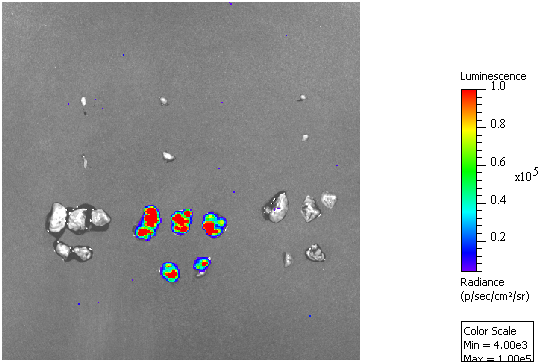

Supplement: Supplementary file 12 — Figure EV2 Source Data [file 44318_2026_803_MOESM12_ESM.zip › Fig EV2/EV2F/F10 R31 Anti PD-L1.tif]

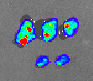

Supplement: Supplementary file 12 — Figure EV2 Source Data [file 44318_2026_803_MOESM12_ESM.zip › Fig EV2/EV2F/F10 R31 Vehicle-1.tif]

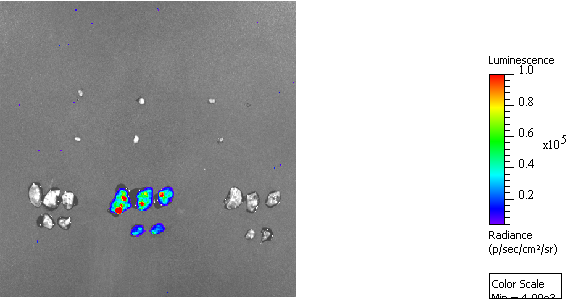

Supplement: Supplementary file 12 — Figure EV2 Source Data [file 44318_2026_803_MOESM12_ESM.zip › Fig EV2/EV2F/F10 R31 Vehicle.tif]

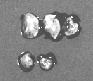

Supplement: Supplementary file 12 — Figure EV2 Source Data [file 44318_2026_803_MOESM12_ESM.zip › Fig EV2/EV2F/F10-R31 Combo.tif]

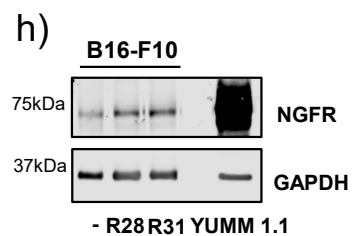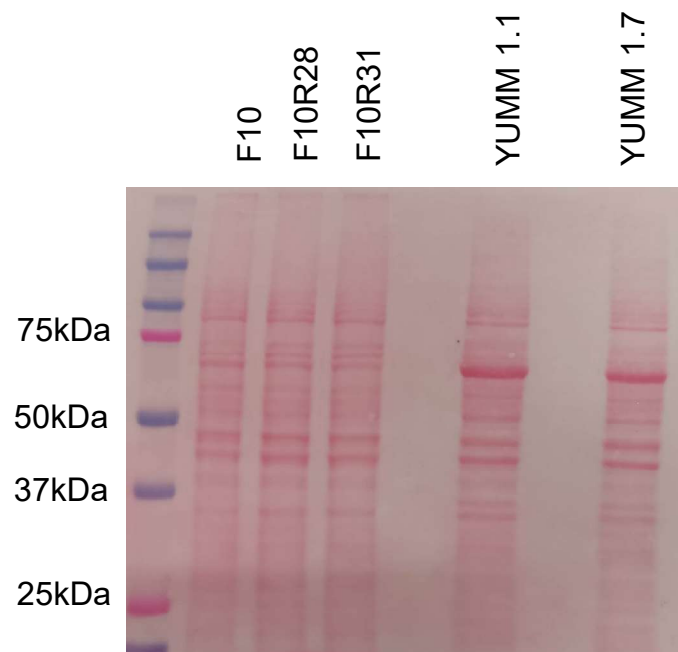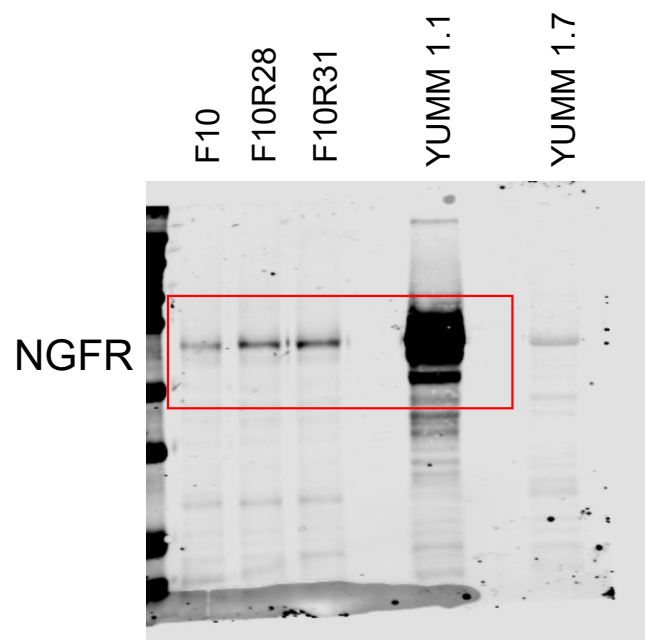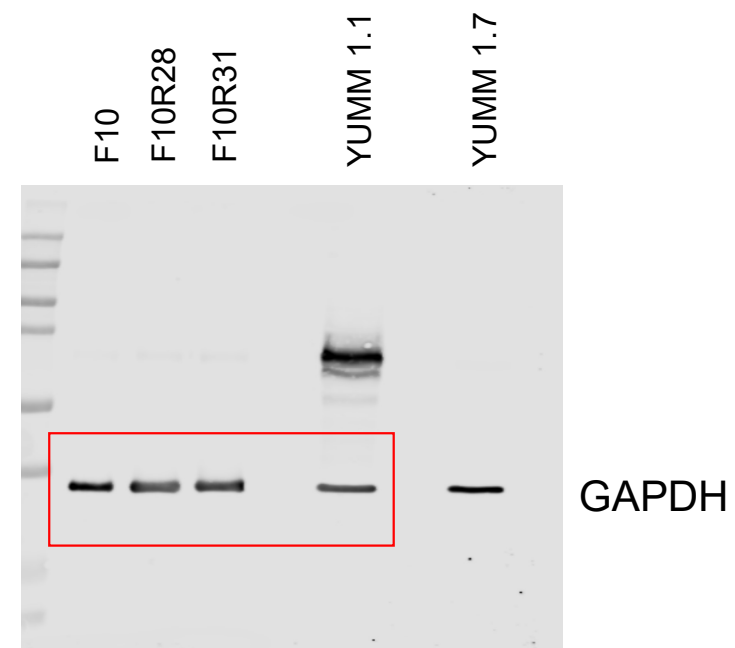

Supplement: Supplementary file 12 — Figure EV2 Source Data [file 44318_2026_803_MOESM12_ESM.zip › Fig EV2/EV2H/EV2H-Readme.pdf]

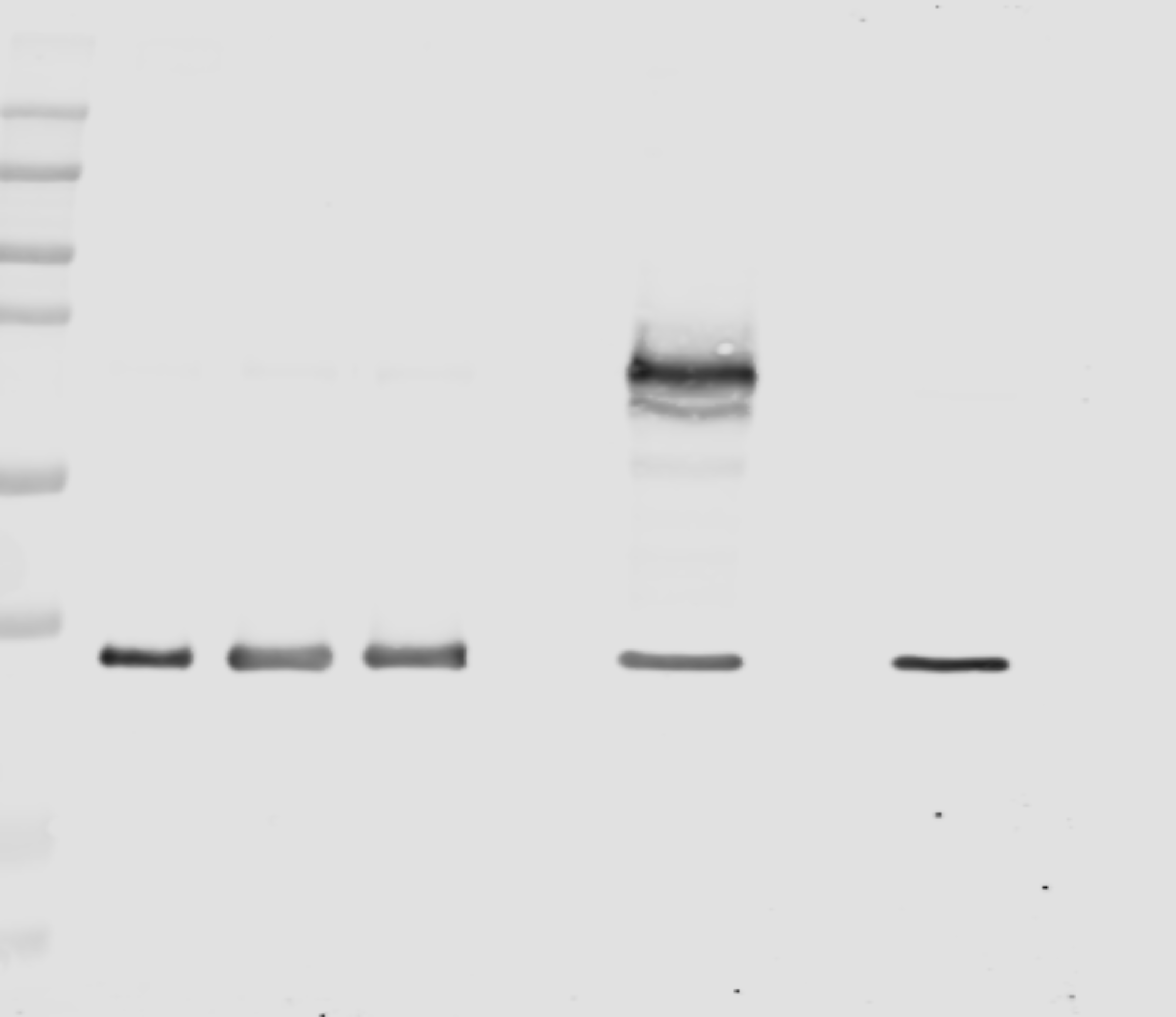

Supplement: Supplementary file 12 — Figure EV2 Source Data [file 44318_2026_803_MOESM12_ESM.zip › Fig EV2/EV2H/GAPDH F10R Y YUMM.tif]

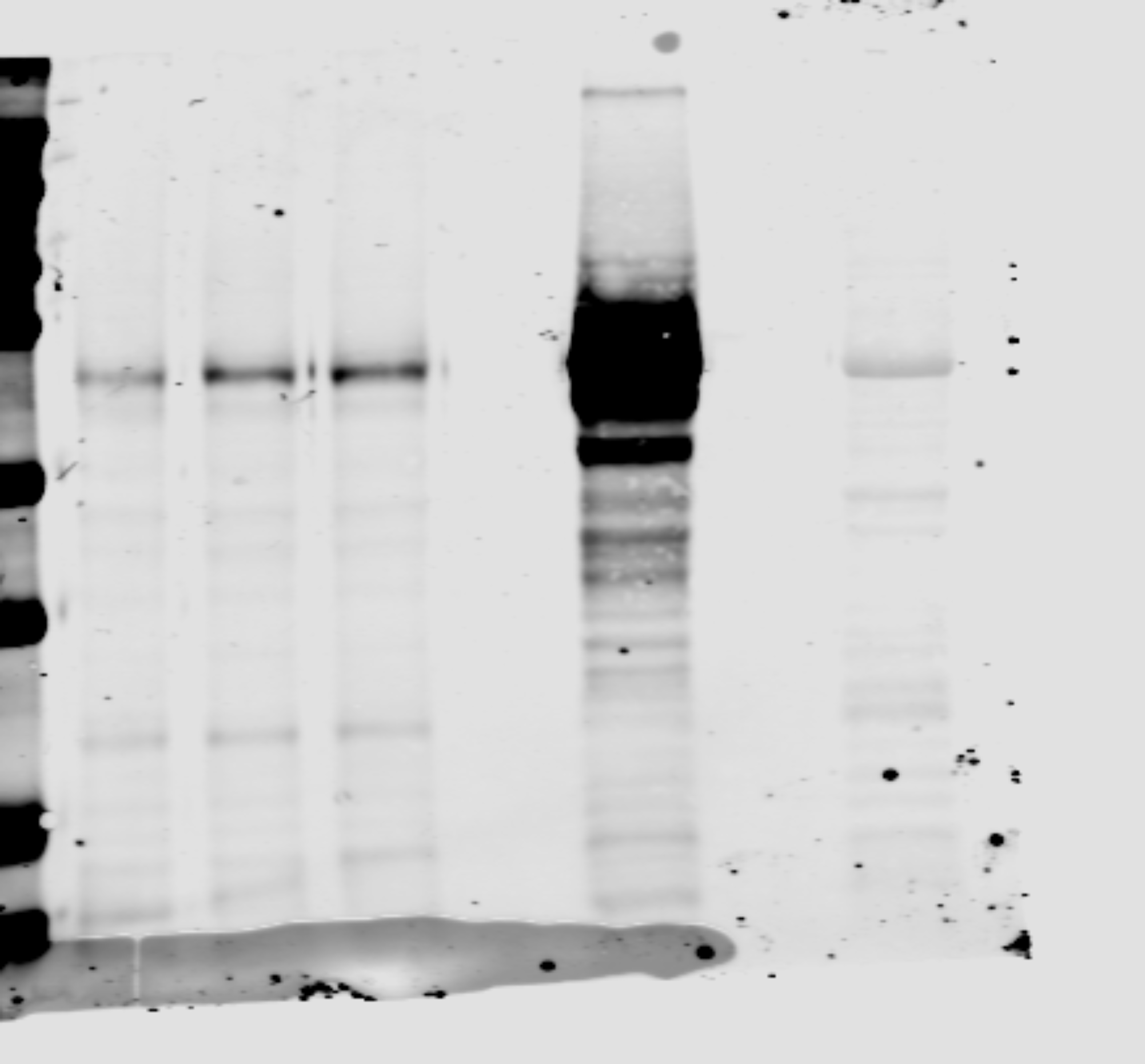

Supplement: Supplementary file 12 — Figure EV2 Source Data [file 44318_2026_803_MOESM12_ESM.zip › Fig EV2/EV2H/NGFR F10R y YUMM.tif]

i)

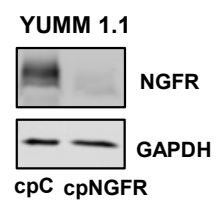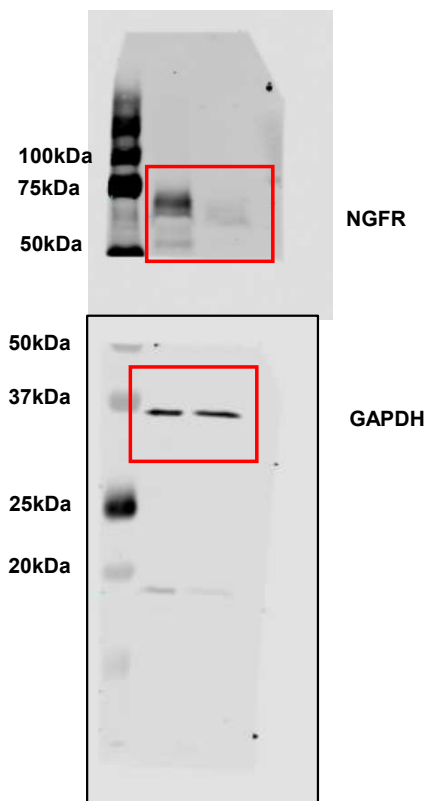

Supplement: Supplementary file 12 — Figure EV2 Source Data [file 44318_2026_803_MOESM12_ESM.zip › Fig EV2/EV2I/EV2I-Readme.pdf]

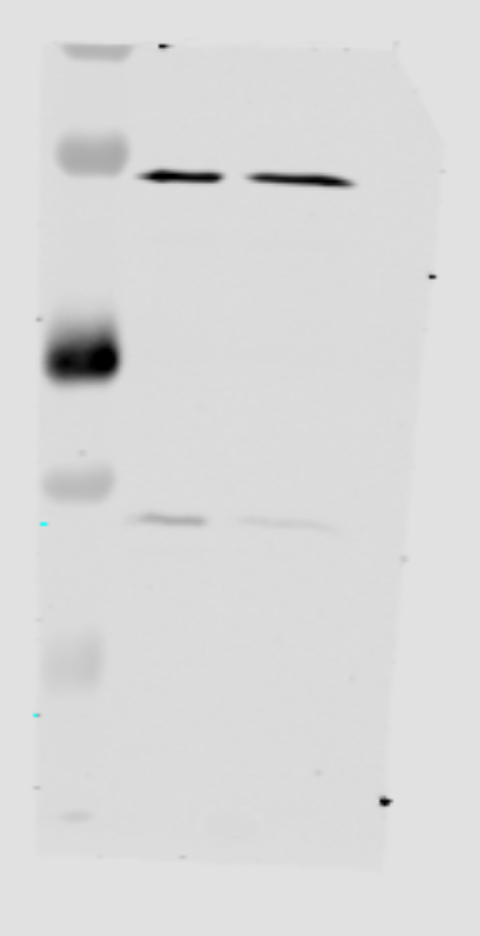

Supplement: Supplementary file 12 — Figure EV2 Source Data [file 44318_2026_803_MOESM12_ESM.zip › Fig EV2/EV2I/GAPDH.tif]

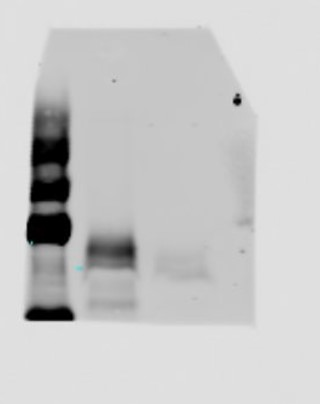

Supplement: Supplementary file 12 — Figure EV2 Source Data [file 44318_2026_803_MOESM12_ESM.zip › Fig EV2/EV2I/NGFR.tif]

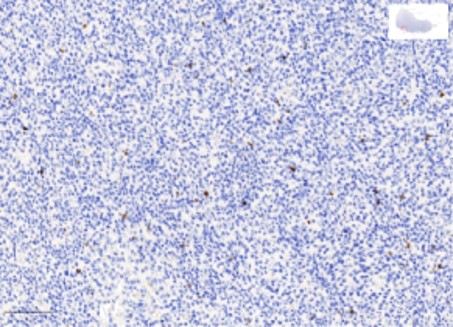

Supplement: Supplementary file 12 — Figure EV2 Source Data [file 44318_2026_803_MOESM12_ESM.zip › Fig EV2/EV2K/cpC.tif]

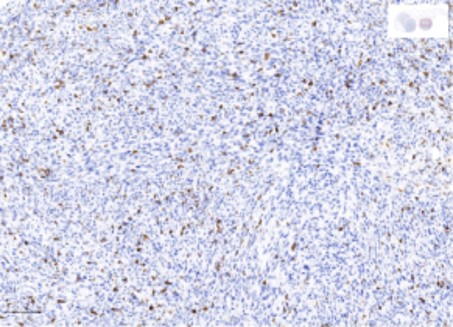

Supplement: Supplementary file 12 — Figure EV2 Source Data [file 44318_2026_803_MOESM12_ESM.zip › Fig EV2/EV2K/cpNGFR.tif]

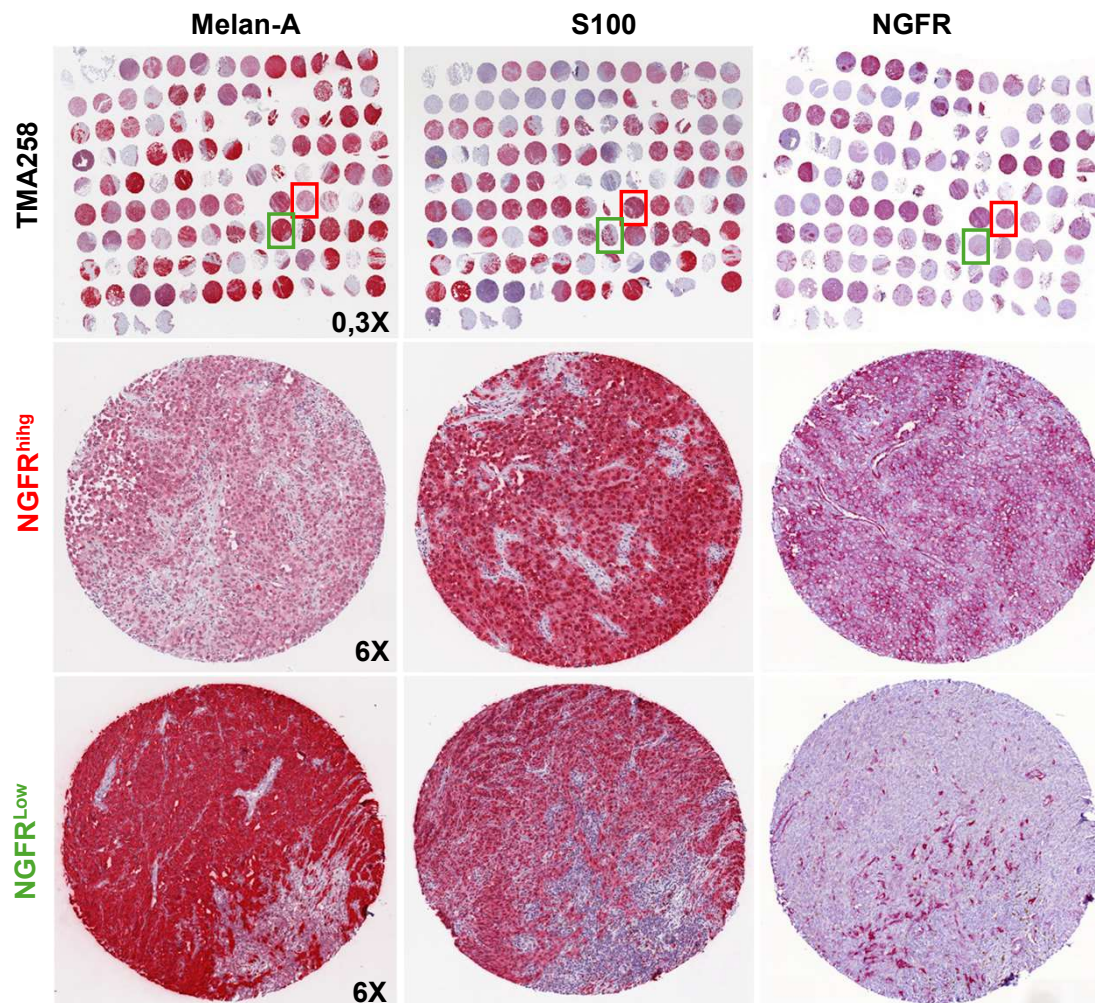

Supplement: Supplementary file 12 — Figure EV2 Source Data [file 44318_2026_803_MOESM12_ESM.zip › Fig EV2/EV2N/EV2M-Readme.pdf]

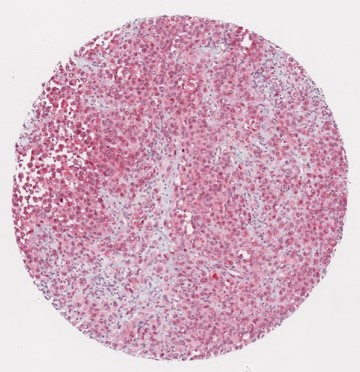

Supplement: Supplementary file 12 — Figure EV2 Source Data [file 44318_2026_803_MOESM12_ESM.zip › Fig EV2/EV2N/MelanA-NGFRHigh.tif]

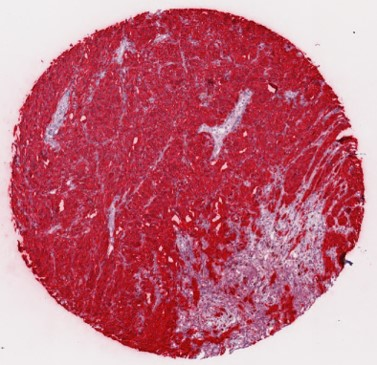

Supplement: Supplementary file 12 — Figure EV2 Source Data [file 44318_2026_803_MOESM12_ESM.zip › Fig EV2/EV2N/MelanA-NGFRLow.tif]

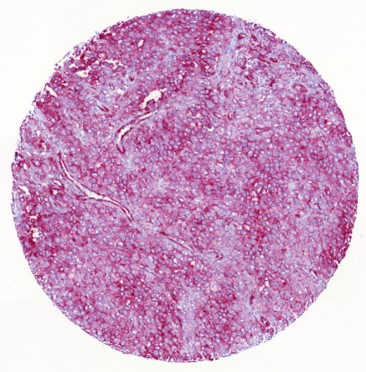

Supplement: Supplementary file 12 — Figure EV2 Source Data [file 44318_2026_803_MOESM12_ESM.zip › Fig EV2/EV2N/NGFR High.tif]

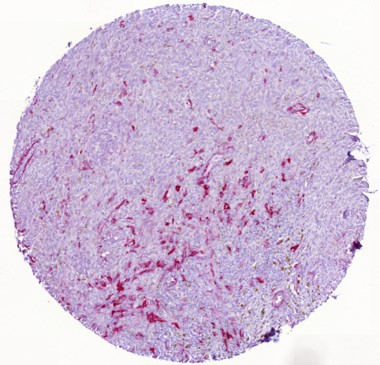

Supplement: Supplementary file 12 — Figure EV2 Source Data [file 44318_2026_803_MOESM12_ESM.zip › Fig EV2/EV2N/NGFR-Low.tif]

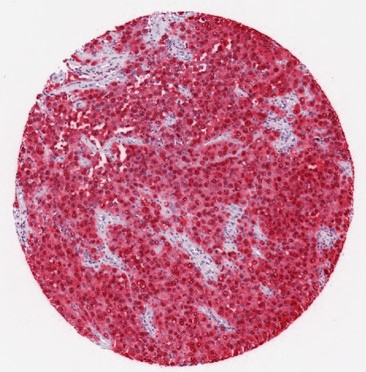

Supplement: Supplementary file 12 — Figure EV2 Source Data [file 44318_2026_803_MOESM12_ESM.zip › Fig EV2/EV2N/S100-NGFRHigh.tif]

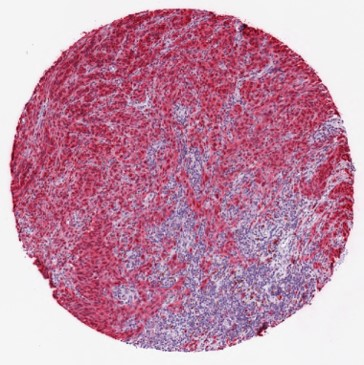

Supplement: Supplementary file 12 — Figure EV2 Source Data [file 44318_2026_803_MOESM12_ESM.zip › Fig EV2/EV2N/S100-NGFRLow.tif]

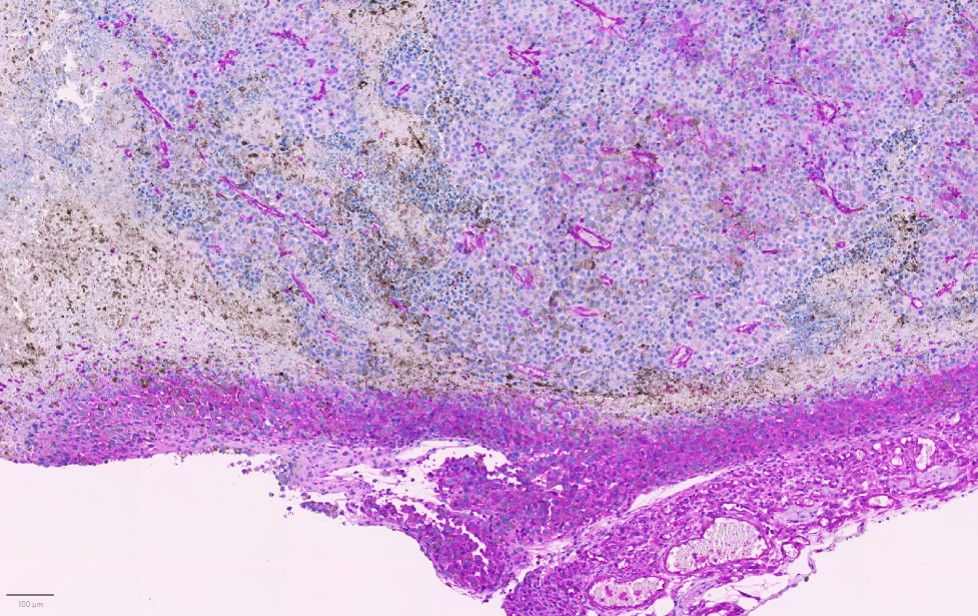

Supplement: Supplementary file 13 — Figure EV3 Source Data [file 44318_2026_803_MOESM13_ESM.zip › Fig EV3/EV3A/pMLC2-F10.tif]

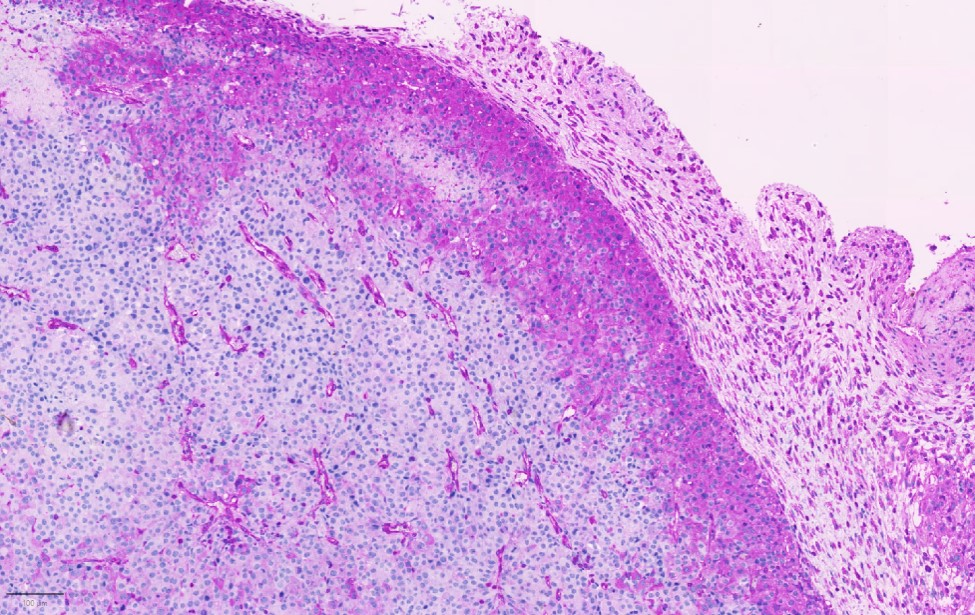

Supplement: Supplementary file 13 — Figure EV3 Source Data [file 44318_2026_803_MOESM13_ESM.zip › Fig EV3/EV3A/pMLC2-R28.tif]

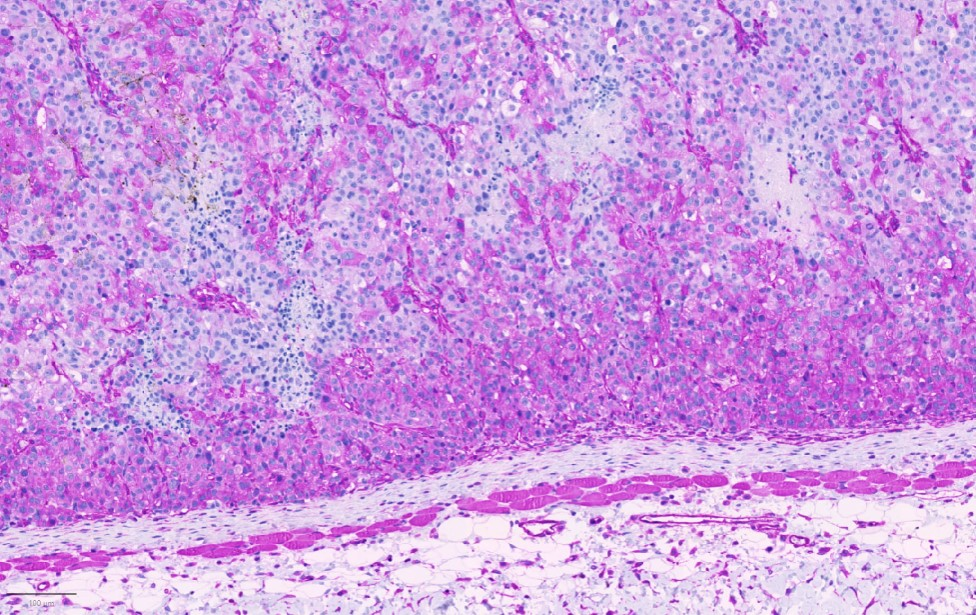

Supplement: Supplementary file 13 — Figure EV3 Source Data [file 44318_2026_803_MOESM13_ESM.zip › Fig EV3/EV3A/pMLC2-R31.tif]

A375P

|  |       |
|--|-------|
|  | NGFR  |
|  | GAPDH |

cpC  
cpNGFR1  
cpNGFR2

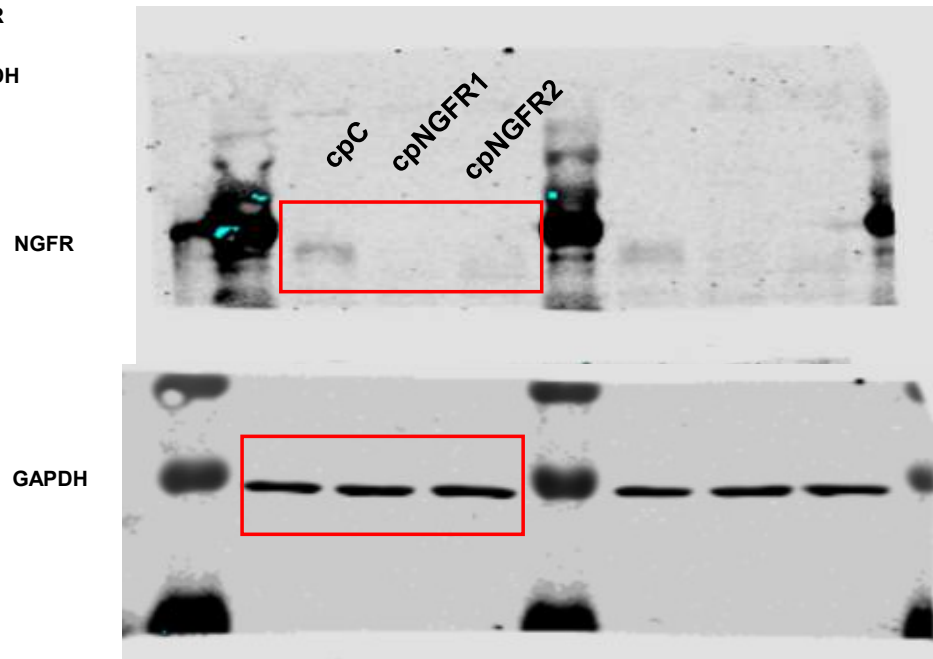

100kDa  
75kDa  
50kDa  
37kDa  
25kDa  
20kDa  
15kDa

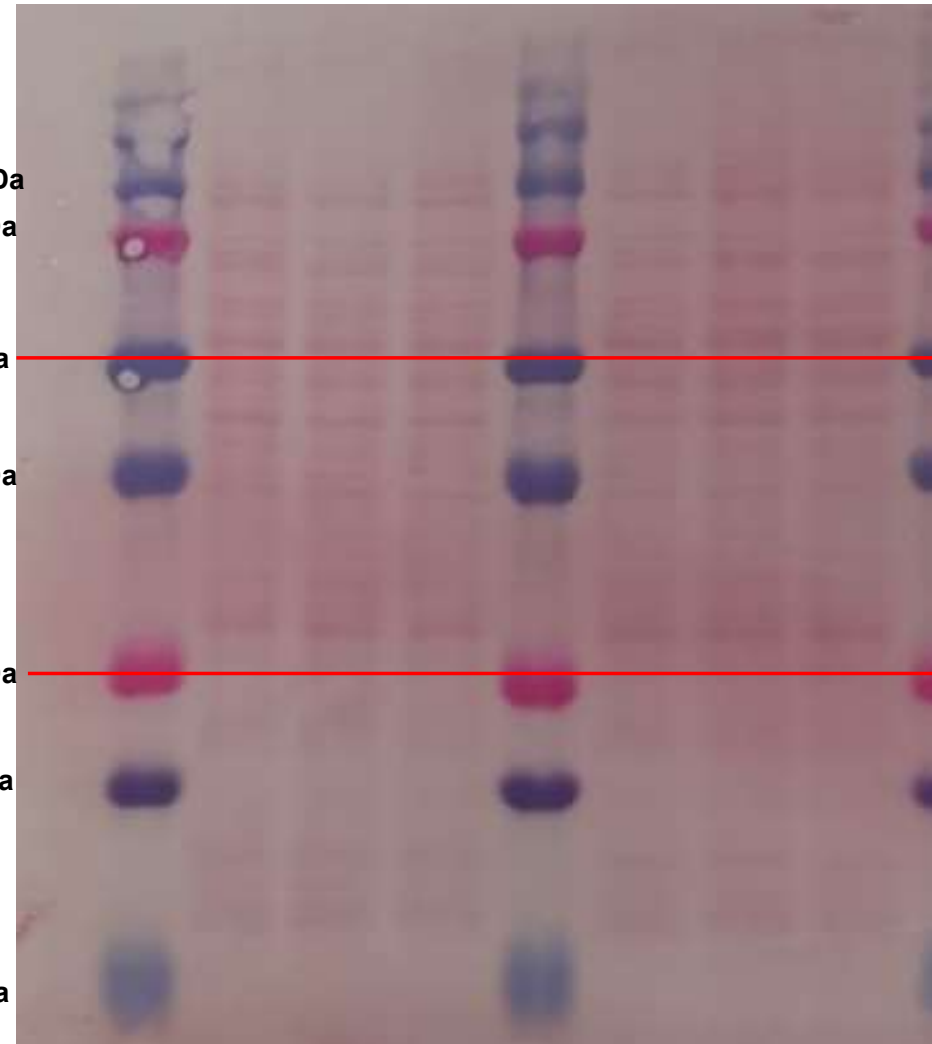

Supplement: Supplementary file 13 — Figure EV3 Source Data [file 44318_2026_803_MOESM13_ESM.zip › Fig EV3/EV3E/EV3E-Readme.pdf]

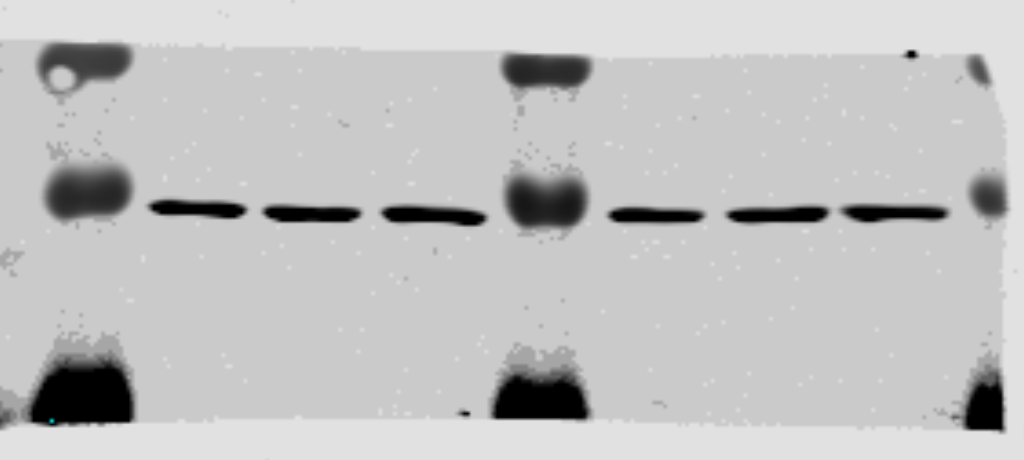

Supplement: Supplementary file 13 — Figure EV3 Source Data [file 44318_2026_803_MOESM13_ESM.zip › Fig EV3/EV3E/GAPDH.tif]

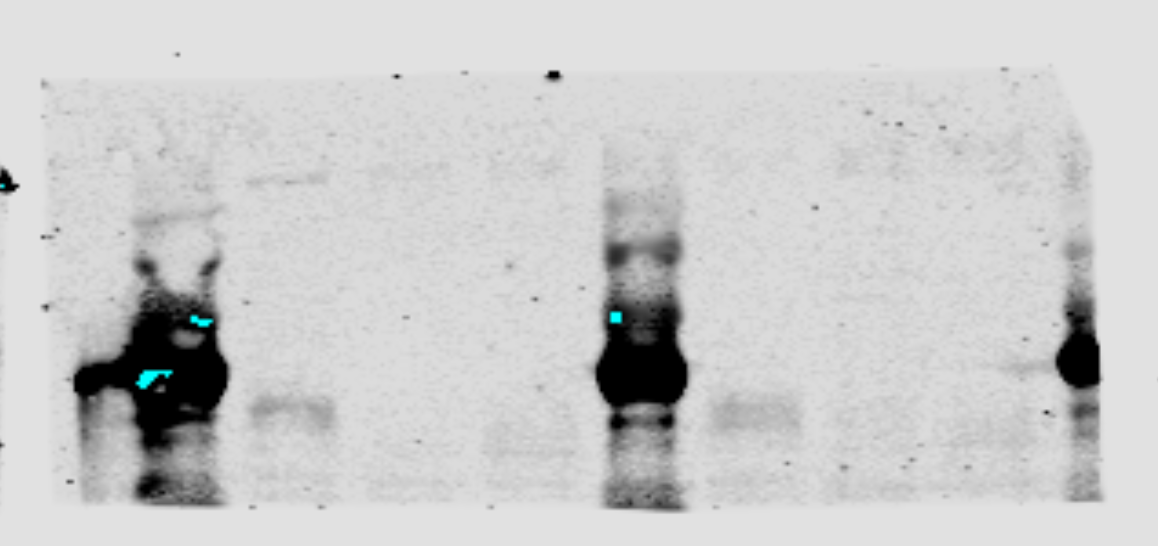

Supplement: Supplementary file 13 — Figure EV3 Source Data [file 44318_2026_803_MOESM13_ESM.zip › Fig EV3/EV3E/NGFR.tif]

f)

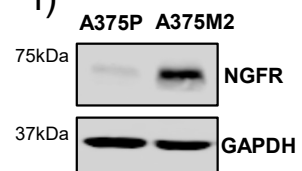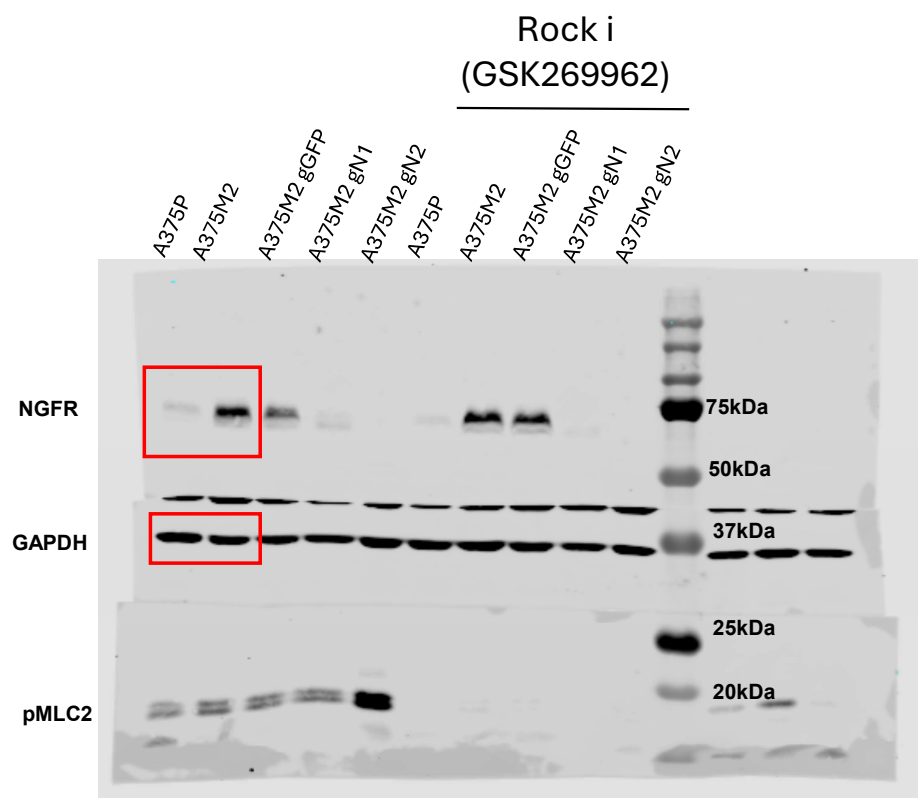

Supplement: Supplementary file 13 — Figure EV3 Source Data [file 44318_2026_803_MOESM13_ESM.zip › Fig EV3/EV3F/EV3F-Readme.pdf]

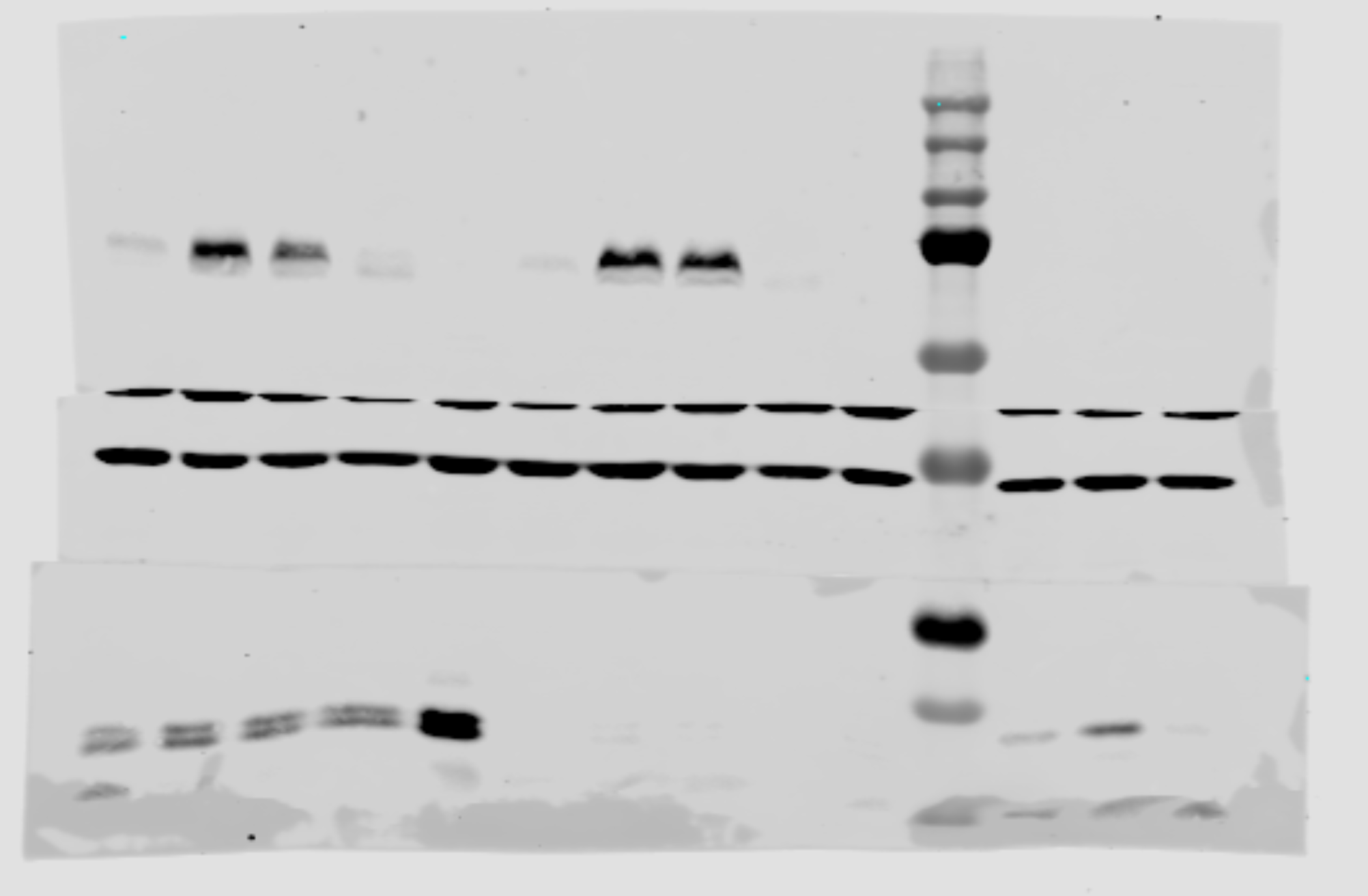

Supplement: Supplementary file 13 — Figure EV3 Source Data [file 44318_2026_803_MOESM13_ESM.zip › Fig EV3/EV3F/NGFR _GAPDH_pMLC2.tif]
