# Supplementary figures and images for: NGFR induces melanoma invasion and immunotherapy resistance through myosin light chain 2 modulation (part 3 of 3)
Source: EMBO J. 2026 May 26;45(14):4988–5023. doi: 10.1038/s44318-026-00803-2 (PMC13373201; doi:10.1038/s44318-026-00803-2)

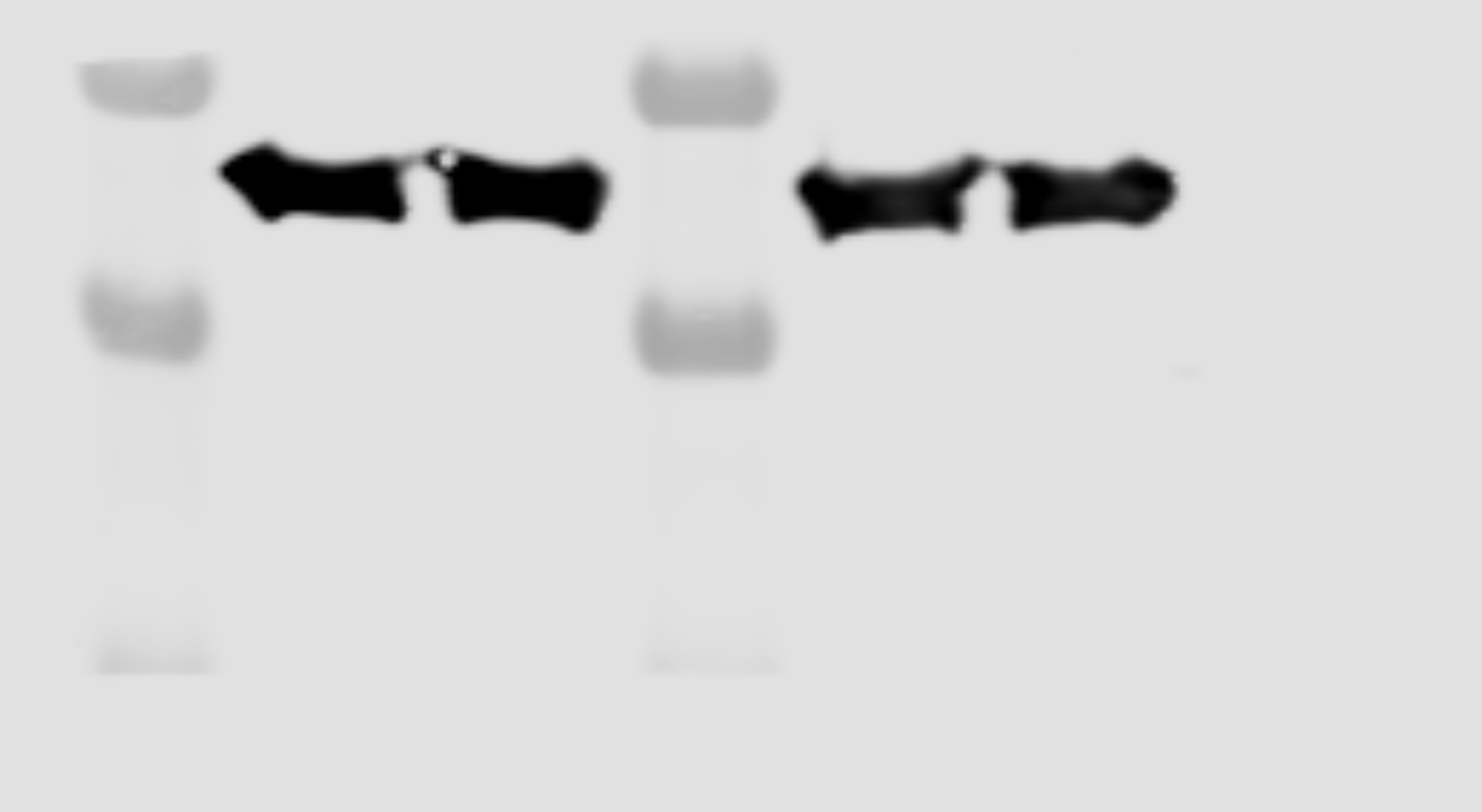

Supplement: Supplementary file 13 — Figure EV3 Source Data [file 44318_2026_803_MOESM13_ESM.zip › Fig EV3/EV3G/act.tif]

Downmodulation of p-MLC2 by THX-B by wb

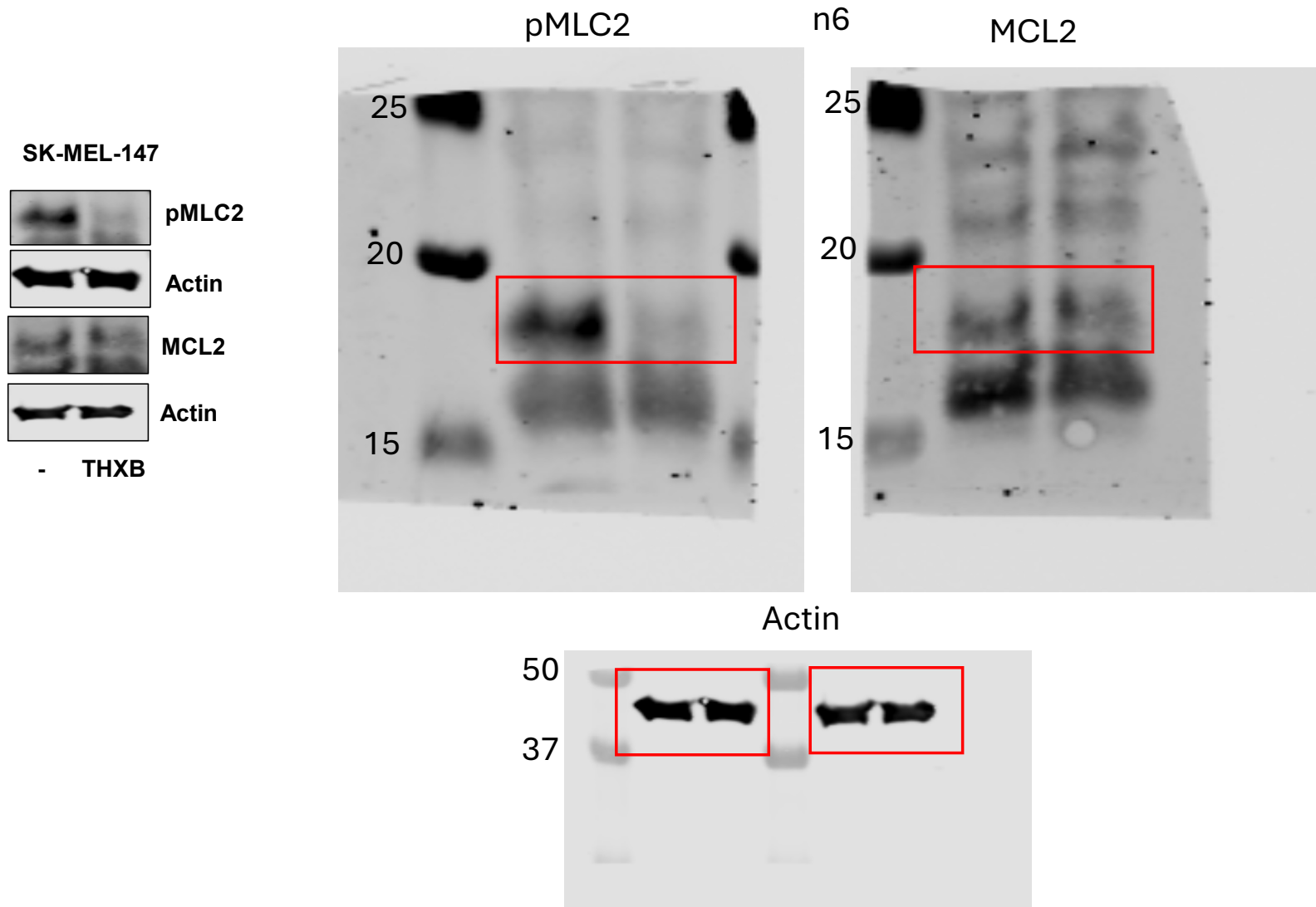

Supplement: Supplementary file 13 — Figure EV3 Source Data [file 44318_2026_803_MOESM13_ESM.zip › Fig EV3/EV3G/EV3G-Readme.pdf]

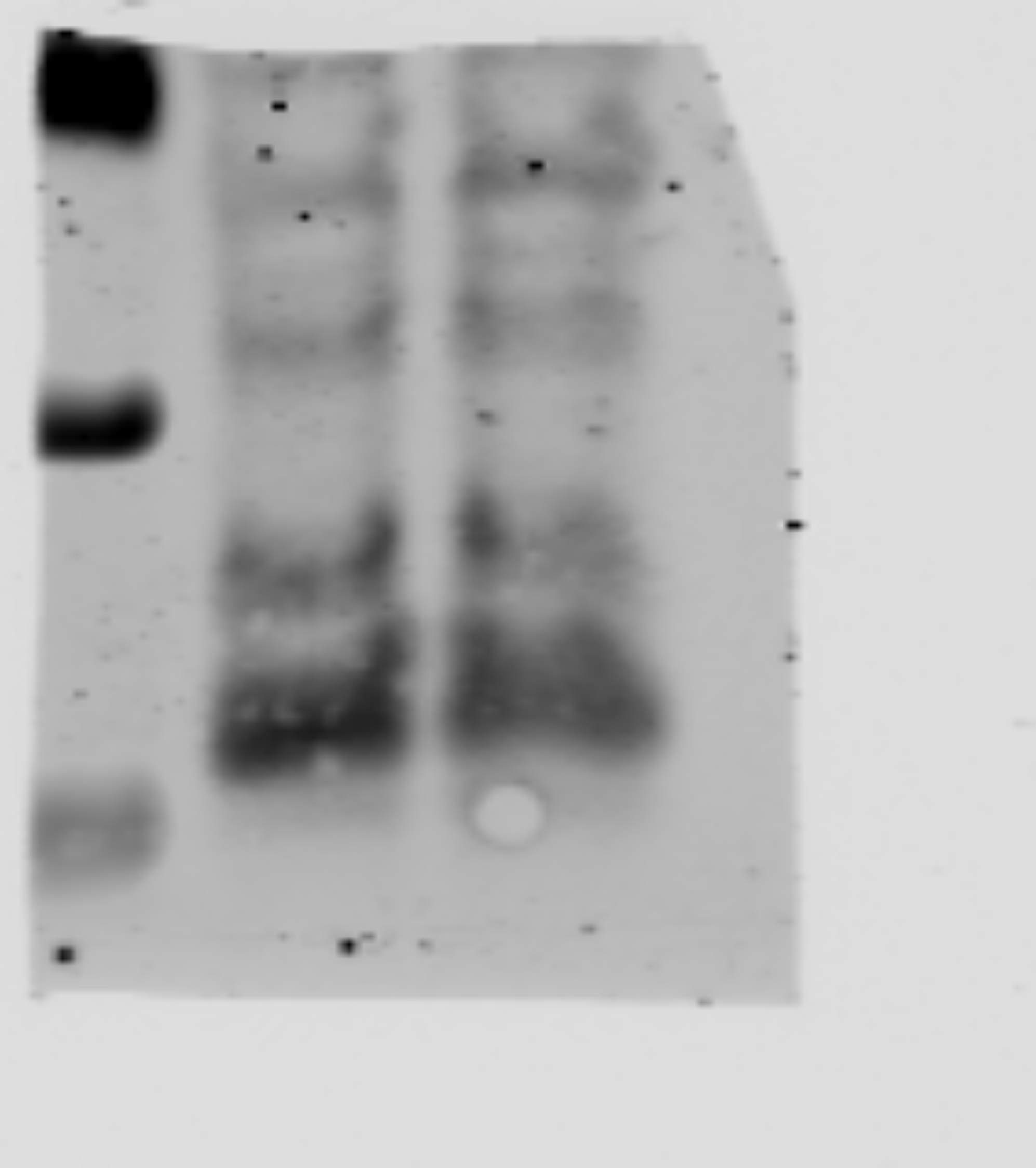

Supplement: Supplementary file 13 — Figure EV3 Source Data [file 44318_2026_803_MOESM13_ESM.zip › Fig EV3/EV3G/myo tot.tif]

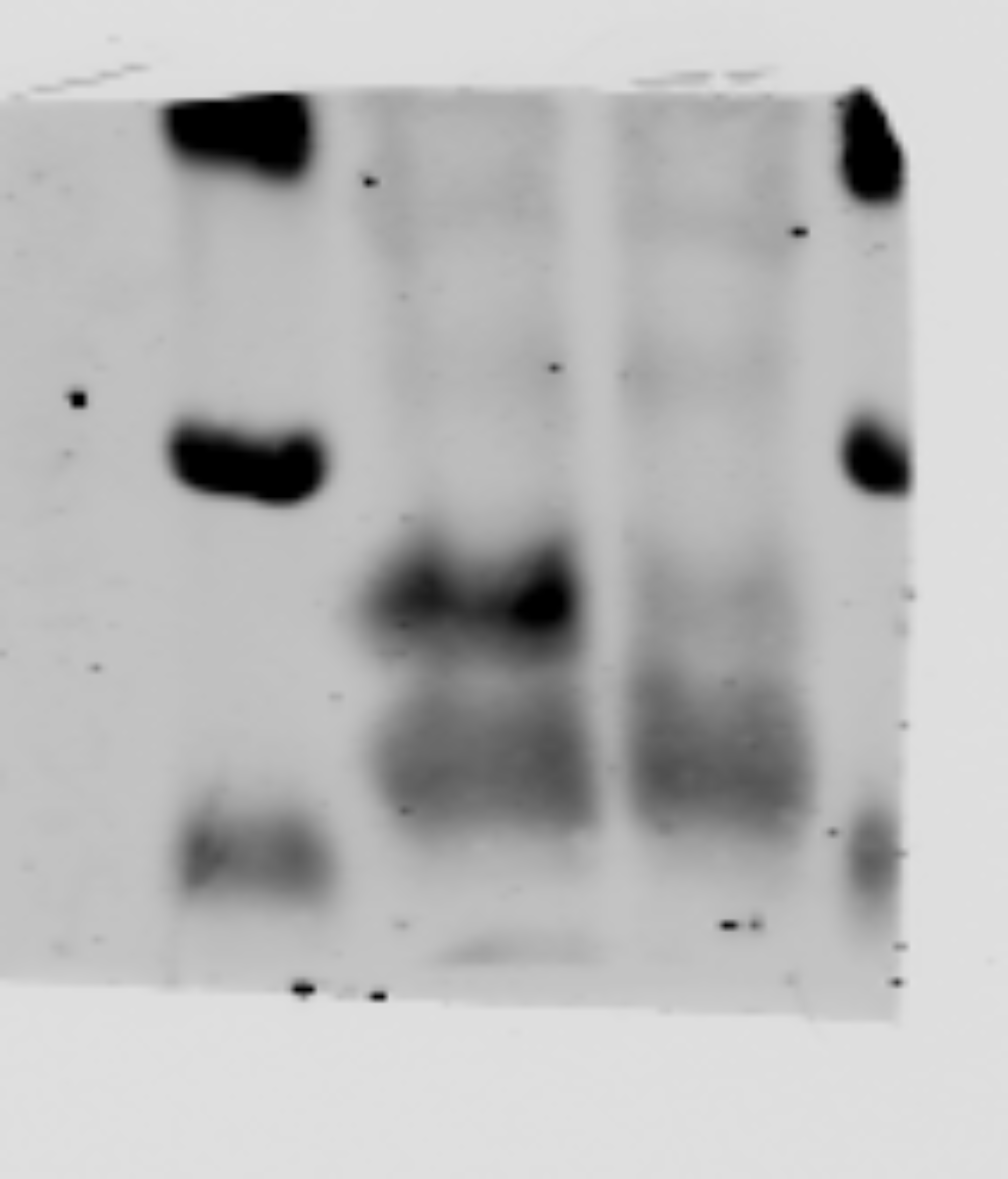

Supplement: Supplementary file 13 — Figure EV3 Source Data [file 44318_2026_803_MOESM13_ESM.zip › Fig EV3/EV3G/pMyo.tif]

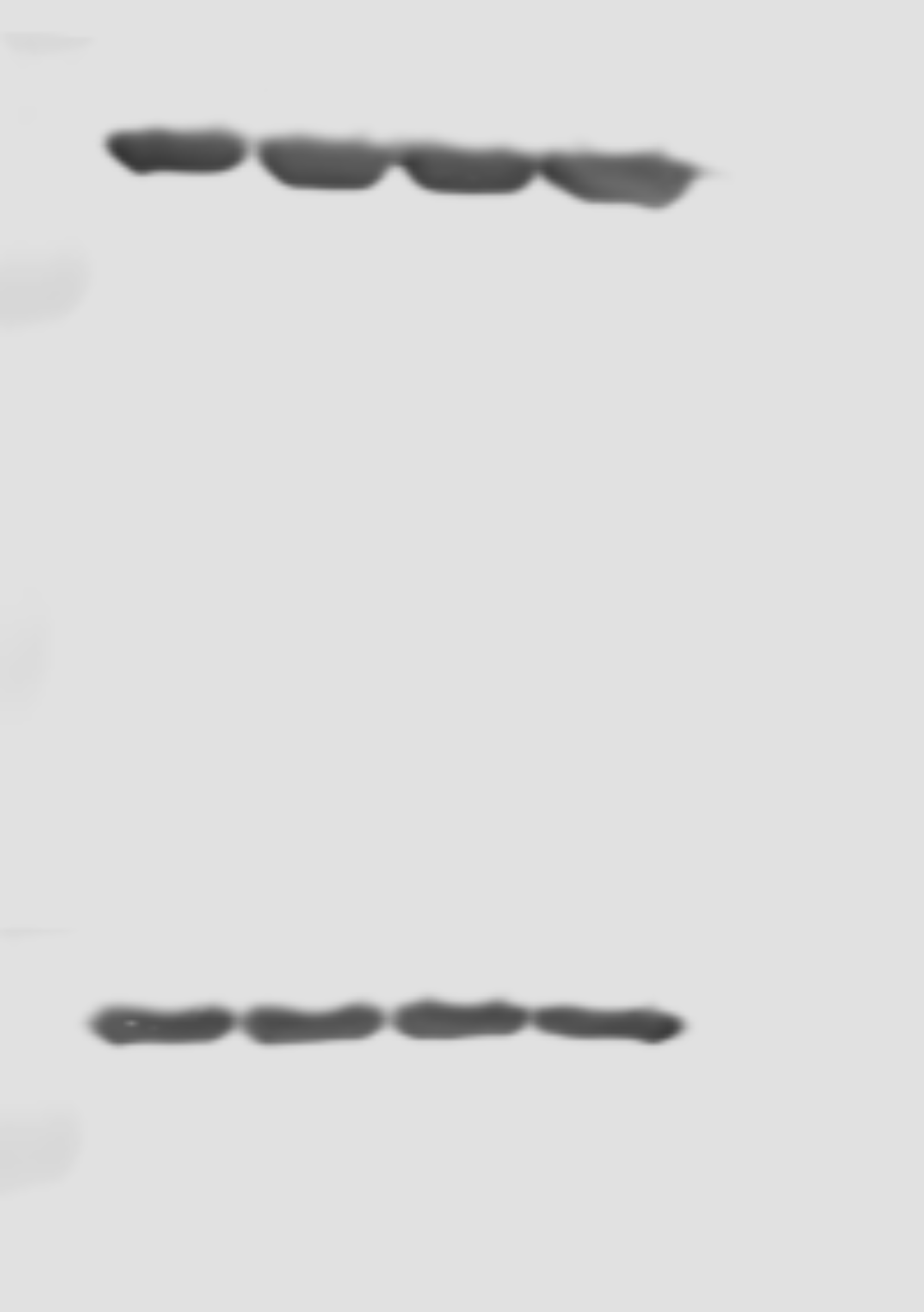

Supplement: Supplementary file 13 — Figure EV3 Source Data [file 44318_2026_803_MOESM13_ESM.zip › Fig EV3/EV3I/Actin.tif]

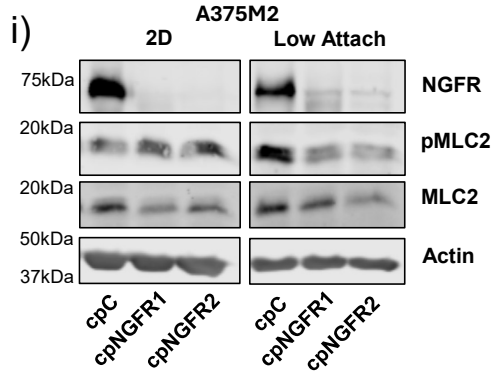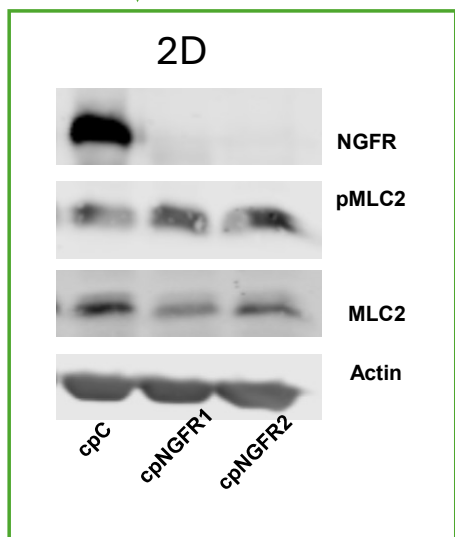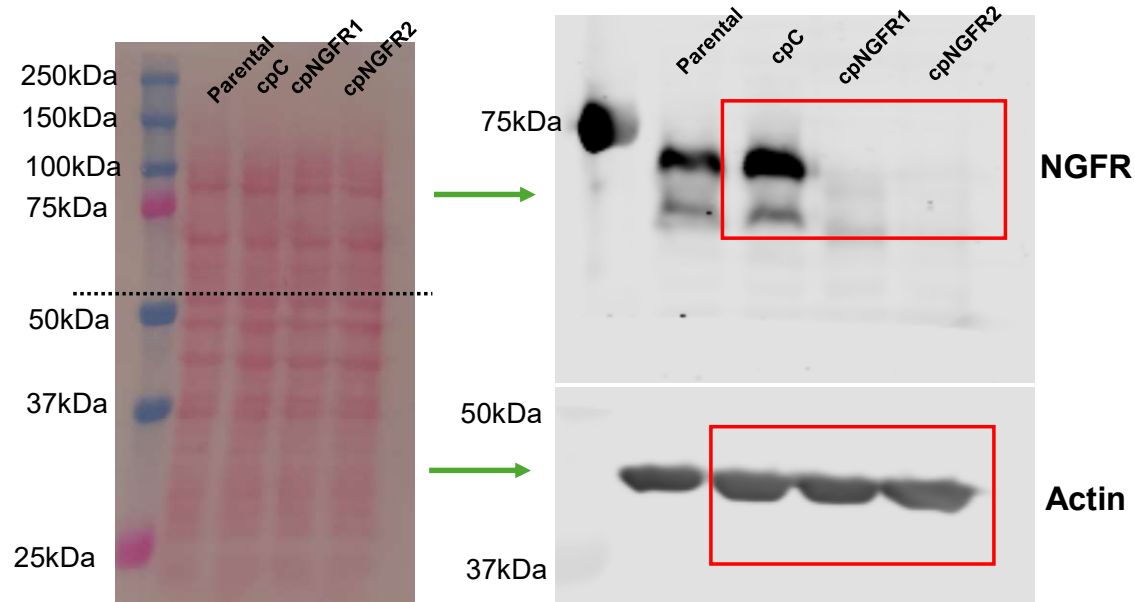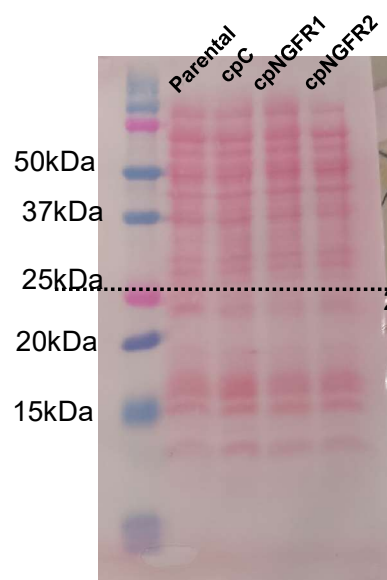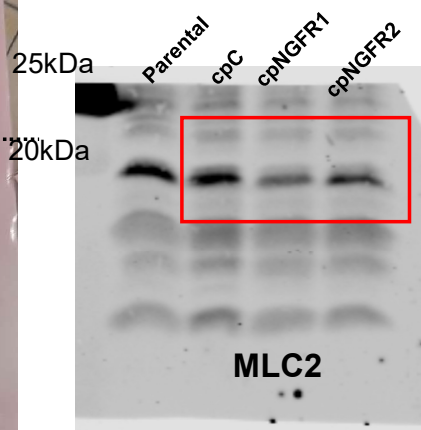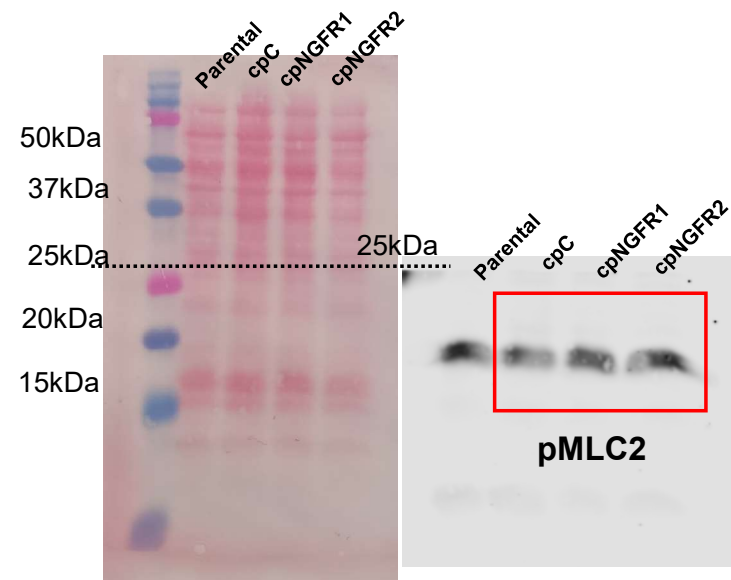

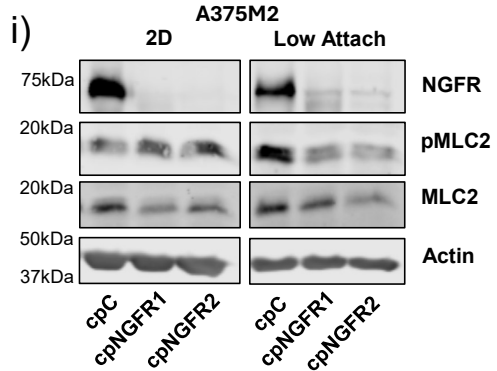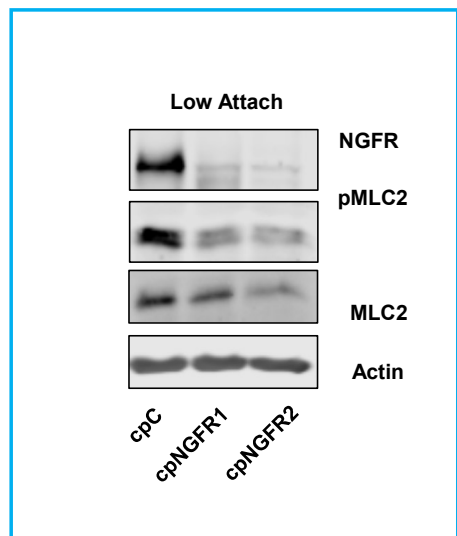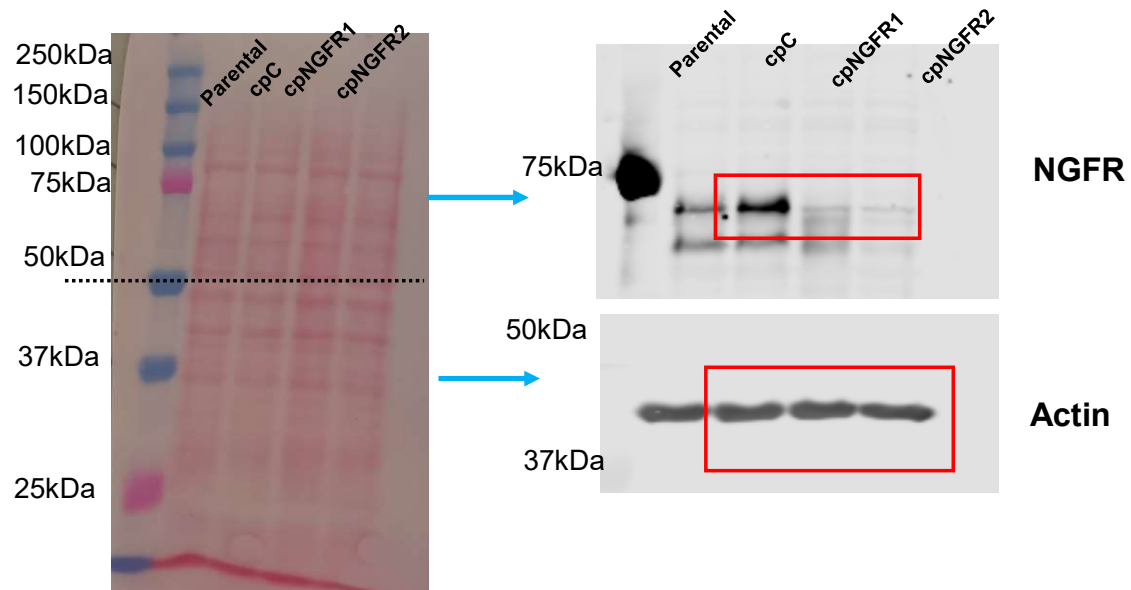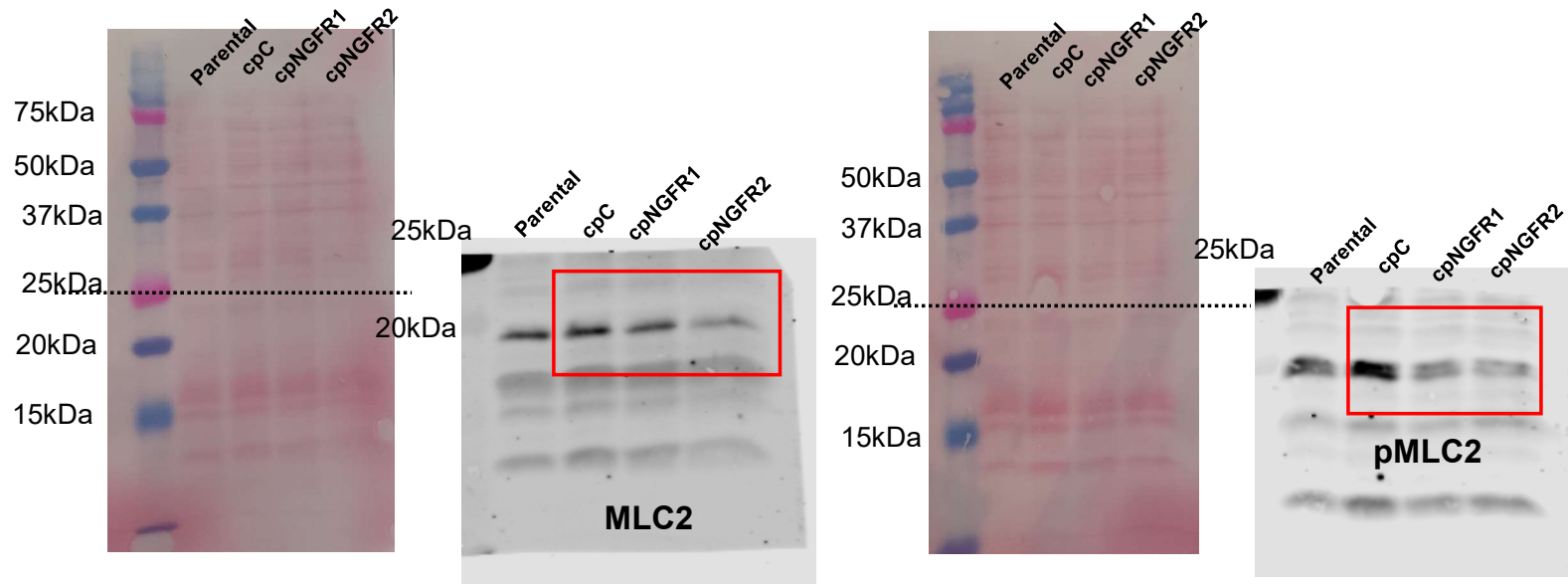

Supplement: Supplementary file 13 — Figure EV3 Source Data [file 44318_2026_803_MOESM13_ESM.zip › Fig EV3/EV3I/EV3I-Readme.pdf]

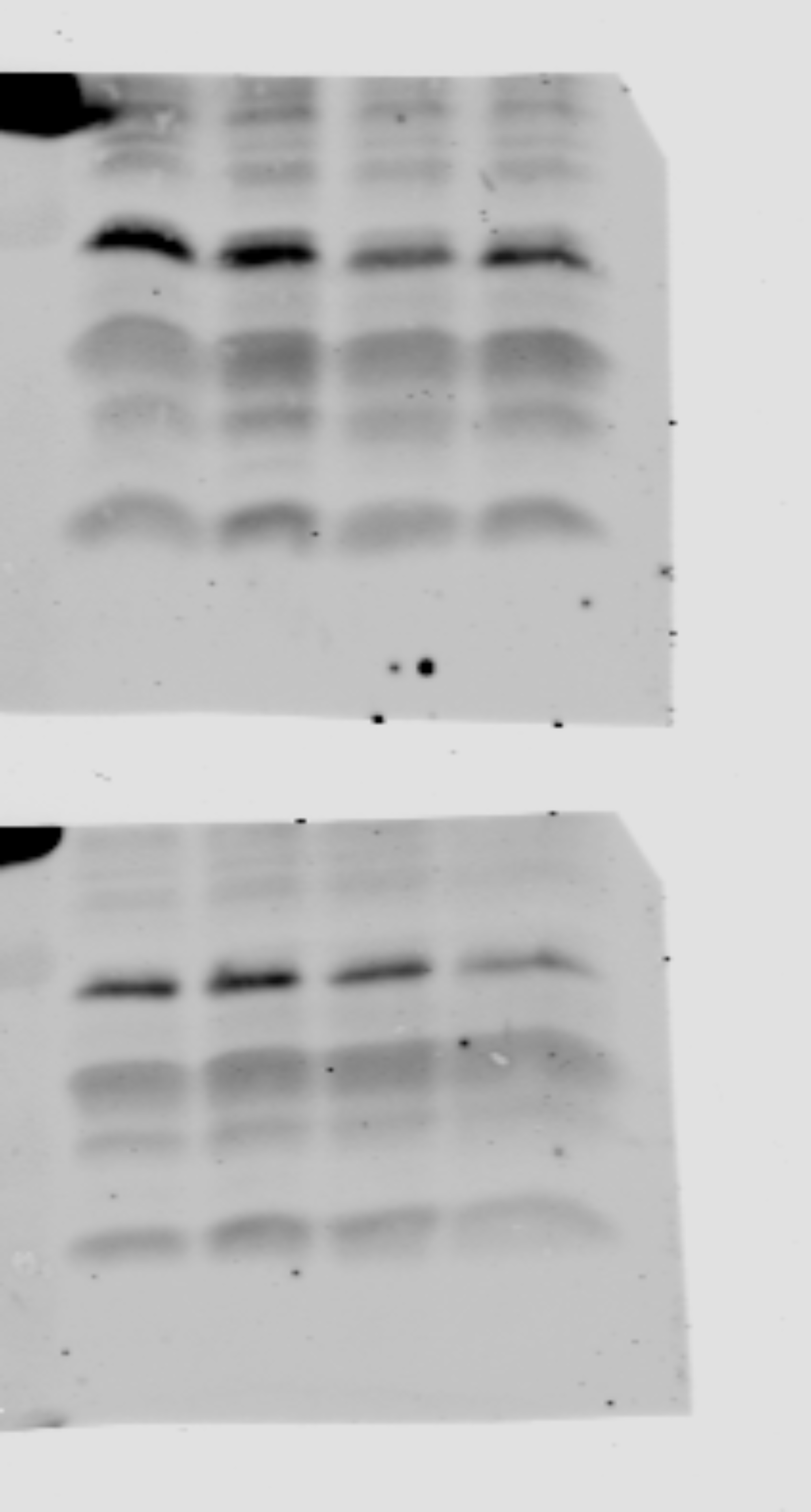

Supplement: Supplementary file 13 — Figure EV3 Source Data [file 44318_2026_803_MOESM13_ESM.zip › Fig EV3/EV3I/MLC2 2D vs low adh.tif]

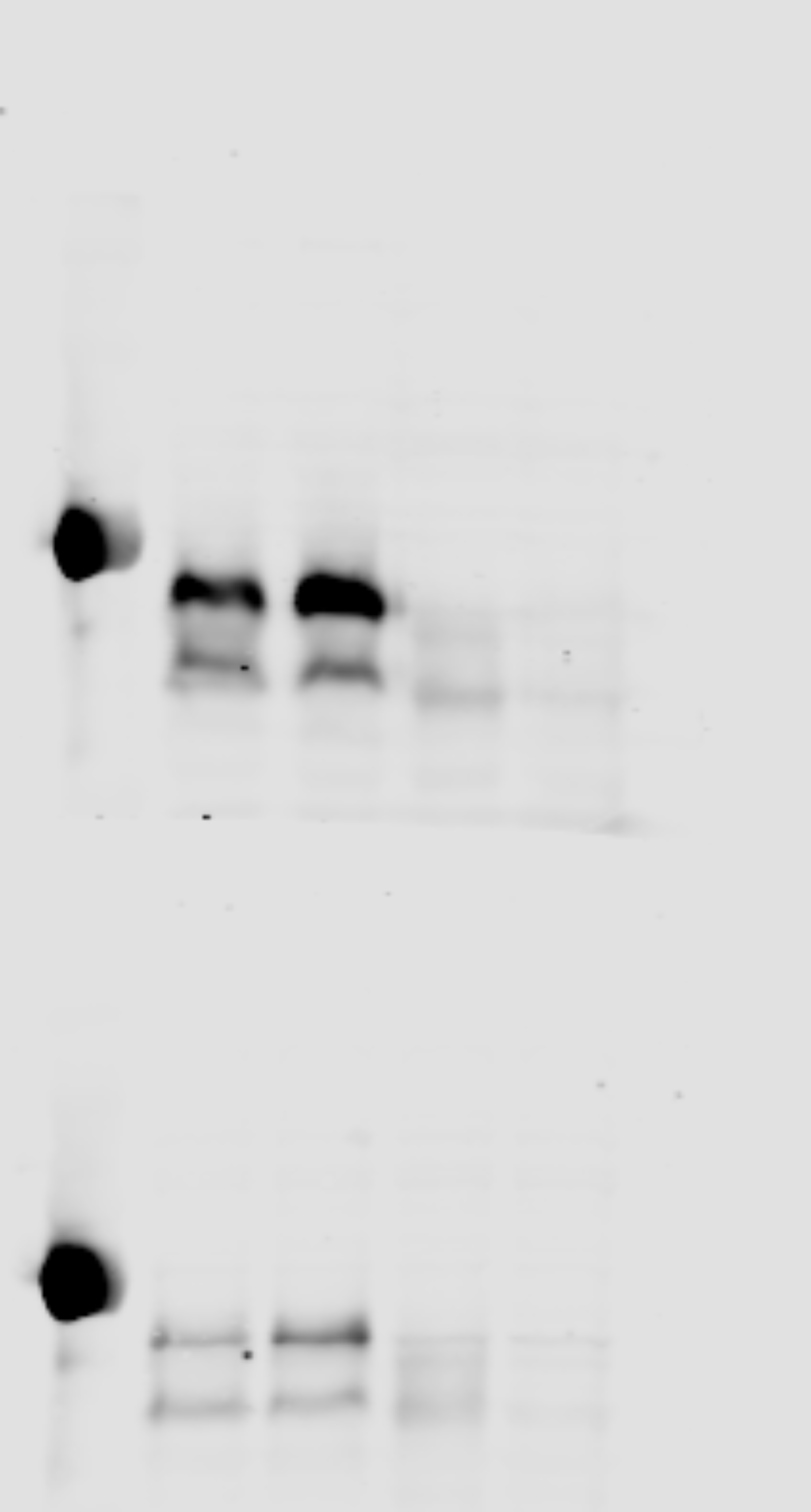

Supplement: Supplementary file 13 — Figure EV3 Source Data [file 44318_2026_803_MOESM13_ESM.zip › Fig EV3/EV3I/NGFR.tif]

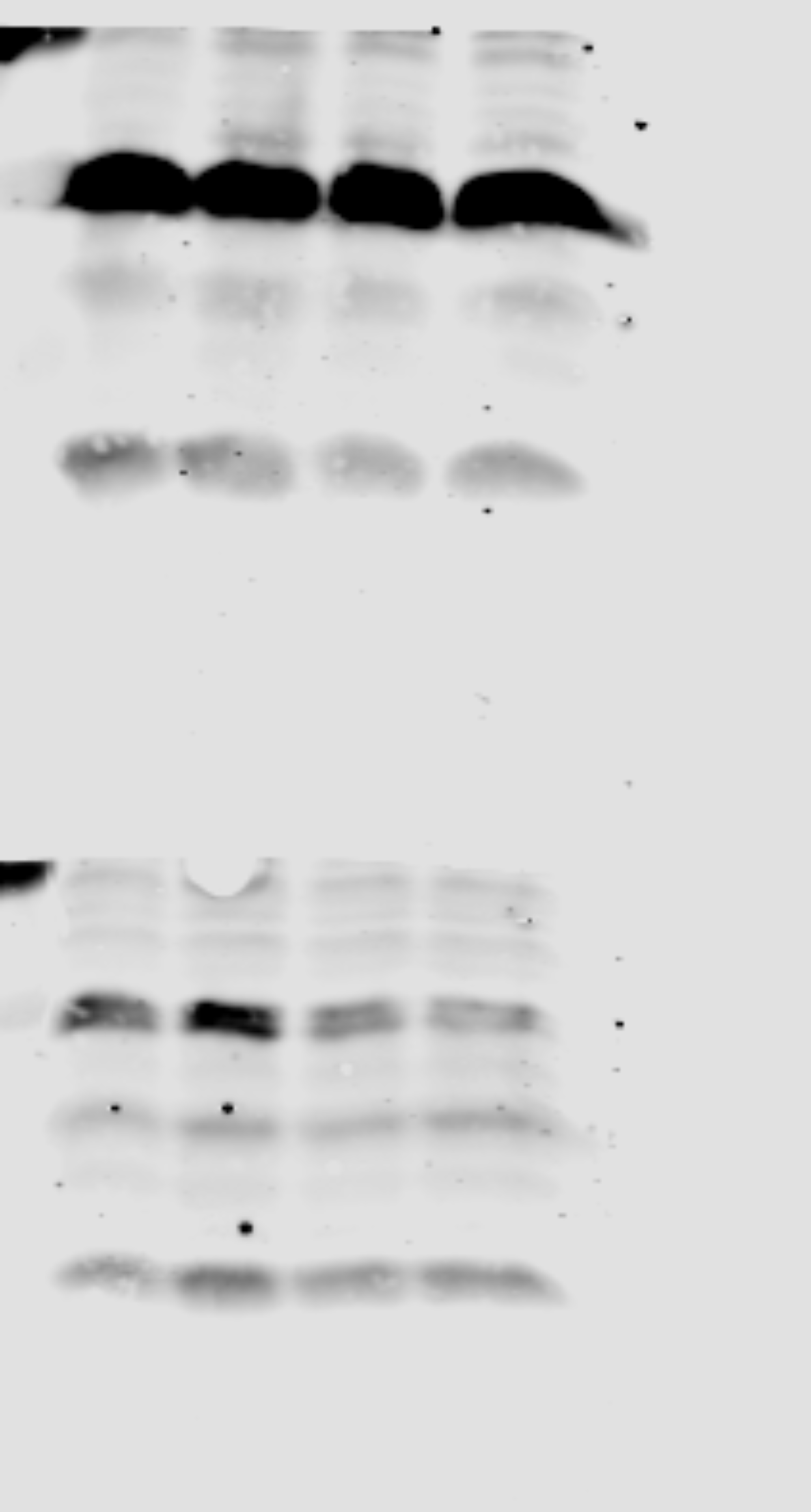

Supplement: Supplementary file 13 — Figure EV3 Source Data [file 44318_2026_803_MOESM13_ESM.zip › Fig EV3/EV3I/pMLC2 2D vs low adh_high exposure.tif]

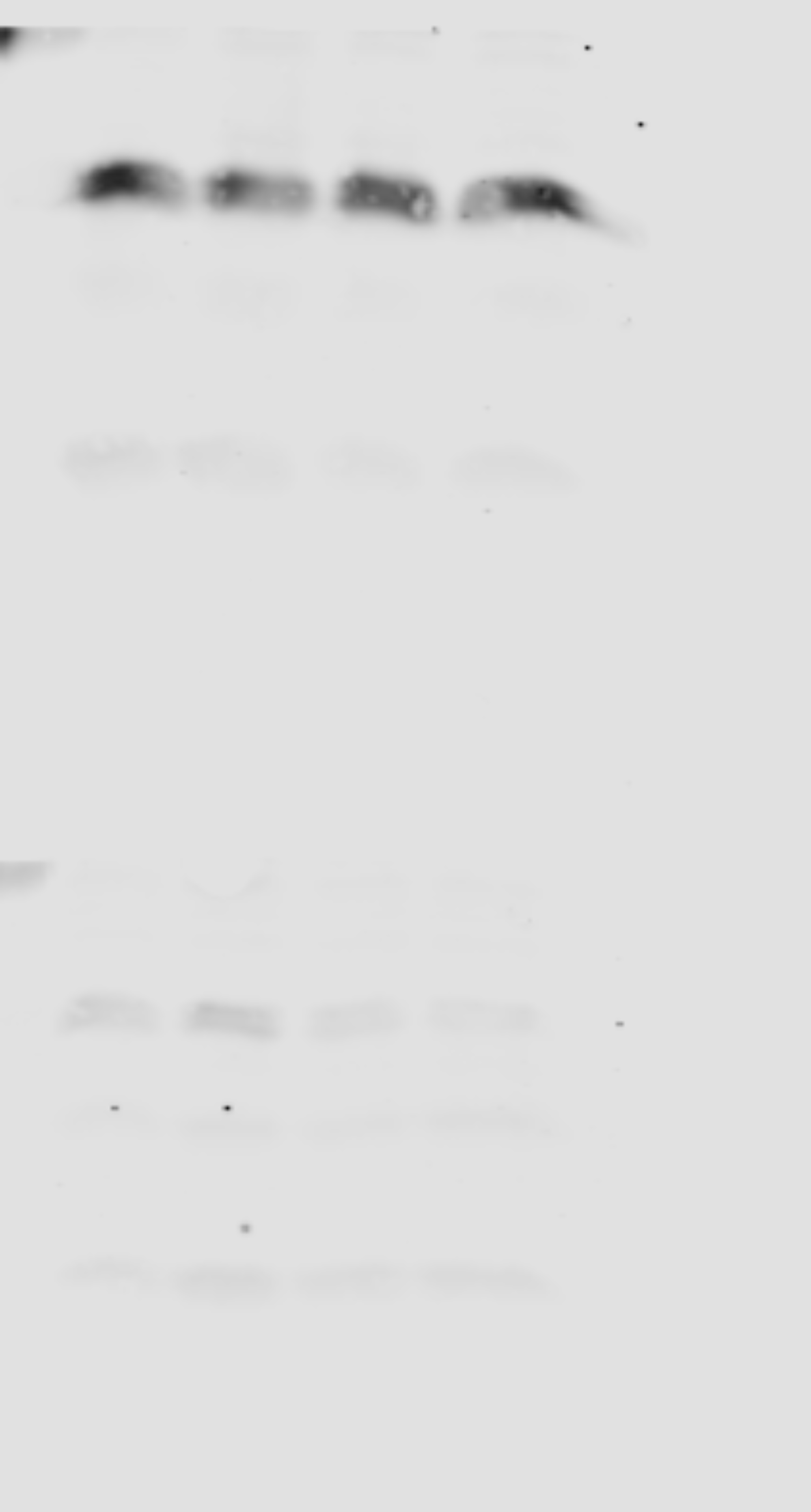

Supplement: Supplementary file 13 — Figure EV3 Source Data [file 44318_2026_803_MOESM13_ESM.zip › Fig EV3/EV3I/pMLC2 2D vs low adh_low exposure.tif]

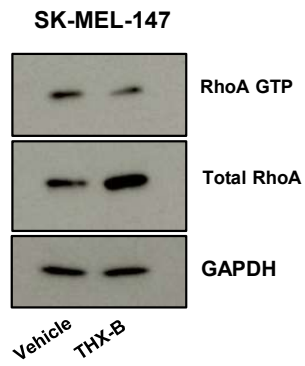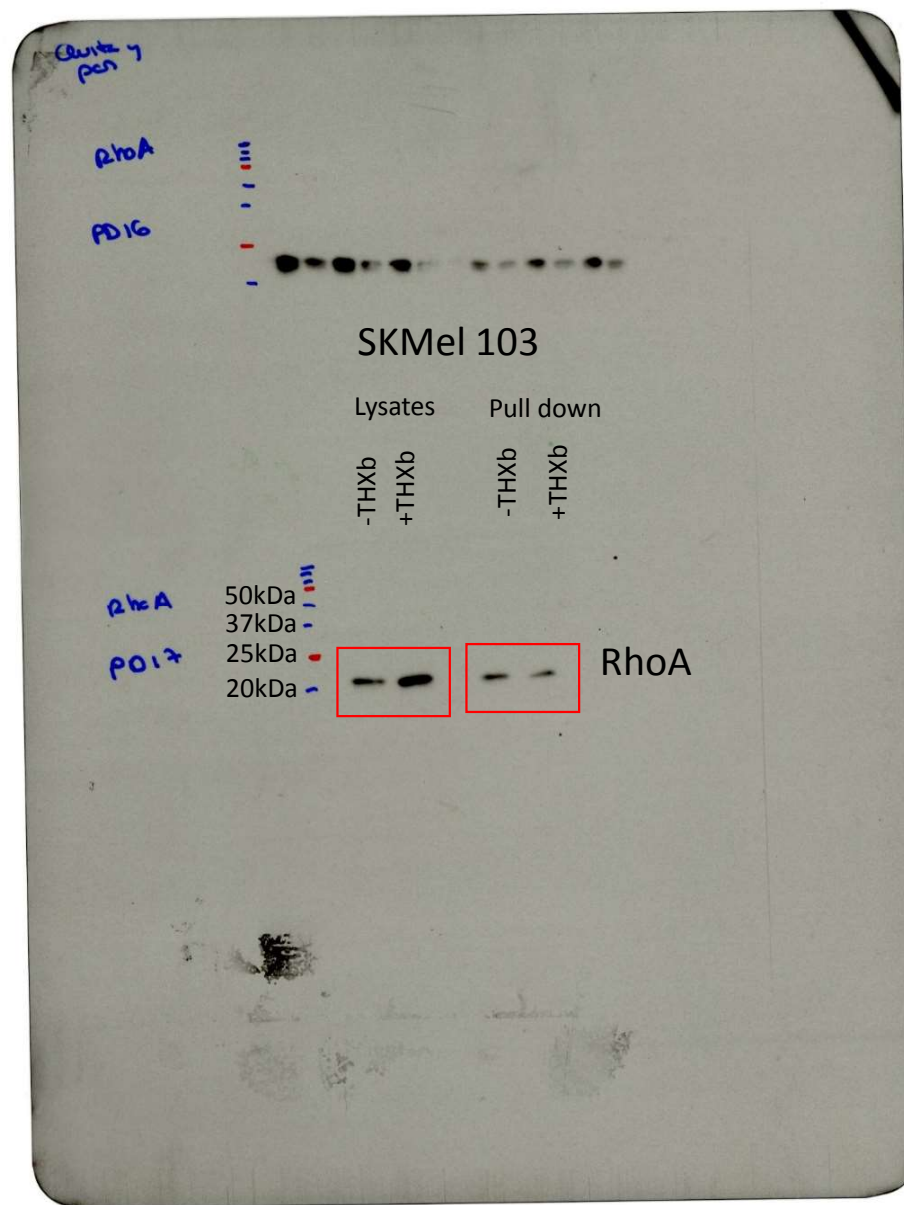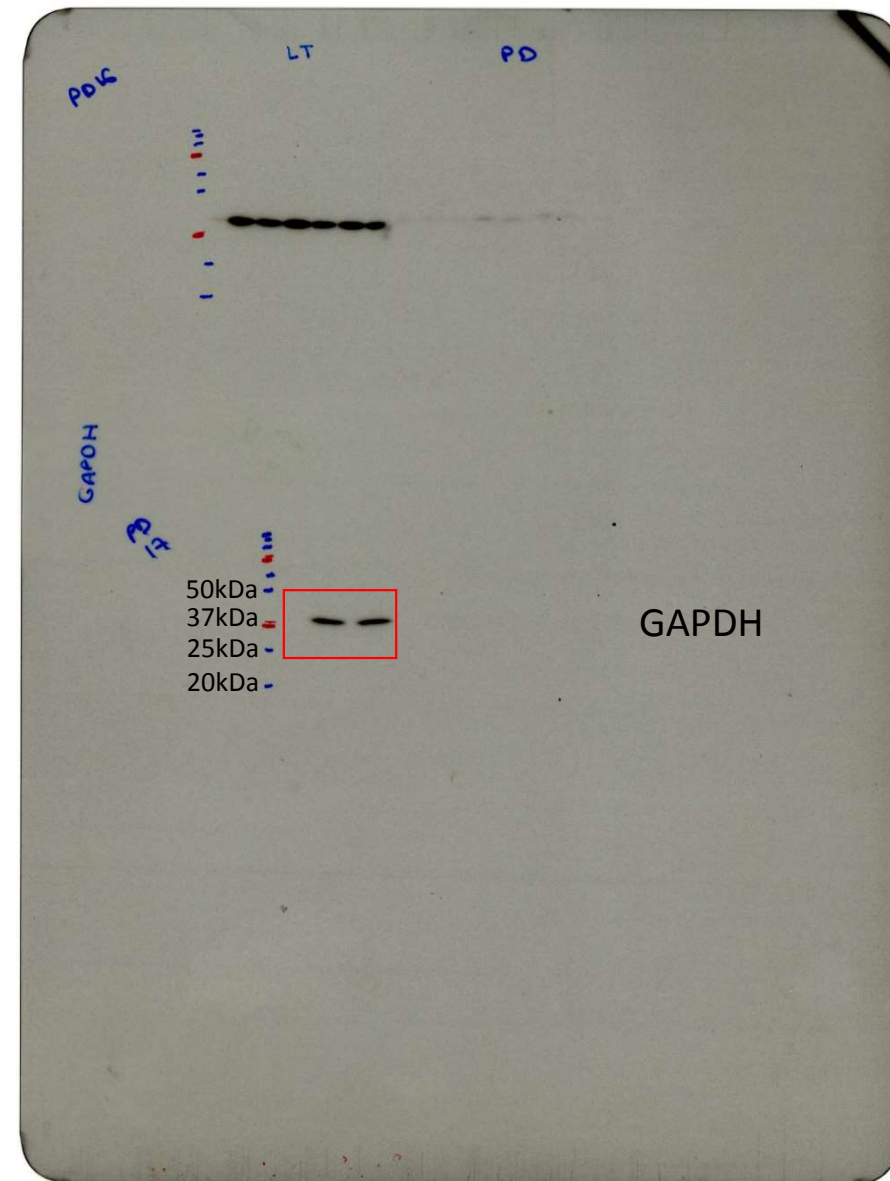

Supplement: Supplementary file 13 — Figure EV3 Source Data [file 44318_2026_803_MOESM13_ESM.zip › Fig EV3/EV3O/EV3O-Readme.pdf]

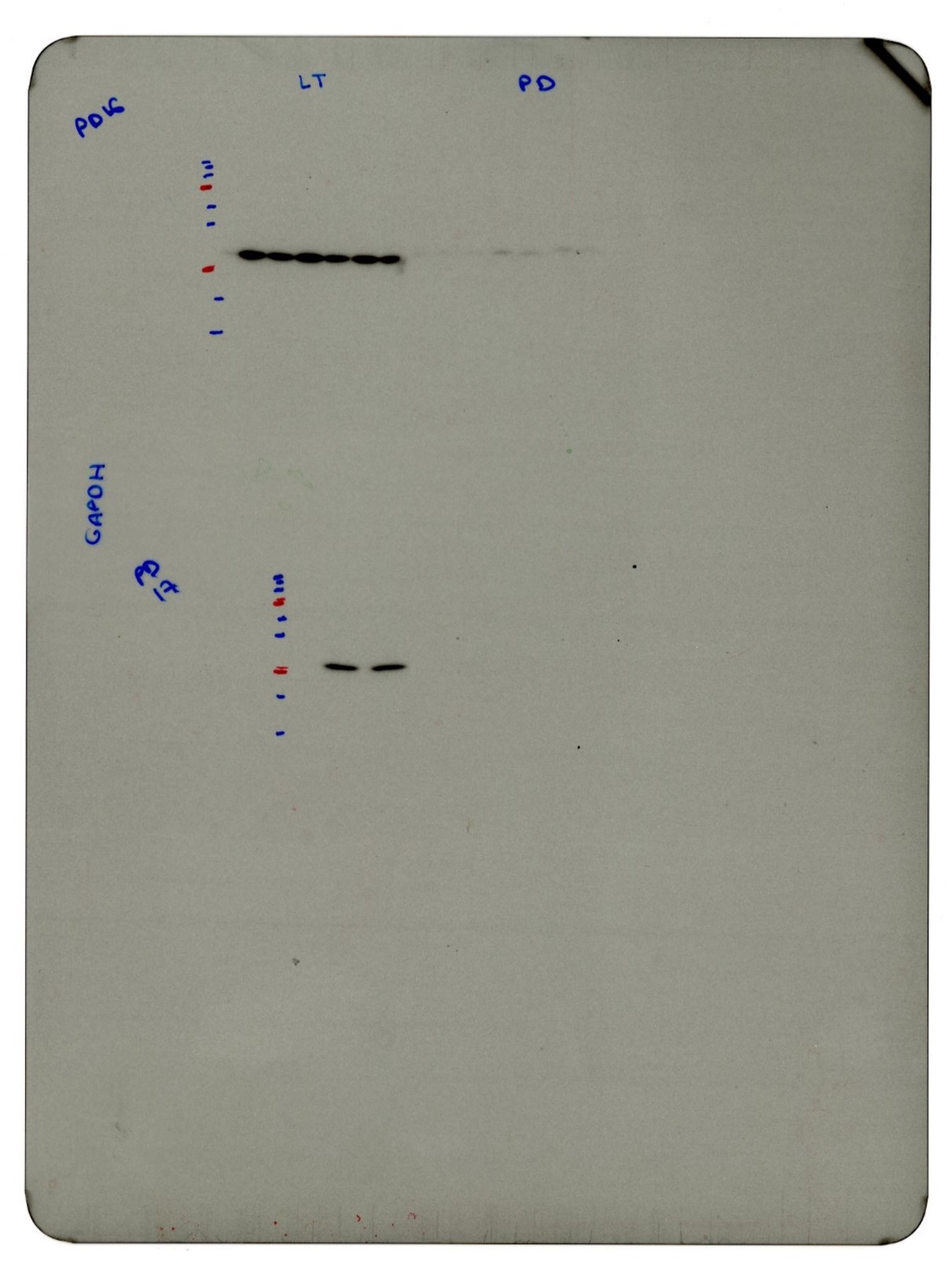

Supplement: Supplementary file 13 — Figure EV3 Source Data [file 44318_2026_803_MOESM13_ESM.zip › Fig EV3/EV3O/GAPDH.jpg]

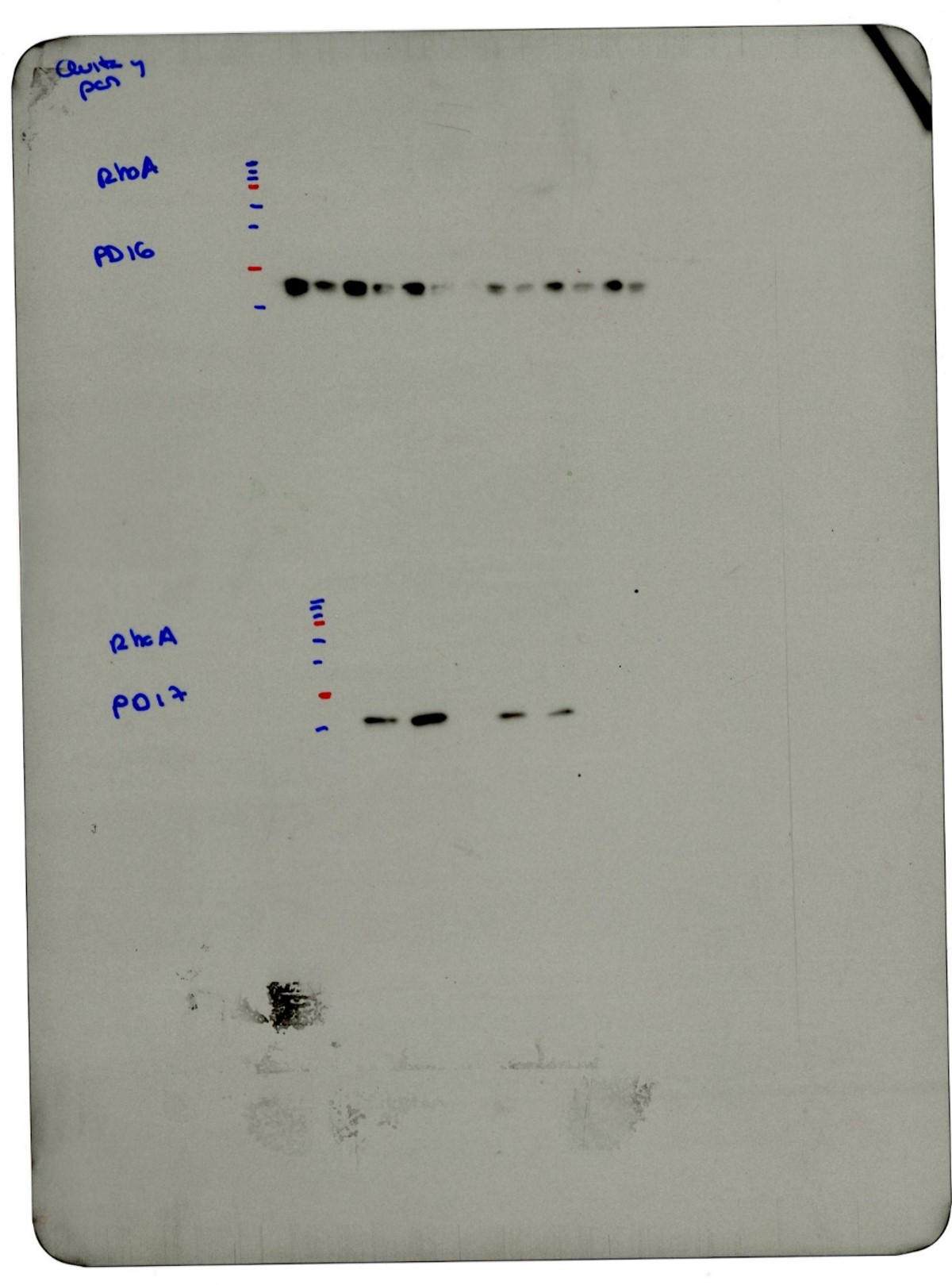

Supplement: Supplementary file 13 — Figure EV3 Source Data [file 44318_2026_803_MOESM13_ESM.zip › Fig EV3/EV3O/RhoA.jpg]

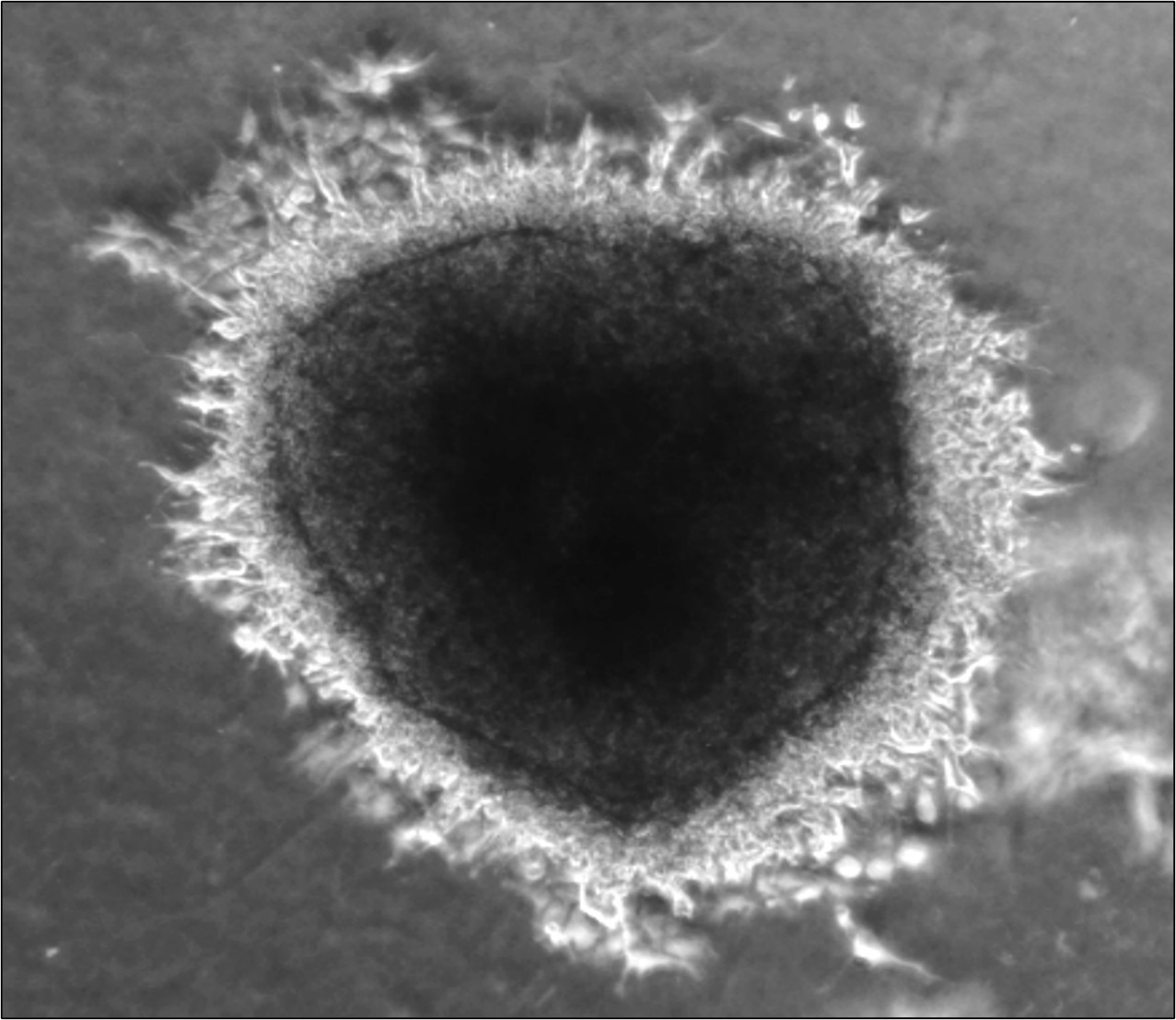

Supplement: Supplementary file 14 — Figure EV4 Source Data [file 44318_2026_803_MOESM14_ESM.zip › Fig EV4/EV4A/cp-C1-ROCKi.tif]

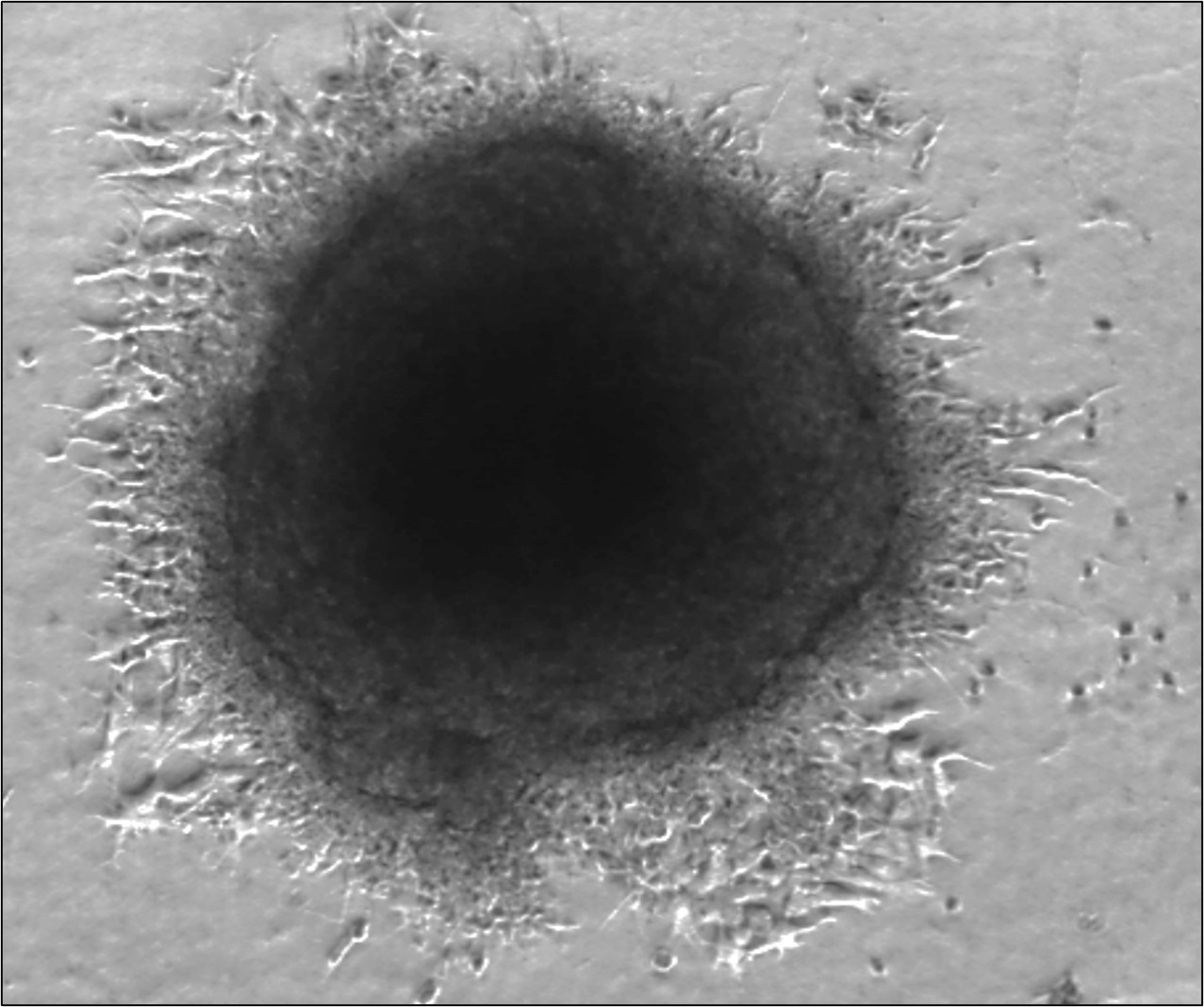

Supplement: Supplementary file 14 — Figure EV4 Source Data [file 44318_2026_803_MOESM14_ESM.zip › Fig EV4/EV4A/cp-C2-ROCKi.tif]

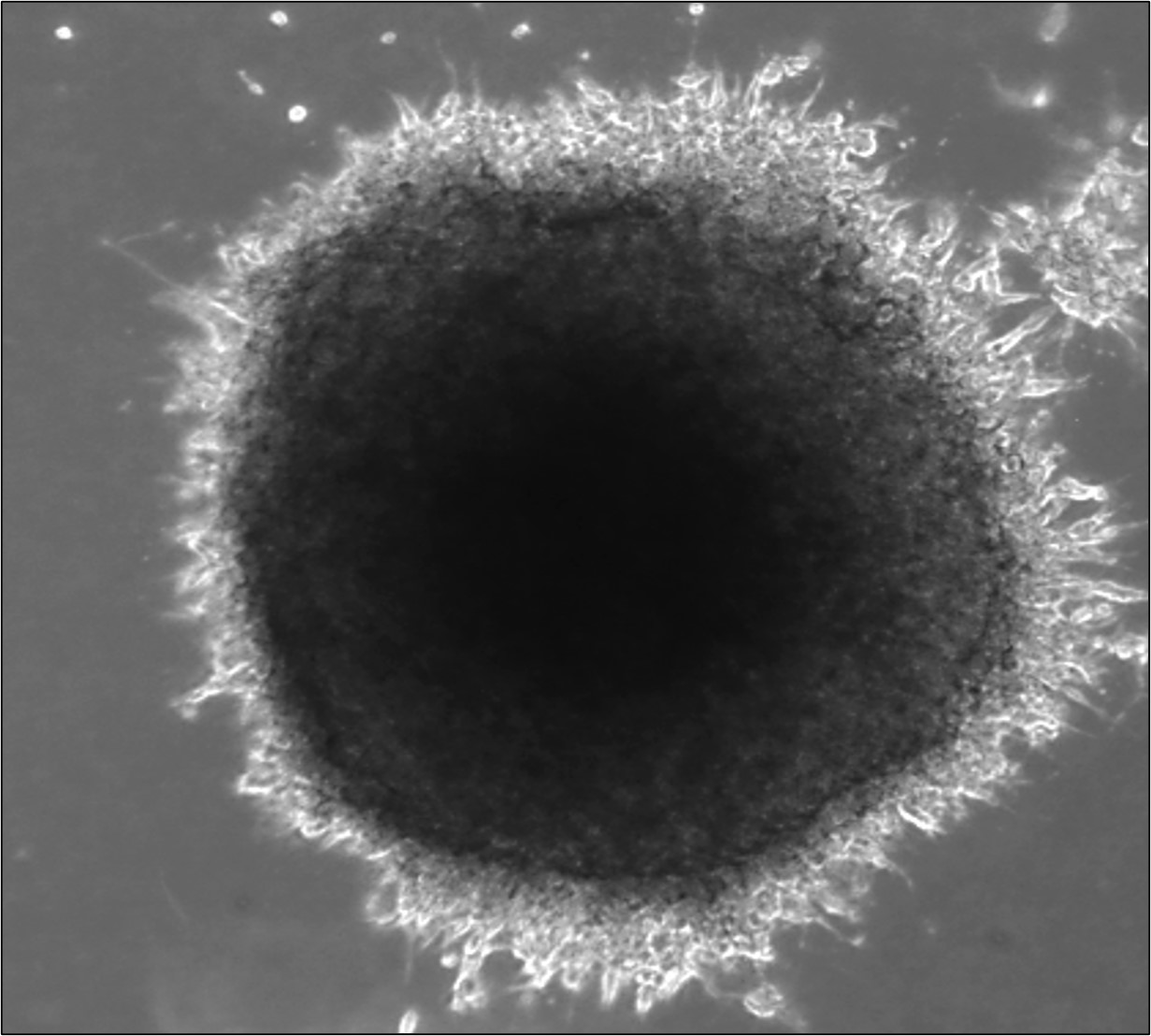

Supplement: Supplementary file 14 — Figure EV4 Source Data [file 44318_2026_803_MOESM14_ESM.zip › Fig EV4/EV4A/cp-NGFR1-ROCKi.tif]

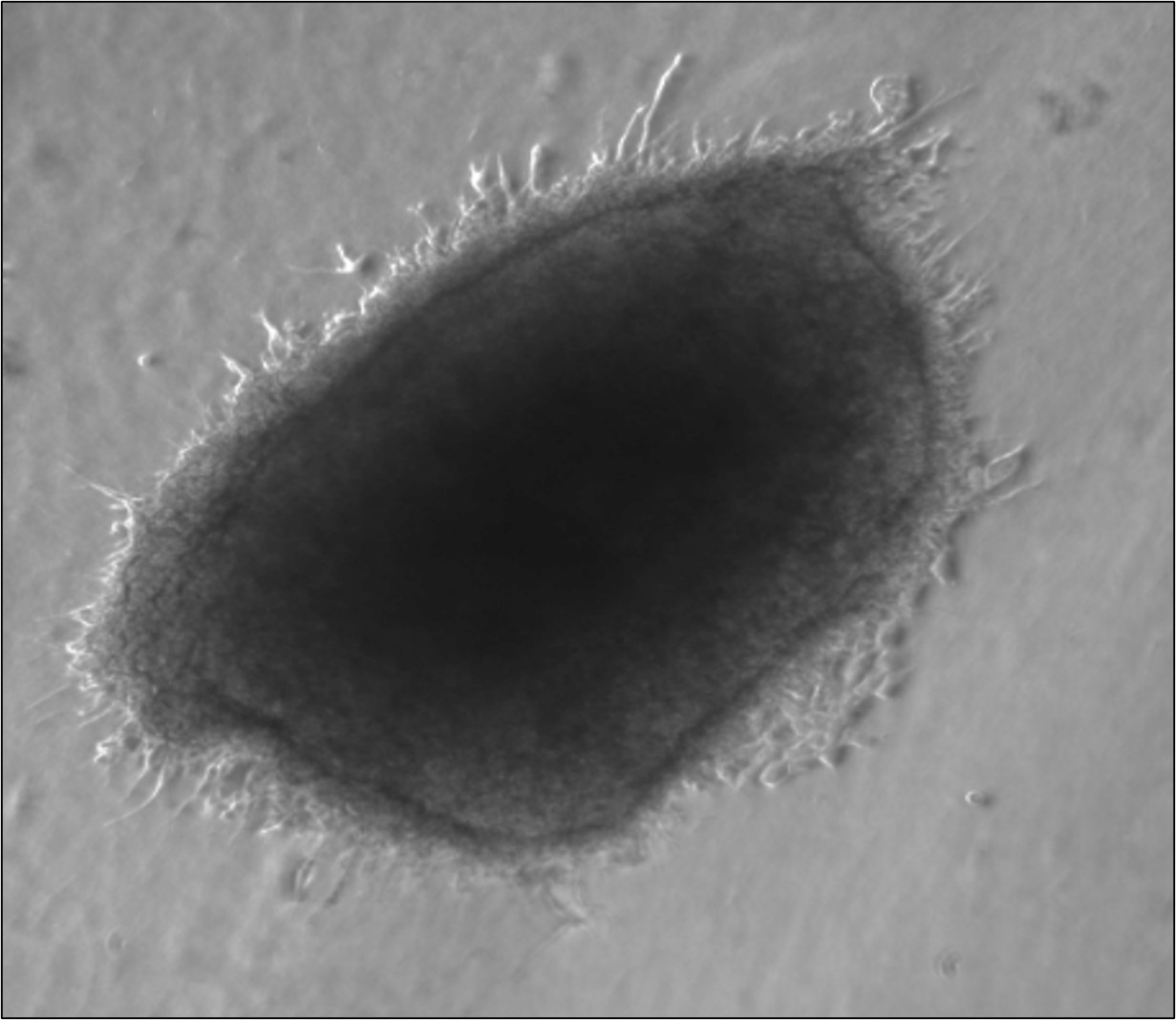

Supplement: Supplementary file 14 — Figure EV4 Source Data [file 44318_2026_803_MOESM14_ESM.zip › Fig EV4/EV4A/cp-NGFR2-ROCKi.tif]

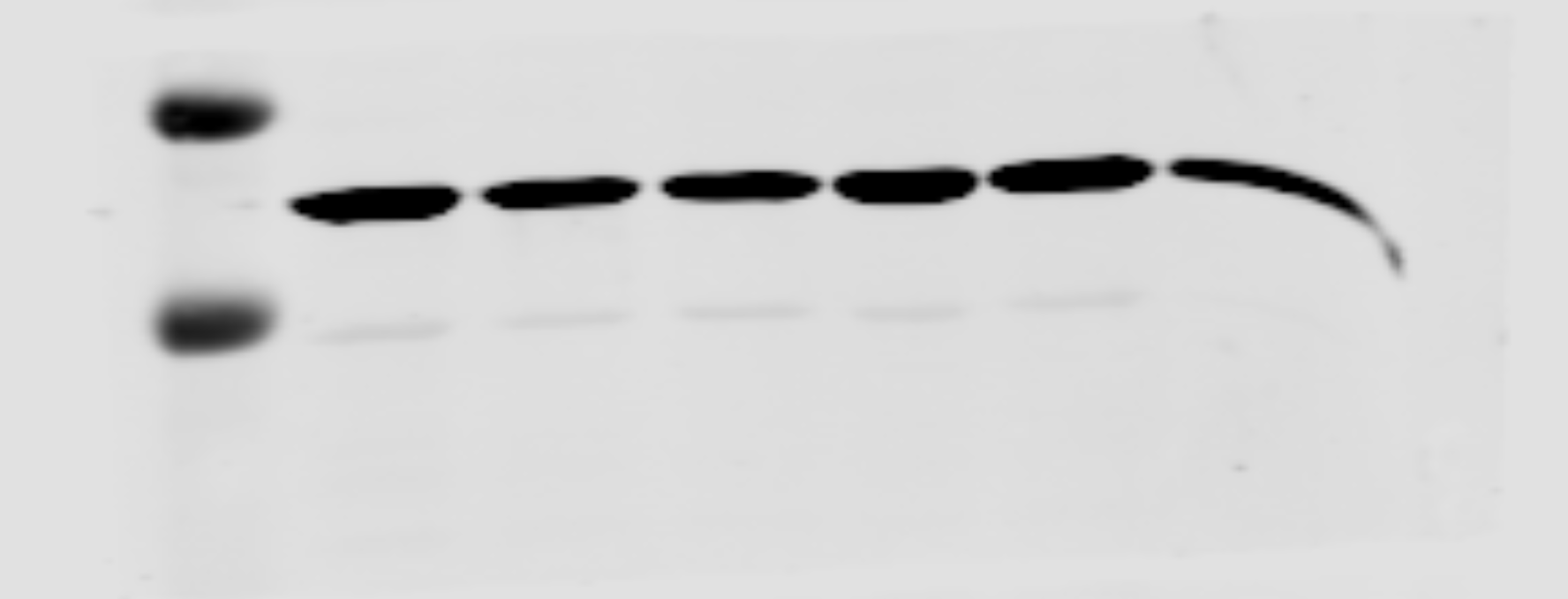

Supplement: Supplementary file 14 — Figure EV4 Source Data [file 44318_2026_803_MOESM14_ESM.zip › Fig EV4/EV4C/ACTIN.tif]

c)

A375P A375M

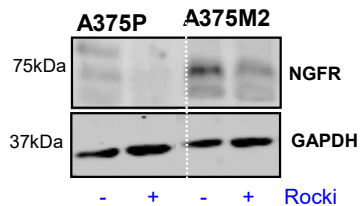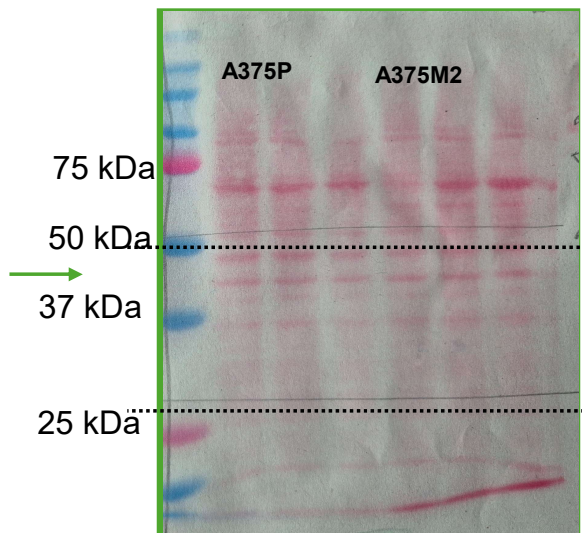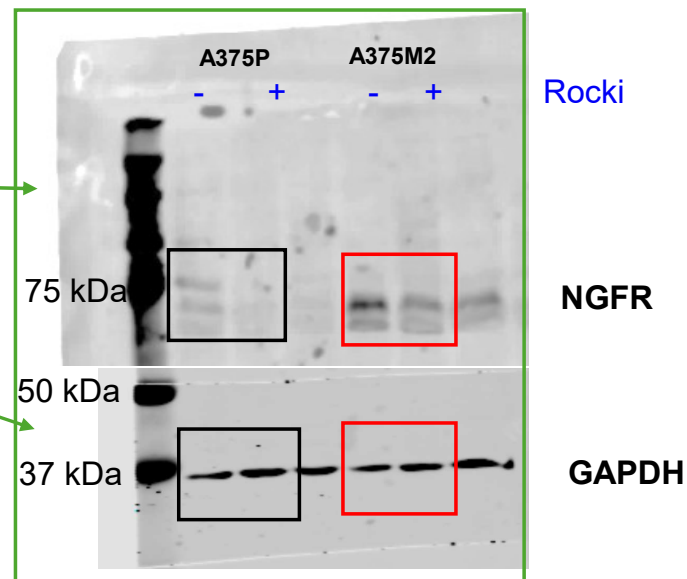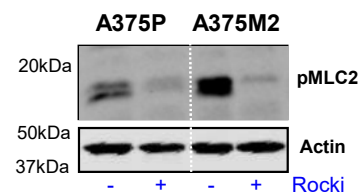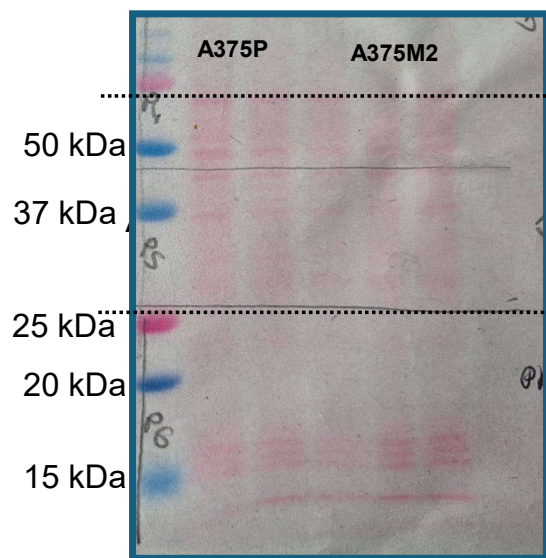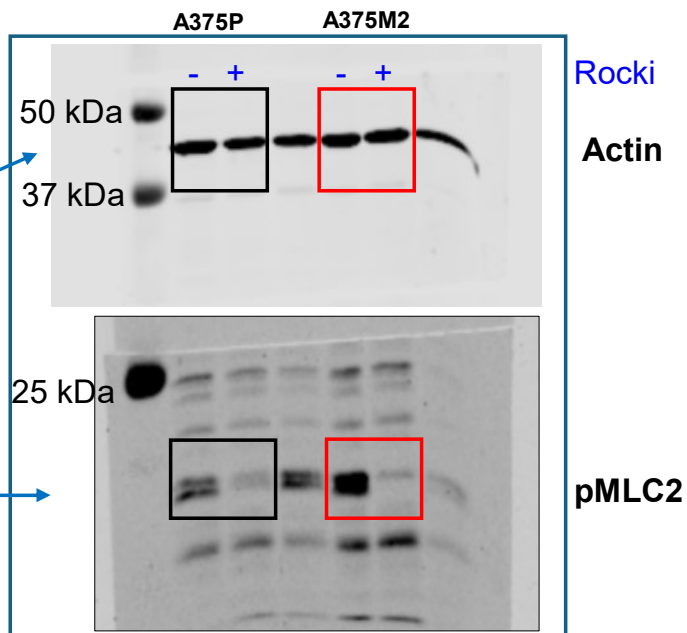

Supplement: Supplementary file 14 — Figure EV4 Source Data [file 44318_2026_803_MOESM14_ESM.zip › Fig EV4/EV4C/EV4C-Readme.pdf]

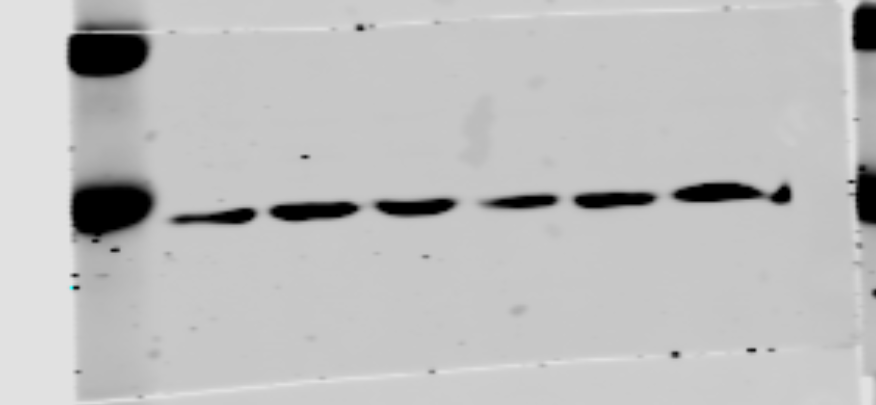

Supplement: Supplementary file 14 — Figure EV4 Source Data [file 44318_2026_803_MOESM14_ESM.zip › Fig EV4/EV4C/GAPDH.tif]

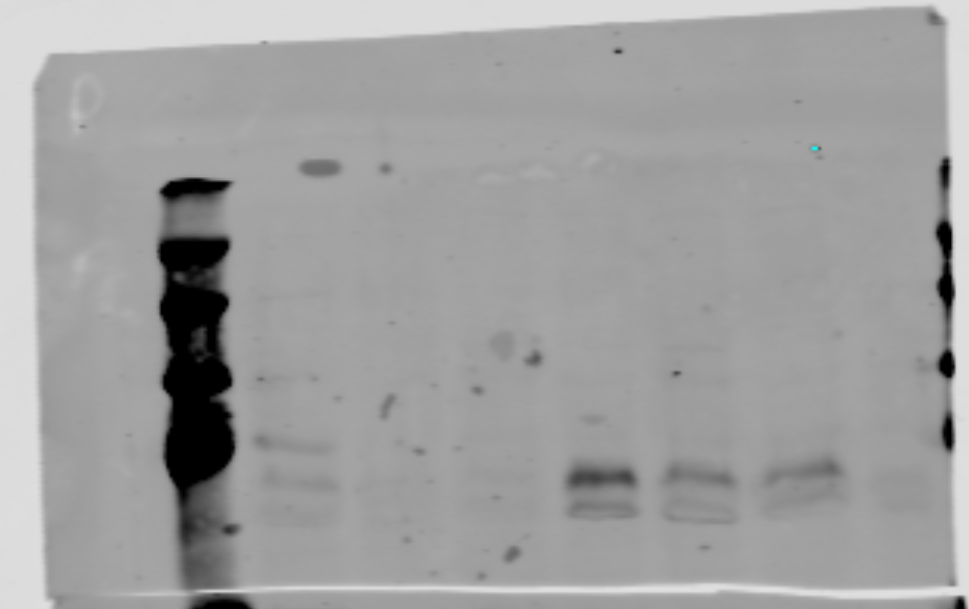

Supplement: Supplementary file 14 — Figure EV4 Source Data [file 44318_2026_803_MOESM14_ESM.zip › Fig EV4/EV4C/NGFR.tif]

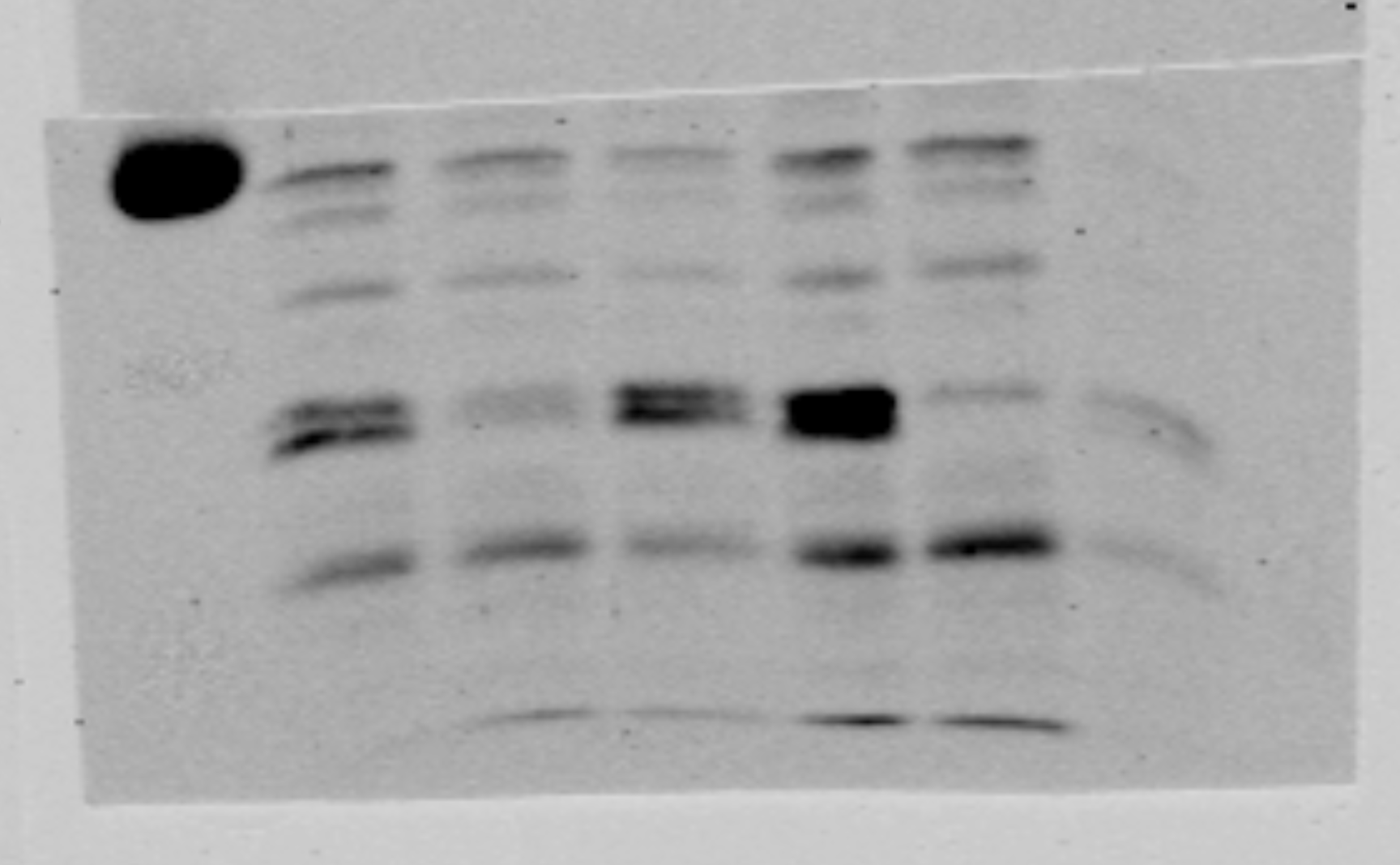

Supplement: Supplementary file 14 — Figure EV4 Source Data [file 44318_2026_803_MOESM14_ESM.zip › Fig EV4/EV4C/pMLC2.tif]

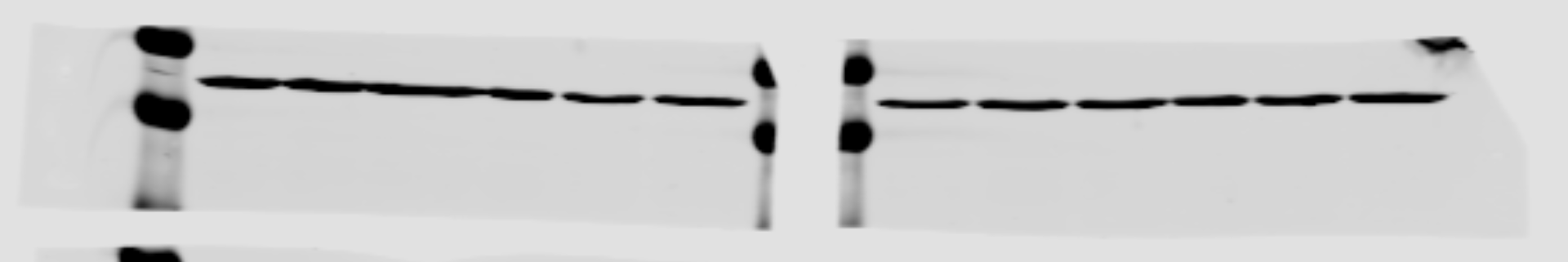

Supplement: Supplementary file 14 — Figure EV4 Source Data [file 44318_2026_803_MOESM14_ESM.zip › Fig EV4/EV4F/ACTIN.tif]

f) SK-MEL-147 - ROCKi

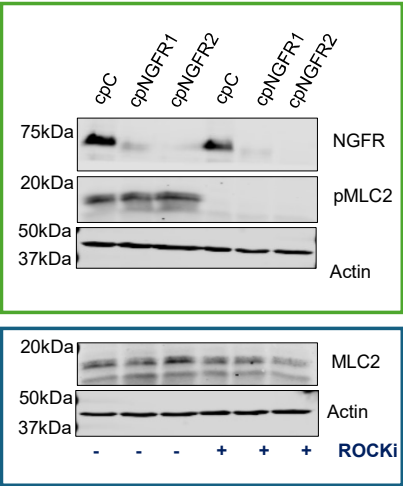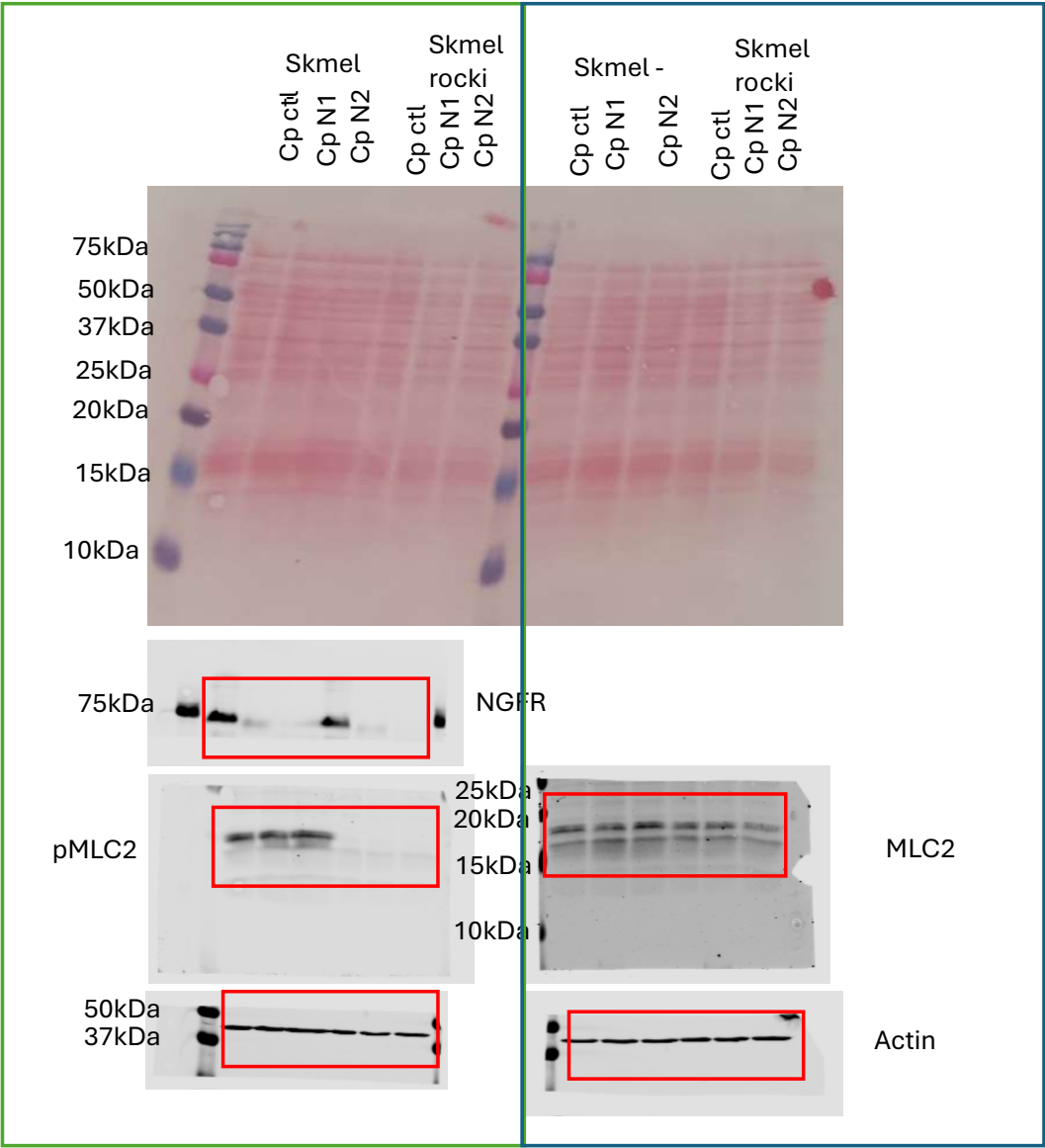

Supplement: Supplementary file 14 — Figure EV4 Source Data [file 44318_2026_803_MOESM14_ESM.zip › Fig EV4/EV4F/EV4F-Readme.pdf]

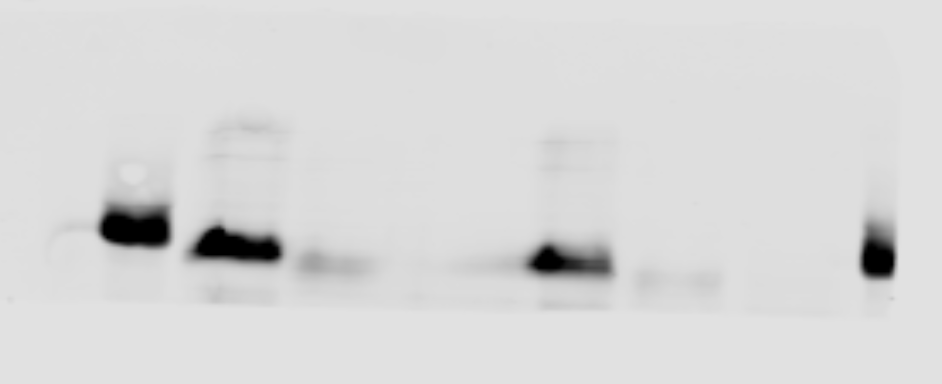

Supplement: Supplementary file 14 — Figure EV4 Source Data [file 44318_2026_803_MOESM14_ESM.zip › Fig EV4/EV4F/NGFR.tif]

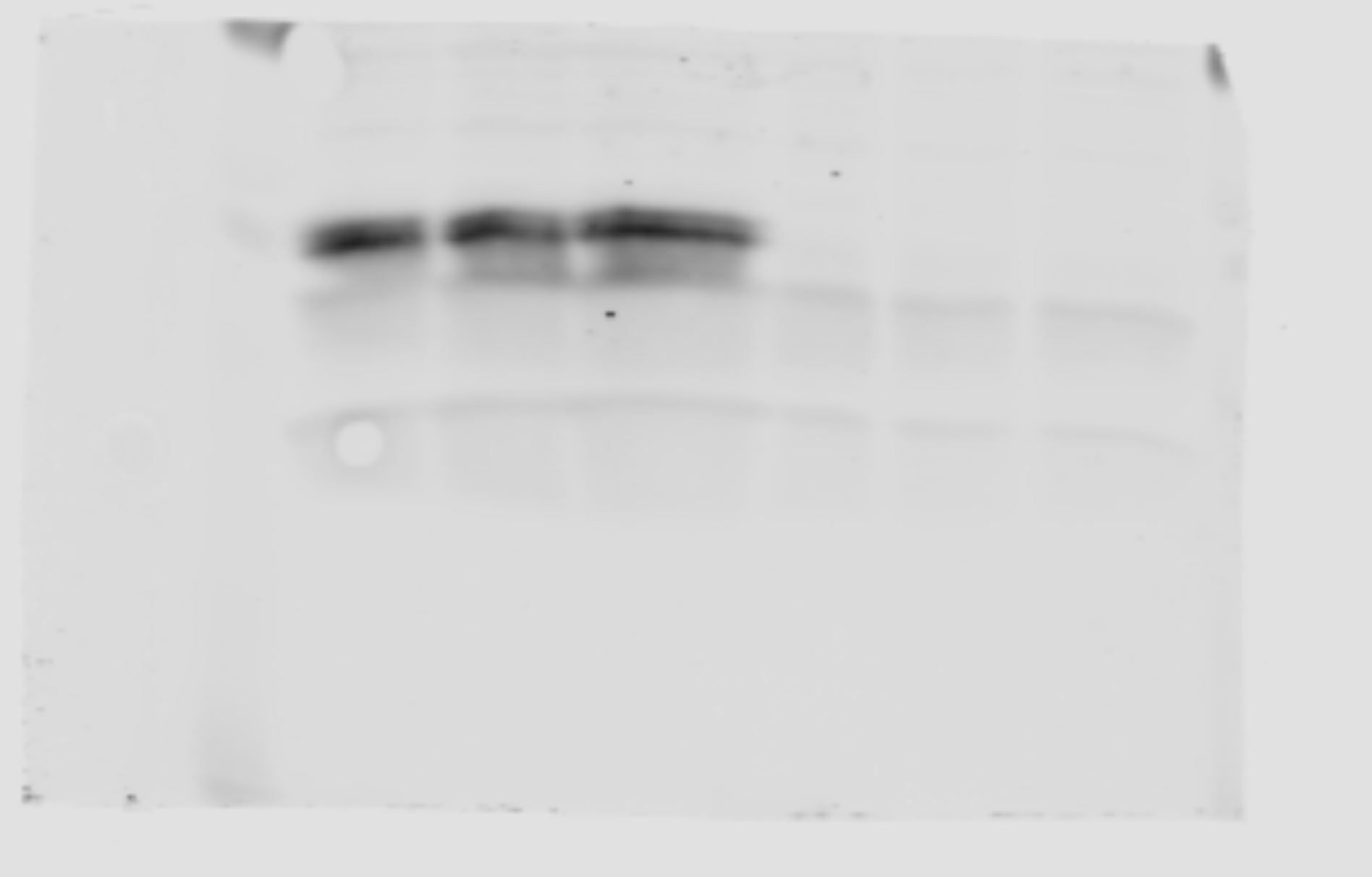

Supplement: Supplementary file 14 — Figure EV4 Source Data [file 44318_2026_803_MOESM14_ESM.zip › Fig EV4/EV4F/pMCL2.tif]

h)

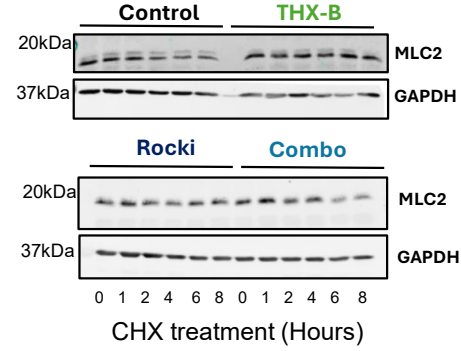

**CHX**

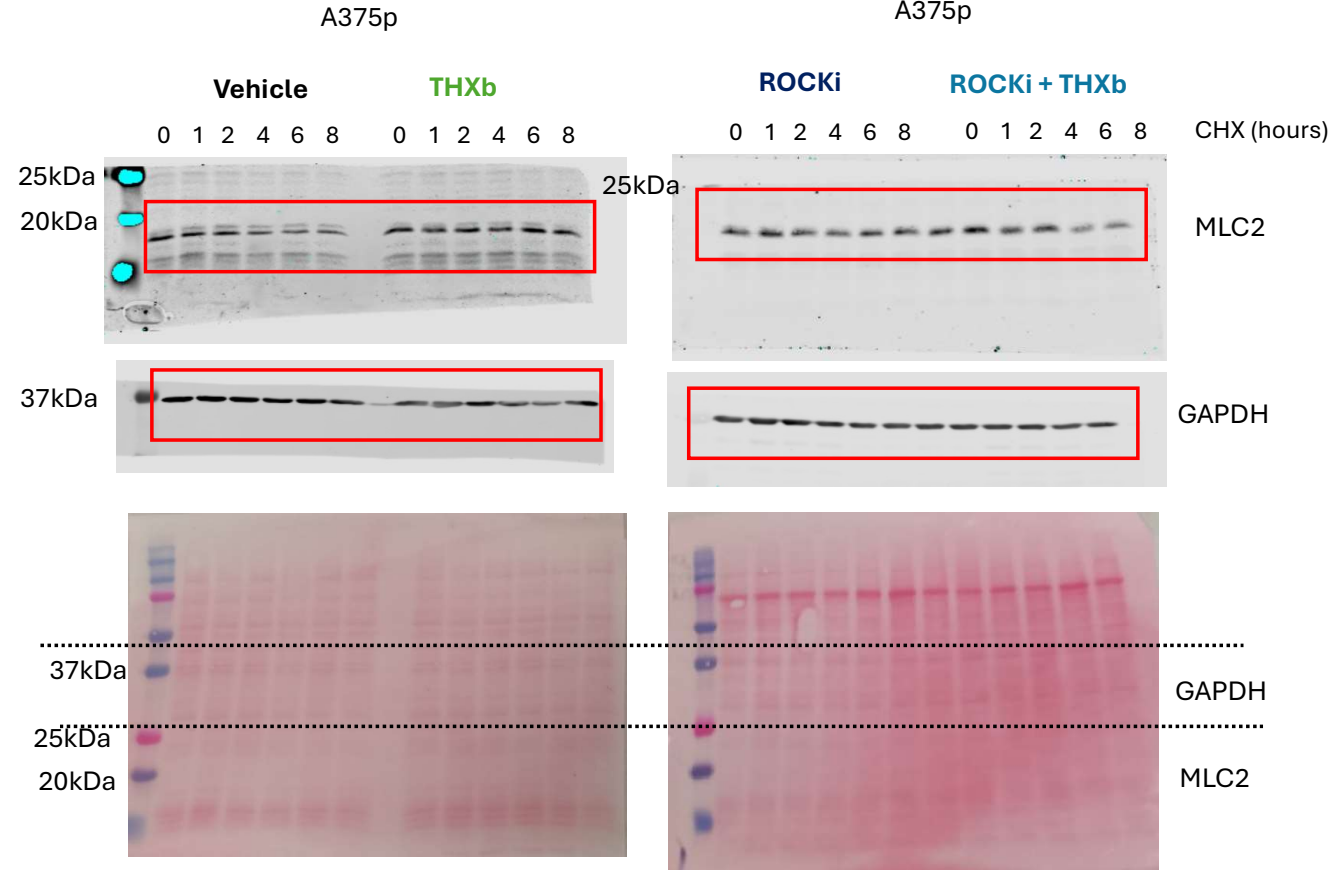

Supplement: Supplementary file 14 — Figure EV4 Source Data [file 44318_2026_803_MOESM14_ESM.zip › Fig EV4/EV4H/EV4H-Readme.pdf]

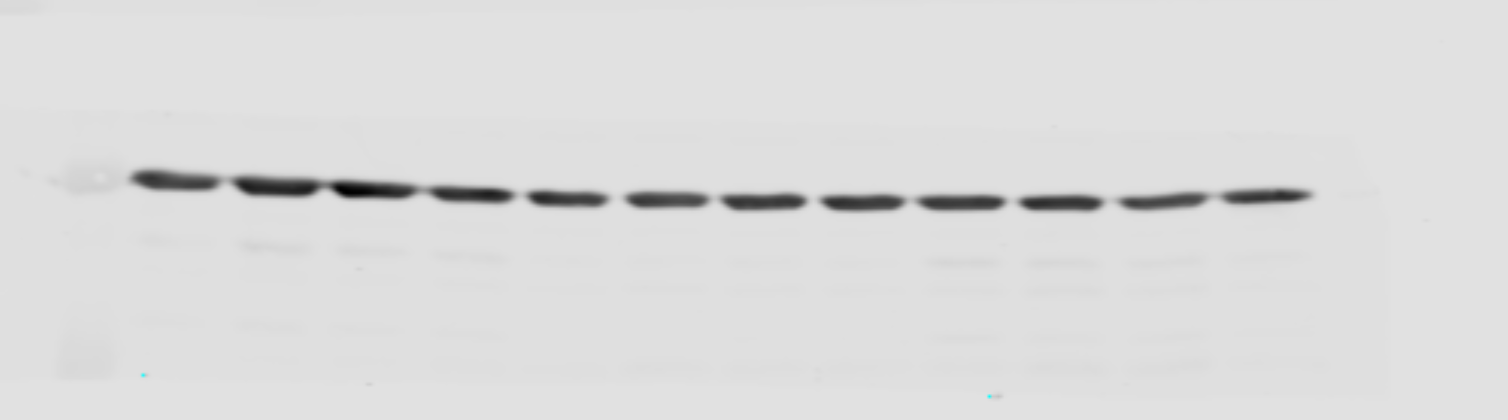

Supplement: Supplementary file 14 — Figure EV4 Source Data [file 44318_2026_803_MOESM14_ESM.zip › Fig EV4/EV4H/ROCKi-Combo-GAPDH.tif]

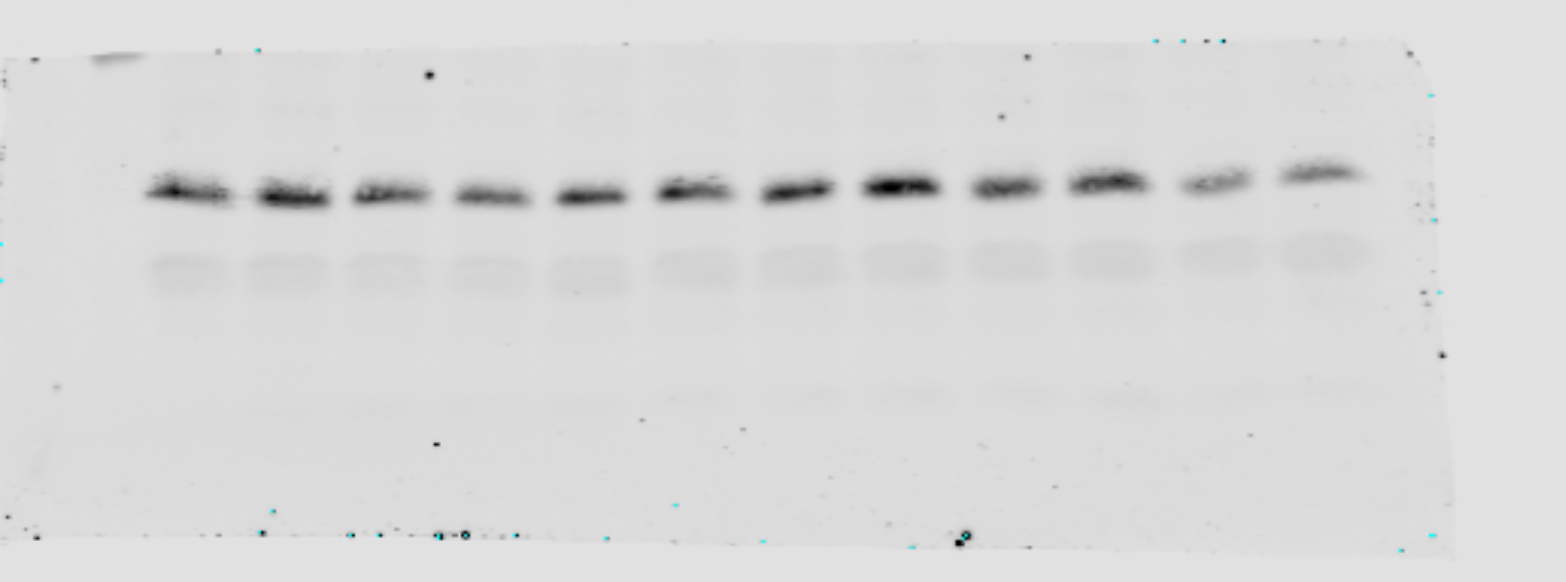

Supplement: Supplementary file 14 — Figure EV4 Source Data [file 44318_2026_803_MOESM14_ESM.zip › Fig EV4/EV4H/ROCKi-Combo-MLC2.tif]

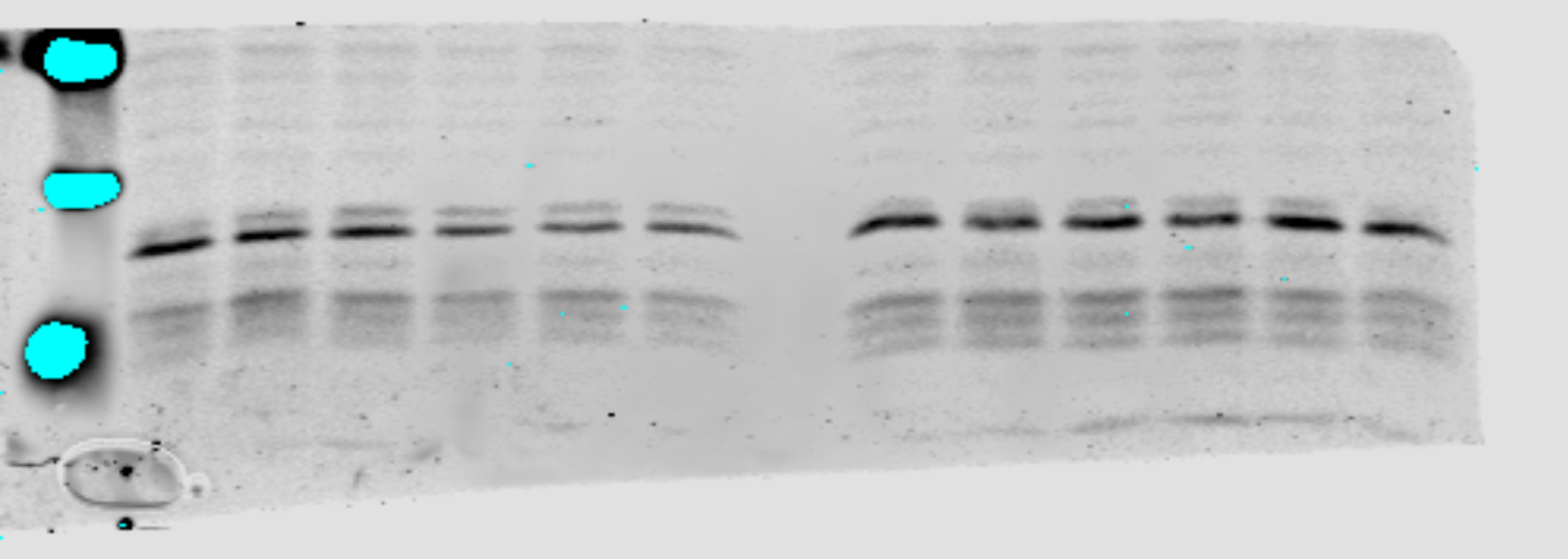

Supplement: Supplementary file 14 — Figure EV4 Source Data [file 44318_2026_803_MOESM14_ESM.zip › Fig EV4/EV4H/Vehicle-THX-B MLC2.tif]

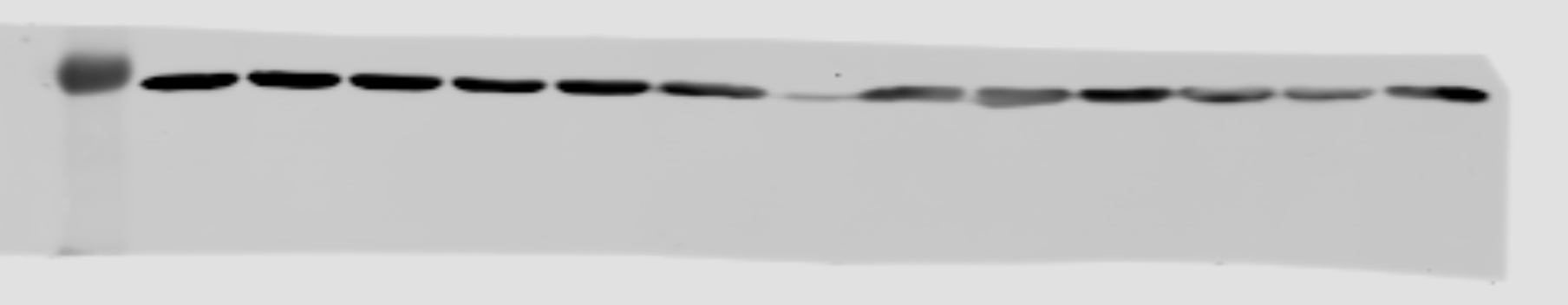

Supplement: Supplementary file 14 — Figure EV4 Source Data [file 44318_2026_803_MOESM14_ESM.zip › Fig EV4/EV4H/Vehicle-THX-B-GAPDH.tif]

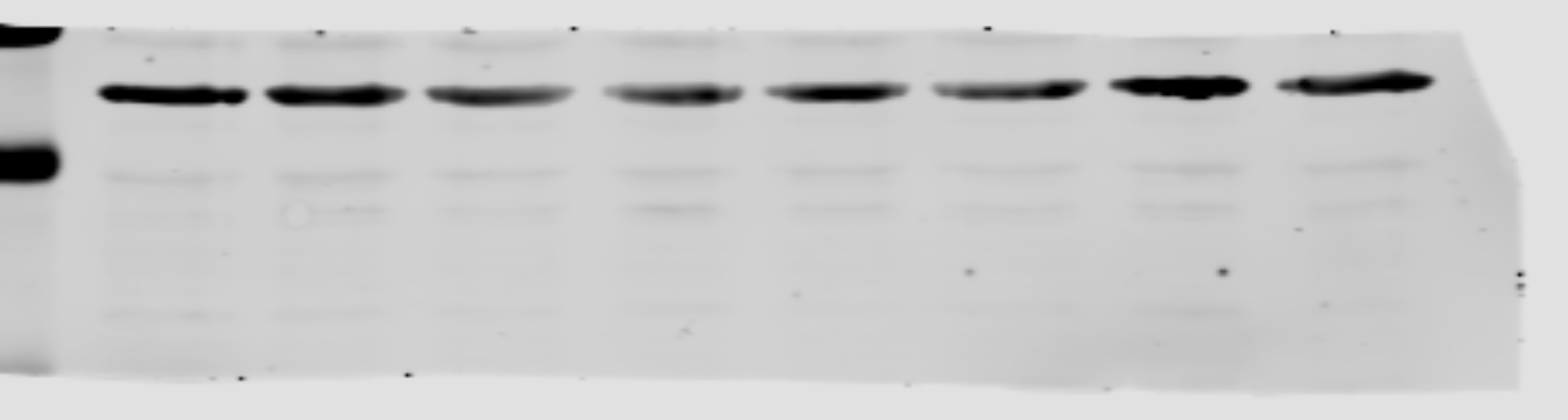

Supplement: Supplementary file 14 — Figure EV4 Source Data [file 44318_2026_803_MOESM14_ESM.zip › Fig EV4/EV4J/Actin.tif]

j)

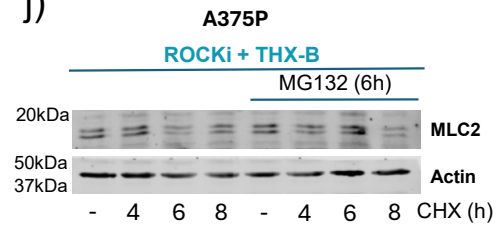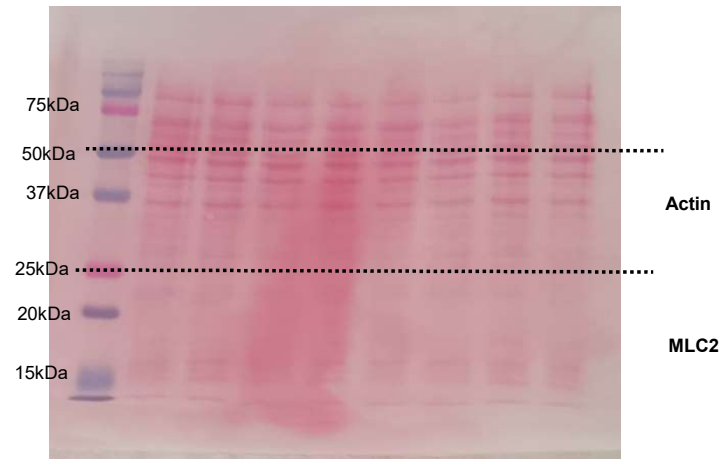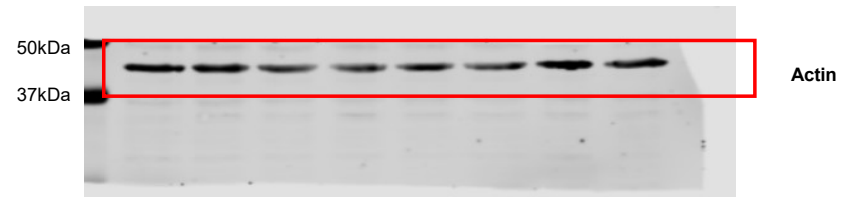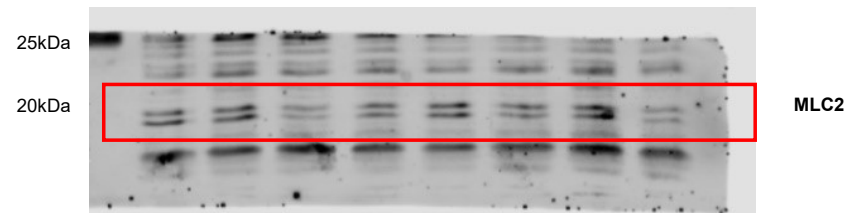

Supplement: Supplementary file 14 — Figure EV4 Source Data [file 44318_2026_803_MOESM14_ESM.zip › Fig EV4/EV4J/EV4J-Readme.pdf]

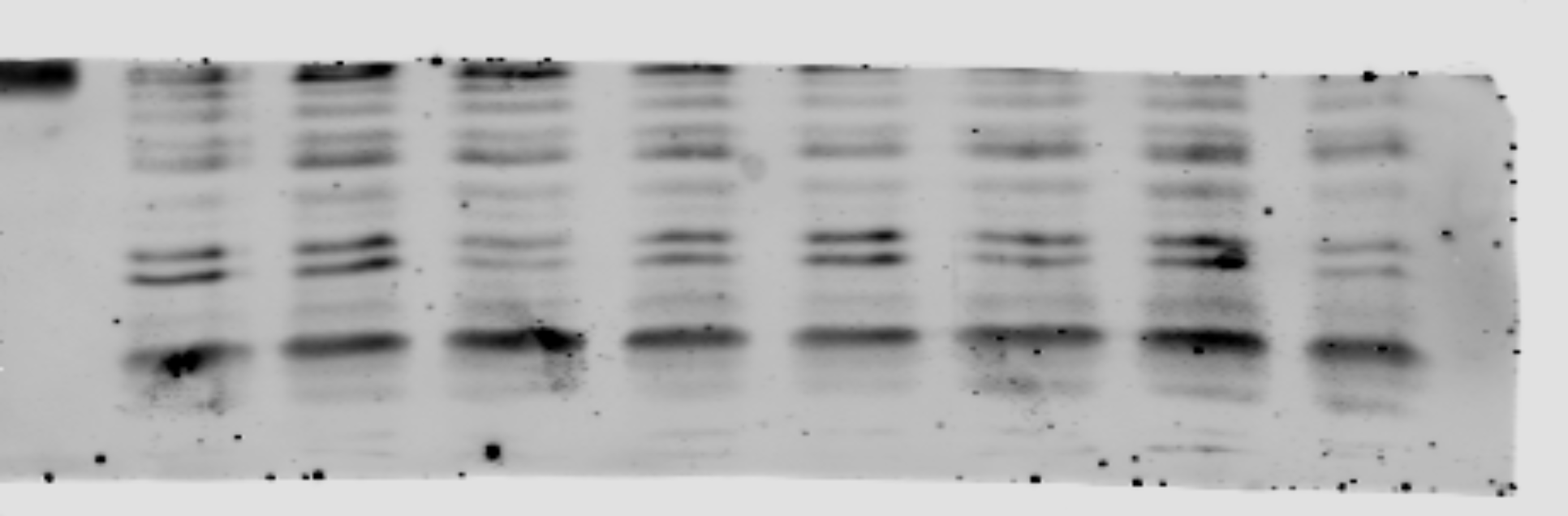

Supplement: Supplementary file 14 — Figure EV4 Source Data [file 44318_2026_803_MOESM14_ESM.zip › Fig EV4/EV4J/MLC2.tif]

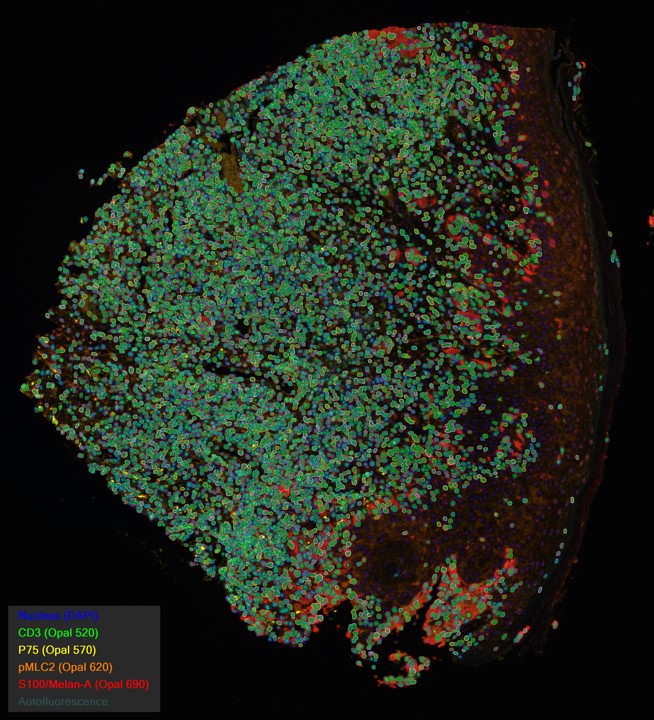

Supplement: Supplementary file 15 — Figure EV5 Source Data [file 44318_2026_803_MOESM15_ESM.zip › Fig EV5/EV5A/Cell segmentation.jpg]

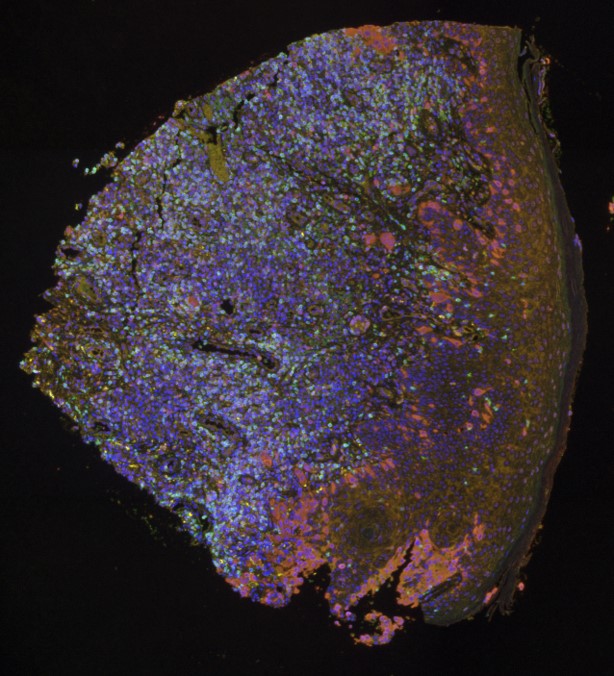

Supplement: Supplementary file 15 — Figure EV5 Source Data [file 44318_2026_803_MOESM15_ESM.zip › Fig EV5/EV5A/Raw image.jpg]

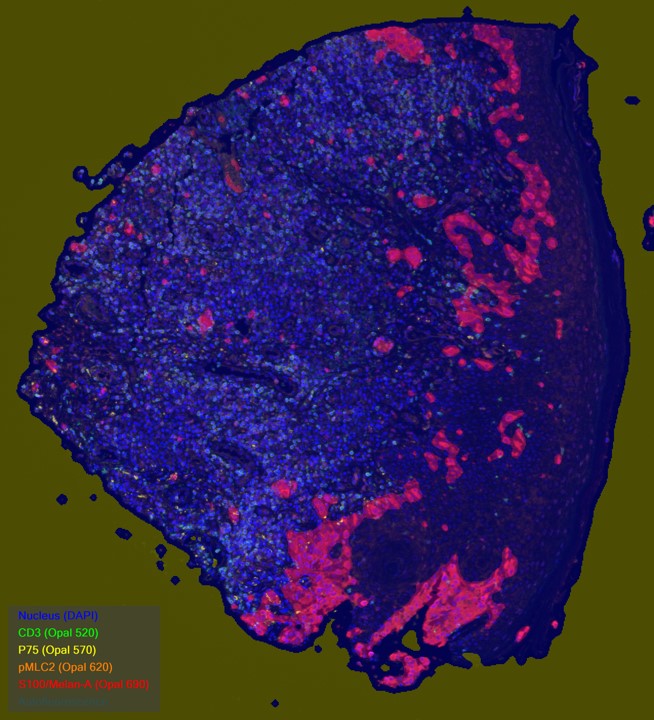

Supplement: Supplementary file 15 — Figure EV5 Source Data [file 44318_2026_803_MOESM15_ESM.zip › Fig EV5/EV5A/tissue segmentation.jpg]

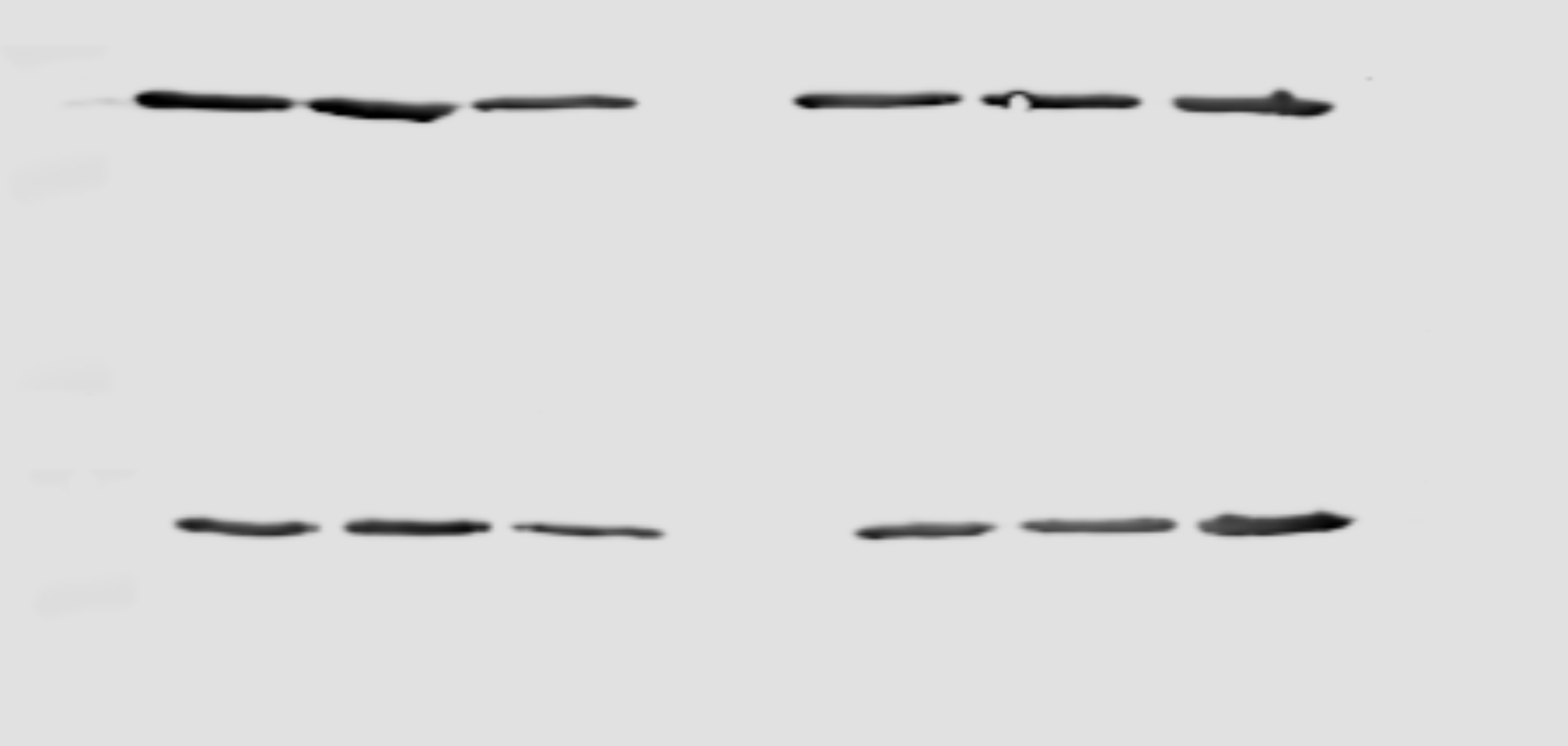

Supplement: Supplementary file 15 — Figure EV5 Source Data [file 44318_2026_803_MOESM15_ESM.zip › Fig EV5/EV5B/ACTIN.tif]

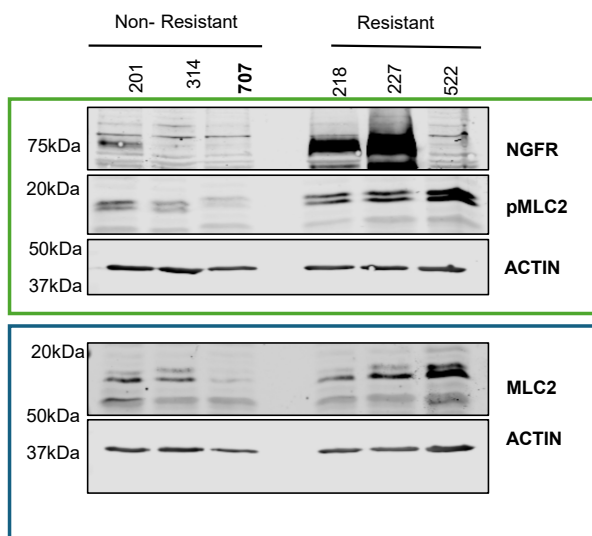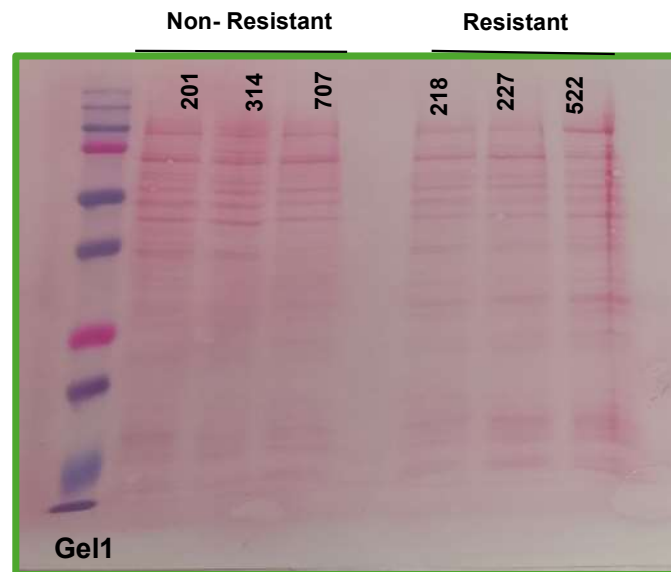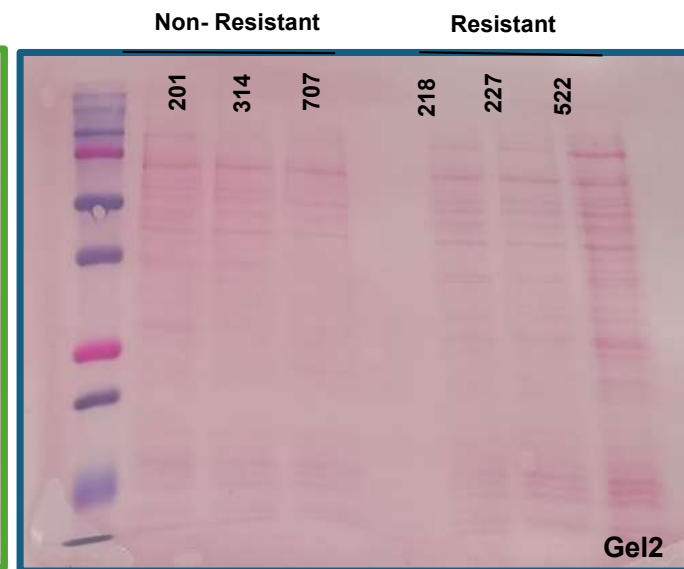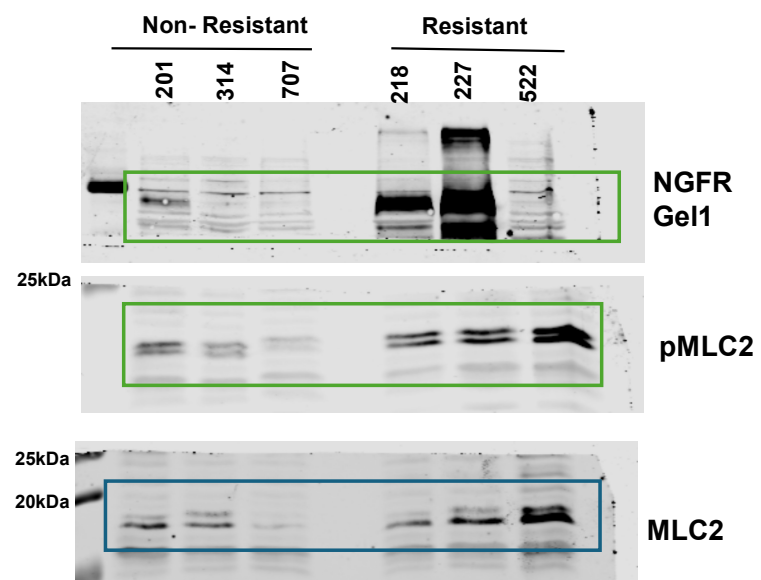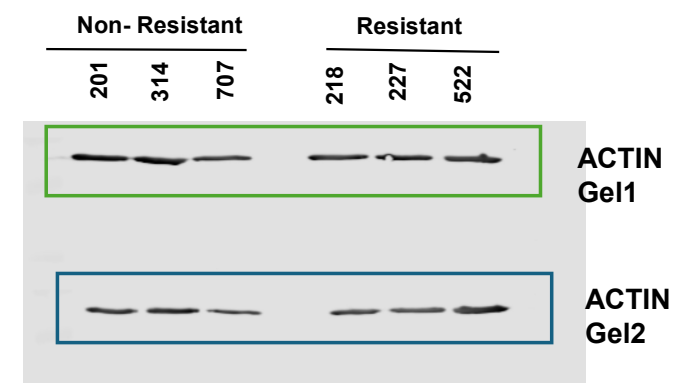

Supplement: Supplementary file 15 — Figure EV5 Source Data [file 44318_2026_803_MOESM15_ESM.zip › Fig EV5/EV5B/EV5A-Readme.pdf]

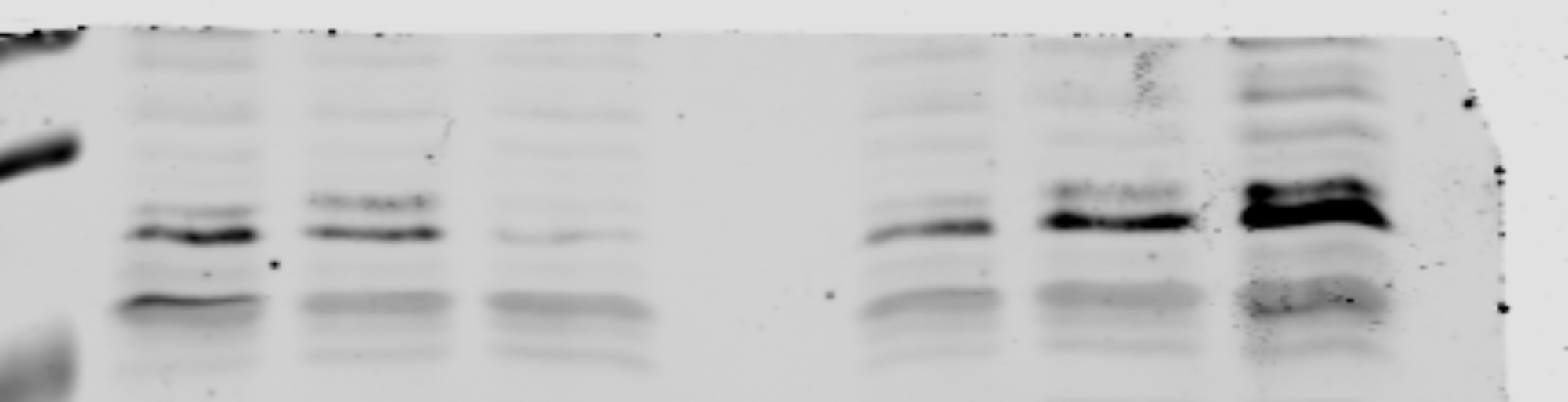

Supplement: Supplementary file 15 — Figure EV5 Source Data [file 44318_2026_803_MOESM15_ESM.zip › Fig EV5/EV5B/MLC2.tif]

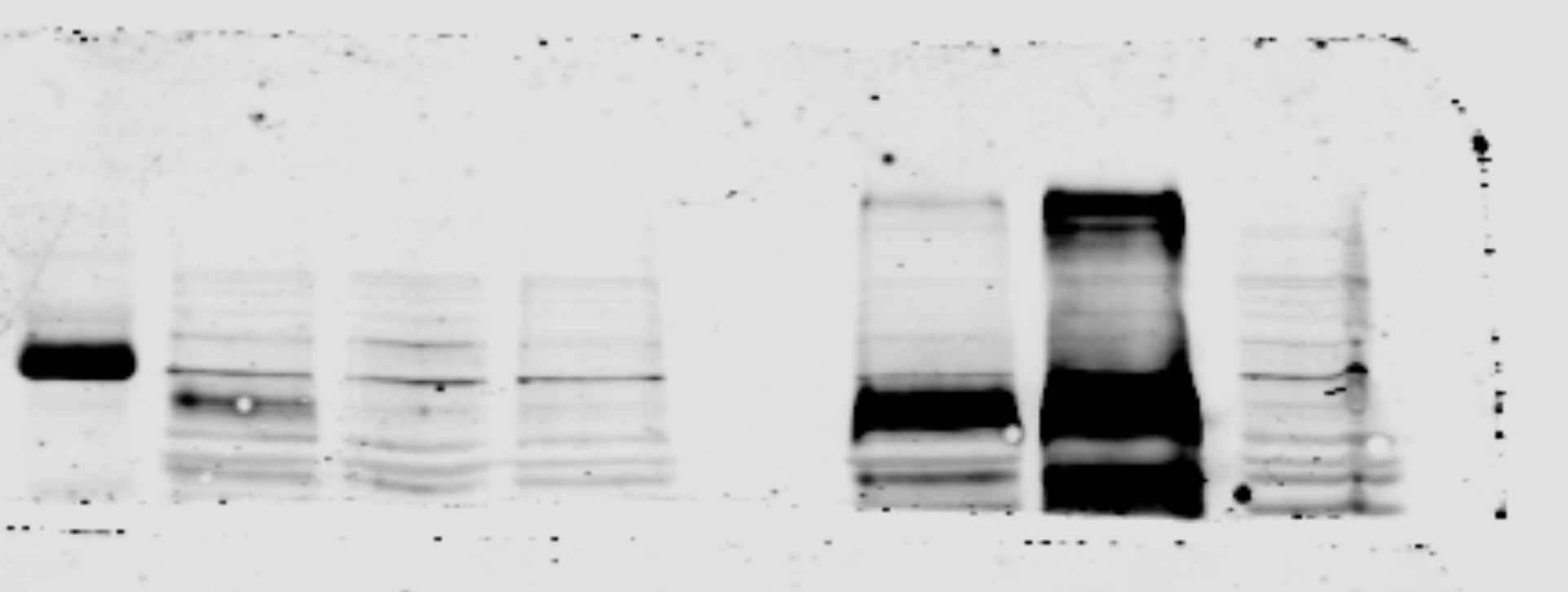

Supplement: Supplementary file 15 — Figure EV5 Source Data [file 44318_2026_803_MOESM15_ESM.zip › Fig EV5/EV5B/NGFR.tif]

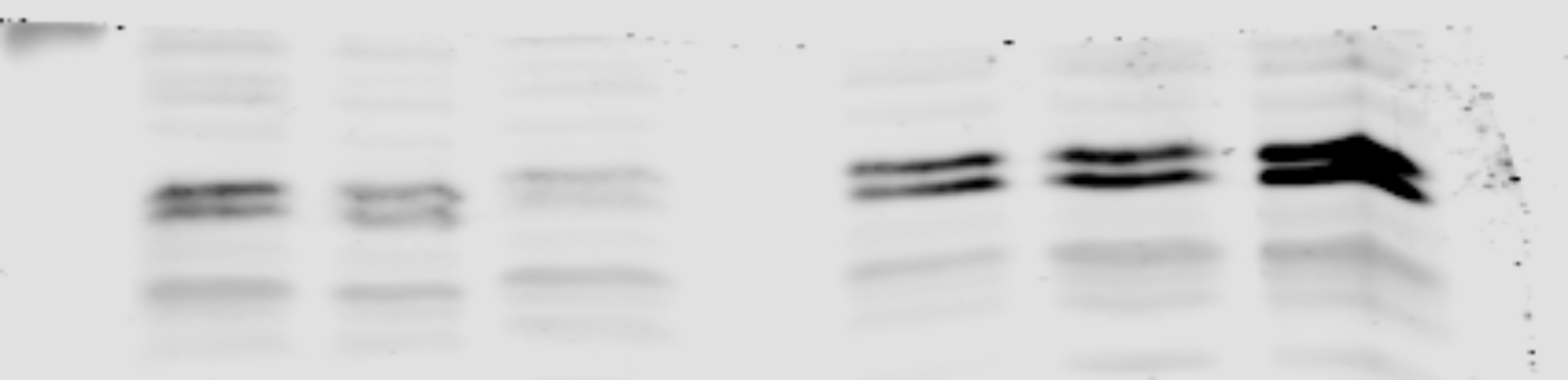

Supplement: Supplementary file 15 — Figure EV5 Source Data [file 44318_2026_803_MOESM15_ESM.zip › Fig EV5/EV5B/pMLC2.tif]
