# Supplementary material for: NGFR induces melanoma invasion and immunotherapy resistance through myosin light chain 2 modulation
Source: EMBO J. 2026 May 26;45(14):4988–5023. doi: 10.1038/s44318-026-00803-2 (PMC13373201; doi:10.1038/s44318-026-00803-2)
Supplement: Supplementary file 16 — Expanded View Figures [file 44318_2026_803_MOESM16_ESM.pdf]

Expanded View Figures

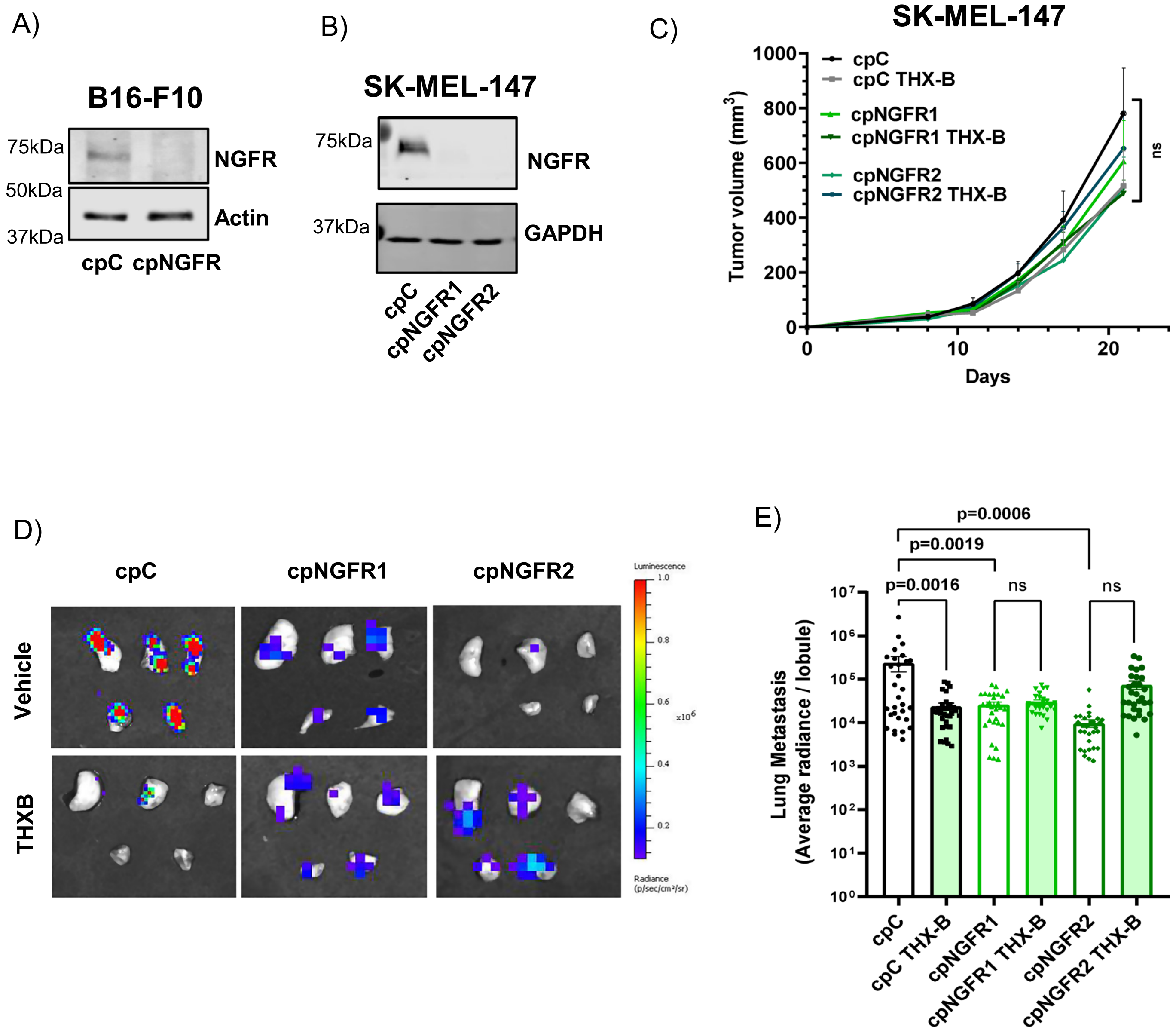

Figure EV1. THX-B decreases lung metastasis of human SK-MEL-147 melanoma tumors.

(**A**) Representative western blot showing NGFR expression in B16-F10 cpControl (cpC) and cpNGFR cells. (**B**) Representative western blot showing NGFR expression in SK-MEL-147, cpControl (cpC), and cpNGFR1/2 cells. (**C**) Tumor growth of SK-MEL-147 cpControl, cpNGFR1, or cpNGFR2 -GFPLuc tumors on Nude mice upon twice a week i.p. treatment with THX-B (5 mg/kg) or vehicle, starting at D7. Data were Mean + SEM of $n = 6$ mice/group. Two-way ANOVA with Tukey multi-comparison test was applied. ns, not significant. (**D**) Representative images of lung metastasis. (**E**) Quantification of the ex vivo SK-MEL-147 GFPLuc lung metastatic burden by measuring the average radiance per lobule by IVIS at endpoint. Two-way ANOVA were used to analyze tumor growth (**C**). Average radiance/lobule was calculated by one-way ANOVA with Tukey correction for multi-comparison (**E**). ns, not significant. Source data are available online for this figure.

 © The Author(s)

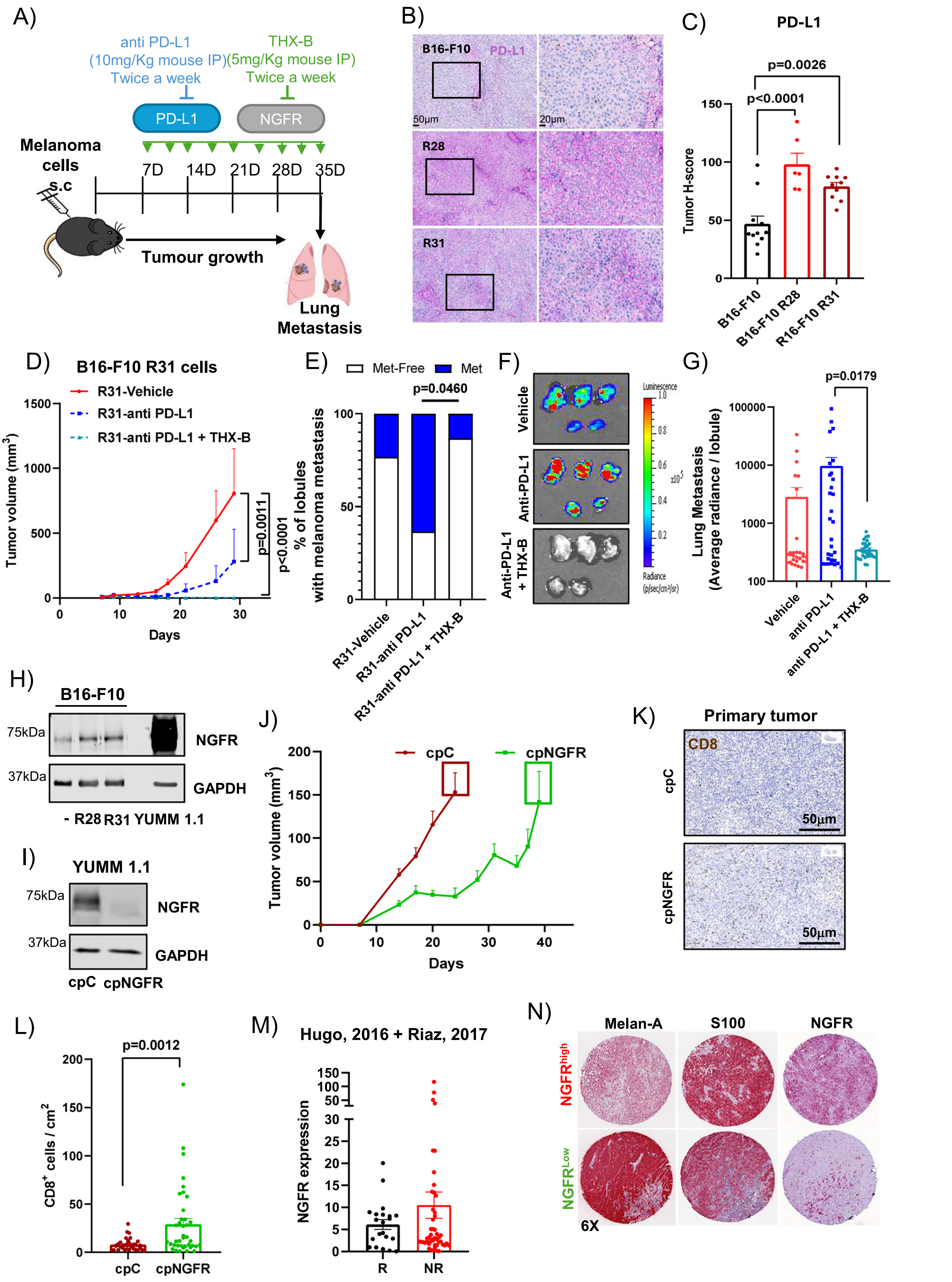
A)
anti PD-L1
(10mg/Kg mouse IP)
Twice a week
THX-B
(5mg/Kg mouse IP)
Twice a week
PD-L1
NGFR
Melanoma cells s.c
7D 14D 21D 28D 35D
Tumour growth
Lung Metastasis
B)
B16-F10 PD-L1
R28
R31
50µm
20µm
C)
PD-L1
p=0.0026
p<0.0001
Tumor H-score
B16-F10
B16-F10 R28
R16-F10 R31
D)
B16-F10 R31 cells
R31-Vehicle
R31-anti PD-L1
R31-anti PD-L1 + THX-B
Tumor volume (mm3)
p=0.0011
p<0.0001
Days
E)
Met-Free
Met
p=0.0460
% of lobules with melanoma metastasis
R31-Vehicle
R31-anti PD-L1
R31-anti PD-L1 + THX-B
F)
Vehicle
Anti-PD-L1
Anti-PD-L1 + THX-B
Luminescence
Radiance (p/sec/cm²/sr)
G)
p=0.0179
Lung Metastasis (Average radiance / lobule)
Vehicle
anti PD-L1
anti PD-L1 + THX-B
H)
B16-F10
75kDa
37kDa
NGFR
GAPDH
- R28 R31 YUMM 1.1
I)
YUMM 1.1
NGFR
GAPDH
cpC cpNGFR
J)
cpC
cpNGFR
Tumor volume (mm3)
Days
K)
Primary tumor
CD8
cpC
cpNGFR
50µm
L)
p=0.0012
CD8+ cells / cm2
cpC
cpNGFR
M)
Hugo, 2016 + Riaz, 2017
NGFR expression
R
NR
N)
Melan-A
S100
NGFR
NGFRhigh
NGFRLow
6X

Figure EV2. Targeting NGFR restores immunotherapy responsiveness and promotes intratumoral CD8⁺ T-cell recruitment of IT-resistant tumors.

(**A**) Scheme of the experiment performed to study the effect of the THX-B and anti-PD-L1 combination therapy in melanoma subcutaneous immunocompetent models. Dosages of treatment are indicated. Treatments were administrated as monotherapy or in combination intraperitoneally twice a week beginning 7 days after tumor cell injection. (**B**, **C**) PD-L1 expression was measured by IHC of B16-F10 control and anti-PD-L1-resistant (R28 and R31) tumors. $n = 6$–11 different fields from 3 to 6 tumors /group. (**B**) Representative IHC image of PD-L1 staining in different groups. Black square indicates magnified area in the right. (**C**) Qupath quantification of intra-tumor PD-L1 expression. Data were mean + SEM. (**D–G**) B16-F10-R31 cells (200,000) were injected into the flank of B6 mice. On day 7, treatment started as indicated in (**A**) ($n = 6$ mice/group). (**D**) Tumor growth curves showing mean + SEM. (**E**) Percentage of lung lobules with melanoma metastases. (**F**) Representative IVIS image. (**G**) Quantification of the average radiance/lobule. Data were mean + SEM. ($n = 6$ mice/group). 1 (**H**) NGFR expression in melanoma cell lines with acquired (B16-F10 R28 and R31) or intrinsic (Yumm1.1) immunotherapy resistance. A representative Western blot is shown. (**I**) Representative western blot of the efficient KO of NGFR in Yumm1.1 cells. (**J**) Tumor growth of cpC and cpNGFR YUMM1.1 tumors in B6 mice sacrificed at the same tumor size (100–300 mm^3). The square indicates the mean size of the tumors analyzed by IHC. Data were mean + SEM of $n = 12$ tumors from 6 mice (injected in both flanks). (**K**) Representative image of $CD8^+$ IHC staining in tumors from (**J**). Scale bar (50 µm) is indicated. (**L**) The number of $CD8^+$ T cells was calculated from 3 to 4 consecutive slides/tumor using QuPath-0.5.1 software and normalized to the area of the tumor. Data were mean + SEM. (**M**) NGFR expression in responders (R) or non-responders (NR) to anti-PD-1 therapies from datasets GSE78220 (Hugo et al, 2016) and GSE91061 (Riaz et al, 2017). Mean + SEM of $n = 70$ patients is represented. (**N**) Representative immunohistochemistry of TMAs from melanoma patients under immunotherapy. Serial sections stained for Melan-A, S100 and NGFR are shown. Upper panel: full TMA 258. Red and green boxes indicate representative cores with high or low NGFR expression, respectively. Objective magnification as indicated. (**N**) Statistical analyses performed were one-way ANOVA (**C**), Two-way ANOVA (**D**) and unpaired Student *t*-test (**E**, **G**, **I**). Tukey correction was used for multiple comparison. Source data are available online for this figure.

 © The Author(s)

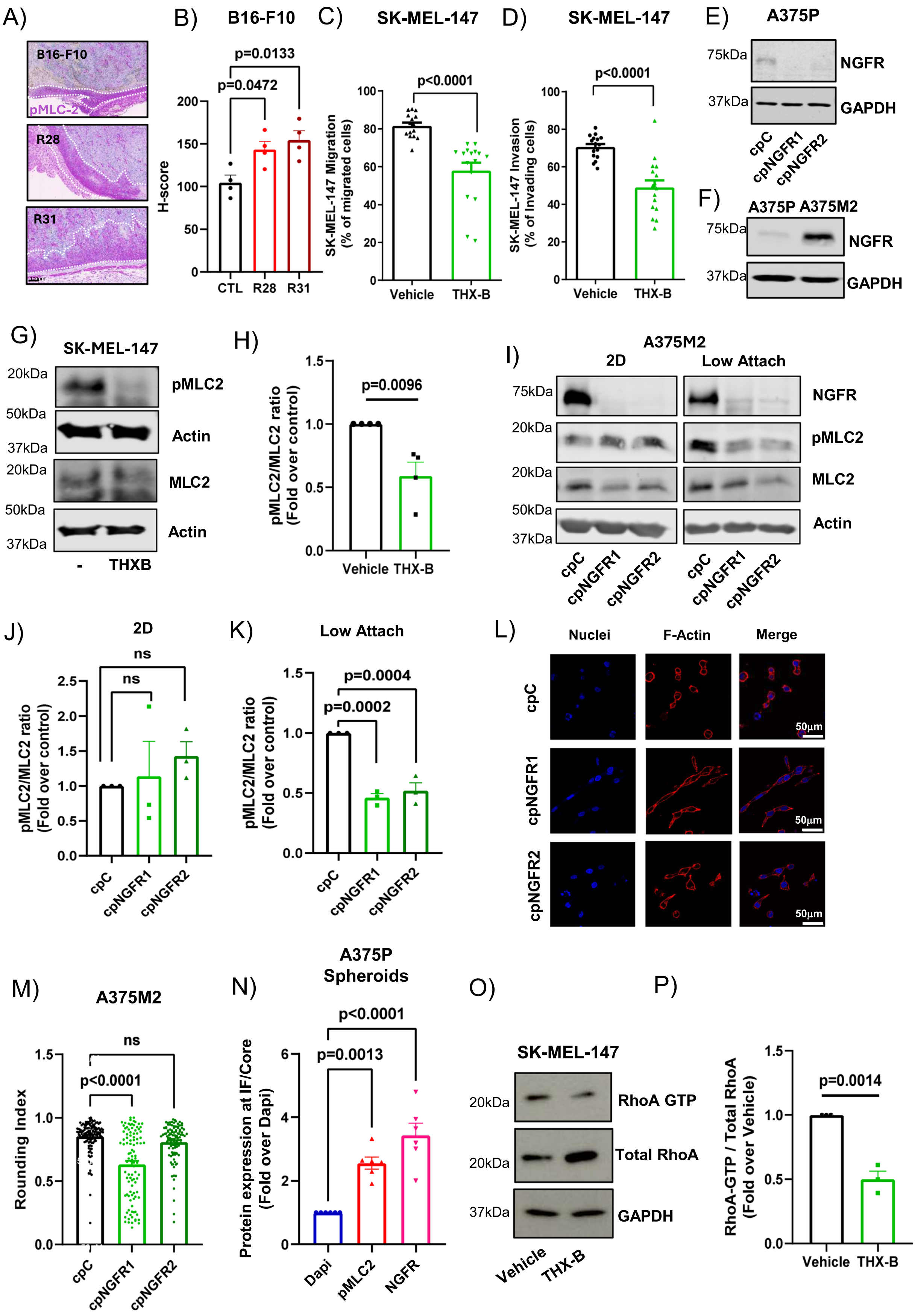
A)
B16-F10
pMLC-2
R28
R31
B) B16-F10
p=0.0133
p=0.0472
H-score
CTL R28 R31
C) SK-MEL-147
p<0.0001
SK-MEL-147 Migration (% of migrated cells)
Vehicle THX-B
D) SK-MEL-147
p<0.0001
SK-MEL-147 Invasion (% of Invading cells)
Vehicle THX-B
E) A375P
75kDa NGFR
37kDa GAPDH
cpC cpNGFR1 cpNGFR2
F) A375P A375M2
75kDa NGFR
37kDa GAPDH
G) SK-MEL-147
20kDa pMLC2
50kDa Actin
37kDa
20kDa MLC2
50kDa Actin
37kDa
- THXB
H)
p=0.0096
pMLC2/MLC2 ratio (Fold over control)
Vehicle THX-B
I) A375M2
2D Low Attach
75kDa NGFR
20kDa pMLC2
20kDa MLC2
50kDa Actin
37kDa
cpC cpNGFR1 cpNGFR2 cpC cpNGFR1 cpNGFR2
J) 2D
ns
ns
pMLC2/MLC2 ratio (Fold over control)
cpC cpNGFR1 cpNGFR2
K) Low Attach
p=0.0004
p=0.0002
pMLC2/MLC2 ratio (Fold over control)
cpC cpNGFR1 cpNGFR2
L)
Nuclei F-Actin Merge
cpC
cpNGFR1
cpNGFR2
50µm
M) A375M2
ns
p<0.0001
Rounding Index
cpC cpNGFR1 cpNGFR2
N) A375P Spheroids
p<0.0001
p=0.0013
Protein expression at IF/Core (Fold over Dapi)
Dapi pMLC2 NGFR
O)
SK-MEL-147
20kDa RhoA GTP
20kDa Total RhoA
37kDa GAPDH
Vehicle THX-B
P)
p=0.0014
RhoA-GTP / Total RhoA (Fold over Vehicle)
Vehicle THX-B

© The Author(s)

Figure EV3. NGFR is required for the ROCK-MLC2 ameboid metastasis of human melanoma tumors.

(**A**) Representative IHC of pMLC2 activation at the invasive front of parental (CTL) or anti-PD-L1-resistant (R28 and R31) B16-F10 tumors. The invasive front (IF) was indicated by white dots. (**B**) Qu-Path quantification of pMLC2 expression at the IF (defined by white dots). One-way ANOVA with Tukey multi-comparison correction was performed. Representative data (Mean + SEM) from 1 experiment $n = 4$ mice replicated in 2 independent experiments. (**C**, **D**) Transwell SK-MEL-147 cell migration (**C**) and invasion (**D**) to FBS (% of total cells) after treatment with vehicle (DMSO 1.6%) or THX-B (20 µM, ON). Data were mean + SEM of four fields per condition of two independent experiments performed in duplicate. Student's *t*-test. (**E**) Representative Western blot of NGFR knockdown in A375P cells. (**F**) Representative Western blot showing increased NGFR expression levels in ameboid A375M2 cells compared to parental A375P cells. (**G**) Representative Western blot showing reduced pMLC2 levels in SK-MEL-147 cells upon THX-B treatment (20 µM, 24 h). (**H**) Quantification of the pMLC2/total MLC2 ratio and referred to SK-MEL-147 treated with vehicle. Data were mean + SEM of $n = 4$ independent experiments. Unpaired *t*-test was performed. (**I**) Representative Western blot of the effective CRISPR/Cas9 knockdown of NGFR in A375M2 cells and the activation of pMLC2 in 2D standard adhesion or low adhesion conditions. (**J**, **K**) Quantification of the pMLC2/total MLC2 ratio in A375M2 cpControl vs NGFR KO cells in 2D conditions (**J**) or low-attachment plates (**K**) Data were mean + SEM of $n = 3$ independent experiments. One-way ANOVA with Dunnett multi-comparison test was performed. ns not significant. (**L**) Confocal images of cpC, cpNGFR1 and cpNGFR2 A375M2 cells seeded on top of collagen for 24 h. (**M**) Quantification of rounded versus elongated cells. Data represent mean + SEM of 126, 102, and 108 cells from two independent experiments. One-way ANOVA with Dunnett correction was performed. ns, not significant. (**N**) Quantification of DAPI, NGFR, and pMLC2 protein expression at the invasive front of A375P-3D invasive spheroids upon 3 days of culture into collagen I matrix. Mean intensity was calculated as the ratio between the expression at the IF vs Core of the spheroid and represented as fold over the DAPI condition +SEM. $n = 6$ spheroids/group. One-way ANOVA analysis with Tukey multi-comparison was performed. (**O**, **P**) Pull down of RhoA-GTP in SK-MEL-147 cells treated with THX-B (20 µM, 24 h). (**O**) Representative Western blot. (**P**) RhoA-GTP relative activation to total RhoA expression is represented as fold over the untreated condition. $n = 3$ independent experiments were performed and represented as mean + SEM. An unpaired *t*-test (**P**) statistics test was applied. Source data are available online for this figure.

 © The Author(s)

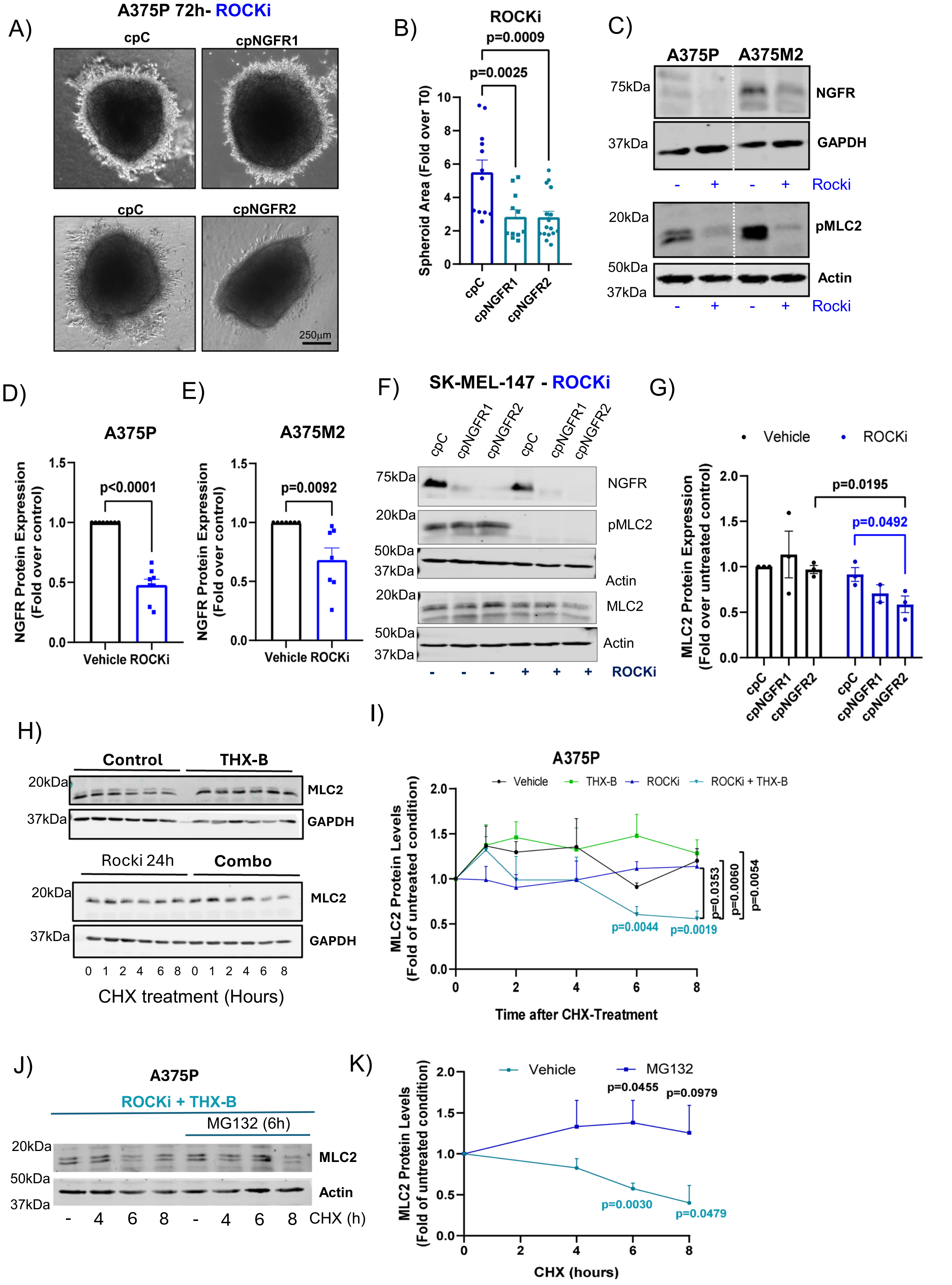
A)
A375P 72h- ROCKi
cpC
cpNGFR1
cpC
cpNGFR2
250µm
B)
ROCKi
p=0.0009
p=0.0025
Spheroid Area (Fold over T0)
cpC
cpNGFR1
cpNGFR2
C)
A375P
A375M2
75kDa
NGFR
37kDa
GAPDH
Rocki
20kDa
pMLC2
50kDa
Actin
37kDa
Rocki
D)
A375P
p<0.0001
NGFR Protein Expression (Fold over control)
Vehicle ROCKi
E)
A375M2
p=0.0092
NGFR Protein Expression (Fold over control)
Vehicle ROCKi
F)
SK-MEL-147 - ROCKi
cpC
cpNGFR1
cpNGFR2
cpC
cpNGFR1
cpNGFR2
75kDa
NGFR
20kDa
pMLC2
50kDa
37kDa
Actin
20kDa
MLC2
50kDa
Actin
37kDa
ROCKi
G)
Vehicle
ROCKi
p=0.0195
p=0.0492
MLC2 Protein Expression (Fold over untreated control)
cpC
cpNGFR1
cpNGFR2
cpC
cpNGFR1
cpNGFR2
H)
Control
THX-B
20kDa
MLC2
37kDa
GAPDH
Rocki 24h
Combo
20kDa
MLC2
37kDa
GAPDH
0 1 2 4 6 8 0 1 2 4 6 8
CHX treatment (Hours)
I)
A375P
Vehicle
THX-B
ROCKi
ROCKi + THX-B
MLC2 Protein Levels (Fold of untreated condition)
p=0.0044
p=0.0019
p=0.0353
p=0.0060
p=0.0054
Time after CHX-Treatment
J)
A375P
ROCKi + THX-B
MG132 (6h)
20kDa
MLC2
50kDa
Actin
37kDa
- 4 6 8 - 4 6 8 CHX (h)
K)
Vehicle
MG132
p=0.0455
p=0.0979
MLC2 Protein Levels (Fold of untreated condition)
p=0.0030
p=0.0479
CHX (hours)

Figure EV4. Depletion of NGFR in combination with ROCKi compromised MLC2 stability.

(**A**) Multicellular spheroids of cpC, cpNGFR1 and cpNGFR2 A375P (3×10^3cells) were prepared as in Fig. 4J–L in the presence of ROCKi (1 μM). Representative bright-field images of T2 (72 h) invasive spheroids upon ROCKi are shown. (**B**) Invasion area at T2 was normalized to mean invasion area at T0 and plotted with error bars representing ±SEM ($n = 11$–16 spheroids/group from two independent experiments). One-way ANOVA with Dunnett multiple comparison was performed. (**C**) Representative western blot of NGFR / GAPDH, pMLC2/Actin of A375P and A375M2 cells treated with ROCKi (1 μM) 24 h. (**D**, **E**) NGFR protein expression represented as fold over vehicle-treated condition in A375P (**D**) and A375M2 (**E**) cells. Data were mean + SEM of $n = 7$ and 8 independent experiments (A375M2 and A375P cells, respectively). Student's *t*-test was used as a statistical method. (**F**) Representative Western blot of NGFR, MLC2, and GAPDH of cpC, cpNGFR1, and cpNGFR2 SK-MEL-147 cells treated or not with ROCKi (1 μM) 24 h. (**G**) MLC2 protein expression was quantified and represented as fold over untreated cpC cells. Data were mean + SEM of 2–3 independent experiments. Student's *t*-test was used. (**H**) Representative immunoblots of A375P cells treated with vehicle, THX-B (20 μM), ROCKi (1 μM), and the combination of THX-B and ROCKi for 24 h. Cycloheximide (20 μg/ml) was added to cells for the indicated periods before cell lysis. Total MLC2 and GAPDH levels were shown. (**I**) MLC2 protein levels were normalized with GAPDH expression and depicted as fold of expression over 0 h of cycloheximide treatment. Data (mean + SEM.) from four independent experiments are shown. Unpaired *t*-test was performed comparing MLC2 expression at each time point vs time 0 of cycloheximide treatment. (**J**) Representative immunoblots of A375P cells treated with the combination of THX-B and ROCKi for 24 h and cycloheximide (20 μg/ml) for the indicated times. MG132 (10 μM) was added for 6 h to half of the wells to block proteasome-dependent degradation. Total MLC2 and GAPDH levels were shown. (**K**) MLC2 protein levels were normalized with GAPDH expression and depicted as a fold of expression over 0 h of cycloheximide treatment in the control group. Data (mean + SEM.) from four independent experiments are shown. Unpaired *t*-test was performed comparing the protein decay at each time point vs T0 of cycloheximide treatment (*p* in blue) and comparing protein levels at each point of MG132-treated vs untreated A375P cells (*p* in black). Source data are available online for this figure.

 © The Author(s)

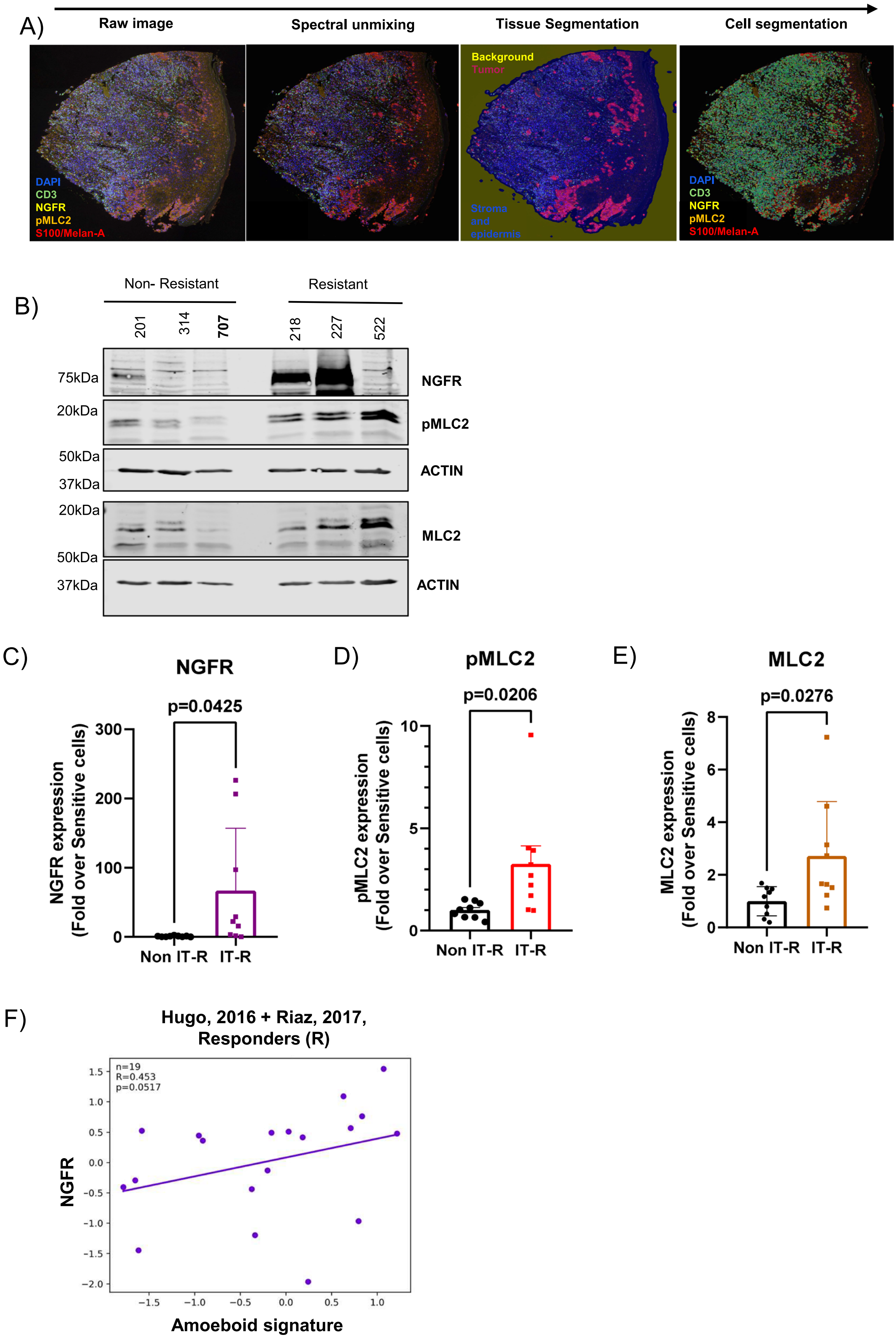
A)
Raw image
Spectral unmixing
Tissue Segmentation
Cell segmentation
DAPI
CD3
NGFR
pMLC2
S100/Melan-A
Background
Tumor
Stroma and epidermis
B)
Non- Resistant
Resistant
201
314
707
218
227
522
75kDa
20kDa
50kDa
37kDa
NGFR
pMLC2
ACTIN
MLC2
C)
NGFR
p=0.0425
NGFR expression (Fold over Sensitive cells)
Non IT-R
IT-R
D)
pMLC2
p=0.0206
pMLC2 expression (Fold over Sensitive cells)
E)
MLC2
p=0.0276
MLC2 expression (Fold over Sensitive cells)
F)
Hugo, 2016 + Riaz, 2017, Responders (R)
n=19
R=0.453
p=0.0517
NGFR
Amoeboid signature

Figure EV5. NGFR and pMLC2 are enriched in patient-derived immunotherapy-resistant cell lines.

(**A**) Representative images of a core of TMAs from primary melanomas illustrating the analysis pipeline performed with the *inForm*® software. Different staining and tissue sections are indicated in colors. (**B**) Representative immunoblots of NGFR, pMLC2, MLC2, and Actin of non-immunotherapy resistant vs immunotherapy-resistant cell lines derived from metastatic samples from melanoma patients. (**C–E**) Quantification of the NGFR expression (**B**), pMLC2/MLC2 ratio (**C**), and MLC2 total levels (**D**) in cells indicated in (**A**). Data were mean + SEM of cell lysates grouped as non-IT-resistant and IT-resistant cells of three different lines in three independent experiments. Student's *t*-test was performed. (**F**) Associations between NGFR and the ameboid signature in responder patients ($n = 19$) were quantified using Spearman's rank correlation (R) with corresponding p values. Results were visualized using scatter plots with a fitted linear trend line and annotation of n, R, and p value. Source data are available online for this figure.

 © The Author(s)
