# Supplementary material for: NGFR induces melanoma invasion and immunotherapy resistance through myosin light chain 2 modulation
Source: EMBO J. 2026 May 26;45(14):4988–5023. doi: 10.1038/s44318-026-00803-2 (PMC13373201; doi:10.1038/s44318-026-00803-2)
Supplement: Supplementary file 3 — Peer Review File [file 44318_2026_803_MOESM3_ESM.pdf]

THE EMBO JOURNAL

NGFR induces melanoma invasion and immunotherapy resistance through myosin light chain 2 modulation

Laura Nogués, Vanesa Santos, Juan García-Agulló, Susana Garcia-Silva, Oscar Maiques, Alberto Hernández-Barranco, Marina Mazariegos, Manuel Pérez-Martinez, Raúl Torres-Ruíz, Annalisa Saltari, Patrick Turko, María Mannino, Ali Nejatie, Hassan Nassour, Diego Megías, Isabel Peset, Carmen Blanco-Aparicio, Sonia Martinez, Mitchell Levesque, H. Saragovi, Victoria Sanz-Moreno, and Hector Peinado

Corresponding author(s): Laura Nogués (lnogues@cbm.csic.es)

Review Timeline:

Submission Date:	17th Jul 25
Editorial Decision:	12th Aug 25
Revision Received:	13th Feb 26
Editorial Decision:	16th Mar 26
Revision Received:	14th Apr 26
Accepted:	29th Apr 26

Editor: Daniel Klimmeck

Transaction Report:

(Note: With the exception of the correction of typographical or spelling errors that could be a source of ambiguity, letters and reports are not edited. Depending on transfer agreements, referee reports obtained elsewhere may or may not be included in this compilation. Referee reports are anonymous unless the Referee chooses to sign their reports.)

Dear Dr Peinado,

Thank you again for the submission of your manuscript (EMBOJ-2025-121910) to The EMBO Journal, as well as for your patience with our feedback at this time. As mentioned earlier, your study was assessed by three reviewers with expertise in metastasis biology and skin cancer invasion, whose comments are enclosed below.

As you will see from the experts' reports, the referees acknowledge the analysis and potential interest and value of your findings. However, they also express a number of important issues which need to be addressed thoroughly to make them supportive of publication in the EMBO Journal. The reviewers raise a number of issues related to the additional controls and improved methods - cohort annotation required, statistics applied and overall discussion of related literature, that would need to be conclusively addressed to achieve the level of robustness and clarity needed for The EMBO Journal.

Given the overall interest stated and broader angle of your findings, we are able to invite you to revise your manuscript experimentally to address the referees' comments. However, please note that the extent of revisions requested appear threshold in our view for the amount of complementary work we typically invite for our venue; also, I need to stress that we do require strong support from the referees on a revised version of the study in order to move on to publication of the work.

Please feel free to contact me if you have any questions or need further input on the referee comments.

We generally allow three months as standard revision time. As a matter of policy, competing manuscripts published during this period will not negatively impact on our assessment of the conceptual advance presented by your study. However, we request that you contact the editor as soon as possible upon publication of any related work, to discuss how to proceed. Should you foresee a problem in meeting this three-month deadline, please let us know in advance and we may be able to grant an extension.

When submitting your revised manuscript, please carefully review the instructions below.

Please feel free to approach me any time should you have additional questions related to this.

Thank you for the opportunity to consider your work for publication.

I look forward to your revision.

Best regards,

Daniel Klimmeck

Daniel Klimmeck, PhD
Senior Editor
The EMBO Journal

Instruction for the preparation of your revised manuscript:

1) a .docx formatted version of the manuscript text (including legends for main figures, EV figures and tables). Please make sure that the changes are highlighted to be clearly visible.

2) individual production quality figure files as .eps, .tif, .jpg (one file per figure).

3) a .docx formatted letter INCLUDING the reviewers' reports and your detailed point-by-point response to their comments. As part of the EMBO Press transparent editorial process, the point-by-point response is part of the Review Process File (RPF), which will be published alongside your paper.

4) a complete author checklist, which you can download from our author guidelines (https://wol-prod-cdn.literatumonline.com/pb-assets/embo-site/Author Checklist%20-%20EMBO%20J-1561436015657.xlsx). Please insert information in the checklist that is also reflected in the manuscript. The completed author checklist will also be part of the RPF.

5) Please note that all corresponding authors are required to supply an ORCID ID for their name upon submission of a revised manuscript.

6) It is mandatory to include a 'Data Availability' section after the Materials and Methods. Before submitting your revision, primary datasets produced in this study need to be deposited in an appropriate public database, and the accession numbers and database listed under 'Data Availability'. Please remember to provide a reviewer password if the datasets are not yet public (see https://www.embopress.org/page/journal/14602075/authorguide#datadeposition).
In case you have no data that requires deposition in a public database, please state so in this section. Note that the Data Availability Section is restricted to new primary data that are part of this study.
*** Note - All links should resolve to a page where the data can be accessed. ***

7) Our journal encourages inclusion of *data citations in the reference list* to directly cite datasets that were re-used and obtained from public databases. Data citations in the article text are distinct from normal bibliographical citations and should directly link to the database records from which the data can be accessed. In the main text, data citations are formatted as follows: "Data ref: Smith et al, 2001" or "Data ref: NCBI Sequence Read Archive PRJNA342805, 2017". In the Reference list, data citations must be labeled with "[DATASET]". A data reference must provide the database name, accession number/identifiers and a resolvable link to the landing page from which the data can be accessed at the end of the reference. Further instructions are available at .

8) At EMBO Press we ask authors to provide source data for the main and EV figures. Our source data coordinator will contact you to discuss which figure panels we would need source data for and will also provide you with helpful tips on how to upload and organize the files.

Numerical data can be provided as individual .xls or .csv files (including a tab describing the data). For 'blots' or microscopy, uncropped images should be submitted (using a zip archive or a single pdf per main figure if multiple images need to be supplied for one panel). Additional information on source data and instruction on how to label the files are available at .

9) We replaced Supplementary Information with Expanded View (EV) Figures and Tables that are collapsible/expandable online (see examples in https://www.embopress.org/doi/10.15252/embj.201695874). A maximum of 5 EV Figures can be typeset. EV Figures should be cited as 'Figure EV1, Figure EV2" etc. in the text and their respective legends should be included in the main text after the legends of regular figures.

- For the figures that you do NOT wish to display as Expanded View figures, they should be bundled together with their legends in a single PDF file called *Appendix*, which should start with a short Table of Content. Appendix figures should be referred to in the main text as: "Appendix Figure S1, Appendix Figure S2" etc. See detailed instructions regarding expanded view here: .

- Additional Tables/Datasets should be labelled and referred to as Table EV1, Dataset EV1, etc. Legends have to be provided in a separate tab in case of .xls files. Alternatively, the legend can be supplied as a separate text file (README) and zipped together with the Table/Dataset file.

10) When assembling figures, please refer to our figure preparation guideline in order to ensure proper formatting and readability in print as well as on screen:
http://bit.ly/EMBOPressFigurePreparationGuideline

Please remember: Digital image enhancement is acceptable practice, as long as it accurately represents the original data and conforms to community standards. If a figure has been subjected to significant electronic manipulation, this must be noted in the figure legend or in the 'Materials and Methods' section. The editors reserve the right to request original versions of figures and the original images that were used to assemble the figure.

11) For data quantification: please specify the name of the statistical test used to generate error bars and P values, the number (n) of independent experiments (specify technical or biological replicates) underlying each data point and the test used to calculate p-values in each figure legend. The figure legends should contain a basic description of n, P and the test applied. Graphs must include a description of the bars and the error bars (s.d., s.e.m.).

Please remember: Digital image enhancement is acceptable practice, as long as it accurately represents the original data and conforms to community standards. If a figure has been subjected to significant electronic manipulation, this must be noted in the figure legend or in the 'Materials and Methods' section. The editors reserve the right to request original versions of figures and the original images that were used to assemble the figure.

Further information is available in our Guide For Authors: https://www.embopress.org/page/journal/14602075/authorguide

We realize that it is difficult to revise to a specific deadline. In the interest of protecting the conceptual advance provided by the work, we recommend a revision within 3 months (10th Nov 2025). Please discuss the revision progress ahead of this time with

the editor if you require more time to complete the revisions. Use the link below to submit your revision:

https://emboj.msubmit.net/cgi-bin/main.plex

Referee #1:

The authors of the study titled "NGFR induces melanoma invasion and immunotherapy resistance through Myosin II modulation" demonstrated that NGFR is expressed at the invasive front of melanoma tumors and that it regulates the MCL2 phosphorylation via RhoA-ROCK pathway and that together with ROCK it stabilizes Myosin II protein reinforcing its stability. They also showed that higher expression of NGFR in patients samples correlated with worse progression free survival. By using multiple melanoma mouse models, they demonstrated that targeting NGFR by THB-X alone or in combination with aPDL-1 therapy reduces distant metastasis, but not the tumor growth. Moreover, they showed that treatment with THX-B increases the infiltration of CD8 T cells and sensitize the tumors to immunotherapy.

Overall, the study is interesting, and its novelty lies in linking NGFR, MLC2, and the immune-resistant phenotype at the invasive fronts of primary and metastatic melanoma tumours. While the in vivo and in vitro experiments were thoroughly performed, I have some minor comments regarding the analyses of the human samples and a minor discussion point.

1. Human cohort of 46 patients
a. The methodology for analysing this cohort is unclear. More detail in the Methods section would make the manuscript easier to follow.
b. Please clarify how the adjustment for $S100^+$ and $MLANA^+$ was performed.
c. Including a representative images of the stainings would strengthen the paper.
d. Detailed cohort information is lacking, e.g., number of females/males, patient age range, etc.
e. The authors should also consider analyses on publicly available datasets that include melanoma samples undergoing immunotherapy, particularly single-cell datasets.
2. TMA data of the two patient cohorts
a. There is insufficient information on how the TMA data were analysed. How exactly was NGFR expression assessed?
b. How was the invasive front defined?
3. Discussion point
The authors could expand the discussion by addressing potential inducers of NGFR expression in melanoma cells at the invasive front. This induction likely differs substantially between spheroid experiments and the in vivo setting, where melanoma cells are in close proximity to various TME cell.

Referee #2:

In this study, Nogués et al. have conducted very comprehensive studies to investigate the role of NGFR in the invasive phenotype of melanomas, and have begun to address the potential molecular mechanisms involved, implicating MLC and the RhoA pathway. This study uses a robust combination of analyses in various melanoma cell lines and patient-derived material, the use of genetic and pharmacological modulation approaches, and complementary studies using cultured cells and in vivo models. The manuscript is very clear and well organized, and the work sheds light on factors previously unappreciated during melanoma progression/metastasis with important clinical implications. There are some aspects of the manuscript, as presented, that require attention, as they are also important for the interpretation of some data. Specific comments are listed below.

1. In the text and in Supplemental Fig. 2a, it is not clear if the anti-PD-L1 administration occurred only once, and at what interval following tumour implantation.

2. The experiments shown in Fig. 2a-2c show a greater proportion of metastasis-free lung lobules with the combined anti-PD-L1 + THX-B treatment. In this experiment, no group treated with only THX-B was included. This is important for the interpretation of the experiment. The effects of the treatment combination shown in Fig. 2a-2c appears to be very similar to those shown by THX-B treatment alone (Fig. 1i-1k). An alternative interpretation of the data in Fig. 2a-2c is that anti-PDL-1 treatment has no effect in this model, and that it does not influence the effects of THX-B either. That is, the anti-tumorigenic effects observed result exclusively from the presence of THX-B, and that the effects of THX-B monotherapy are not substantially different from those of the combination therapy. Thus, based on the data shown, it is not obvious that THX-B improves anti-PDL-1 treatment. Similar considerations apply to the experiments shown in Fig. 2k-2m.

3. The immunofluorescence experiments of Figs. 5a-5d appear to be consistent on decreased pMLC2 immunoreactivity in cells in which NGFR pathways are impaired. However, it is difficult to fully interpret these data, without a clearer quantification of pMLC2 levels. The experiments of Figs. 5a-5d need to be complemented by immunoblot analysis to better show the magnitude

of the changes in pMLC2 levels, and also whether those changes are associated with alterations in total cellular MLC2 abundance, or specifically with alterations in phosphorylation.

4. The data shown in Supplemental Fig. 5f-5g are not convincing. There appear to be only two points that gave rise to the histogram, and it is impossible to have a proper statistical analysis when n=2.

5. The experiments of Fig. 6h and Supplemental Fig. 6F are difficult to interpret, as they require vehicle-treated control samples. As presented, it is not clear that the changes in MLC2 are specifically associated with Rock1 inhibition.

6. The data shown in the immunoblots of Fig. 6J appear to originate from different blots, as suggested by the different banding pattern of each individual panel. If they arise from different blots, then appropriate loading controls for each blot should be included for accurate quantification and evaluation of the data.

Minor comments:

1. It would be helpful if all immunoblots shown included positions of molecular mass markers.

2. The Y axis of Fig. 3e needs to be re-labelled. The graph appears to show the percentage of animals that survive over time, not the percentage of tumours that survive over time.

3. In the "Cell culture on thick layers of collagen 1" section in the Methods, the units need to be revised for tail collagen concentrations and coverslip size, as the units are erroneously given in terms of volume (cm3 and mm3, respectively).

Referee #3:

The study presented by Nogués. Et al. identify NGFR as a central regulator of melanoma cell invasion and immunotherapy resistance through activation and stabilization of Myosin II via the RhoA-ROCK-MLC2 pathway. Using in vitro models, in vivo experiments, and patient-derived samples, the authors demonstrate that pharmacological inhibition of NGFR with THX-B reduces metastasis and restores sensitivity to anti-PD-L1 therapy, suggesting a novel combinatorial strategy for overcoming immune resistance.

The study is well designed, moving from genetic and pharmacological perturbations to preclinical models and human patient validation. It proposes a mechanistically novel connection between NGFR signaling and actomyosin contractility (via ROCK-MLC2), implicating this axis in both invasion and immune evasion.
The use of immunotherapy-resistant cell lines derived from patients provides strong clinical relevance.
Results are coherent across systems, and the potential therapeutic value of targeting NGFR to overcome resistance is clearly supported.

However, there are some weaknesses in the study that may need to be addressed:

• The specificity of THX-B is not explicitly controlled in this manuscript. Although THX-B has been previously characterized as a selective NGFR inhibitor, no data are shown here to confirm that its observed effects are NGFR-dependent. It would be good if the authors show the lack of effects in NGFR-KO cells, or rescue experiments.
• The authors provide strong indirect evidence that MLC2 is degraded via the proteasome when NGFR and ROCK are inhibited, using CHX chase and MG132 rescue experiments. However, they do not directly demonstrate MLC2 ubiquitination. Since the conclusion points to a novel post-translational regulatory mechanism, the inclusion, or at least discussion, of potential ubiquitination pathways would strengthen the mechanistic depth.
• The potential systemic or immune-cell-specific effects of THX-B on macrophages or lymphocytes are not discussed, despite observed changes in $CD8^+$ T cell infiltration. It would be helpful if the authors could show or discuss whether THX-B acts exclusively on tumor cells.
• The use of B16-F10 models, while standard, may not fully recapitulate the complexity of human melanoma immune responses. A brief discussion of this limitation would be appropriate.

Dear Editors,

We appreciate the opportunity to revise and resubmit our manuscript. We thank the reviewers for their constructive comments and have revised the manuscript accordingly. We addressed all points raised and implemented the corresponding changes throughout the text and figures. Below we provide a point-by-point response

Referee #1:

The authors of the study titled "NGFR induces melanoma invasion and immunotherapy resistance through Myosin II modulation" demonstrated that NGFR is expressed at the invasive front of melanoma tumors and that it regulates the MCL2 phosphorylation via RhoA-ROCK pathway and that together with ROCK it stabilizes Myosin II protein reinforcing its stability. They also showed that higher expression of NGFR in patients samples correlated with worse progression free survival. By using multiple melanoma mouse models, they demonstrated that targeting NGFR by THB-X alone or in combination with aPDL-1 therapy reduces distant metastasis, but not the tumor growth. Moreover, they showed that treatment with THX-B increases the infiltration of CD8 T cells and sensitize the tumors to immunotherapy.

Overall, the study is interesting, and its novelty lies in linking NGFR, MLC2, and the immune-resistant phenotype at the invasive fronts of primary and metastatic melanoma tumours. While the in vivo and in vitro experiments were thoroughly performed, I have some minor comments regarding the analyses of the human samples and a minor discussion point.

1. Human cohort of 46 patients.

a. The methodology for analysing this cohort is unclear. More detail in the Methods section would make the manuscript easier to follow.

We expanded the Methods to provide a step-by-step description of how the 46-patient TMA cohort was quantified and analyzed. Briefly, TMA cores were de-arrayed in QuPath v0.4.4, H–DAB color deconvolution was applied to quantify staining intensities, and positivity was defined using a single global threshold; cell counts were normalized per mm^2.

b. Please clarify how the adjustment for $S100^+$ and $MLANA^+$ was performed.

We clarified this in Methods (“Image processing and analysis of the TMAs”). Because NGFR, S100 and MelanA were stained on consecutively cut sections, we used S100 and MelanA as tumour markers to estimate tumour content per core. Specifically, we computed **S100_MelanA_mean** as the mean of the fraction of $S100^+$ cells and the

fraction of MelanA^{+} cells in the corresponding core, and then calculated an adjusted NGFR score as:

Adjusted_NGFR_percent = NGFR_percent_positive × S100_MelanA_mean. This normalization accounts for variable tumour representation across cores and restricts NGFR quantification to tumour-enriched regions. We also provided the Raw data from these analyses in the revised version.

c. Including a representative image of the stainings would strengthen the paper.

As suggested, we added representative images to the Extended View (**new Fig. EV 2n**) to facilitate interpretation.

d. Detailed cohort information is lacking, e.g., number of females/males, patient age range, etc.

Following your suggestion, we added two summary tables (**new Table EV1 and Table EV2**) reporting the main clinical and pathological characteristics of the patient cohorts used in Fig. 3h and Fig. 7f–g.

e. The authors should also consider analyses on publicly available datasets that include melanoma samples undergoing immunotherapy, particularly single-cell datasets.

We thank the reviewer for suggesting the analysis of publicly available datasets. We focused on two series of melanoma patients (**GSE78220 and GSE91061, Hugo et al, 2016 and Riez et al, 2017 respectively)**, as validation cohorts representative of responders (R) or non-responders (NR) to immunotherapy in melanoma. In these datasets, NGFR expression showed a trend towards higher levels in non-responders (**new Fig. EV2m**). When patients were stratified by NGFR expression, the highest-expression quartile was enriched for non-responders (**new Fig. 3g)**. In addition, NGFR levels positively correlated with an amoeboid gene signature, with this association being particularly evident in immunotherapy-resistant cases (**new Fig. 7h–i and Fig. EV5f)**. Together, these analyses support the potential utility of NGFR (and an amoeboid-associated programme) to help identify patients less likely to respond to immune checkpoint therapy.

2. TMA data of the two patient cohorts.

a. There is insufficient information on how the TMA data were analysed. How exactly was NGFR expression assessed?

Apologies for the confusion. We have clarified this in the revised Methods by adding a dedicated section, "Image processing and analysis of the TMAs and included representative images in Extended View data (**Fig. EV 5a**).

In brief, NGFR expression in the TMA was quantified by digital pathology in QuPath v0.4.4. TMA cores (1 mm) were de-arrayed, tissue detection was used to exclude background/artefacts, and cells were segmented with Watershed Cell Detection. After H–DAB colour deconvolution, per-cell optical density was measured, and a single global threshold was applied to classify cells as $NGFR^+$ or $NGFR^-$; per-core NGFR positivity was reported as $NGFR^+$ cell density/fraction normalized to total detected cells per mm^2.

For the responder/non-responder cohort, consecutive sections were stained for NGFR, S100 and MelanA; tumour content per core was estimated as the mean fraction of $S100^+$ and $MelanA^+$ cells (S100_MelanA_mean), and NGFR was adjusted as NGFR_percent_positive × S100_MelanA_mean. Associations with time-to-progression (TTP) were tested using a mixed-effects Cox proportional hazards model in R (see Methods for full details)

b. How was the invasive front defined?

As now specified in Methods (Patient samples and Tissue microarray, TMA), TMA cores were selected per case from distinct regions corresponding to the tumor body (TB) and the invasive front (IF). The IF was defined histologically by board-certified pathologists based on H&E and/or MLANA staining (see below an example). Cores were annotated as TB (central tumor mass) or IF (peripheral region at the tumor–stroma interface where tumor cells infiltrate the surrounding tissue).

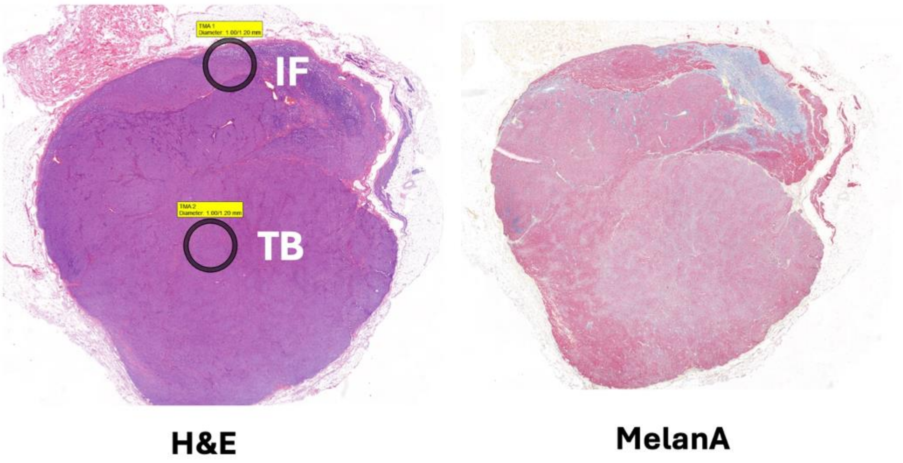

3. Discussion point. The authors could expand the discussion by addressing potential inducers of NGFR expression in melanoma cells at the invasive front. This induction likely differs substantially between spheroid experiments and the in vivo setting, where melanoma cells are in close proximity to various TME cell.

We expanded the Discussion to consider potential inducers of NGFR expression at the invasive front. Specifically, we discuss neurotrophin/NGF signaling as a plausible trigger and emphasize that NGFR regulation is likely context-dependent and may differ between spheroid assays and the in vivo tumor–stroma interface, where melanoma cells are exposed to paracrine cues from multiple TME cell types.

We also highlight that ECM remodeling and increased stiffness can enhance mechanotransduction through the RhoA–ROCK–Myosin II pathway, reinforcing amoeboid/invasive behavior and providing a biophysical route to NGFR^high states at invasive fronts, consistent with our proposed NGFR–ROCK axis.

Finally, we incorporated the inflammatory/stromal component that is largely absent from spheroids but prominent in vivo, including TNFα–NFκB signaling from immune/stromal cells, which has been linked to dedifferentiation and immune resistance and can connect inflammation to NGFR^high phenotypes.

Together, these additions provide a framework in which microenvironmental crosstalk (inflammatory cues and ECM mechanics) converges with tumor-intrinsic programes to engage the RhoA–ROCK–Myosin II machinery, thereby promoting invasion and reduced sensitivity to immunotherapy.

Reviewer#2:

In this study, Nogués et al. have conducted very comprehensive studies to investigate the role of NGFR in the invasive phenotype of melanomas and have begun to address the potential molecular mechanisms involved, implicating MLC and the RhoA pathway. This study uses a robust combination of analyses in various melanoma cell lines and patient-derived material, the use of genetic and pharmacological modulation approaches, and complementary studies using cultured cells and in vivo models. The manuscript is very clear and well organized, and the work sheds light on factors previously unappreciated during melanoma progression/metastasis with important clinical implications. There are some aspects of the manuscript, as presented, that require attention, as they are also important for the interpretation of some data. Specific comments are listed below.

1. In the text and in Supplemental Fig. 2a, it is not clear if the anti-PD-L1 administration occurred only once, and at what interval following tumour implantation.

Thanks for the comment. In the revised version we added a schematic of the treatment schedule (**new Fig. EV 2a**): anti–PD-L1 and THX-B were given i.p. twice weekly, as single agents or in combination, starting on day 7 post tumour cell injection.

2. The experiments shown in Fig. 2a-2c show a greater proportion of metastasis-free lung lobules with the combined anti-PD-L1 + THX-B treatment. In this experiment, no group treated with only THX-B was included. This is important for the interpretation of the experiment. The effects of the treatment combination shown in Fig. 2a-2c appears to be very similar to those shown by THX-B treatment alone (Fig. 1i-1k). An alternative interpretation of the data in Fig. 2a-2c is that anti-PDL-1 treatment has no effect in this model, and that it does not influence the effects of THX-B either. That is, the anti-tumorigenic effects observed result exclusively from the presence of THX-B, and that

the effects of THX-B monotherapy are not substantially different from those of the combination therapy. Thus, based on the data shown, it is not obvious that THX-B improves anti-PDL-1 treatment. Similar considerations apply to the experiments shown in Fig. 2k-2m.

We agree that the original presentation did not allow a clear assessment of the relative contribution of each treatment arm. We therefore updated Fig. 2a–d to include all groups in the same experiment (vehicle, anti–PD-L1, THX-B, and anti–PD-L1+THX-B), enabling a direct comparison of primary tumor growth and lung metastasis. In this setting, anti–PD-L1 primarily affects primary tumor growth but mice still develop metastasis, whereas THX-B has a stronger impact on metastatic burden; the combination provides control of both endpoints (**updated Fig. 2a–d)**.

We made the same adjustment for the experiments in Fig. 2k–m by adding the missing monotherapy control(s) (**updated Fig. 2j–m**). In the anti–PD-L1–resistant context, THX-B reduces metastatic burden, while the combination improves primary tumor control relative to either single agent.

3. The immunofluorescence experiments of Figs. 5a-5d appear to be consistent on decreased pMLC2 immunoreactivity in cells in which NGFR pathways are impaired. However, it is difficult to fully interpret these data, without a clearer quantification of pMLC2 levels. The experiments of Figs. 5a-5d need to be complemented by immunoblot analysis to better show the magnitude of the changes in pMLC2 levels, and also whether those changes are associated with alterations in total cellular MLC2 abundance, or specifically with alterations in phosphorylation.

As requested, we complemented the immunofluorescence analysis (Fig. 5a–d) with immunoblotting to quantify **pMLC2 and total MLC2** and to assess whether the effect reflects changes in phosphorylation versus total protein abundance. As shown in the **new Fig. EV 3g–h**, THX-B treatment in SK-MEL-147 cells significantly reduces pMLC2 levels (and the pMLC2/MLC2 ratio), consistent with the immunofluorescence results in Fig. 5a.

We expanded the analysis to cpNGFR knockout A375M2 and SK-MEL-147 cells. In cells plated on collagen, quantification of pMLC2 immunofluorescence showed a significant reduction upon NGFR depletion (**new Fig. 5 c-f**). Interestingly, immunoblotting showed no change in pMLC2/MLC2 under standard 2D adhesion but revealed a decrease under low-attachment conditions (**new Fig. EV 3i–k**). Together, these data support that NGFR modulates MLC2 phosphorylation in contexts relevant to amoeboid behavior.

4. The data shown in Supplemental Fig. 5f-5g are not convincing. There appear to be only two points that gave rise to the histogram, and it is impossible to have a proper statistical analysis when n=2.

We apologize for the omission and agree with your concern, we have now performed an additional assay to have 3 independent experiments and the proper statistical analysis **(New Fig. EV 3o-p**)

5. The experiments of Fig. 6h and Supplemental Fig. 6F are difficult to interpret, as they require vehicle-treated control samples. As presented, it is not clear that the changes in MLC2 are specifically associated with Rock1 inhibition.

We agree that vehicle-treated controls are required for proper interpretation. We have now included the missing vehicle controls in **Fig. 6h–i** and **Fig. EV 4f–g**. Our data show that the ROCKi decreases phosphorylated MLC2, but not the total levels of MLC2. However, combining ROCK inhibition with either genetic NGFR loss or pharmacological NGFR inhibition (THX-B) is associated with reduced total MLC2 levels (**Fig. 6h–k** and **Fig. EV 4f–k)**, supporting an additional mechanism of regulation.

6. The data shown in the immunoblots of Fig. 6J appear to originate from different blots, as suggested by the different banding pattern of each individual panel. If they arise from different blots, then appropriate loading controls for each blot should be included for accurate quantification and evaluation of the data.

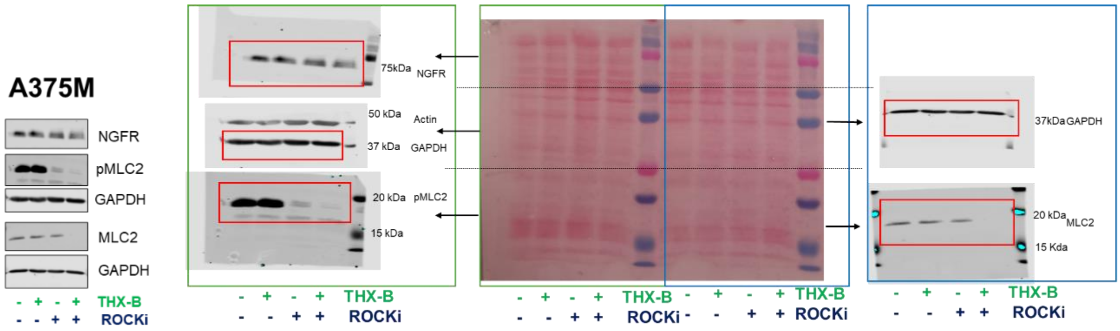

We agree that this was not sufficiently clear from the figure. The pMLC2 and total MLC2 panels in Fig. 6j were obtained from the **same samples, same gel and the same transferred membrane, using the recommended methodological approach used** in previously published work from our collaborator Dr. Sanz Moreno group (e.g.,Orgaz et al, *Cancer Cell 2020*; Georgouli et al, *Cell 2019)*. After transfer, the membrane was **cut and probed separately** for pMLC2 and total MLC2, which explains the different background/banding appearance.

Following the reviewer’s suggestion, we have now **included the corresponding loading controls** for these panels in the revised figure, and we provide the full membranes/ponceau staining in the raw data for transparency.

Minor comments:

1. It would be helpful if all immunoblots shown included positions of molecular mass markers.

We have added the molecular weight (kDa) of the molecular mass marker proximal to all the bands shown in the manuscript. In addition, we will enclose all the uncropped western blots and all loading controls or ponceaus from the figures in the raw data file, including the molecular mass markers.

2. The Y axis of Fig. 3e needs to be re-labelled. The graph appears to show the percentage of animals that survive over time, not the percentage of tumours that survive over time.

Thank you very much for noticing this error. We have corrected it by replacing ‘tumor survival’ with ‘mice survival’ in **Figure 3f**

3. In the "Cell culture on thick layers of collagen 1" section in the Methods, the units need to be revised for tail collagen concentrations and coverslip size, as the units are erroneously given in terms of volume (cm3 and mm3, respectively).

Thank you again for noticing this error. We have corrected it indicating **5μg/ml of Rat Tail type I collagen (Millipore 08-115) was placed onto 12mm diameter coverslips.**

Referee #3:

The study presented by Nogués. Et al. identify NGFR as a central regulator of melanoma cell invasion and immunotherapy resistance through activation and stabilization of Myosin II via the RhoA-ROCK-MLC2 pathway. Using in vitro models, in vivo experiments, and patient-derived samples, the authors demonstrate that pharmacological inhibition of NGFR with THX-B reduces metastasis and restores sensitivity to anti-PD-L1 therapy, suggesting a novel combinatorial strategy for overcoming immune resistance. The study is well designed, moving from genetic and pharmacological perturbations to preclinical models and human patient validation. It proposes a mechanistically novel connection between NGFR signaling and actomyosin contractility (via ROCK-MLC2), implicating this axis in both invasion and immune evasion. The use of immunotherapy-resistant cell lines derived from patients provides strong clinical relevance. Results are coherent across systems, and the potential

therapeutic value of targeting NGFR to overcome resistance is clearly supported. However, there are some weaknesses in the study that may need to be addressed.

• The specificity of THX-B is not explicitly controlled in this manuscript. Although THX-B has been previously characterized as a selective NGFR inhibitor, no data are shown here to confirm that its observed effects are NGFR-dependent. It would be good if the authors show the lack of effects in NGFR-KO cells, or rescue experiments.

We agree that demonstrating NGFR-dependence within this study strengthens the interpretation of THX-B effects. Based on your recommendation we added two additional experiments of NGFR loss-of-function control *in vivo*. First, we validated the impact of genetic NGFR KO reducing lung metastasis (**New Fig 1g-j).** Then, we treated tumors generated from control cells (NGFR^high) or NGFR-KO cells with THX-B. THX-B reduced metastatic burden in the NGFR^high group, whereas no additional reduction was observed in NGFR-KO tumours (**new EV1c–e**). These data support that the anti-metastatic activity of THX-B in our model is NGFR-dependent.

• The authors provide strong indirect evidence that MLC2 is degraded via the proteasome when NGFR and ROCK are inhibited, using CHX chase and MG132 rescue experiments. However, they do not directly demonstrate MLC2 ubiquitination. Since the conclusion points to a novel post-translational regulatory mechanism, the inclusion, or at least discussion, of potential ubiquitination pathways would strengthen the mechanistic depth.

We agree that identifying the ubiquitination pathway(s) underlying MLC2 turnover would further strengthen the mechanistic depth. In this manuscript, our data support proteasome-dependent regulation of MLC2 upon NGFR/ROCK inhibition (**CHX chase and MG132 rescue; Fig. 6**). We also obtained preliminary evidence that MLC2 can be ubiquitinated (MLC2 IP followed by K48-ubiquitin immunoblotting), with increased signal upon proteasome inhibition (**see below Rebuttal Figure 1**). However, we consider these ubiquitination data not yet sufficiently comprehensive to draw robust conclusions about the responsible ubiquitin linkage(s) and upstream enzymes. We therefore did not include them as a main mechanistic claim, but we have added a discussion placing these observations in context and highlighting this as an important direction for future work. We thank the reviewer for this suggestion.

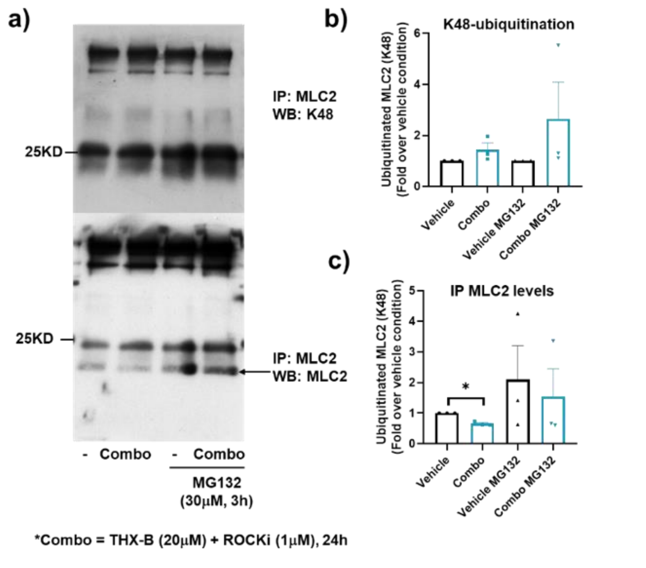

Rebuttal Figure 1. K48 MLC2 ubiquitination by NGFR/ROCK. A375M2 cells seeded on p100 plates were treated with vehicle or THX-B (20μM) and ROCKi (1μM) 24hours. When indicated, MG132 (30μM, 3h) was also added. Cells were lysed in 20 mM Tris pH7.4, 0.3M NaCl, 1% Tx-100, 1 mM EGTA, 10 mM MgCl2 and 10 mM ATP plus proteases and phosphatases inhibitors. After clearance, MLC2 was immunoprecipitated using MLC2 antibody (sc-376606, 2μg/μg of total protein). **a) Representative immunoblots of K48 and MLC2 levels. b) Quantification of MLC2 ubiquitination and total IP levels**. n=3 Unpaired T test was [illegible]

The potential systemic or immune-cell-specific effects of THX-B on macrophages or lymphocytes are not discussed, despite observed changes in $CD8^+$ T cell infiltration. It would be helpful if the authors could show or discuss whether THX-B acts exclusively on tumor cells.

We agree that potential systemic or immune cell–intrinsic effects of THX-B should be considered, particularly given the changes in $CD8^+$ T-cell infiltration. We have therefore added a Discussion paragraph addressing this point. Based on our *in vivo* data, THX-B retains anti-metastatic activity in nude mice, suggesting that its effect on metastatic burden does not require adaptive immunity, although we cannot exclude contributions from innate immune compartments (e.g Macrophages, Neutrophils or NK cells). Importantly, genetic NGFR depletion in tumor cells recapitulates the increase in $CD8^+$ infiltration, supporting that at least part of the immune phenotype is driven by tumor-cell NGFR signaling. Overall, our data are most consistent with a predominantly tumor-cell-dependent mechanism, while acknowledging that additional systemic and/or immune cell–intrinsic effects of THX-B remain possible. This is particularly relevant given that NGFR signaling has been implicated at the interface between neurotrophin pathways and immune regulation, we have now discussed these ideas, we thank the reviewer for this critical point raised.

• The use of B16-F10 models, while standard, may not fully recapitulate the complexity of human melanoma immune responses. A brief discussion of this limitation would be appropriate.

We agree with the reviewer that the B16-F10 model, while widely used for mechanistic and immunotherapy studies, does not fully capture the genomic heterogeneity, antigenic diversity and immune complexity of human melanoma. To mitigate this limitation, we validated our main conclusions across multiple complementary systems, including additional murine melanoma models (YUMM1.1, YUMMER1.7 and 5555), human melanoma models, patient-derived melanoma cells, and analyses of clinical cohorts. Together, these datasets support the robustness and translational relevance of our findings. We now explicitly acknowledge the limitations of any single murine model in the Discussion and highlight the need for future validation in additional genetically diverse, patient-derived and in vivo settings.

Dear Dr Peinado, dear Dr Nogues,

Thank you again for submitting your amended manuscript (EMBOJ-2025-121910R) to The EMBO Journal. Your revised study was sent back to the referees for their scientific reassessment, and we have received detailed re-reports from all of them, which I enclose below. As you will see, the referees state that the work has been substantially enhanced by the revisions and they are now in favour of publication, pending minor revision.

Thus, we are pleased to inform you that your manuscript has been accepted in principle for publication in The EMBO Journal.

Please carefully consider the remaining minor issues still raised by referee #1 by adjusting data presentation and discussion of the findings in the manuscript text where appropriate.

Also, we now need you to take care of a number of minor issues related to formatting and data annotation, which I will share shortly in a separate message, together with additional changes and requests by our production team for Source Data provision.

As you might have seen on our web page, every paper at the EMBO Journal now includes a 'Synopsis', displayed on the html and freely accessible to all readers. The synopsis includes a 'model' figure as well as 2-5 one-short-sentence bullet points that summarize the article. I would appreciate if you could provide this figure and the bullet points.

Please submit a revised version of the manuscript using the link enclosed below, addressing the advisor's comments.

Thank you again for giving us the chance to consider your manuscript for The EMBO Journal, I look forward to hearing from you and receiving your final revised version of the manuscript.

Best regards,

Daniel Klimmeck

Daniel Klimmeck PhD
Senior Editor
The EMBO Journal

****

Formatting changes required for the revised version of the manuscript:

>> please limit the abstract to maximally 175 words.

>> Author information: please clarify name discrepancy - M. P.-M. (manuscript) vs M. P. (online system); R.T.-R. (manuscript) vs R.T. (online system); C. B. (manuscript) vs C. B.-A. (online system); H. U. S. (manuscript) vs H. U. S. (online system).

>> Author Contributions: Remove the author contributions information from the manuscript text. Note that CRediT has replaced the traditional author contributions section as of now because it offers a systematic machine-readable author contributions format that allows for more effective research assessment. and use the free text boxes beneath each contributing author's name to add specific details on the author's contribution.

More information is available in our guide to authors.
https://link.springer.com/journal/44318/submission-guidelines

>> Adjust the title of the 'Conflict of Interest' section to 'Disclosure and Competing Interests Statement'.

- The manuscript sections should be in the following order: Title page - Abstract - Introduction - Results - Discussion - Methods - Data Availability - Acknowledgments - Disclosure and Competing Interests Statement - References - Figure Legends - (Main Tables with legends if applicable) - Expanded View Figure Legends.

>> Figure callouts: please add a callout for Fig. 6B.

>> Funding: Please add the complete funders information to the 'Acknowledgments' section; 'C33043/A24478 CRUK | Cancer

Research UK Therapeutic Discovery Laboratories (CRUK-TDL)' has to be added to the manuscript.

>> Add a Reagents and Tools table to the Methods section, as a separate file using the existing template in the Guide For Authors, listing key reagents, experimental models, software and relevant equipment.

>> Figures in separate files: Nomenclature in legends for EV figures in the manuscript needs to be adjusted to 'Figure EV1' etc. .

>> Figure 5 - label K is missing in figure caption. Please amend the caption.

>> Source Data: source data files need to be provided as one zip folder per figure each folder having clearly labelled panels; SD for Figure 4M is currently missing.

>> References: adjust the reference format to EMBO Journal format, alphabetical and 10 authors et al. . DOIs should only be used for preprints and datasets that have not been published yet. Add dataset references for the public GSE datasets used in the study.

>> Data availability section: Adjust the current statement to 'No novel large-scale data amenable to data repository deposition were generated in this study.'
Move current Public transcriptomic analyses information to the 'Data availability section'.

>> Author Checklist: adjust public dataset referencing information in the 'Data Availability ' section.

>> Dataset EV legends: Tables EV1 and EV2 need to be removed from ms and uploaded as separate files.

>> Please indicate redisplay of data from Figures between Figure 2L Vehicle and Figure 4F R28, in the figure legends of the latter figure.

>> Consider additional changes and comments from our production team as indicated below:

Figure Legends - Comments
- Please define the annotated p values ****/***/**/* as well as provide the exact p-values for the same in the legend of figure 5K as appropriate.
- Please note that the exact p values are not provided in the legends of figures 1B, E, H, J, M, O; 2A, B, D, F, I, J, K, M; 3A, C, D, E, F, G; 4B, C; 4B, C, D, E, H, J, L; 5B, D, F, G, I; 6B, C, D, F, G, I, K, M, O; 7B, D, F; EV1 E, EV2 C, D, E, G, L; EV3 B, C, D, H, K, M, N, P; EV4 B, D, E, G, I, K; EV5 C-E
- Please indicate the statistical test used for data analysis in the legends of figures 2F, I; 5K, 7E, EV1 C
- Please note that the box plots need to be defined in terms of minima, maxima, centre, bounds of box and whiskers, and percentile in the legends of figures 2I, 7B, D
- Please note that information related to n is missing in the legends of figures 3C, D, E; 4D, E; 5K, EV2 D
- Please note that the error bars are not defined in the legends of figures 3C, D, E; 4D, E; 5K, 6F, G, I, K; EV1 C, E; EV2 C, D, J, L, M; EV3 B, H, J, K, M, N, P"
- Please note that the scale bar needs to be defined for figures 4K, 5H, 7A, EV2 K
- Please note that scale bar and its definition are missing for figure 2H.

Further information is available in our Guide For Authors: https://link.springer.com/journal/44318/submission-guidelines

Please use the link below to submit your revision:

https://emboj.msubmit.net/cgi-bin/main.plex

Referee #1:

The authors have satisfactorily addressed my previous comments and substantially improved the clarity and robustness of the manuscript. I appreciate the additional explanations and clarifications provided throughout the revised version.

I have only a few minor remaining points:
1. In Figure EV2n, there is a small typographical error: "hihg" should read "high".
2. In the text, "Riez et al." should be corrected to "Riaz et al."
3. As the additional analyses of the human cohorts rely on bulk transcriptomic data, the authors should explicitly acknowledge in the discussion that the measured gene expression signals may be influenced by tumor microenvironment components. Consequently, some observed differences could partially reflect variation in non-malignant cell composition rather than tumor-intrinsic changes.

Referee #2:

In this second version of the manuscript, the authors have thoroughly and effectively addressed all the concerns raised previously. This study is clear and makes significant contributions to the mechanistic understanding of melanoma behaviour and potential sensitivity to various therapeutic regimes.

Referee #3:

The authors have carefully revised the manuscript and have addressed the major concerns raised in the previous round of review.

They now provide convincing evidence for the NGFR-dependence of THX-B activity by comparing its effects in NGFR-expressing and NGFR-knockout models, which strengthens the specificity of the proposed therapeutic mechanism. The mechanistic analysis of MLC2 stabilization has also been clarified. Although direct ubiquitination assays are not included, the cycloheximide chase experiments, proteasome inhibition rescue, and the expanded discussion provide sufficient support for a proteasome-dependent regulatory mechanism within the scope of the study.

The authors have appropriately discussed potential tumor-intrinsic versus immune-mediated effects and acknowledged the limitations of murine melanoma models. The combination of genetic, pharmacological, in vivo, and patient-derived data coherently supports the central conclusions.

Importantly, the study provides a conceptual link between NGFR signaling, actomyosin regulation, invasive behavior, and immunotherapy resistance. This mechanistic framework has broader relevance to tumor plasticity and therapy resistance beyond melanoma.

Overall, the conclusions are justified by the presented data, and I consider the manuscript suitable for publication pending minor revisions.

As you might have seen on our web page, every paper at the EMBO Journal now includes a 'Synopsis', displayed on the html and freely accessible to all readers. The synopsis includes a 'model' figure as well as 2-5 one-short-sentence bullet points that summarize the article. I would appreciate if you could provide this figure and the bullet points.

We provide synopsis figure and the bullet points as requested.

>> please limit the abstract to maximally 175 words

We updated a new version of abstract with 165 words.

>> Author information: please clarify name discrepancy
- M. P.-M. (manuscript) vs M. P. (online system); R.T.-R. (manuscript) vs R.T. (online system); C. B. (manuscript) vs C. B.-A. (online system); H. U. S. (manuscript) vs H. U. S. (online system).

We have attempted to update the authors' names in the online submission system; however, we are not authorized to modify the authors' profile information. Therefore, we kindly ask you to use the names as they appear in the text of the manuscript, which are as follows:

Manuel Pérez-Martinez
Raúl Torres-Ruíz
Diego Megías
Carmen Blanco-Aparicio
H. Uri Saragovi

>> Author Contributions: Remove the author contributions information from the manuscript text. Note that CRediT has replaced the traditional author contributions section as of now because it offers a systematic machine-readable author contributions format that allows for more effective research assessment. and use the free text boxes beneath each contributing author's name to add specific details on the author's contribution.

We have removed Author Contributions from main text of the manuscript and use the CRediT system available in your submission system.

>> Adjust the title of the 'Conflict of Interest' section to 'Disclosure and Competing Interests Statement'.

Title has been adjusted.

- The manuscript sections should be in the following order: Title page - Abstract - Introduction - Results - Discussion - Methods - Data Availability - Acknowledgments - Disclosure and Competing Interests Statement - References - Figure Legends - (Main Tables with legends if applicable) - Expanded View Figure Legends.

We have modified the section order based on your suggestions.

>> Figure callouts: please add a callout for Fig. 6B.

Please find the callout for figure 6b in the text as follows:

“We first compared the effect of ROCKi treatment on the invasive behavior of NGFR-expressing versus NGFR-KO cells. While ROCK inhibition reduced the invasive capacity of A375P spheroids, this effect was not statistically significant (Fig. 6a–b).”

>> Funding: Please add the complete funders information to the 'Acknowledgments' section; 'C33043/A24478 CRUK | Cancer Research UK Therapeutic Discovery Laboratories (CRUK-TDL)' has to be added to the manuscript.

We have completed V.S.M Lab funding:

V.S-M lab thanks Breast Cancer Now for funding this work as part of Programme Funding to the Breast Cancer Now Toby Robins Research Centre. V.S-M lab was further supported by The Institute of Cancer Research, Cancer Research UK (CRUK), C33043/A24478 Cancer Research UK Therapeutic Discovery Laboratories (CRUK-TDL); Barts Charity; Worldwide Cancer Research 22-0329 and UKRI grant reference EP/X033392.

>> Add a Reagents and Tools table to the Methods section, as a separate file using the existing template in the Guide For Authors, listing key reagents, experimental models, software and relevant equipment.

We included in the files the Reagents and Tools table filled according to your template.

>> **Figures in separate files: Nomenclature in legends for EV figures in the manuscript needs to be adjusted to 'Figure EV1' etc. .**

We have adjusted the nomenclature for EV figures in the legends and figures.

>> **Figure 5 - label K is missing in figure caption. Please amend the caption.**

Thanks for your comment, there was a labeling inconsistency between the figure and its caption that has been now corrected.

>> Source Data: source data files need to be provided as one zip folder per figure each folder having clearly labelled panels; SD for Figure 4M is currently missing.

There was a mistake in the author checklist. There is no figure 4M, they only reached 4L. It has been updated in the new source-data-checklist. Moreover, source data files are provided now as one zip folder per figure (i.e Fig1 or Fig EV1) with the panels clearly labelled as Number-Letter. (i.e 1A. 1B... or EV1A, EV1B...)

>> References: adjust the reference format to EMBO Journal format, alphabetical and 10 authors et al. . DOIs should only be used for preprints and datasets that have not been published yet. Add dataset references for the public GSE datasets used in the study.

The updated version of the manuscript includes References with EMBO Journal format. When used for analysis of GSE datasets [DATASET] is added at the end of the reference.

i.e: Hugo W, Zaretsky JM, Sun L, Song C, Moreno BH, Hu-Lieskovan S, Berent-Maoz B, Pang J, Chmielowski B, Cherry G *et al* (2016) Genomic and Transcriptomic Features of Response to Anti-PD-1 Therapy in Metastatic Melanoma. *Cell* 165: 35-44 [DATASET]

>> Data availability section: Adjust the current statement to 'No novel large-scale data amenable to data repository deposition were generated in this study.' Move current Public transcriptomic analyses information to the 'Data availability section'.

We have included the statement proposed and move the public transcriptomic analyses information to Data availability section.

>> Author Checklist: adjust public dataset referencing information in the 'Data Availability ' section.

An updated version of the Author Checklist has been uploaded with this modification.

>> Dataset EV legends: Tables EV1 and EV2 need to be removed from ms and uploaded as separate files.

Table EV1 and Table EV2 have been uploaded as separate files.

>> Please indicate redisplay of data from Figures between Figure 2L Vehicle and

Figure 4F R28, in the figure legends of the latter figure.

Thanks for the comment, we have modified figure 4e legend to clarify the data used for the analysis:

Figure 4e) Melanoma metastasis intensity measured by luciferase average radiance expression per lobule of each mouse. Data are mean + SEM. Unpaired *t*-test was performed. Figures e-f includes a redisplay of some of the data originally presented in Figure 2 a and I (vehicle treated groups), as required for comparative purposes.

>> Consider additional changes and comments from our production team as indicated below:

Figure Legends - Comments

- Please define the annotated p values ****/***/**/* as well as provide the exact p-values for the same in the legend of figure 5K as appropriate.
- Please note that the exact p values are not provided in the legends of figures 1B, E, H, J, M, O; 2A, B, D, F, I, J, K, M; 3A, C, D, E, F, G; 4B, C; 4B, C, D, E, H, J, L; 5B, D, F, G, I; 6B, C, D, F, G, I, K, M, O; 7B, D, F; EV1 E, EV2 C, D, E, G, L; EV3 B, C, D, H, K, M, N, P; EV4 B, D, E, G, I, K; EV5 C-E

Thanks for the suggestion. We have included exact p-values in the figures of the manuscript.

- Please indicate the statistical test used for data analysis in the legends of figures 2F, I; 5K, 7E, EV1 C

We have updated the figure legends of the indicated figures including the statistical test used.

- **Please note that the box plots need to be defined in terms of minima, maxima, centre, bounds of box and whiskers, and percentile in the legends of figures 2I, 7B, D**

We have defined the box and whiskers graphs for the indicated figures: Example: Figure 2I. Box plots show the mean (center), the 25th–75th percentiles (box), and the whiskers denote minimum and maximum values.

- Please note that information related to n is missing in the legends of figures 3C, D, E; 4D, E; 5K, EV2 D

Thanks for the comment. We have included the n of fig 3C. However, for the rest of experiments in figure 3 and 4, n was already indicated in the figure. I have highlighted here the n for the figures 3 and 4. Moreover, we have also indicated whether grouped experiments share the n. Figure 5k was also updated indicating the number of experiments performed.

3 c) The number of CD8+ T cells was calculated using QuPath-0.5.1 software and normalized by the number of cells. One way ANOVA with Tukey correction for multiple comparison was performed. *p<0.05; ***p<0.001; ****p<0.0001. n=13-17 tumor slides from mice of 2 independent approaches. **d)** 10^6 cpC or cpNGFR1 YUMM1.1 cells were subcutaneously injected into B16 mice. Tumor growth was measured using a caliper twice a week. N=9-10 mice per group from 2 independent experiments. Two-way ANOVA was performed. **** p< 0.0001. **e-f)** YUMMER1.7 tumors growth in syngeneic mice were evaluated using anti-PD-1 mAb, THX-B or their combination versus controls. Mice were inoculated by subcutaneous injections of 600000 tumor cells in the left flank. Once tumors were visible (10 days), anti-PD-1 (10 mg/Kg), THX-B (5mg/Kg) or a combination of anti-PD-1/THX-B, was intraperitoneal administrated once a week. n=10 mice, 2 independent experiments

4d) 300000 B16-F10 GFPLuc wild type or anti PD-L1 resistant R28 and R31 cells were injected in the flank of B6 mice. Tumor growth was measured twice a week. (n= 14-21 mice/ group from 2 (R31 or 3 (F10wt and R28) independent experiments. Two-way ANOVA statistical analysis with Tukey correction was used. ****p < 0.0001. **e-f)** Mice were sacrificed when tumors from control group reached 1000mm^3 and lungs were analyzed *ex vivo* at endpoint by measuring Luc expression at the IVIS system. n=6-12 mice / group from 1 (R31) or 2 (R28) or 3(F10wt) independent experiments.

- Please note that the error bars are not defined in the legends of figures 3C, D, E; 4D, E; 5K, 6F, G, I, K; EV1 C, E; EV2 C, D, J, L, M; EV3 B, H, J, K, M, N, P"

We have corrected this mistake and now errors bar are defined in the legends of all the experiments.

- Please note that the scale bar needs to be defined for figures 4K, 5H, 7A, EV2 K.

- Please note that scale bar and its definition are missing for figure 2H.

We have included scale bars in the figures.

We sincerely thank the reviewers for their positive and constructive feedback on the revised manuscript. We have modified the manuscript according to the minor comments raised by Referee #1 as indicated below:

Referee #1:

The authors have satisfactorily addressed my previous comments and substantially improved the clarity and robustness of the manuscript. I appreciate the additional explanations and clarifications provided throughout the revised version.

I have only a few minor remaining points:

1. In Figure EV2n, there is a small typographical error: "hihg" should read "high".
2. In the text, "Riez et al." should be corrected to "Riaz et al."

Thank you very much for letting us know. Errors have been corrected.

3. As the additional analyses of the human cohorts rely on bulk transcriptomic data, the authors should explicitly acknowledge in the discussion that the measured gene expression signals may be influenced by tumor microenvironment components. Consequently, some observed differences could partially reflect variation in non-malignant cell composition rather than tumor-intrinsic changes.

Thanks for the comment. We have included this suggestion in the following paragraph in the discussion section (highlighted in blue below):

Our previous work using melanoma patient's samples showed that NGFR expression is elevated in local metastatic sites such as lymph nodes. This increased expression correlates directly with poorer patient survival, indicating that NGFR is a reliable marker for early melanoma metastasis in lymph nodes(Garcia-Silva *et al.*, 2021). Moreover, although other studies have demonstrated a correlation between NGFR expression and immunotherapy resistance(Boshuizen *et al.*, 2020), its potential as a predictive marker for treatment response has not been analyzed. Building on these findings, our analyses indicate that higher tumor NGFR expression is associated with earlier relapse and reduced progression-free survival in immunotherapy-treated melanoma patients. Moreover, NGFR-based readouts at the invasive front of the tumors, including NGFR-to-pMLC2 metrics, were associated with subsequent development of distant metastasis and resistance to therapy. However, as some of the transcriptomic analyses rely on bulk RNA sequencing, these signals may also incorporate contributions from the tumor microenvironment in addition of those mediated by tumor-intrinsic NGFR expression.

Taken together, our results are consistent with NGFR being linked to metastatic progression and reduced benefit from immune checkpoint therapy, and they support the potential utility of NGFR / pMLC2 as stratification biomarkers to identify patients at higher risk of early metastasis and/or primary resistance—an approach that will require prospective validation.

Referee #2:

In this second version of the manuscript, the authors have thoroughly and effectively addressed all the concerns raised previously. This study is clear and makes significant contributions to the mechanistic understanding of melanoma behaviour and potential sensitivity to various therapeutic regimes.

Referee #3:

The authors have carefully revised the manuscript and have addressed the major concerns raised in the previous round of review.

They now provide convincing evidence for the NGFR-dependence of THX-B activity by comparing its effects in NGFR-expressing and NGFR-knockout models, which strengthens the specificity of the proposed therapeutic mechanism. The mechanistic analysis of MLC2 stabilization has also been clarified. Although direct ubiquitination assays are not included, the cycloheximide chase experiments, proteasome inhibition rescue, and the expanded discussion provide sufficient support for a proteasome-dependent regulatory mechanism within the scope of the study.

The authors have appropriately discussed potential tumor-intrinsic versus immune-mediated effects and acknowledged the limitations of murine melanoma models. The combination of genetic, pharmacological, in vivo, and patient-derived data coherently supports the central conclusions.

Importantly, the study provides a conceptual link between NGFR signaling, actomyosin regulation, invasive behavior, and immunotherapy resistance. This mechanistic framework has broader relevance to tumor plasticity and therapy resistance beyond

melanoma.

Overall, the conclusions are justified by the presented data, and I consider the manuscript suitable for publication pending minor revisions.

Dear Dr Nogués, dear Dr Peinado,

Thank you for submitting the revised version of your manuscript. I have now evaluated your amended manuscript and concluded that the remaining minor concerns have been sufficiently addressed.

I am thus pleased to inform you that your manuscript has been accepted for publication in the EMBO Journal.

Your manuscript will be processed for publication by EMBO Press. It will be copy edited and you will receive page proofs prior to publication. Please note that you will be contacted by Springer Nature Author Services to complete licensing and payment information.

Please note that it is The EMBO Journal policy for the transcript of the editorial process (containing referee reports and your response letter) to be published as an online supplement to each paper. If you do NOT want this, you will need to inform the Editorial Office via email immediately. More information is available here: https://www.embopress.org/transparent-process#Review_Process

You may qualify for financial assistance for your publication charges - either via a Springer Nature fully open access agreement or an EMBO initiative. Check your eligibility: https://www.embopress.org/page/journal/14602075/authorguide#chargesguide

Should you be planning a Press Release on your article, please get in contact with embo_production@springernature.com as early as possible in order to coordinate publication and release dates.

If you have any questions, please do not hesitate to contact the Editorial Office. Thank you for your contribution to The EMBO Journal.

Thank you again for this contribution to The EMBO Journal and congratulations on a successful publication! Please consider us again in the future for your most exciting work.

Best regards,

Daniel Klimmeck

Daniel Klimmeck, PhD
Senior Editor
The EMBO Journal
EMBO
Postfach 1022-40
Meyerhofstrasse 1
D-69117 Heidelberg
contact@embojournal.org

*****
Please note that it is The EMBO Journal policy for the transcript of the editorial process (containing referee reports and your response letters) to be published as an online supplement to each paper. If you should prefer removal of any referee-only figures included in the point-by-point response(s), e.g. because they may still be used for future publication or because they have been reproduced from published work by others, please do let us know immediately via response email.
More information is available here: https://link.springer.com/partners/embo-press/editorial-policies#Peer%20review

** Click here to be directed to your login page: https://emboj.msubmit.net

EMBO Press Author Checklist

Corresponding Author Name: Héctor Peinado and Laura Nogués	
Journal Submitted to: The EMBO Journal	
Manuscript Number: EMBOJ-2025-121919-T	

USEFUL LINKS FOR COMPLETING THIS FORM
The EMBO Journal - Author Guidelines
EMBO Reports - Author Guidelines
Molecular Systems Biology - Author Guidelines
EMBO Molecular Medicine - Author Guidelines

Reporting Checklist for Life Science Articles (updated January
This checklist is adapted from Materials Design Analysis Reporting (MDAR) Checklist for Authors. MDAR establishes a minimum set of requirements in transparent reporting in the life sciences (see Statement of Task: 10.31222/osf.io/9sm4x). Please follow the journal's guidelines in preparing your
Please note that a copy of this checklist will be published alongside your article.

Abridged guidelines for figures
1. Data
The data shown in figures should satisfy the following conditions:
- the data were obtained and processed according to the field's best practice and are presented to reflect the results of the experiments in an accurate and unbiased manner.
- ideally, figure panels should include only measurements that are directly comparable to each other and obtained with the same assay.
- plots include clearly labeled error bars for independent experiments and sample sizes. Unless justified, error bars should not be shown for technical
- if n<5, the individual data points from each experiment should be plotted. Any statistical test employed should be justified.
- Source Data should be included to report the data underlying figures according to the guidelines set out in the authorship guidelines on Data

2. Captions
Each figure caption should contain the following information, for each panel where they are relevant:
- a specification of the experimental system investigated (eg cell line, species name).
- the assay(s) and method(s) used to carry out the reported observations and measurements.
- an explicit mention of the biological and chemical entity(ies) that are being measured.
- an explicit mention of the biological and chemical entity(ies) that are altered/varied/perturbed in a controlled manner.
- the exact sample size (n) for each experimental group/condition, given as a number, not a range;
- a description of the sample collection allowing the reader to understand whether the samples represent technical or biological replicates (including how many animals, litters, cultures, etc.).
- a statement of how many times the experiment shown was independently replicated in the laboratory.
- definitions of statistical methods and measures:
  - common tests, such as t-test (please specify whether paired vs. unpaired), simple χ2 tests, Wilcoxon and Mann-Whitney tests, can be unambiguously identified by name only, but more complex techniques should be described in the methods section;
  - are tests one-sided or two-sided?
  - are there adjustments for multiple comparisons?
  - exact statistical test results, e.g., P values = x but not P values < x;
  - definition of 'center values' as median or average;
  - definition of error bars as s.d. or s.e.m.

Please complete ALL of the questions below.
Select "Not Applicable" only when the requested information is not relevant for your study.

Materials

Newly Created Materials	Information included in the manuscript?	In which section is the information available? (Reagents and Tools Table, Materials and Methods, Figures, Data Availability Section)
New materials and reagents need to be available; do any restrictions apply?	Not Applicable	

Antibodies	Information included in the manuscript?	In which section is the information available? (Reagents and Tools Table, Materials and Methods, Figures, Data Availability Section)
For **antibodies** provide the following information:<br>- Commercial antibodies: RRID (if possible) or supplier name, catalogue number and or/clone number<br>- Non-commercial: RRID or citation	Yes	In Methods sections: Multiparameter Immunohistochemistry Staining of TMAs, Immunoblotting, Histological analyses, Immunofluorescence, Spheroids formation and 3D collagen I invasion assay, RhoA-GTP pulldown assay

DNA and RNA sequences	Information included in the manuscript?	In which section is the information available? (Reagents and Tools Table, Materials and Methods, Figures, Data Availability Section)
**Short novel DNA or RNA including primers, probes**: provide the sequences.	Yes	In the methods section "CRISPR editing and shRNA tools" and "Quantitative qRT-PCR"

Cell materials	Information included in the manuscript?	In which section is the information available? (Reagents and Tools Table, Materials and Methods, Figures, Data Availability Section)
**Cell lines:** Provide species information, strain. Provide accession number in repository **OR** supplier name, catalog number, clone number, and/**OR** RRID.	Yes	In the Methods section "Cell lines and reagents"
**Primary cultures:** Provide species, strain, sex of origin, genetic modification status.	Not Applicable	
Report if the cell lines were recently **authenticated** (e.g., by STR profiling) and tested for mycoplasma contamination.	Yes	In the Methods section "Cell lines and reagents"

Experimental animals	Information included in the manuscript?	In which section is the information available? (Reagents and Tools Table, Materials and Methods, Figures, Data Availability Section)
**Laboratory animals or Model organisms:** Provide species, strain, sex, age, genetic modification status. Provide accession number in repository **OR** supplier name, catalog number, clone number, **OR** RRID.	Yes	In the Methods section "Mice"
**Animal observed in or captured from the field:** Provide species, sex, and age where possible.	Not Applicable	
Please detail **housing and husbandry conditions**.	Yes	In the Methods section"Mice" and "Ethical Compliance"

Plants and microbes	Information included in the manuscript?	In which section is the information available? (Reagents and Tools Table, Materials and Methods, Figures, Data Availability Section)
**Plants:** provide species and strain, ecotype and cultivar where relevant, unique accession number if available, and source (including location for collected wild specimens).	Not Applicable	
**Microbes:** provide species and strain, unique accession number if available, and source.	Not Applicable	

Human research participants	Information included in the manuscript?	In which section is the information available? (Reagents and Tools Table, Materials and Methods, Figures, Data Availability Section)
If collected and within the bounds of privacy constraints report on age, sex and gender or ethnicity for all study participants.	Yes	In the Methods section Patient samples and Tissue microarray (TMA), Public transcriptomic analyses and Ethical Compliance and in the Table EV1 and Table EV2.

Core facilities	Information included in the manuscript?	In which section is the information available? (Reagents and Tools Table, Materials and Methods, Figures, Data Availability Section)
If your work benefited from core facilities, was their service mentioned in the acknowledgments section?	Yes	Acknowledgements section

Design

Study protocol	Information included in the manuscript?	In which section is the information available? (Reagents and Tools Table, Materials and Methods, Figures, Data Availability Section)

If study protocol has been **pre-registered, provide DOI in the manuscript**. For clinical trials, provide the trial registration number **OR** cite DOI.	Not Applicable	
Report the **clinical trial registration number** (at ClinicalTrials.gov or equivalent), where applicable.	Not Applicable	

Laboratory protocol	Information included in the manuscript?	In which section is the information available? (Reagents and Tools Table, Materials and Methods, Figures, Data Availability Section)
Provide DOI OR other citation details if **external detailed step-by-step protocols** are available.	Not Applicable	

Experimental study design and statistics	Information included in the manuscript?	In which section is the information available? (Reagents and Tools Table, Materials and Methods, Figures, Data Availability Section)
Include a statement about **sample size** estimate even if no statistical methods were used.	Yes	In the Methods sections "Mice" and "Statistical Analysis"
Were any steps taken to minimize the effects of subjective bias when allocating animals/samples to treatment (e.g. **randomization procedure**)? If yes, have they been described?	Yes	In the Methods sections "Mice"
Include a statement about **blinding** even if no blinding was done.	Yes	In the Methods sections "Mice"
Describe **inclusion/exclusion criteria** if samples or animals were excluded from the analysis. Were the criteria pre-established?<br><br>If sample or data points were omitted from analysis, report if this was due to attrition or intentional exclusion and provide justification.	Yes	
For every figure, are **statistical tests** justified as appropriate? Do the data meet the assumptions of the tests (e.g., normal distribution)? Describe any methods used to assess it. Is there an estimate of variation within each group of data? Is the variance similar between the groups that are being statistically compared?	Yes	In the Methos section "Statistical Analysis" and in the Figure Legends.

Sample definition and in-laboratory replication	Information included in the manuscript?	In which section is the information available? (Reagents and Tools Table, Materials and Methods, Figures, Data Availability Section)
In the figure legends: state number of times the experiment was **replicated** in laboratory.	Yes	In the Figure Legends
In the figure legends: define whether data describe **technical or biological replicates**.	Yes	In the Figure Legends

Ethics

Ethics	Information included in the manuscript?	In which section is the information available? (Reagents and Tools Table, Materials and Methods, Figures, Data Availability Section)
Studies involving **human participants**: State details of **authority granting ethics approval** (IRB or equivalent committee(s), provide reference number for approval.	Yes	In the Methods section "Ethical Compliance"
Studies involving **human participants**: Include a statement confirming that **informed consent** was obtained from all subjects and that the experiments conformed to the principles set out in the WMA Declaration of Helsinki and the Department of Health and Human Services Belmont Report.	Yes	In the Methods section "Ethical Compliance"
Studies involving **human participants: For** publication of **patient photos**, include a statement confirming that consent to publish was obtained.	Not Applicable	
Studies involving experimental **animals**: State details of **authority granting ethics approval** (IRB or equivalent committee(s), provide reference number for approval. Include a statement of compliance with ethical regulations.	Yes	In the Methods section "Ethical Compliance"
Studies involving **specimen and field samples:** State if relevant **permits** obtained, provide details of authority approving study; if none were required, explain why.	Not Applicable	

Dual Use Research of Concern (DURC)	Information included in the manuscript?	In which section is the information available? (Reagents and Tools Table, Materials and Methods, Figures, Data Availability Section)
Could your study fall under dual use research restrictions? Please check biosecurity documents and list of **select agents and toxins** (CDC): https://www.selectagents.gov/sat/list.htm	Not Applicable	
If you used a select agent, is the security level of the lab appropriate and reported in the manuscript?	Not Applicable	
If a study is subject to dual use research of concern regulations, is the name of the **authority granting approval and reference number** for the regulatory approval provided in the manuscript?	Not Applicable	

Reporting

The MDAR framework recommends adoption of discipline-specific guidelines, established and endorsed through community initiatives. Journals have their own policy about requiring specific guidelines and recommendations to complement MDAR.

Adherence to community standards	Information included in the manuscript?	In which section is the information available? (Reagents and Tools Table, Materials and Methods, Figures, Data Availability Section)
State if relevant guidelines or checklists (e.g., **ICMJE, MIBBI, ARRIVE, PRISMA**) have been followed or provided.	Not Applicable	
For **tumor marker prognostic studies,** we recommend that you follow the **REMARK** reporting guidelines (see link list at top right). See author guidelines, under 'Reporting Guidelines'. Please confirm you have followed these guidelines.	Not Applicable	
For **phase II and III randomized controlled trials**, please refer to the **CONSORT** flow diagram (see link list at top right) and submit the CONSORT checklist (see link list at top right) with your submission. See author guidelines, under 'Reporting Guidelines'. Please confirm you have submitted this list.	Not Applicable	

Data Availability

Data availability	Information included in the manuscript?	In which section is the information available? (Reagents and Tools Table, Materials and Methods, Figures, Data Availability Section)
Have **primary datasets** been deposited according to the journal's guidelines (see 'Data Deposition' section) and the respective accession numbers provided in the Data Availability Section?	Not Applicable	
Were **human clinical and genomic datasets** deposited in a public access-controlled repository in accordance to ethical obligations to the patients and to the applicable consent agreement?	Not Applicable	
Are **computational models** that are central and integral to a study available without restrictions in a machine-readable form? Were the relevant accession numbers or links provided?	Not Applicable	
If publicly available data were reused, provide the respective **data citations in the reference list**.	Yes	In the Methods section "Data availability" and in the Figure Legends of Fig. 3g, 7h-i and Fig. EV 2m)
